# Cascade Transformation of the Ansamycin Benzoquinone Core into Benzoxazole Influencing Anticancer Activity and Selectivity

**DOI:** 10.1021/acs.joc.3c00493

**Published:** 2023-06-05

**Authors:** Natalia Skrzypczak, Krystian Pyta, Wiktor Bohusz, Aleksandra Leśniewska, Maria Gdaniec, Piotr Ruszkowski, Wojciech Schilf, Franz Bartl, Piotr Przybylski

**Affiliations:** † Faculty of Chemistry, Adam Mickiewicz University, Uniwersytetu Poznańskiego 8, 61-614 Poznań, Poland; ‡ Institute of Organic Chemistry, Polish Academy of Sciences, Kasprzaka 44/52, 01-224 Warsaw, Poland; § Department of Pharmacology, Poznań University of Medical Sciences, Rokietnicka 5a, 60-806 Poznań, Poland; ∥ Lebenswissenschaftliche Fakultät, Institut für Biologie, Biophysikalische Chemie Humboldt-Universität zu Berlin, Invalidenstraße 42, 10115 Berlin, Germany

## Abstract

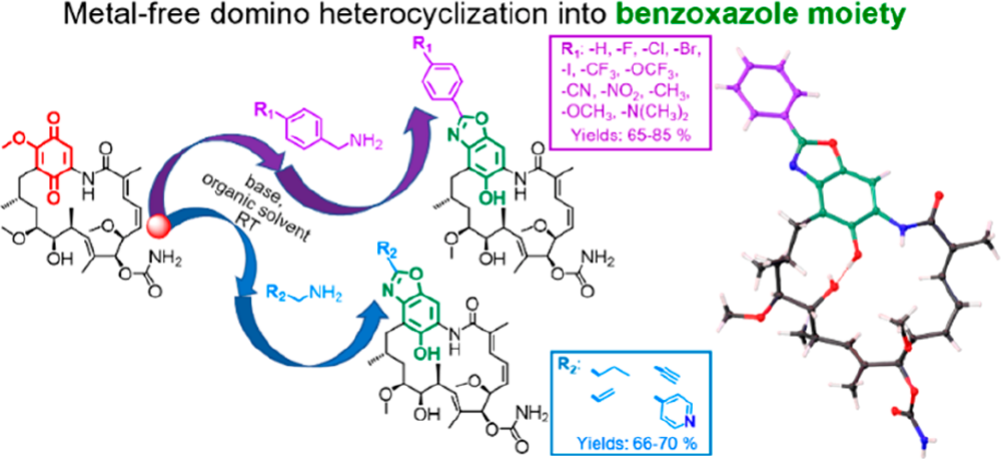

The metal-free cascade transformation of geldanamycin benzoquinone core is proposed at relatively mild conditions. This approach yields new benzoxazole ansamycin antibiotics and enables their functionalization in an atom-economic manner, irrespective of the type of amine used. The analysis of the heterocyclization course reveals the dependence of its rate on the nature of the *para*-substituent within the benzylamine moiety (EDG/EWG) and the strength of the base. The reduction of the ansamycin core enables an increase in anticancer potency and selectivity.

All ansamycins are built from the lactam-containing flexible ansa bridge that links two nonadjacent positions of the relatively rigid core, e.g., benzenoid, naphthalenoid or atypical.^1−4^ Geldanamycin (**GDM**, Figure 1) is a natural product that belongs to the benzoquinone ansamycins isolated from *Streptomyces hygroscopicus var. geldanus*.^5^**GDM** and its C(17)-congeners are known for their high anticancer effects, as they bind to the *N*-terminal domain (NBD) of heat shock protein named chaperone (Hsp90).^6^ In the search for new anticancer agents, **GDM** was modified mainly within the benzoquinone portion at C(17) and C(19).^7−12^ Some of the ansamycins containing a benzene core showed enough promising anticancer activities to be considered in clinical trials.^13^ Therefore, many different approaches involving semisynthetic, mutasynthetic, and genetic manipulations have been applied to benzenoid ansamycins in order to reduce the quinone, improve useful biological potency, and decrease toxicity.^9,14−22^ Modifications of the **GDM** core via the fusion of additional rings, imidazole, morpholine, benzo[*g*]quinoxaline, benzoxazine, oxazolidine, and tetrahydrodiazepine at C(17)–C(18), were rarely accompanied by reduction of the quinone core. Moreover, reported synthetic strategies did not enable further tailoring of the structure of the attached substituent to the core toward interactions with the target (heat shock protein Hsp90).^23,24^ Modern synthetic strategies affording benzoxazole systems are based mainly on bifunctional reactants.^25−34^ Unfortunately, these approaches allow the transformation of bifunctional reactants via intermolecular reactions utilizing metal catalysts, which is in contradiction to green chemistry rules.^35,36^ To the best of our knowledge, benzoxazoles have never been obtained via an intramolecular and metal-free strategy directly from amino-benzoquinones as a starting material, whereas approaches with metal catalysts are very widespread.^37−41^

**Figure 1:**
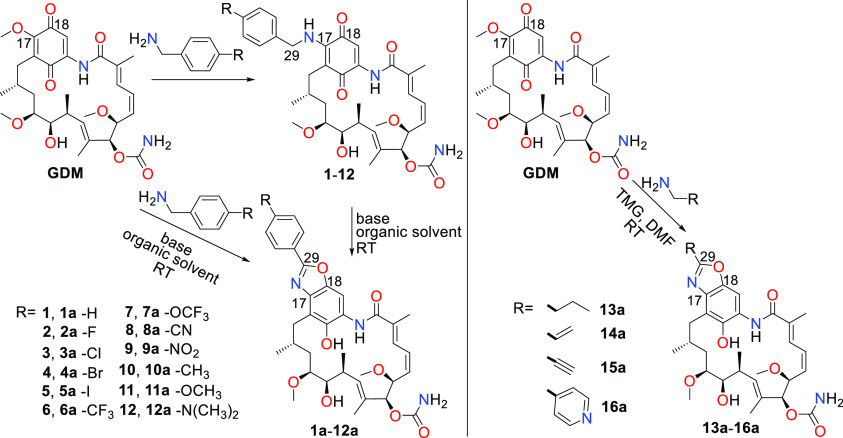
Two-step and cascade synthetic approaches leading to new benzoxazole derivatives of **GDM**.

In order to synthesize **1**–**12** derivatives^23,42^ we applied our well-established protocol with TEA as the base (Figure 1, left).^12^ However, under these reaction conditions, with **GDM** with 4-cyanobenzylamine or 4-nitrobenzylamine as reactants, other products than the expected **8** and **9** were formed, respectively (Figure 1, left). Instead of the expected C(17)-benzylamine products, the benzoxazole derivatives **8a** and **9a** were formed via domino transformations, involving addition/elimination followed by heterocyclization of the benzoquinone (Figure 1, left). To get a deeper insight into the reaction course we attempted to obtain **1a**–**12a** in a two-step approach. Isolated derivatives **1**–**12**, containing either electron-withdrawing (EWG) or electron-donating (EDG) groups, were treated with a base to yield derivatives **1a**–**12a** (Figure 1). Structures of these products, bearing a benzoxazole ring as a new core, were confirmed by NMR (**1a**–**12a**, Supporting Information) and X-ray methods (**1a**–**11a**, Figure 2, Figures S2–S12). The NMR spectra of **1a**–**11a** revealed conformational equilibria in solution (see Supplementary Data), whereby the arrangement of the ansa bridge relative to the core in the structure of predominant conformers **1a**–**12a** in solution is similar to those in **GDM** and most of C(17)-amine analogs in solid (Figure 2, Figure S1). Conformational lability of the ansa bridge in solution is characteristic for a whole family of ansamycins as we reported earlier.^43^ The predominant conformation of the ansa-bridge with *trans*-lactam for **1a**–**12a** in solution is different from the arrangement of the ansa bridge with the *cis*-lactam, crucial for binding of ansamycins to the Hsp90 and achieved via the atropisomerization process.^5,9,10,12^ In the structures of **1a**–**12a** in crystal, a newly formed benzoxazole ring is nearly coplanar with the phenyl substituent, whereas the C(21)–OH phenol group is intramolecularly stabilized via H-bond with the C(11)–OH hydroxyl of the ansa bridge (Figure 2, Figures S1–S12).

**Figure 2:**
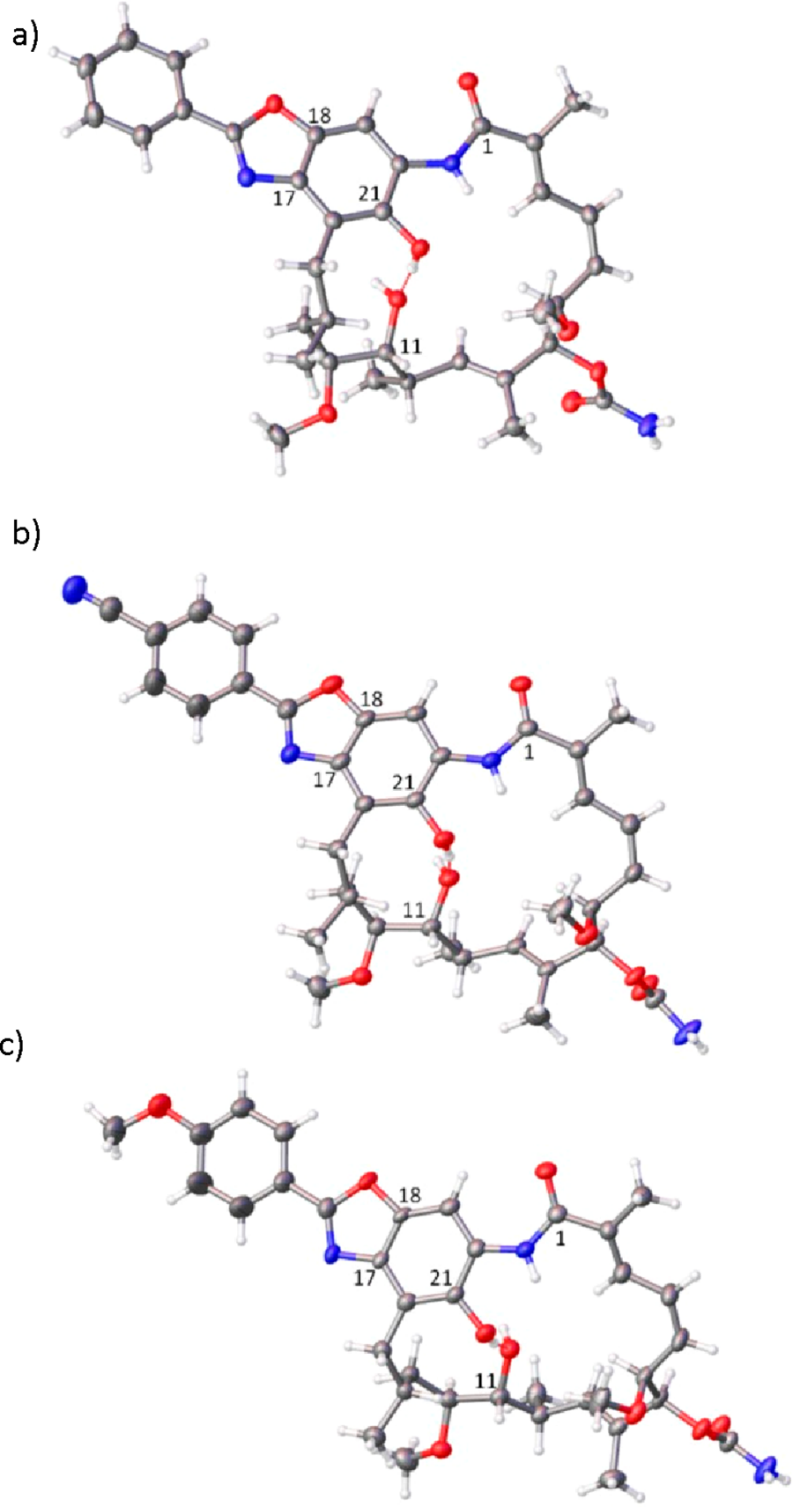
Selected X-ray structures of novel ansamycins bearing benzoxazole cores: (a) **1a** (CCDC 2155685); (b) **8a** (CCDC 2155691); (c) **11a** (CCDC 2155694).

To optimize the heterocyclization conditions, simple benzyl derivative **1** was tested with various base–solvent systems (Table 1, Tables S1 and S2). These tests indicated that the most effective solvent for the heterocyclization is DMF used at room temperature (Table S1). In turn, the highest conversion of **1** (>99%) and yield of **1a** (65–80%) were observed after 1 h for strong organic bases in DMF, wherein the p*K*_a_^MeCN^ values of the base were >23 (for P_1_–H, TMG, and TMGN; Table 1). Interestingly, these strong organic bases, used at the stoichiometric amount relative to **1** in DMF, were efficient in transforming **1** into **1a** in up to 1 h. In turn, the use of these bases in, e.g., 2-fold excess markedly shortened the reaction time. The use of weaker aliphatic organic bases (TEA, quinuclidine, DMAP) resulted in a low reactant conversion after a few days, even when the base was used in a high excess relative to **1**. Moreover, for inorganic bases, such as NaH and K_2_CO_3_ used in DMF, the conversion was also high (90–99%), although yields were lower (64–74%) and reaction times were longer (1.5–24 h, Table 1). Thus, the heterocyclization rate and yield of **1** into **1a** are strongly dependent on the used solvent and the strength of the selected base.

**Table 1:** Tests with Various Bases Required for Heterocyclization of **1** in DMF at Room Temperature

	Yield of **1a** (%)/conversion of **1** (%)				
Base^a^	0.5 h	1 h	1.5 h	24 h	96 h
NaH	34/54	40/62	51/72	64/90	–
K_2_CO_3_	35/59	60/83	74/>99	–	–
TEA	–	<1/2	–	21/27	43/50
Quinuclidine	3/5	5/8	8/10	25/30	–
DMAP	–	<1/2	–	11/19	27/36
TMG	90/>99	90/>99	–	–	–
DBU	71/83	80/95	–	–	–
TMGN	67/89	79/98	–	–	–
TBD	73/84	75/88	–	–	–
P_1_–H	80/>99	80/>99	–	–	–

a Structures of all bases are given in Table S1.

In order to evaluate the influence of the benzylamine *para*-substituents of different nature (EWG, EDG) on the rate of conversion **1**–**12** into **1a**–**12a**, tests under the earlier-optimized conditions (TMG, molar ratio 1:1, 0 °C, DMF; Table S2, Figure 3) were performed. These experiments revealed three groups of substituents exhibiting different effects on the reactant consumption as follows: acceleration (**3**–**9**), neutral (**1**, **2**), and slowing down (**10**–**12**). The use of **1** resulted in ca. 70% conversion after 20 min (Figure 3, Table S2). The most beneficial substituents at the C(17)-benzyl moiety were those classified as electron-withdrawing ones (**3**–**9**) since, after 20 min, 85–98% of conversion was observed. The changes in the conversion over a wide range were caused by the EWG, which acted through the inductive (**3**–**7**) and/or by resonance (**8**, **9**) effects. Furthermore, those of resonance-assisted EWGs appeared to be more favorable for a faster conversion of reactants, compared to inductive EWGs. This observation is in contrast with data obtained for EDGs in **10**–**12**, for which the reaction rate was notably slowed down after 20 min (54–59%, Table S2, Figure 3). The presence of fluorine in the benzylamine moiety (**2**) contributes to a conversion rate which is comparable to that of an unsubstituted benzyl moiety (**1**, Figure S18). As mentioned above (Table 1), the strength of the base is crucial for the initiation of the quinone core heterocyclization, irrespective of the type of substituent in the benzylamine reactant (EDG/EWG, Figure 4). Thus, our tests showed that the conversion of **GDM** into **1a**–**12a** is feasible and efficient when the used base is strong enough. As indicated by LC-MS mechanism studies (Figures S19–S22), the first step of this transformation is a deprotonation of the N(17)–H group assisted by a partial reduction of the benzoquinone (A, Figure 4). The key step of transforming the benzoquinone into benzoxazole is tautomerization of A into B (Figure 4). This reaction enables the formation of a conjugated diaryl system via an imine moiety (B), allowing the total reduction of the quinone. The rate of this transformation depends on the nature of the *para*-substituent (R; Figure 4) in the benzylamine. This substituent is crucial because it can promote or hinder the proton transfer of the C(29)H_2_ benzyl position to the electron-rich oxygen O(18). The role of the R-substituent is related to the stabilization of the carbanion, which is initially formed from structure A at the C(29). This stabilization is the most efficient when a *para*-EWG is present within the benzylamine moiety, irrespective of the resonance (−NO_2_, −CN) or inductive (−Cl, −Br, −I, −CF_3_, −OCF_3_) effects. The −NO_2_ and −CN groups are the most favorable for the core aromatization as they enable the most efficient negative charge delocalization via the resonance. The nucleophilic attack of the O(18)^−^ phenolate group on the imine moiety C(29)=N (C, Figure 4) affords a partially saturated heterocyclic core (D and E, Figure 4). The spontaneous oxidation in the air yields benzoxazole in ansamycin scaffolds **1a**–**12a** (F, Figure 4).

**Figure 3:**
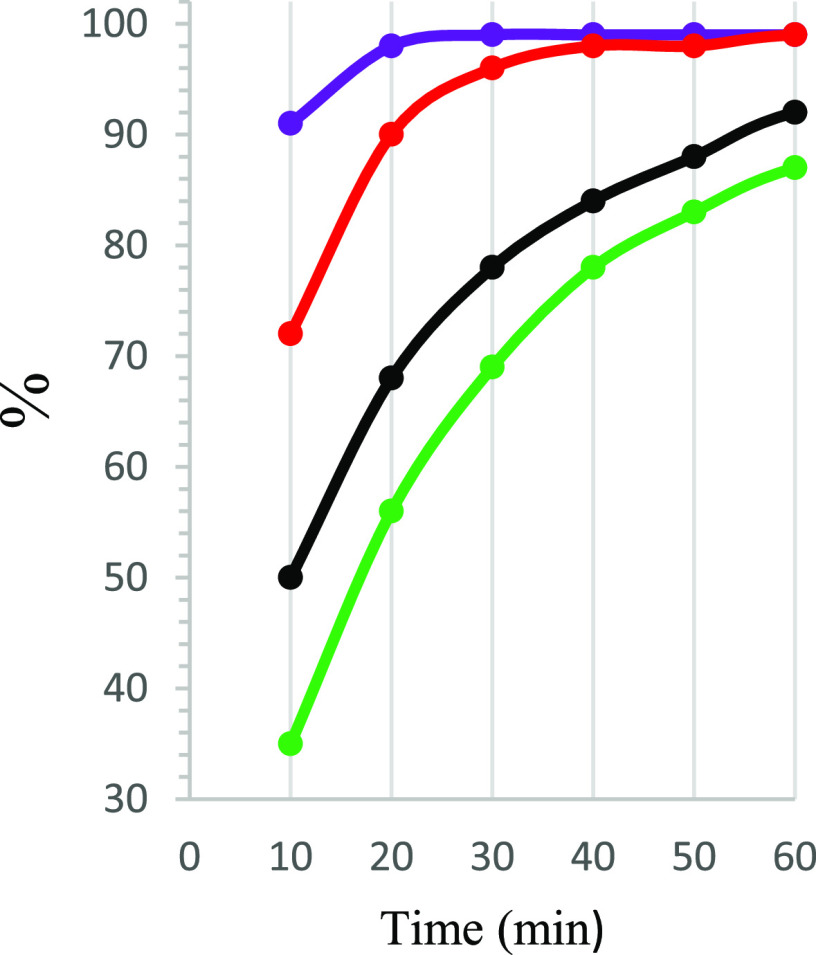
Progress of the substrate conversion [%] per unit of time [min] for **1** (black), **6** (red), **8** (purple), and **10** (green).

**Figure 4:**
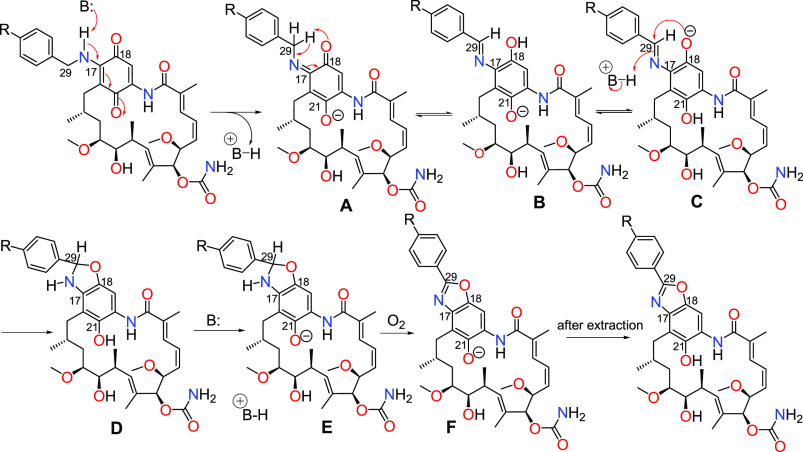
Plausible mechanism of transformation C(17)-amine congeners of **GDM** into novel benzoxazole ansamycin derivatives.

To investigate the utility of our heterocyclization approach toward obtaining the other functionalized benzoxazole cores, reactions with *n*-butylamine, allylamine, propargylamine, and pyridin-4-ylmethanamine (Figure 1, right, **13a**–**16a**) were performed. The one-pot reaction at room temperature was slow for *n*-butylamine (reaction time ∼ 24 h), whereas for those reactants where C*sp*^2^ carbon is linked to the −CH_2_–NH_2_ group, a significant increase in reaction rate was observed (reaction time ∼ 6 h). The studies on the reaction scope revealed that the installation of different-type substituents at the benzoxazole core of ansamycins is feasible using our metal-free, cascade protocol under mild conditions.

**GDM** and its derivatives were studied in several cancer cell lines (SKBR-3, SKOV-3, and PC-3) and in normal cells (Human dermal fibroblasts HDF, Table S4). Overall, the transformation of **GDM** into its simple amine (**1**–**12**) and benzoxazole (**1a**–**12a**) derivatives increased the lipophilicity (ilogP ≥ 3.5; Table S4). The analysis of the anticancer activity indicates that compounds **2**, **7**, **3**, **3a**, **4a**, **6a, 9a**, **12**, and **12a** showed comparable anticancer potency (IC_50_ = 0.71–0.99 μM, Table S4) with **GDM**. Compound **2** showed markedly better selectivity indices (SI ∼ 3, Table S4) than **GDM**. Considering derivatives **12** and **12a** of similar anticancer potencies (IC_50_ < 1 μM), formation of the benzoxazole ring improves selectivity (SI ∼2.3), also relative to toxic **GDM** (SI ∼1.7) showing the lowest ilogP = 3.04 (Table S4). A comparison of selectivities (SI) for pairs **1**/**1a**, **3**/**3a**, **4**/**4a**, **10**/**10a**, **11**/**11a**, indicates that the heterocyclization slightly improves this parameter (Table S4). Furthermore, data in Table S4 reveal that, by heterocyclization of the ansamycin, an increase in anticancer potency is noted in most cases, if compared to **GDM** nonheterocyclic derivatives. This result can be helpful in future designing Hsp90 inhibitors, which are not susceptible to conjugation with glutathione (toxic effect) as it takes place for **GDM**.

A newly developed and optimized cascade method for reductive heterocyclization of the benzoquinone ansamycin core yielded novel benzoxazole derivatives **1a**–**16a**. This metal-free synthetic approach with the use of an organic base is the most efficient utilizing relatively mild reaction conditions (aprotic solvents; rt or lower temperatures). The proposed cascade reaction offers the installation of different-type substituents at the new benzoxazole core of ansamycins, whereby the rate of this reaction is dependent on the presence/absence of the C*sp*^2^ carbon that is linked to the −CH_2_–NH_2_ moiety in the amine reactant. Furthermore, in the case of benzyl amines as reactants, the presence of EWG groups in the aromatic ring, revealing mesomeric and inductive effects, guarantee the shortest reaction times in obtaining the benzoxazole core. Our domino protocol enables optimization of the ansamycin scaffold relative to the molecular target (Hsp90) and gives a promising perspective in view of increasing the selectivity indices.

## Data Availability

The data underlying this study are available in the published article and its Supporting Information.

## Supporting Information Available

Experimental procedures, progress of the substrate conversion for heterocyclization reaction (Tables S1 and S2, Figure S18) and monitoring of the reaction mechanism (Figures S19–S22); biological data (Table S4) and assays details, copy of ^1^H, ^13^C NMR, FT-IR and ESI MS spectra (Figures S23–S88) and spectral assignments, X-ray structural details of **1a**–**11a** (Figures S1–S17; Table S3). (PDF)

## References

[ref1] Skrzypczak N.; Przybylski P. Modifications, Biological Origin and Antibacterial Activity of Naphthalenoid Ansamycins. Nat. Prod. Rep. 2022, 39, 1653 10.1039/D2NP00002D. 35244668

[ref2] Skrzypczak N.; Przybylski P. Structural Diversity and Biological Relevance of Benzenoid and Atypical Ansamycins and Their Congeners. Nat. Prod. Rep. 2022, 39, 1678 10.1039/D2NP00004K. 35262153

[ref3] Kirschning A.; Harmrolfs K.; Knobloch T. The Chemistry and Biology of the Maytansinoid Antitumor Agents. Comptes Rendus Chimie 2008, 11 (11), 1523–1543. 10.1016/j.crci.2008.02.006.

[ref4] Funayama S.; Cordell G. A. Ansamycin AntibioticsA Discovery, Classification, Biosynthesis and Biological Activities. In Studies in Natural Products Chemistry; Elsevier: 2000; Vol. 23, pp 51–106. 10.1016/S1572-5995(00)80127-1.

[ref5] Franke J.; Eichner S.; Zeilinger C.; Kirschning A. Targeting Heat-Shock-Protein 90 (Hsp90) by Natural Products: Geldanamycin, a Show Case in Cancer Therapy. Nat. Prod. Rep. 2013, 30 (10), 1299–1323. 10.1039/c3np70012g. 23934201

[ref6] Chen F.; Zhu C.; Jiang H. [3 + 1+1] Annulation Reaction of Benzo-1,2-Quinones, Aldehydes and Hydroxylamine Hydrochloride: Access to Benzoxazoles with Inorganic Nitrogen Source. Advanced Synthesis & Catalysis 2021, 363 (8), 2124–2132. 10.1002/adsc.202001521.

[ref7] Deboer C.; Meulman P. A.; Wnuk R. J.; Peterson D. H. GELDANAMYCIN, A NEW ANTIBIOTIC. J. Antibiot. 1970, 23 (9), 442–447. 10.7164/antibiotics.23.442. 5459626

[ref8] Schnur R. C.; Corman M. L.; Gallaschun R. J.; Cooper B. A.; Dee M. F.; Doty J. L.; Muzzi M. L.; DiOrio C. I.; Barbacci E. G. ErbB-2 Oncogene Inhibition by Geldanamycin Derivatives: Synthesis, Mechanism of Action, and Structure-Activity Relationships. J. Med. Chem. 1995, 38 (19), 3813–3820. 10.1021/jm00019a011. 7562912

[ref9] Kitson R. R. A.; Chang C.-H.; Xiong R.; Williams H. E. L.; Davis A. L.; Lewis W.; Dehn D. L.; Siegel D.; Roe S. M.; Prodromou C.; Ross D.; Moody C. J. Synthesis of 19-Substituted Geldanamycins with Altered Conformations and Their Binding to Heat Shock Protein Hsp90. Nat. Chem. 2013, 5 (4), 307–314. 10.1038/nchem.1596. 23511419 PMC3839848

[ref10] Kitson R. R. A.; Moody C. J. An Improved Route to 19-Substituted Geldanamycins as Novel Hsp90 Inhibitors – Potential Therapeutics in Cancer and Neurodegeneration. Chem. Commun. 2013, 49 (76), 8441 10.1039/c3cc43457e. PMC3835074 23770604

[ref11] Tian Z.-Q.; Liu Y.; Zhang D.; Wang Z.; Dong S. D.; Carreras C. W.; Zhou Y.; Rastelli G.; Santi D. V.; Myles D. C. Synthesis and Biological Activities of Novel 17-Aminogeldanamycin Derivatives. Bioorg. Med. Chem. 2004, 12 (20), 5317–5329. 10.1016/j.bmc.2004.07.053. 15388159

[ref12] Skrzypczak N.; Pyta K.; Ruszkowski P.; Gdaniec M.; Bartl F.; Przybylski P. Synthesis, Structure and Anticancer Activity of New Geldanamycin Amine Analogs Containing C(17)- or C(20)- Flexible and Rigid Arms as Well as Closed or Open Ansa-Bridges. Eur. J. Med. Chem. 2020, 202, 112624 10.1016/j.ejmech.2020.112624. 32663707

[ref13] Biamonte M. A.; Van de Water R.; Arndt J. W.; Scannevin R. H.; Perret D.; Lee W.-C. Corrections to Heat Shock Protein 90: Inhibitors in Clinical Trials. J. Med. Chem. 2010, 53 (5), 2332–2332. 10.1021/jm100114u. 20055425

[ref14] Eichner S.; Floss H. G.; Sasse F.; Kirschning A. New, Highly Active Nonbenzoquinone Geldanamycin Derivatives by Using Mutasynthesis. ChemBioChem. 2009, 10 (11), 1801–1805. 10.1002/cbic.200900246. 19554593

[ref15] Knobloch T.; Harmrolfs K.; Taft F.; Thomaszewski B.; Sasse F.; Kirschning A. Mutational Biosynthesis of Ansamitocin Antibiotics: A Diversity-Oriented Approach to Exploit Biosynthetic Flexibility. ChemBioChem. 2011, 12 (4), 540–547. 10.1002/cbic.201000608. 22238146

[ref16] Hermane J.; Bułyszko I.; Eichner S.; Sasse F.; Collisi W.; Poso A.; Schax E.; Walter J.-G.; Scheper T.; Kock K.; Herrmann C.; Aliuos P.; Reuter G.; Zeilinger C.; Kirschning A. New, Non-Quinone Fluorogeldanamycin Derivatives Strongly Inhibit Hsp90. ChemBioChem. 2015, 16 (2), 302–311. 10.1002/cbic.201402375. 25572106

[ref17] Jürjens G.; Kirschning A. Synthesis of a Cytotoxic Ansamycin Hybrid. Org. Lett. 2014, 16 (11), 3000–3003. 10.1021/ol5011278. 24874463

[ref18] Skrzypczak N.; Pyta K.; Ruszkowski P.; Mikołajczak P.; Kucińska M.; Murias M.; Gdaniec M.; Bartl F.; Przybylski P. Anticancer Activity and Toxicity of New Quaternary Ammonium Geldanamycin Derivative Salts and Their Mixtures with Potentiators. Journal of Enzyme Inhibition and Medicinal Chemistry 2021, 36 (1), 1898–1904. 10.1080/14756366.2021.1960829. 34344239 PMC8344233

[ref19] Pyta K.; Skrzypczak N.; Ruszkowski P.; Bartl F.; Przybylski P. Regioselective Approach to Colchiceine Tropolone Ring Functionalization at C(9) and C(10) Yielding New Anticancer Hybrid Derivatives Containing Heterocyclic Structural Motifs. Journal of Enzyme Inhibition and Medicinal Chemistry 2022, 37 (1), 597–605. 10.1080/14756366.2022.2028782. 35067138 PMC8788354

[ref20] Hug J. J.; Krug D.; Müller R. Bacteria as Genetically Programmable Producers of Bioactive Natural Products. Nat. Rev. Chem. 2020, 4 (4), 172–193. 10.1038/s41570-020-0176-1. 37128046

[ref21] Kitson R. R. A.; Moody C. J. Synthesis of Novel Geldanamycin Derivatives. Tetrahedron 2021, 82, 131927 10.1016/j.tet.2021.131927.

[ref22] Eichner S.; Knobloch T.; Floss H. G.; Fohrer J.; Harmrolfs K.; Hermane J.; Schulz A.; Sasse F.; Spiteller P.; Taft F.; Kirschning A. The Interplay between Mutasynthesis and Semisynthesis: Generation and Evaluation of an Ansamitocin Library. Angew. Chem., Int. Ed. 2012, 51 (3), 752–757. 10.1002/anie.201106249. 22135226

[ref23] Schnur R. C.; Corman M. L.; Gallaschun R. J.; Cooper B. A.; Dee M. F.; Doty J. L.; Muzzi M. L.; Moyer J. D.; DiOrio C. I. Inhibition of the Oncogene Product P185erbB-2 in Vitro and in Vivo by Geldanamycin and Dihydrogeldanamycin Derivatives. J. Med. Chem. 1995, 38 (19), 3806–3812. 10.1021/jm00019a010. 7562911

[ref24] Ge J.; Normant E.; Porter J. R.; Ali J. A.; Dembski M. S.; Gao Y.; Georges A. T.; Grenier L.; Pak R. H.; Patterson J.; Sydor J. R.; Tibbitts T. T.; Tong J. K.; Adams J.; Palombella V. J. Design, Synthesis, and Biological Evaluation of Hydroquinone Derivatives of 17-Amino-17-Demethoxygeldanamycin as Potent, Water-Soluble Inhibitors of Hsp90. J. Med. Chem. 2006, 49 (15), 4606–4615. 10.1021/jm0603116. 16854066

[ref25] Sattar R.; Mukhtar R.; Atif M.; Hasnain M.; Irfan A. Synthetic Transformations and Biological Screening of Benzoxazole Derivatives: A Review. J. Heterocyclic Chem. 2020, 57 (5), 2079–2107. 10.1002/jhet.3944.

[ref26] Osmaniye D.; Korkut Çelikateş B.; Sağlık B. N.; Levent S.; Acar Çevik U.; Kaya Çavuşoğlu B.; Ilgın S.; Özkay Y.; Kaplancıklı Z. A. Synthesis of Some New Benzoxazole Derivatives and Investigation of Their Anticancer Activities. Eur. J. Med. Chem. 2021, 210, 112979 10.1016/j.ejmech.2020.112979. 33183865

[ref27] Rajasekhar S.; Maiti B.; Chanda K. A Decade Update on Benzoxazoles, a Privileged Scaffold in Synthetic Organic Chemistry. Synlett 2017, 28 (05), 521–541. 10.1055/s-0036-1588671.

[ref28] Ferlin F.; van der Hulst M. K.; Santoro S.; Lanari D.; Vaccaro L. Continuous Flow/Waste-Minimized Synthesis of Benzoxazoles Catalysed by Heterogeneous Manganese Systems. Green Chem. 2019, 21 (19), 5298–5305. 10.1039/C9GC01641D.

[ref29] Tiwari A. R.; Bhanage B. M. Copper-Catalyzed Synthesis of Benzoxazoles via Tandem Cyclization of 2-Halophenols with Amidines. Org. Biomol. Chem. 2016, 14 (33), 7920–7926. 10.1039/C6OB01264G. 27480248

[ref30] Ptiček L.; Hok L.; Grbčić P.; Topić F.; Cetina M.; Rissanen K.; Pavelić S. K.; Vianello R.; Racané L. Amidino Substituted 2-Aminophenols: Biologically Important Building Blocks for the Amidino-Functionalization of 2-Substituted Benzoxazoles. Org. Biomol. Chem. 2021, 19 (12), 2784–2793. 10.1039/D1OB00235J. 33704342

[ref31] Gao S.; Gao L.; Meng H.; Luo M.; Zeng X. Iron-Catalyzed Synthesis of Benzoxazoles by Oxidative Coupling/Cyclization of Phenol Derivatives with Benzoyl Aldehyde Oximes. Chem. Commun. 2017, 53 (71), 9886–9889. 10.1039/C7CC04965J. 28825080

[ref32] Endo Y.; Bäckvall J.-E. Biomimetic Oxidative Coupling of Benzylamines and 2-Aminophenols: Synthesis of Benzoxazoles. Chemistry – A European Journal 2012, 18 (43), 13609–13613. 10.1002/chem.201202187. 22968931

[ref33] Gevondian A. G.; Kotovshchikov Y. N.; Latyshev G. V.; Lukashev N. V.; Beletskaya I. P. Domino Construction of Benzoxazole-Derived Sulfonamides via Metal-Free Denitrogenation of 5-Iodo-1,2,3-Triazoles in the Presence of SO2 and Amines. J. Org. Chem. 2021, 86 (8), 5639–5650. 10.1021/acs.joc.1c00115. 33822625

[ref34] Kotovshchikov Y. N.; Latyshev G. V.; Navasardyan M. A.; Erzunov D. A.; Beletskaya I. P.; Lukashev N. V. Annulation-Induced Cascade Transformation of 5-Iodo-1,2,3-Triazoles to 2-(1-Aminoalkyl)Benzoxazoles. Org. Lett. 2018, 20 (15), 4467–4470. 10.1021/acs.orglett.8b01755. 30040429

[ref35] Geogheghan K. Medal for Metal-Free Methods. Nat. Chem. 2021, 13 (12), 1163–1163. 10.1038/s41557-021-00851-7. 34811472

[ref36] Poliakoff M.; Licence P. Green Chemistry. Nature 2007, 450 (7171), 810–812. 10.1038/450810a. 18064000

[ref37] Yang B.; Hu W.; Zhang S. Synthesis of Benzoxazoles via an Iron-Catalyzed Domino C–N/C–O Cross-Coupling Reaction. RSC Adv. 2018, 8 (5), 2267–2270. 10.1039/C7RA13080E. 35541485 PMC9077259

[ref38] Viirre R. D.; Evindar G.; Batey R. A. Copper-Catalyzed Domino Annulation Approaches to the Synthesis of Benzoxazoles under Microwave-Accelerated and Conventional Thermal Conditions. J. Org. Chem. 2008, 73 (9), 3452–3459. 10.1021/jo702145d. 18376860

[ref39] Evindar G.; Batey R. A. Parallel Synthesis of a Library of Benzoxazoles and Benzothiazoles Using Ligand-Accelerated Copper-Catalyzed Cyclizations of Ortho-Halobenzanilides. J. Org. Chem. 2006, 71 (5), 1802–1808. 10.1021/jo051927q. 16496964

[ref40] Peng L.; Hu Z.; Zhao Y.; Peng L.; Xu Z.; Yin S.-F.; Tang Z.; Qiu R.; Kambe N. One-Pot Synthesis of Phosphorylnaphth[2,1-d]Oxazoles and Products as P,N-Ligands in C–N and C–C Formation. Org. Biomol. Chem. 2022, 20 (20), 4110–4114. 10.1039/D2OB00565D. 35551357

[ref41] Lohmann U.; Hartke K. Unabhängige Synthese der violetten Farbstoffe aus 1,2-Naphthochinon-4-sulfonsäure und primären aliphatischen Aminen sowie eines Nebenproduktes. Arch. Pharm. Pharm. Med. Chem. 1984, 317 (4), 313–323. 10.1002/ardp.19843170407.

[ref42] Li Z.; Jia L.; Wang J.; Wu X.; Hao H.; Xu H.; Wu Y.; Shi G.; Lu C.; Shen Y. Design, Synthesis and Biological Evaluation of 17-Arylmethylamine-17-Demethoxygeldanamycin Derivatives as Potent Hsp90 Inhibitors. Eur. J. Med. Chem. 2014, 85, 359–370. 10.1016/j.ejmech.2014.07.101. 25105924

[ref43] Pyta K.; Janas A.; Skrzypczak N.; Schilf W.; Wicher B.; Gdaniec M.; Bartl F.; Przybylski P. Specific Interactions between Rifamycin Antibiotics and Water Influencing Ability To Overcome Natural Cell Barriers and the Range of Antibacterial Potency. ACS Infect. Dis. 2019, 5 (10), 1754–1763. 10.1021/acsinfecdis.9b00176. 31461259

