# Supplementary material for: Cascade Transformation of the Ansamycin Benzoquinone Core into Benzoxazole Influencing Anticancer Activity and Selectivity
Source: J Org Chem. 2023 Jun 5;88(13):9469–74. doi: 10.1021/acs.joc.3c00493 (PMC10337034; doi:10.1021/acs.joc.3c00493)
Supplement: Supplementary file 1 — jo3c00493_si_001.pdf [file jo3c00493_si_001.pdf]

Supporting Info

Cascade transformation of the ansamycin benzoquinone core into benzoxazole influencing anticancer activity and selectivity

Natalia Skrzypczak[a], Krystian Pyta[a, d], Wiktor Bohusz[a], Aleksandra Leśniewska[a], Maria Gdaniec[a], Piotr Ruszkowski[c], Wojciech Schilf[b], Franz Bartl[d] and Piotr Przybylski[a]*

[a] Faculty of Chemistry, Adam Mickiewicz University, Uniwersytetu Poznańskiego 8, 61-614 Poznań, Poland [piotrp@amu.edu.pl]
[b] Institute of Organic Chemistry, Polish Academy of Sciences, Kasprzaka 44/52, 01-224 Warsaw, Poland
[c] Department of Pharmacology, Poznań University of Medical Sciences, Rokietnicka 5a, 60-806 Poznań, Poland
[d] Lebenswissenschaftliche Fakultät, Institut für Biologie, Biophysikalische Chemie Humboldt-Universität zu Berlin Invalidenstraße 42, Berlin, Germany

*corresponding author e-mail: piotrp@amu.edu.pl

Supporting Info SI-1
HPLC measurements: SI-6
FT-IR measurements: SI-6
NMR measurements: SI-6
ESI MS analyses: SI-7
Elemental analyses: SI-7
X-ray crystal structure analyses: SI-7
Biological assays SI-8
General synthetic procedures: SI-9
Cyclization reaction tests: SI-10
Spectral characteristic of a **GDM** analogs **1**-**12**: SI-10
Spectral characteristic of a **GDM** analogs **1a**-**16a**: SI-15

Table S1 Cyclization reaction tests, presented for the amine derivative of **GDM**, which in position C(17) contains an aminobenzyl substituent, leading to the formation of an oxazole ring using various organic and inorganic bases as catalysts, and carried out in various solvents. SI-22

Table S2 Cyclization reaction tests, presented for the amine derivatives (**1-12**) of **GDM**, leading to the formation of an oxazole ring using TMG as catalysts, and carried out in DMF (0°C). SI-24

Table S3 Crystal data and refinement details SI-25

Table S4 Comparison of biological activity between **GDM**, benzoxazole (**1a-12a**) and C(17)-amine derivatives (**1-12**) in cancer cell lines (SKBR-3, SKOV-3, PC-3) and in healthy cell line (HDF); the activity expressed as IC_{50} [μM]; selectivity indices (SI) are given in square brackets SI-27

Figure S18 Progress of the substrate conversion [%] per unit of time [min] for derivatives **1a-11a**. The colors and shapes were assigned appropriately to the derivatives: **1a**-black square, **2a**-pink square, **3a**-red triangle, **4a**-blue triangle, **6a**-green triangle, **7a**-purple triangle, **8a**-orange dot, **9a**-blue dot, **10a**-green diamond, **11a**-purple diamond, **12a**-orange diamond. SI-38

References: SI-104

Figure S1 Overlay of the geldanamycin (CCDC 1988068)[7] (red) and **2a** (blue) molecules (rms = 0.233 Å) in conformations found in their crystal structures. Intramolecular O-H···O hydrogen bond in **2a** is shown with a dashed line. Only N-H and O-H hydrogen atoms are shown. SI-29
Figure S2 The asymmetric unit of **1a**. Displacement ellipsoids are shown at the 50% probability level. SI-30
Figure S3 The asymmetric unit of **2a**. Displacement ellipsoids are shown at the 50% probability level. SI-30
Figure S4 The asymmetric unit of **3a**. Displacement ellipsoids are shown at the 50% probability level. The isopropanol molecule and the substituent at C(29) are disordered. 31
Figure S5 The asymmetric unit of **4a**. Displacement ellipsoids are shown at the 50% probability level. The isopropanol molecule and the substituent at C(29) are disordered. SI-31
Figure S6 The asymmetric unit of **5a**. Displacement ellipsoids are shown at the 50% probability level. The isopropanol molecule and the substituent at C(29) are disordered. SI-32

Figure S7 The asymmetric unit of **6a**. Displacement ellipsoids for the ordered part of the molecule are shown at the 50% probability level. The substituent at C(29) shown as a 'ball and stick' model is disordered over three sites. The isopropanol is shown with dashed bonds. SI-32
Figure S8 The asymmetric unit of **7a**. Displacement ellipsoids are shown at the 50% probability level. The substituent at C(29) is disordered over three sites and only one site with the major occupancy factor is shown in the diagram. SI-33
Figure S9 The asymmetric unit of **8a**. Displacement ellipsoids are shown at the 50% probability level. SI-33
Figure S10 The asymmetric unit of **9a**. Displacement ellipsoids are shown at the 50% probability level. The substituent at C(29) is disordered over two sites. The isopropanol molecule has a partial occupancy. SI-34
Figure S11 The asymmetric unit in **10a**. Displacement ellipsoids are shown at the 50% probability level. The isopropanol molecule and the substituent at C(29) are disordered. SI-34
Figure S12 The asymmetric unit of **11a**. Displacement ellipsoids are shown at the 50% probability level. The substituent at C(29) is disordered over three sites and only one site with the major occupancy factor is shown in the diagram. The isopropanol solvent molecule has a partial occupancy. SI-35
Figure S13 View of the 2D assembly *via* hydrogen bonds in **2a**. This structural motif is observed in all studied crystal structures. SI-35
Figure S14 Crystal packing of **2a** shown in the projection along the b axis. Hydrogen bonded isopropanol molecules form a chain extended along the b axis. SI-36
Figure S15 Crystal packing of **7a** shown in the projection along the b axis. SI-36
Figure S16 Crystal packing of **8a** shown in the projection along the b axis. The isopropanol solvent molecule in the site with the major occupation is not hydrogen-bonded to the host molecules.... SI-37
Figure S17 Crystal packing of **10a** shown in the projection along the b axis. Isopropanol molecules are hydrogen boded to the host molecules. SI-37
Figure S18 Progress of the substrate conversion [%] per unit of time [min] for derivatives **1a-11a**. The colors and shapes were assigned appropriately to the derivatives: **1a**-black square, **2a**-pink square, **3a**-red triangle, **4a**-blue triangle, **6a**-green triangle, **7a**-purple triangle, **8a**-orange dot, **9a**-blue dot, **10a**-green diamond, **11a**-purple diamond, **12a**-orange diamond. SI-38
Figure S19 HPLC chromatogram recorded for the reaction mixture during conversion **2** to **2a**, captured with intermediates (**2'**and **2"**). UV-vis and MS spectrum for the product of the first intermediate **2'**. SI-39
Figure S20 HPLC chromatogram recorded for the reaction mixture during conversion **2** to **2a**, captured with intermediates (**2'** and **2"**). UV-vis and MS spectrum for the product **2a**. SI-40
Figure S21 HPLC chromatogram recorded for the reaction mixture during conversion **2** to **2a**, captured with intermediates (**2'** and **2"**). UV-vis and MS spectrum for substrate **2**. SI-41
Figure S22 HPLC chromatogram recorded for the reaction mixture during conversion **2** to **2a**, captured with intermediates (**2'**and **2"**). UV-vis and MS spectrum for the product of the second intermediate **2"**. SI-42
Figure S23 ^{1}H and $^{13}C\{1H\}$ spectra of compound **1** in $CDCl_3$. SI-43
Figure S24 FT-IR spectrum of compound **1** (in KBr). SI-44
Figure S25 ^{1}H and $^{13}C\{1H\}$ spectra of compound **2** in $CDCl_3$. SI-45
Figure S26 FT-IR spectrum of compound **2** (in KBr). SI-46
Figure S27 ^{1}H and $^{13}C\{1H\}$ spectra of compound **3** in $CDCl_3$. SI-47
Figure S28 FT-IR spectrum of compound **3** (in KBr). SI-48
Figure S29 ^{1}H and $^{13}C\{1H\}$ spectra of compound **4** in $CDCl_3$. SI-49
Figure S30 FT-IR spectrum of compound **4** (in KBr). SI-50

Figure S31 ^1H and $^{13}C\{1H\}$ spectra of compound **5** in $CDCl_3$. SI-51
Figure S32 FT-IR spectrum of compound **5** (in KBr). SI-52
Figure S33 ^1H and $^{13}C\{1H\}$ spectra of compound **6** in $CDCl_3$. SI-53
Figure S34 FT-IR spectrum of compound **6** (in KBr). SI-54
Figure S35 ^1H and $^{13}C\{1H\}$ spectra of compound **7** in $CDCl_3$. SI-55
Figure S36 FT-IR spectrum of compound **7** (in KBr). SI-56
Figure S37 ^1H and $^{13}C\{1H\}$ spectra of compound **8** in $CDCl_3$. SI-57
Figure S38 FT-IR spectrum of compound **8** (in KBr). SI-58
Figure S39 ^1H and $^{13}C\{1H\}$ spectra of compound **9** in $CDCl_3$. SI-59
Figure S40 FT-IR spectrum of compound **9** (in KBr). SI-60
Figure S41 ^1H and $^{13}C\{1H\}$ spectra of compound **10** in $CDCl_3$. SI-61
Figure S42 FT-IR spectrum of compound **10** (in KBr). SI-62
Figure S43 ^1H and $^{13}C\{1H\}$ spectra of compound **11** in $CDCl_3$. SI-63
Figure S44 FT-IR spectrum of compound **11** (in KBr). SI-64
Figure S45 ^1H and $^{13}C\{1H\}$ spectra of compound **12** in $CDCl_3$. SI-65
Figure S46 FT-IR spectrum of compound **12** (in KBr). SI-66
Figure S47 ^1H and $^{13}C\{1H\}$ spectra of compound **1a** in DMSO-d_6+ ACN-d_3. .... SI-67
Figure S48 FT-IR spectrum of compound **1a** (in KBr). SI-68
Figure S49 ^1H and $^{13}C\{1H\}$ spectra of compound **2a** in DMSO-d_6+ ACN-d_3. .... SI-69
Figure S50 FT-IR spectrum of compound **2a** (in KBr). SI-70
Figure S51 ^1H and $^{13}C\{1H\}$ spectra of compound **3a** in DMSO-d_6+ ACN-d_3. .... SI-71
Figure S52 FT-IR spectrum of compound **3a** (in KBr). SI-72
Figure S53 ^1H and $^{13}C\{1H\}$ spectra of compound **4a** in DMSO-d_6+ ACN-d_3. .... SI-73
Figure S54 FT-IR spectrum of compound **4a** (in KBr). SI-74
Figure S55 ^1H and $^{13}C\{1H\}$ spectra of compound **5a** in DMSO-d_6+ ACN-d_3. .... SI-75
Figure S56 FT-IR spectrum of compound **5a** (in KBr). SI-76
Figure S57 ^1H and $^{13}C\{1H\}$ spectra of compound **6a** in DMSO-d_6+ ACN-d_3. .... SI-77
Figure S58 FT-IR spectrum of compound **6a** (in KBr). SI-78
Figure S59 ^1H and $^{13}C\{1H\}$ spectra of compound **7a** in DMSO-d_6+ ACN-d_3. .... SI-79
Figure S60 FT-IR spectrum of compound **7a** (in KBr). SI-80
Figure S61 ^1H and $^{13}C\{1H\}$ spectra of compound **8a** in DMSO-d_6+ ACN-d_3. .... SI-81
Figure S62 FT-IR spectrum of compound **8a** (in KBr). SI-82
Figure S63 ^1H and $^{13}C\{1H\}$ spectra of compound **9a** in DMSO-d_6+ ACN-d_3. .... SI-83
Figure S64 FT-IR spectrum of compound **9a** (in KBr). SI-84
Figure S65 ^1H and $^{13}C\{1H\}$ spectra of compound **10a** in DMSO-d_6+ ACN-d_3. .... SI-85
Figure S66 FT-IR spectrum of compound **10a** (in KBr). SI-86
Figure S67 ^1H and $^{13}C\{1H\}$ spectra of compound **11a** in DMSO-d_6+ ACN-d_3. .... SI-87
Figure S68 FT-IR spectrum of compound **11a** (in KBr). SI-88
Figure S69 ^1H and $^{13}C\{1H\}$ spectra of compound **12a** in DMSO-d_6+ ACN-d_3. .... SI-89
Figure S70 ^1H-^1H COSY spectrum of compound **12a** in DMSO-d_6+ ACN-d_3. .... SI-90
Figure S71 ^1H-^{13}C HSQC spectrum of compound **12a** in DMSO-d_6+ ACN-d_3. .... SI-90
Figure S72 ^1H-^{13}C HMBC spectra of compound **12a** in DMSO-d_6+ ACN-d_3. .... SI-91
Figure S73 FT-IR spectrum of compound **12a** (in KBr). SI-92
Figure S74 ^1H and $^{13}C\{1H\}$ spectra of compound **13a** in DMSO-d_6+ ACN-d_3. .... SI-93
Figure S75 ^1H-^1H COSY spectra of compound **13a** in DMSO-d_6+ ACN-d_3. .... SI-94
Figure S76 ^1H-^{13}C HSQC spectra of compound **13a** in DMSO-d_6+ ACN-d_3. .... SI-95
Figure S77 ^1H-^{13}C HMBC spectra of compound **13a** in DMSO-d_6+ ACN-d_3. .... SI-96
Figure S78 FT-IR spectrum of compound **13a** (in KBr). SI-97

Figure S79 ESI-MS spectrum of compound **13a**. SI-97
Figure S80 ^{1}H and ^{13}C{1H} spectra of compound **14a** in DMSO-d_6+ ACN-d_3. .... SI-98
Figure S81 FT-IR spectrum of compound **14a** (in KBr). SI-99
Figure S82 ESI-MS spectrum of compound **14a**. SI-99
Figure S83 ^{1}H and ^{13}C{1H} spectra of compound **15a** in DMSO-d_6+ ACN-d_3. .... SI-100
Figure S84 FT-IR spectrum of compound **15a** (in KBr). SI-101
Figure S85 ESI-MS spectrum of compound **15a**. SI-101
Figure S86 ^{1}H and ^{13}C{1H} spectra of compound **16a** in DMSO-d_6+ ACN-d_3. .... SI-102
Figure S87 FT-IR spectrum of compound **16a** (in KBr). SI-103
Figure S88 ESI-MS spectrum of compound **16a**. SI-103

General Experimental:

Geldanamycin was purchased from Carbosynth, batch number AG236511801. Solvents CD_3CN, DMSO-d_6, $CDCl_3$ for NMR spectroscopic measurements as well as, methanol, acetone, Dimethyl sulfoxide (DMSO), N,N-Dimethylformamide (DMF), Acetonitrile (ACN), Triethylamine (TEA), Tetrahydrofuran (THF), NaH K_2CO_3, quinuclidine, 4-(Dimethylamino)pyridine (DMAP), 1,1,3,3-Tetramethylguanidine (TMG), 1,8-Diazabicyclo[5.4.0]undec-7-ene (DBU), 1,8-Bis(tetramethylguanidino)naphthalene (TMGN), 1,5,7-Triazabicyclo[4.4.0]dec-5-ene (TBD), Imino-tris(dimethylamino)phosphorane (Phosphazene base P_1-H), benzylamine, 4-Fluorobenzylamine, 4-Chlorobenzylamine, 4-Bromobenzylamine, 4-Iodobenzylamine, 4-Methylbenzylamine, 4-Methoxybenzylamine, 4-(Trifluoromethyl)benzylamine, 4-(Trifluoromethoxy)benzylamine, 4-Cyanobenzylamine, 4-(Dimethylamino)benzylamine, 4-Nitrobenzylamine, butylamine, allylamine, propargylamine and pyridin-4-ylmethanamine for the syntheses of **GDM** derivatives were purchased from Sigma-Aldrich. While methanol, methylene chloride and acetone used for column chromatography were purchased from Chemsolve. H_2O HPLC gradient grade and CH_3CN HPLC gradient grade were purchased from J. T. Baker.

HPLC measurements:

The purity of the **GDM** analogs **1-12**, **1a-12a** was determined by HPLC method using Dionex Ultimate 3000 equipped with an LPG-3400 SD gradient pump using Thermo GOLD C18 150×4.6 mm (5 μm) and Accucore XL column, TCC-3000SD thermostat to columns (column temp. equal 25 ºC) and Dionex VWD- 3400RS variable wavelength UV-vis detector (detection at λ_{max}=220, 260 and 341 nm); the flow rates were 0.5 mL/min (for **1-12**) and 0.75 mL/min (for **1a-12a**) with injection volumes of 10 μL in acetonitrile mixtures and the mobile phase: 35:65 H_2O/CH_3CN.

The reaction progress of the **GDM** analogs **13a-16a** was determined by HPLC method using Dionex Ultimate 3000 equipped with an LPG-3400 SD gradient pump using X-bridge C8 150×3.0 mm (3.5 μm) column, TCC-3000SD thermostat to columns (column temp. equal 25 ºC) and Dionex VWD- 3400RS variable wavelength UV-vis detector (detection at λ_{max}=220, 260 and 341 nm); the flow rates were 0.75 mL/min with injection volumes of 5 μL in acetonitrile/methanol mixtures and the mobile phase: 55:45 H_2O/CH_3CN.

FT-IR measurements:

The FT-IR spectra of **GDM** analogs **1-12** and **1a-12a** were recorded in the KBr pellet. FT-IR measurements were performed at a spectrometer equipped with a DTGS detector and two-columnar purge gas generator at resolution 1 cm^{-1}, NSS = 150, range 4000-400 cm^{-1}. The Happ-Genzel apodization function was used.

NMR measurements:

The ^1H and ^{13}C measurements of derivatives of **GDM** (**1-12**) were performed in $CDCl_3$ using a 600 MHz and 500 MHz Bruker BioSpin GmBH spectrometer. The operating frequencies for ^1H measurements were 600.25 MHz; example parameters: spectral width, sw = 6.3636; acquisition time at = 2.8443 s; relaxation delay d_1 = 1.0 s; T = 298.0 K; TMS was used as the internal standard. No window function or zero filling were used. The digital resolution was

0.2 Hz/point. ^{13}C NMR spectra were recorded at the operating frequency 150.95 MHz; sw = 40650.4 Hz, d_1 = 1.0 s, T = 298.0 K, and TMS as the internal standard. Line broadening parameters of 0.5 or 1 Hz were applied. ^{1}H and ^{13}C NMR resonances unambiguously assigned based on the $^{1}H–^{13}C$ HMBC, $^{1}H–^{13}C$ HSQC, and $^{1}H–^{1}H$ COSY couplings. The ^{1}H and ^{13}C measurements of derivatives of **GDM** (**1a-12a**) were performed in ACN-d_3+DMSO-d_6 using a 500 MHz Varian-Agilent spectrometer. The operating frequencies for ^{1}H measurements were 499.83 MHz; example parameters: spectral width, sw = 8012.8; acquisition time at = 4.000 s; relaxation delay d_1 = 1.0 s; T = 243.15 K; TMS was used as the internal standard. No window function or zero filling were used. The digital resolution was 0.2 Hz/point. ^{13}C NMR spectra were recorded at the operating frequency 125.7 MHz; sw = 37878.8 Hz, d_1 = 1.0 s, T = 243.15 K, and TMS as the internal standard. Line broadening parameters of 0.5 or 1 Hz were applied. ^{1}H and ^{13}C NMR resonances unambiguously assigned based on the $^{1}H–^{13}C$ HMBC, $^{1}H–^{13}C$ HSQC, and $^{1}H–^{1}H$ COSY couplings.

ESI MS analyses:

The mass spectra were recorded on ZQ Waters spectrometer using Electrospray ionization method. (range of m/z range from 100 to 2000).

Elemental analyses:

The elemental analyses of new **GDM** analogs (**1-12** and **1a-12a**) were carried out on Vario ELIII (Germany).

X-ray crystal structure analyses:

Compounds **1a-11a** were poorly soluble in most organic solvents. Single crystals suitable for X-ray analysis were obtained by slow cooling of their isopropanol solutions. All diffraction experiments were carried out at 130 K with an Oxford Diffraction SuperNova diffractometer using mirror-monochromated Cu Kα radiation. Diffraction data were processed with the CrysAlis PRO software.[1] Most of the crystals showed extended shape of reflections resulting in poor resolution of the diffraction data. The structures were solved with SHELXT[2] and refined by full-matrix least-squares on F^2 with SHELXL-2018[3] within Olex2.[4] The assumed absolute structure of the studied compounds conforms with the known absolute configuration of geldanamycin. The crystal structures of compounds **1a-11a** form two isostructural series, both belonging to the $P2_1$ space group. The first group consists of **1a** and **2a** that crystallize as solvates with two isopropanol molecules in the asymmetric unit. All crystal components of these crystals are ordered. The structures were refined with anisotropic displacement parameters for non-hydrogen atoms. Hydrogen atoms were placed in idealized positions, except some hydroxyl H atoms which were located in electron density difference maps and had their coordinates refined. In the second group the asymmetric unit cell consists of one molecule of the ansamycin derivative **3a-11a** and a varied amount of isopropanol molecules. Apart from **8a** (cyano derivative), in all other cases disorder of the benzoxazole and its aromatic substituent was observed. This substituent was located in two or three different sites as a result of small rotation around the two bonds connecting the *ansa* chain and the benzoxazole fragment. The benzoxazole aromatic substituent and solvent isopropanol molecules are packed together in a wide channel running along the *b* axis. In the case of trifluoromethoxy derivative **7a,** with the largest substituent in the para-position of the benzene ring, no solvent molecules were identified. In the structures of **3a, 4a, 8a, 10a** with relatively small groups in the para-position of the benzene ring (CH_3, Cl, Br CN) there is one molecule of isopropanol in the asymmetric unit. In case of larger substituents in the para-position, the amount of isopropanol in the asymmetric unit changes from 0.85 for **11a** (OCH_3),

through 0.70 for **9a** (NO_2) to 0.46 for **6a** (CF_3). The solvent molecules are also disordered over two or three sites. In the refinement process numerous restraints and constraints were imposed on the geometry, displacement parameters and occupancy factors of the disordered fragments.

Crystal data and the refinement details are collected in Table 1S. The asymmetric units in the crystal structures of **1a-11a** are shown in Figs. 1S-12S. In all cases the crystal structures are constructed from hydrogen-bonded 2D assemblies shown in Fig. 13S and Figs. 14S-17S. These assemblies closely resemble robust hydrogen-bonded layers found in the geldanamycin structure and in the crystal structures of its C(17) amino derivatives. This fact explains why in the solid state conformationally flexible geldanamycin and its derivatives exhibit little conformational diversity.

Overlay of geldanamycin and **1a** molecules in the conformation adopted in the solid state is shown in Fig. 1S.

CCDC 2155685-2155694 contain the supplementary crystallographic data for this paper. These data can be obtained free of charge via http://www.ccdc.cam.ac.uk/conts/retrieving.html (or from the CCDC, 12 Union Road, Cambridge CB2 1EZ, UK; Fax: +44 1223 336033; E-mail: deposit@ccdc.cam.ac.uk

Biological assays

Human cancer cells SKBR-3 (human breast cancer cell line) and SKOV-3 (ovarian cancer cell line) were cultured in McCoy's Modified Medium. Human cancer cells PC-3 (human prostate cancer cell line) were cultured in an F-12K medium. Human Dermal Fibroblasts cell line (HDF) was cultured in Fibroblast Basal Medium. Each medium was supplemented with 10% fetal bovine serum, 1% L-glutamine, and 1% penicillin/streptomycin solution. The cell lines were kept in the incubator at 37°C. The optimal plating density of cell lines was determined to be 5×10^4. All the cell lines and mediums were obtained from the American Type Culture Collection (ATCC) supplied by LGC-Standards. The protein-staining SRB (Sigma-Aldrich) microculture colourimetric assay, developed by the National Cancer Institute (USA) for in vitro antitumor screening was used in this study, to estimate the cell number by providing a sensitive index of total cellular protein content, being linear to cell density. The monolayer cell culture was trypsinized and the cell count was adjusted to 5×10^4 cells. To each well of the 96 well microtiter plate, 0.1 mL of the diluted cell suspension (approximately 10,000 cells) was added. After 24 hours, when a partial monolayer was formed, the supernatant was washed out and 100 μL of six different compound concentrations (0.1, 0.2, 1, 2, 10, and 20 μM) were added to the cells in microtitre plates. The tested **GDM**, **1-11** and **1a-11a** were dissolved in DMSO (containing 10% of water) (100 μL) and the content of DMSO did not exceed 0.1%; this concentration was found to be nontoxic to the cell lines. The cells were exposed to compounds for 72 hours at 37 °C in a humidified atmosphere (90% RH) containing 5% CO_2. After that, 25 μL of 50% trichloroacetic acid was added to the wells and the plates were incubated for 1 hour at 4°C. The plates were then washed out with distilled water to remove traces of medium and next dried by the air. The air-dried plates were stained with 100 μL of 0.4% sulforhodamine B (prepared in 1% acetic acid) and kept for 30 minutes at room temperature. The unbound dye was removed by rapidly washing with 1% acetic acid and then air dried overnight. The protein-bound dye was dissolved in 100 μL of 10 mM unbuffered Tris base (pH 10.5) for optical density determination at 490 nm. All cytotoxicity experiments were performed three times. Cell survival was Found as the percentage

absorbance compared to the control (nontreated cells). **GDM** was used as the internal standards. Additionally, biological assays were performed in the Human Dermal Fibroblasts cell line (HDF) with the aim to evaluate the cytotoxicity of **GDM** and its **1-11** and **1a-11a** analogues in healthy cells. Results of anticancer studies of novel analogues of **GDM** are shown in Table 2. SI indexes were from equation $SI = IC_{50\ \text{normal cell line HDF}}/IC_{50\ \text{respective cancerous cell line}}$ (Table 2). A beneficial SI > 1.0 indicates a compound with efficacy against cancer cells greater than the toxicity against normal cells.

General synthetic procedures:

The general synthetic procedure of derivatives of **GDM 1−12** together with the analytical data (FT-IR, HRMS, HPLC and elemental data): First, 200 mg (0.36 mmol) of **GDM** was dissolved in a 4.4 mL mixture of THF/MeOH (10:1) and then a four-fold excess of amine was added (1.44 mmol): benzylamine (**1**), 4-Fluorobenzylamine (**2**), 4-Chlorobenzylamine (**3**), 4-Bromobenzylamine (**4**), 4-Iodoobenzylamine (**5**), 4-(Trifluoromethyl)benzylamine (**6**), 4-(Trifluoromethoxy)benzylamine (**7**), 4-Cyanobenzylamine (**8**), 4-Nitrobenzylamine (**9**), 4-Methylbenzylamine (**10**), 4-Methoxybenzylamine (**11**), 4-(Dimethylamino)benzylamine (**12**). Then, to each mixture, 0.5 ml of TEA was added (except **8** and **9**). The mixtures were stirred at 60 °C for a few hours (2-4h) and after that, the solvent was evaporated. Products, derived from respective amine hydrochlorides before evaporation were treated with ethyl acetate and extracted twice with 25 mL of water. All of these products were purified by column chromatography on silica gel (25 cm × 1 cm, silica gel 60, 0.040−0.063 mm/230−400 mesh ASTM, Fluka) with methylene chloride/acetone or methylene chloride/methanol as eluent. After evaporation of the solvent, **1**-**12** derivatives were obtained as violet powder.

The general synthetic procedure of derivatives of **GDM 1a**−**12a** together with the analytical data (FT-IR, HRMS, HPLC and elemental data): First, 100 mg (1 eq.) **GDM** amine analogs (**1-12**) was dissolved in 3 mL DMF and then a 1.1 eq. of TMG was added. The mixtures were stirred at rt. for one hour and after that, the products before evaporation were treated with ethyl acetate and extracted twice with 200 mL of water. All of these products were purified by column chromatography on silica gel (25 cm × 1 cm, silica gel 60, 0.040−0.063 mm/230−400 mesh ASTM, Fluka) with methylene chloride/acetone or methylene chloride/methanol as eluent. After evaporation of the solvent, **1a-12a** derivatives were obtained as a yellow powder.

The one-pot synthetic procedure of derivative of **8a** and **9a** and from **GDM** together with the analytical data (FT-IR, HRMS, HPLC and elemental data): First, 200 mg (0.36 mmol) of **GDM** was dissolved in a 4.4 mL mixture of THF/MeOH (10:1) and then a four-fold excess of amine was added (1.44 mmol). Then, to each mixture, 0.5 ml of TEA was added. The mixture was stirred at 60 °C for two hours and after that, the solvent was evaporated. Before evaporation was treated with ethyl acetate and extracted twice with 200 mL of water. All of these products were purified by column chromatography on silica gel (25 cm × 1 cm, silica gel 60, 0.040−0.063 mm/230−400 mesh ASTM, Fluka) with methylene chloride/methanol as eluent. After evaporation of the solvent, **8a** and **9a** derivatives were obtained as a yellow powder.

The one-pot synthetic procedure of derivative of **13a**-**16a** from **GDM** together with the analytical data (FT-IR, HRMS, HPLC and elemental data): First, 100 mg (0.18 mmol) of **GDM** was dissolved in a 1 mL of DMF and then a two-fold excess of amine was added (0.36 mmol): butylamine (**13a**), allylamine (**14a**), propargylamine (**15a**) and pyridin-4-ylmethanamine (**16a**). Then, to each mixture, 0.18 mmol of TMG was added. The mixture was stirred to the complete disappearance of GDM and the intermediate. Before evaporation reaction mixture was treated with ethyl acetate and extracted twice with 200 mL of water. All of these products were purified by column chromatography on silica

gel (25 cm × 1 cm, silica gel 60, 0.040−0.063 mm/230−400 mesh ASTM, Fluka) with methylene chloride/methanol as eluent. After evaporation of the solvent, **13a-16a** derivatives were obtained as a yellow powder.

Cyclization reaction tests:

Compound **1** (5 mg, 1 eq.) was dissolved in various solvents (1 mL) depending on the test performed. A stoichiometric amount of various bases has been added (1eq.). All base and solvent combinations are shown in **Table 1S**. The mixture was stirred at room temperature and 5 µL sample of reaction mixture was taken to check of the reaction progress (before injection extraction in the solvent system EtOAc:H_2O was performed, the organic layer was evaporated to dryness and the residue was dissolved in acetonitrile). All HPLC analysis were performed with the use the flow rates were 0.75 mL/min the mobile phase: 35:65 H_2O/CH_3CN.

Compounds **1-12** (5 mg, 1 eq.) was dissolved in the 1 mL of DMF. After cooling the reaction mixture to 0 ºC 1eq. of TMG was added. The mixture was stirred at room temperature every 10 minutes to 80 minutes 5 µL sample of the reaction mixture was taken to check the reaction progress (before injection extraction in the solvent system EtOAc:H_2O was performed, the organic layer was evaporated to dryness and the residue was dissolved in acetonitrile). All HPLC analysis were performed with the use the flow rates were 0.75 mL/min the mobile phase: 35:65 H_2O/CH_3CN. Details of the reactions are shown in **Table 2S**.

Spectral characteristic of a **GDM** analogs **1-12**:
Compound ***1.*** (4*E*,6*Z*,8*S*,9*S*,10*E*,12*S*,13*R*,14*S*,16*R*)-19-(benzylamino)-13-hydroxy-8,14-dimethoxy-4,10,12,16-tetramethyl-3,20,22-trioxo-2-azabicyclo[16.3.1]docosa-1(21),4,6,10,18-pentaen-9-yl carbamate (193 mg, yield 85 %) mp 281-283 ºC, HPLC (OD-H, water/acetonitrile = 35/65, flow rate = 0.5 mL/min, l = 260 nm) tR = 9.461 min (major). Anal. Calcd for $C_{35}H_{45}N_3O_8$: C, 66.12; H, 7.13; N, 6.61. Found: C, 66.14; H, 7.12; N, 6.60. HRMS (MALDI-TOF) m/z: $[M + H]^+$ Calcd for $C_{35}H_{45}N_3O_8$ 635.3207; Found 635.1937. FT-IR (KBr): ν_{as}(N-H)$_{carbamate}$=3487.45 cm^{-1}, ν_s(N-H)$_{carbamate}$=3435.17 cm^{-1}, ν(N-H)$_{lactam}$=3329.74 cm^{-1}, ν(O-H)=3201.94 cm^{-1}, ν_{as}(C-H)=2930.83 cm^{-1}, ν_s(C-H)=2824.87 cm^{-1}, ν(C=O)$_{carbamate}$=1731.60 cm^{-1}, ν(C=O)$_{lactam}$=1692.48 cm^{-1}, ν(C=O)$_{quinone}$=1648.57 cm^{-1}, ν(C=C)=1612.53 cm^{-1} , δ(N-H)$_{lactam}$=1578.07 cm^{-1}, δ(N-H)$_{substituent}$=1580.08 cm^{-1}, δ(N-H)$_{carbamate}$=1489.51 cm^{-1}, ν(C-N)$_{carbamate}$=1373.63 cm^{-1}, ν(C-O-C)$_{carbamate}$=1266.21 cm^{-1}, ν(C-O-C)$_{methoxy}$=1055.05 cm^{-1}, γ(=C-H)$_{ar}$=730.00 cm^{-1}, γ(N-H)$_{carbamate}$=698.68 cm^{-1}. ^1H NMR (600 MHz, $CDCl_3$) δ 9.16 (s, 1H, NH-1), 7.41 – 7.37 (m, 2H, H-32,34), 7.37 – 7.33 (m, 1H, H-33), 7.30 (s, 1H, H-19), 7.29 – 7.26 (m, 2H, H-31,35), 6.96 (d, $J^3_{H-3,H-4}$= 11.6 Hz, 1H, H-3), 6.58 (td, $J^3_{H-3,H-4}$= 11.4 Hz, $J^4_{H-4,H-22}$= 1.1 Hz, 1H, H-4), 6.47 (t, $J^3_{NH-17,H-29}$= 5.5 Hz, 1H, NH-17), 5.90 (d, $J^3_{H-9,H-10}$ = 9.7 Hz, 1H, H-9), 5.88 – 5.84 (m, 1H, H-5), 5.19 (s, 1H, H-7), 4.85 (bs, 2H, NH_2-24), 4.76 (dd, J^2 = 14.5 Hz, $J^3_{NH-17,H-29}$= 5.8 Hz, 1H, H-29), 4.62 (dd, J^2 = 14.5 Hz, $J^3_{NH-17,H-29}$= 5.5 Hz, 1H, H-29), 4.31 (dt, $J^3_{H-6,H-7}$=9.8 Hz, $J^3_{H-4,H-6}$= 1.2 Hz, 1H, H-6), 4.19 (s, 1H, OH-11), 3.58 (ddd, $J^3_{H-11,H-12}$=9.0 Hz, $J^3_{H-10,H-11}$=6.7 Hz, $J^4_{H-11,H-13}$=2.2 Hz, 1H, H-11), 3.44 (dt, $J^3_{H-11,H-12}$= 8.9 Hz, $J^3_{H-12,H-13}$= 2.8 Hz, 1H, H-12), 3.36 (s, 3H, H-27), 3.27 (s, 3H, H-23), 2.74 (dqd, $J^3_{H-9,H-10}$= 9.1 Hz, $J^3_{H-10,H-26}$= 6.8 Hz, $J^4_{H-10,H-25}$= 2.1 Hz, 1H, H-10), 2.66 (d, J^2= 14.1 Hz, 1H, H-15), 2.45 (dd, J^2 = 14.2 Hz, $J^3_{H-14,H-15}$= 10.3 Hz, 1H, H-15), 2.02 (d, $J^4_{H-4,H-22}$= 1.4 Hz, 3H, H-22), 1.80 (d, $J^4_{H-10,H-25}$= 1.3 Hz, 3H, H-25), 1.77 (d, $J^3_{H-12,H-13}$= 3.1 Hz, 2H, H-13), 1.70 (bs, 1H, H-14), 1.02 (d, $J^3_{H-10,H-26}$ = 6.4 Hz, 3H, H-26), 0.99 (d, $J^3_{H-14,H-28}$ = 6.9 Hz, 3H, H-28). dqd - Doublet of Quartet of Doublets; bs - broad singlet
$^{13}C\{1H\}$ NMR (151 MHz, $CDCl_3$) δ 183.9 (C-18), 181.2 (C-21), 168.5 (C-1), 156.2 (C-24), 144.8 (C-17), 141.4 (C-20), 136.8 (C-30), 136.0 (C-5), 135.1 (C-2), 133.9 (C-9), 132.9 (C-8), 129.3* (C-32,34), 128.6 (C-33), 127.9* (C-31,35), 127.1 (C-3), 126.7 (C-4), 109.1 (C-19), 109.0 (C-16), 81.8 (C-7), 81.6 (C-12), 81.4 (C-6), 72.8 (C-11), 57.3 (C-23), 56.9 (C-27), 50.2 (C-29), 35.2 (C-13), 34.6 (C-15), 32.5 (C-10), 28.6 (C-14), 23.1 (C-28), 12.9 (C-22), 12.7 (C-25), 12.5 (C-26).

Compound ***2.*** (4*E*,6*Z*,8*S*,9*S*,10*E*,12*S*,13*R*,14*S*,16*R*)-19-((4-fluorobenzyl)amino)-13-hydroxy-8,14-dimethoxy-4,10,12,16-tetramethyl-3,20,22-trioxo-2-azabicyclo[16.3.1]docosa-1(21),4,6,10,18-pentaen-9-yl carbamate (156 mg, yield 67 %), mp 115-117 ºC, HPLC (OD-H, water/acetonitrile = 35/65, flow rate = 0.5 mL/min, l = 260 nm) tR = 9.195 min (major). Anal. Calcd for $C_{35}H_{44}FN_3O_8$: C, 64.30; H, 6.78; F, 2.91; N, 6.43. Found C, 64.31; H, 6.77; F, 2.90; N, 6.42. HRMS (MALDI-TOF) m/z: $[M + H]^+$ Calcd for $C_{35}H_{44}FN_3O_8$ 653.3112; Found 653.3715. FT-IR (KBr): ν_{as}(N-H)$_{carbamate}$=3507.71 cm^{-1}, ν_s(N-H)$_{carbamate}$=3438.28 cm^{-1}, ν_s(N-H)$_{lactam}$=3330.29 cm^{-1}, ν(O-H) =3204.89 cm^{-1}, ν_{as}(C-H)=2931.34 cm^{-1}, ν_s(C-H)=2825.13 cm^{-1}, ν(C=O)$_{carbamate}$=1733.74 cm^{-1},

ν(C=O)$_{lactam}$=1695.38 cm^{-1}, ν(C=O)$_{quinone}$=1648.40 cm^{-1}, ν(C=C) =1609.45 cm^{-1}, δ(N-H)*$_{lactam,substituent}$=1584.00 cm^{-1}, δ(N-H)$_{carbamate}$=1488.08 cm^{-1}, ν(C-F)= 1373.32 cm^{-1}, ν(C-N)$_{carbamate}$ =1324.11 cm^{-1}, ν(C-O-C)$_{carbamate}$ =1191.72 cm^{-1}, ν(C-O-C)$_{substituent}$ =1100.15 cm^{-1}, ν(C-O-C) $_{methoxy}$ =1054.33 cm^{-1} , γ(=C-H)$_{carbamate}$ =783.84 cm^{-1} , γ(=C-H)$_{ar}$=730.44 cm^{-1}, γ(N-H)$_{carbamate}$=689.15 cm^{-1}. ^{1}H NMR (600 MHz, CDCl$_3$) δ 9.15 (s, 1H, NH-1), 7.30 (s, 1H, H-19), 7.27 – 7.24 (m, 2H, H-31,35), 7.10 – 7.05 (m, 2H, H-32,34), 6.95 (d, $J^3_{H-3,H-4}$= 11.7 Hz, 1H, H-3), 6.58 (td, $J^3_{H-3,H-4}$= 11.4 Hz, $J^4_{H-4,H-22}$= 1.1 Hz, 1H, H-4), 6.38 (t, $J^3_{NH-17,H-29}$= 5.6 Hz, 1H, NH-17), 5.90 – 5.84 (m, 2H, H-5,9), 5.18 (s, 1H, H-7), 4.87 (bs, 2H, NH$_2$-24), 4.72 (dd, J^2 = 14.5 Hz, $J^3_{NH-17,H-29}$= 5.7 Hz, 1H, H-29), 4.59 (dd, J^2 = 14.5 Hz, $J^3_{NH-17,H-29}$= 5.5 Hz, 1H, H-29), 4.31 (dt, $J^3_{H-6,H-7}$=9.8 Hz, $J^3_{H-4,H-6}$= 1.2 Hz, 1H, H-6), 4.11 (bs, 1H, OH-11), 3.57 (ddd, $J^3_{H-11,H-12}$=8.9 Hz, $J^3_{H-10,H-11}$=6.5 Hz, $J^4_{H-11,H-13}$=2.3 Hz, 1H, H-11), 3.44 (dt, $J^3_{H-11,H-12}$= 8.9 Hz, $J^3_{H-12,H-13}$= 2.8 Hz, 1H, H-12), 3.36 (s, 3H, H-27), 3.27 (s, 3H, H-23), 2.74 (tt, $J^3_{H-9,H-10}$= 9.3 Hz, $J^3_{H-10,H-26}$= 6.9 Hz, 1H, H-10), 2.69 – 2.64 (m, 1H, H-15), 2.41 (dd, J^2 = 14.1 Hz, $J^3_{H-14,H-15}$= 10.3 Hz, 1H, H-15), 2.02 (d, $J^4_{H-4,H-22}$= 1.4 Hz, 3H, H-22), 1.79 (d, $J^4_{H-10,H-25}$= 1.4 Hz, 3H, H-25), 1.76 (d, $J^3_{H-12,H-13}$= 3.1 Hz, 2H, H-13), 1.70 (bs, 1H, H-14), 1.01 (d, $J^3_{H-10,H-26}$ = 6.5 Hz, 3H, H-26), 0.99 (d, $J^3_{H-14,H-28}$ = 6.9 Hz, 3H, H-28). bs - broad singlet
^{13}C{1H} NMR (151 MHz, CDCl$_3$) δ 183.9 (C-18), 181.3 (C-21), 168.5 (C-1), 162.8 (d, J^1= 247.5 Hz, C-33), 156.2 (C-24), 144.7 (C-17), 141.3 (C-20), 136.1 (C-5), 135.1 (C-2), 133.8 (C-9), 133.0 (C-8), 132.6 (d, J^4= 3.1 Hz, C-30), 129.7* (d, J^3= 8.2 Hz, C-31,35), 127.1 (C-3), 126.7 (C-4), 116.3* (d, J^2= 21.5 Hz, C-32,34), 109.3 (C-19), 109.0 (C-16), 81.8 (C-7), 81.5 (C-12), 81.3 (C-6), 72.8 (C-11), 57.3 (C-23), 56.9 (C-27), 49.5 (C-29), 35.1 (C-13), 34.6 (C-15), 32.5 (C-10), 28.7 (C-14), 23.1 (C-28), 12.9 (C-22), 12.7 (C-25), 12.5 (C-26).

Compound **3.** (4*E*,6*Z*,8*S*,9*S*,10*E*,12*S*,13*R*,14*S*,16*R*)-19-((4-chlorobenzyl)amino)-13-hydroxy-8,14-dimethoxy-4,10,12,16-tetramethyl-3,20,22-trioxo-2-azabicyclo[16.3.1]docosa-1(21),4,6,10,18-pentaen-9-yl carbamate (172 mg, yield 72 %), mp 201-203 ºC, HPLC (OD-H, water/acetonitrile = 35/65, flow rate = 0.5 mL/min, l = 260 nm) tR = 11.507 min (major). Anal. Calcd for $C_{35}H_{44}ClN_3O_8$: C, 62.73; H, 6.62; Cl, 5.29; N, 6.27. Found C, 62.72; H, 6.63; Cl, 5.28; N, 6.28. HRMS (MALDI-TOF) m/z: [M + H]$^+$ Calcd for $C_{35}H_{44}ClN_3O_8$ 669.2817; Found 669.3478. FT-IR (KBr): ν$_s$(N-H)$_{carbamate}$=3439.08 cm^{-1}, ν$_s$(N-H)$_{lactam}$=3325.64 cm^{-1}, ν(O-H) =3199.48 cm^{-1}, ν$_{as}$(C-H)=2929.72 cm^{-1}, ν$_s$(C-H)=2875.63 cm^{-1}, ν(C=O)$_{carbamate}$ =1733.44 cm^{-1}, ν(C=O)$_{lactam}$=1691.34 cm^{-1}, ν(C=O)$_{quinone}$=1648.87 cm^{-1}, ν(C=C) =1608.09 cm^{-1}, δ(N-H)*$_{lactam,substituent}$=1585.91 cm^{-1}, δ(N-H)$_{carbamate}$ =1490.32 cm^{-1}, ν(C-Cl)= 1373.17 cm^{-1}, ν(C-N)$_{carbamate}$ =1323.57 cm^{-1}, ν(C- O-C)$_{carbamate}$ =1190.93 cm^{-1}, ν(C- O-C) $_{methoxy}$ =1056.32 cm^{-1} , γ(=C-H)$_{ar}$ =783.75 cm^{-1} , γ(N-H)$_{carbamate}$=730.21 cm^{-1}. ^{1}H NMR (600 MHz, CDCl$_3$) δ 9.14 (s, 1H, NH-1), 7.38 – 7.36 (m, 2H, H-32,34), 7.31 (s, 1H, H-19), 7.23 – 7.20 (m, 2H, H-31,35), 6.96 (d, $J^3_{H-3,H-4}$= 11.6 Hz, 1H, H-3), 6.58 (ddd, $J^3_{H-3,H-4}$= 11.4 Hz, $J^3_{H-4,H-5}$= 11.0 Hz, $J^4_{H-4,H-22}$= 1.1 Hz, 1H, H-4), 6.40 (t, $J^3_{NH-17,H-29}$= 5.8 Hz, 1H, NH-17), 5.90 – 5.88 (m, 1H, H-9), 5.87 – 5.84 (m, 1H, H-5), 5.19 (d, $J^4_{H-5,H-7}$ = 1.1 Hz, 1H, H-7), 4.77 (bs, 2H, NH$_2$-24), 4.72 (dd, J^2 = 14.7 Hz, $J^3_{NH-17,H-29}$= 5.9 Hz, 1H, H-29), 4.60 (dd, J^2 = 14.7 Hz, $J^3_{NH-17,H-29}$= 5.6 Hz, 1H, H-29), 4.31 (dt, $J^3_{H-6,H-7}$=9.8 Hz, $J^3_{H-4,H-6}$= 1.2 Hz, 1H, H-6), 4.07 (s, 1H, OH-11), 3.57 (d, $J^3_{H-11,H-12}$= 8.8 Hz, 1H, H-11), 3.44 (dt, $J^3_{H-11,H-12}$= 8.8 Hz, $J^3_{H-12,H-13}$= 2.8 Hz, 1H, H-12), 3.36 (s, 3H, H-27), 3.27 (s, 3H, H-23), 2.74 (dqd, $J^3_{H-9,H-10}$= 9.3 Hz, $J^3_{H-10,H-26}$= 6.9 Hz, $J^4_{H-10,H-25}$= 2.3 Hz, 1H, H-10), 2.67 – 2.63 (m, 1H, H-12), 2.38 (dd, J^2 = 14.1 Hz, $J^3_{H-14,H-15}$= 10.2 Hz, 1H, H-15), 2.03 (d, $J^4_{H-4,H-22}$= 1.4 Hz, 3H, H-22), 1.80 (d, $J^4_{H-10,H-25}$= 1.3 Hz, 3H, H-25), 1.76 (d, $J^3_{H-12,H-13}$= 2.8 Hz, 2H, H-13), 1.65 (bs, 1H, H-14), 1.02 – 0.98 (m, 6H, H-26,28). dqd - Doublet of Quartet of Doublets; bs - broad singlet
^{13}C{1H} NMR (151 MHz, CDCl$_3$) δ 183.9 (C-18), 181.4 (C-21), 168.5 (C-1), 156.1 (C-24), 144.7 (C-17), 141.3 (C-20), 136.1 (C-5), 135.3 (C-30), 135.1 (C-2), 134.5 (C-33), 133.8 (C-9), 133.0 (C-8), 129.5* (C-31,35), 129.2* (C-32,34), 127.1 (C-3), 126.7 (C-4), 109.4 (C-19), 109.1 (C-16), 81.8 (C-7), 81.5 (C-12), 81.3 (C-6), 72.8 (C-11), 57.3 (C-23), 56.9 (C-27), 49.5 (C-29), 35.2 (C-13), 34.6 (C-15), 32.5 (C-10), 28.7 (C-14), 23.1 (C-28), 12.9 (C-22), 12.8 (C-25), 12.5 (C-26).

Compound **4.** (4*E*,6*Z*,8*S*,9*S*,10*E*,12*S*,13*R*,14*S*,16*R*)-19-((4-bromobenzyl)amino)-13-hydroxy-8,14-dimethoxy-4,10,12,16-tetramethyl-3,20,22-trioxo-2-azabicyclo[16.3.1]docosa-1(21),4,6,10,18-pentaen-9-yl carbamate (168 mg, yield 66 %), mp 228-231 ºC, HPLC (OD-H, water/acetonitrile = 35/65, flow rate = 0.5 mL/min, l = 260 nm) tR = 12.488 min (major). Anal. Calcd for $C_{35}H_{44}BrN_3O_8$: C, 58.82; H, 6.21; Br, 11.18; N, 5.88. Found C, 58.81; H, 6.22; Br, 11.19; N, 5.87. HRMS (MALDI-TOF) m/z: [M + H]$^+$ Calcd for $C_{35}H_{44}BrN_3O_8$ 713.4129; Found 713.2312. FT-IR (KBr): ν$_{as}$(N-H)$_{carbamate}$=3490.76 cm^{-1}, ν$_s$(N-H)$_{carbamate}$=3432.86 cm^{-1}, ν$_s$(N-H)$_{lactam}$=3322.39 cm^{-1}, ν(O-H) =3198.39 cm^{-1}, ν$_{as}$(C-H)=2930.23 cm^{-1}, ν$_s$(C-H)=2873.61 cm^{-1}, ν(C=O)$_{carbamate}$ =1733.50 cm^{-1}, ν(C=O)$_{lactam}$=1692.90 cm^{-1}, ν(C=O)$_{quinone}$=1649.30 cm^{-1}, ν(C=C) =1586.02 cm^{-1} , δ(N-H) =1488.14 cm^{-1}, ν(C-Br)= 1373.08 cm^{-1}, ν(C-N)$_{carbamate}$ =1326.74 cm^{-1}, ν(C-O-C)$_{carbamate}$ =1190.05 cm^{-1}, ν(C-O-C)$_{substituent}$ =1099.49 cm^{-1}, ν(C-O-C)$_{methoxy}$ =1070.97 cm^{-1} , γ(=C-H)$_{ar}$ =788.58 cm^{-1} , γ(N-H)$_{carbamate}$=729.84 cm^{-1}. ^{1}H NMR (600 MHz, CDCl$_3$) δ 9.14 (s, 1H, NH-1), 7.54 – 7.51 (m, 2H, H-32,34), 7.31 (s, 1H, H-19), 7.17 – 7.14 (m, 2H, H-31,35), 6.95 (d, $J^3_{H-3,H-4}$= 11.6 Hz, 1H, H-3), 6.58 (td, $J^3_{H-3,H-4}$= 11.4 Hz, $J^4_{H-4,H-22}$= 1.1 Hz, 1H, H-4), 6.41 (t, $J^3_{NH-17,H-29}$= 5.5 Hz, 1H, NH-17), 5.90 – 5.84 (m, 2H, H-5,9), 5.19 (s, 1H, H-7), 4.82 (bs, 2H, NH$_2$-24), 4.73 – 4.68 (m, 1H, H-29), 4.59 (dd, J^2 = 14.8 Hz, $J^3_{NH-17,H-29}$= 5.7 Hz, 1H, H-29), 4.31 (dt, $J^3_{H-6,H-7}$=9.7 Hz, $J^3_{H-4,H-6}$= 1.2 Hz, 1H, H-6), 4.07 (s, 1H, OH-11), 3.57 (ddd, $J^3_{H-11,H-12}$=9.0 Hz, $J^3_{H-10,H-11}$=6.6 Hz, $J^4_{H-11,H-13}$=2.0 Hz, 1H, H-11), 3.43 (dt, $J^3_{H-11,H-12}$= 9.0

Hz, $J^3_{H-12,H-13}$= 2.8 Hz, 1H, H-12), 3.36 (s, 3H, H-27), 3.27 (s, 3H, H-23), 2.74 (tt, $J^3_{H-9,H-10}$= 9.2 Hz, $J^3_{H-10,H-26}$= 6.8 Hz, Hz, 1H, H-10), 2.67 – 2.62 (m, 1H, H-15), 2.37 (dd, J^2 = 14.1 Hz, $J^3_{H-14,H-15}$= 10.1 Hz, 1H, H-15), 2.02 (d, $J^4_{H-4,H-22}$= 1.4 Hz, 3H, H-22), 1.80 (d, $J^4_{H-10,H-25}$= 1.3 Hz, 3H, H-25), 1.75 (d, $J^3_{H-12,H-13}$= 2.8 Hz, 2H, H-13), 1.74 (bs, 1H, H-14), 1.02 – 0.98 (m, 6H, H-26,28). bs - broad singlet
^{13}C{1H} NMR (151 MHz, $CDCl_3$) δ 183.9 (C-18), 181.4 (C-21), 168.5 (C-1), 156.2 (C-24), 144.7 (C-17), 141.3 (C-20), 136.1 (C-5), 135.9 (C-30), 135.1 (C-2), 133.8 (C-9), 133.0 (C-8), 132.5* (C-32,34), 129.5* (C-31,35), 127.1 (C-3), 126.7 (C-4), 122.5 (C-33), 109.5 (C-19), 109.0 (C-16), 81.8 (C-7), 81.5 (C-12), 81.3 (C-6), 72.8 (C-11), 57.3 (C-23), 56.9 (C-27), 49.5 (C-29), 35.1 (C-13), 34.6 (C-15), 32.5 (C-10), 28.7 (C-14), 23.1 (C-28), 12.9 (C-22), 12.7 (C-25), 12.5 (C-26).

Compound ***5.*** (4*E*,6*Z*,8*S*,9*S*,10*E*,12*S*,13*R*,14*S*,16*R*)-13-hydroxy-19-((4-iodobenzyl)amino)-8,14-dimethoxy-4,10,12,16-tetramethyl-3,20,22-trioxo-2-azabicyclo[16.3.1]docosa-1(21),4,6,10,18-pentaen-9-yl carbamate, (160 mg, 59%). HPLC (OD-H, water/acetonitrile = 35/65, flow rate = 0.5 mL/min, l = 260 nm) tR =13.911 min (major). Anal. Calcd for $C_{35}H_{44}IN_3O_8$: C, 55.19 H, 5.82; I, 16.66; N, 5.52. Found: C, 55.20 H, 5.84; I, 16.65; N, 5.51. HRMS (MALDI-TOF) m/z: $[M + H]^+$ Calcd for $C_{35}H_{44}IN_3O_8$ 761.2173; Found 761.2176. FT-IR (KBr): $ν_{as}$(N-H)$_{carbamate}$=3489.48 cm^{-1}, $ν_s$(N-H)$_{carbamate}$=3433.25 cm^{-1}, $ν_s$(N-H)$_{lactam}$=3323.38 cm^{-1}, ν(O-H) =3191.22 cm^{-1}, $ν_s$(C-H) =2929.43 cm^{-1}, ν(C=O)$_{carbamate}$ =1733.03 cm^{-1} , ν(C=O)$_{lactam}$=1695.73 cm^{-1}, ν(C=O)$_{quinone}$=1646.62 cm^{-1}, ν(C=C) =1615.53 cm^{-1} , δ(N-H)*$_{lactam,substituent}$=1590.95 cm^{-1}, δ(N-H)$_{carbamate}$ =1485.53 cm^{-1}, ν(C-N)$_{carbamate}$ =1326.30 cm^{-1}, ν(C- O-C)$_{carbamate}$ =1190.30 cm^{-1}, ν(C- O-C)$_{substituent}$ =1100.30 cm^{-1}, ν(C- O-C) $_{methoxy}$ =1030.23 cm^{-1} , γ(=C-H)$_{carbamate}$ =786.67 cm^{-1} , γ(N-H)$_{carbamate}$=707.18 cm^{-1}. ^{1}H NMR (500 MHz, $CDCl_3$, 25ºC) δ 9.13 (s, 1H, NH-1), 7.74 – 7.70 (m, 2H, H-32,34), 7.30 (s, 1H, H-19), 7.04 – 7.00 (m, 2H, H-31,35), 6.95 (d, $J^3_{H3,H4}$ = 11.7 Hz, 1H, H-3), 6.62 – 6.54 (m, 1H, H-4), 6.41 (t, $J^3_{H29,NH17}$ = 5.8 Hz, 1H, NH-17), 5.91 – 5.85 (m, 2H, H-5,9), 5.19 (s, 1H, H-7), 4.84 (s, 2H, NH_2-24), 4.69 (dd, J^2= 14.8 Hz, $J^3_{H29,NH17}$ = 5.9 Hz, 1H, H-29), 4.58 (dd, J^2= 14.8 Hz, $J^3_{H29,NH17}$ = 5.7 Hz, 1H, H-29), 4.33 – 4.29 (m, 1H, H-6), 4.05 (s, 1H, OH-11), 3.59 – 3.54 (m, 1H, H-11), 3.45 – 3.41 (m, 1H, H-12), 3.36 (s, 3H, H-27), 3.27 (s, 3H, H-23), 2.78 – 2.70 (m, 1H, H-10), 2.67 – 2.61 (m, 1H, H-15), 2.36 (dd, J^2= 14.1 Hz, $J^3_{H15,H14}$ = 10.1 Hz, 1H, H-15), 2.02 (d, $J^4_{H3,H22}$ = 1.3 Hz, 3H, H-22), 1.79 (d, $J^4_{H9,H25}$ = 1.4 Hz, 3H, H-25), 1.77 – 1.73 (m, 2H, H-13), 1.65 (s, 1H, H-14), 1.00 (d, $J^3_{H10,H26}$= 7.4 Hz, 3H, H-26), 0.99 (d, $J^3_{H14,H28}$= 6.9 Hz, 3H, H-28). ^{13}C{1H} NMR (126 MHz, $CDCl_3$, 25ºC) δ 183.9 (C-18), 181.4 (C-21), 168.5 (C-1), 156.2 (C-24), 144.7 (C-17), 141.3 (C-20), 138.4* (C-32,34), 136.5 (C-30), 136.1 (C-5), 135.1 (C-2), 133.8 (C-9), 133.0 (C-8), 129.6* (C-31,35), 127.1 (C-3), 126.7 (C-4), 109.5 (C-16), 109.0 (C-19), 94.1 (C-33), 81.8 (C-7), 81.5 (C-6), 81.3 (C-12), 72.8 (C-11), 57.3 (C-23), 56.9 (C-27), 49.6 (C-29), 35.1 (C-13), 34.6 (C-15), 32.5 (C-10), 28.7 (C-14), 23.1 (C-28), 12.9 (C-25), 12.7 (C-22), 12.5 (C-26). (*-overlapped)

Compound ***6.*** (4*E*,6*Z*,8*S*,9*S*,10*E*,12*S*,13*R*,14*S*,16*R*)-13-hydroxy-8,14-dimethoxy-4,10,12,16-tetramethyl-3,20,22-trioxo-19-((4-(trifluoromethyl)benzyl)amino)-2-azabicyclo[16.3.1]docosa-1(21),4,6,10,18-pentaen-9-yl
carbamate (138 mg, yield 55 %), mp 240-242 ºC, HPLC (OD-H, water/acetonitrile = 35/65, flow rate = 0.5 mL/min, l = 260 nm) tR = 11.415 min (major). Anal. Calcd for $C_{36}H_{44}F_3N_3O_8$: C, 61.44; H, 6.30; F, 8.10; N, 5.97. Found: C, 61.42; H, 6.31; F, 8.11; N, 5.95. HRMS (MALDI-TOF) m/z: $[M + H]^+$ Calcd for $C_{36}H_{44}F_3N_3O_8$ 703.3081; Found 703.1479. FT-IR (KBr): $ν_{as}$(N-H)$_{carbamate}$=3494.61 cm^{-1}, $ν_s$(N-H)$_{carbamate}$=3433.17 cm^{-1}, $ν_s$(N-H)$_{lactam}$=3334.37 cm^{-1}, ν(O-H) =3197.32 cm^{-1}, $ν_{as}$(C-H) =2930.96 cm^{-1}, $ν_s$(C-H) =2885.76 cm^{-1}, ν(C=O)$_{carbamate}$ =1737.55 cm^{-1},ν(C=O)$_{lactam}$=1696.51 cm^{-1}, ν(C=O)$_{quinone}$=1647.55 cm^{-1}, ν(C=C) =1619.37 cm^{-1} , δ(N-H)*$_{lactam,substituent}$=1593.01 cm^{-1}, δ(N-H)$_{carbamate}$=1493.50 cm^{-1}, ν(C-N)$_{carbamate}$ =1326.45 cm^{-1}, ν(C-O-C)$_{carbamate}$ =1190.36 cm^{-1}, ν(C-O-C)$_{substituent}$ =1129.14 cm^{-1}, ν(C-O-C) $_{methoxy}$ =1067.06 cm^{-1} , γ(=C-H)$_{ar}$ =786.19 cm^{-1} , γ(N-H)$_{carbamate}$=706.58 cm^{-1}. ^{1}H NMR (600 MHz, $CDCl_3$) δ 9.13 (s, 1H, NH-1), 7.66 (d, $J^3_{H-31,35,H-32,34}$= 8.0 Hz, 2H, H-32,34), 7.40 (d, $J^3_{H-31,35,H-32,34}$= 8.0 Hz, 2H, H-31,35), 7.32 (s, 1H, H-19), 6.96 (d, $J^3_{H-3,H-4}$= 11.6 Hz, 1H, H-3), 6.61 – 6.55 (m, 1H, H-4), 6.48 (t, $J^3_{NH-17,H-29}$= 6.0 Hz, 1H, NH-17), 5.90 – 5.84 (m, 2H, H-5,9), 5.19 (s, 1H, H-7), 5.01 (bs, 2H, NH_2-24), 4.81 (dd, J^2 = 15.1 Hz, $J^3_{NH-17,H-29}$ = 6.1 Hz, 1H, H-29), 4.72 (dd, J^2 = 15.1 Hz, $J^3_{NH-17,H-29}$ = 5.9 Hz, 1H, H-29), 4.34 – 4.28 (m, 1H, H-6), 3.99 (bs, 1H, OH-11), 3.59 – 3.54 (m, 1H, H-11), 3.45 – 3.40 (m, 1H, H-12), 3.35 (s, 3H, H-27), 3.27 (s, 3H, H-23), 2.74 (ddt, $J^3_{H-9,H-10}$= 9.5 Hz, $J^3_{H-10,H-26}$= 7.0 Hz, $J^3_{H-10,H-11}$= 4.2 Hz, 1H, H-10), 2.63 (d, J^2= 14.2 Hz, 1H, H-15), 2.33 (dd, J^2= 14.2 Hz, $J^3_{H-14,H-15}$= 10.1 Hz, 1H, H-15), 2.02 (d, $J^4_{H-4,H-22}$ = 1.3 Hz, 3H, H-22), 1.79 (d, $J^3_{H-9,H-25}$ = 1.4 Hz, 3H, H-25), 1.75 (bs, 1H, H-14), 1.67 (bs, 2H, H-13), 1.01 (d, $J^3_{H-10,H-26}$ = 6.9 Hz, 3H, H-26), 0.99 (d, $J^3_{H-14,H-28}$ = 6.9 Hz, 3H, H-28). bs - broad singlet
^{13}C{1H} NMR (151 MHz, $CDCl_3$) δ 183.9 (C-18), 181.5 (C-21), 168.5 (C-1), 156.2 (C-24), 144.7 (C-17), 141.2 (C-20), 140.9 (C-30),136.1 (C-5), 135.0 (C-2), 133.7 (C-9), 133.0 (C-8), 129.9 (C-33),128.0* (C-31,32,34,35), 127.2 (C-3), 126.4 (C-36),126.3 (C-4), 109.7 (C-19), 109.1 (C-16), 81.8 (C-7), 81.5 (C-12), 81.3 (C-6), 72.8 (C-11), 57.3 (C-23), 56.9 (C-27), 49.4 (C-29), 35.1 (C-13), 34.5 (C-15), 32.5 (C-10), 28.7 (C-14), 23.1 (C-28), 13.0 (C-22), 12.7 (C-25), 12.6 (C-26).

Compound ***7.*** (4*E*,6*Z*,8*S*,9*S*,10*E*,12*S*,13*R*,14*S*,16*R*)-13-hydroxy-8,14-dimethoxy-4,10,12,16-tetramethyl-3,20,22-trioxo-19-((4-(trifluoromethoxy)benzyl)amino)-2-azabicyclo[16.3.1]docosa-1(21),4,6,10,18-pentaen-9-yl

carbamate (200 mg, yield 78 %), mp 234-236 ºC, HPLC (OD-H, water/acetonitrile = 35/65, flow rate = 0.5 mL/min, l = 260 nm) tR = 13.007 min (major). Anal. Calcd for: C, 60.08; H, 6.16; F, 7.92; N, 5.84. Found: C, 60.10; H, 6.17; F, 7.91; N, 5.85. HRMS (MALDI-TOF) m/z: $[M + H]^+$ Calcd for $C_{36}H_{44}F_3N_3O_9$ 719.3030; Found 719.2023. FT-IR (KBr): $\nu_{as}(N\text{-}H)_{carbamate}$=3488.66 cm^{-1}, $\nu_s(N\text{-}H)_{carbamate}$=3430.11 cm^{-1}, $\nu_s(N\text{-}H)_{lactam}$=3329.74 cm^{-1}, ν(O-H) =3202.29 cm^{-1}, $\nu_{as}(C\text{-}H)$ =2932.78 cm^{-1}, $\nu_s(C\text{-}H)$ =2881.71 cm^{-1}, $\nu(C{=}O)_{carbamate}$ =1737.48 cm^{-1}, $\nu(C{=}O)_{lactam}$=1700.82 cm^{-1}, $\nu(C{=}O)_{quinone}$=1649.78 cm^{-1}, ν(C=C) =1612.32 cm^{-1} , $\delta(N\text{-}H)^*_{lactam,substituent}$=1592.63 cm^{-1}, $\delta(N\text{-}H)_{carbamate}$=1505.32 cm^{-1}, $\nu(C\text{-}N)_{carbamate}$ =1328.16 cm^{-1}, $\nu(C\text{-}O\text{-}C)_{carbamate}$ =1263.06 cm^{-1}, $\nu(C\text{-}O\text{-}C)_{substituent}$ =1100.16 cm^{-1}, $\nu(C\text{-}O\text{-}C)_{methoxy}$ =1062.84 cm^{-1} , $\gamma(=C\text{-}H)_{ar}$ =787.72 cm^{-1} , $\gamma(N\text{-}H)_{carbamate}$=705.22 cm^{-1}. ^1H NMR (600 MHz, $CDCl_3$) δ 9.14 (s, 1H, NH-1), 7.32 (s, 1H, H-19), 7.31 – 7.30 (m, 2H, H-31,35), 7.25 – 7.22 (m, 2H, H-32,34), 6.96 (d, $J^3_{H-3,H-4}$= 11.6 Hz, 1H, H-3), 6.61 – 6.55 (m, 1H, H-4), 6.42 (t, $J^3_{NH-17,H-29}$= 5.6 Hz, 1H, NH-17), 5.90 – 5.84 (m, 2H, H-5,9), 5.19 (s, 1H, H-7), 4.85 (bs, 2H, NH_2-24), 4.75 (dd, J^2 = 14.7 Hz, $J^3_{NH-17,H-29}$= 5.9 Hz, 1H, H-29), 4.64 (dd, J^2 = 14.7 Hz, $J^3_{NH-17,H-29}$= 5.9 Hz, 1H, H-29), 4.33 – 4.29 (m, 1H, H-6), 4.05 (s, 1H, OH-11), 3.59 – 3.54 (m, 1H, H-11), 3.44 (dt, $J^3_{H-11,H-12}$= 8.9 Hz, $J^3_{H-12,H-13}$= 2.7 Hz, 1H, H-12), 3.36 (s, 3H, H-27), 3.27 (s, 3H, H-23), 2.74 (dqd, $J^3_{H-9,H-10}$= 9.1 Hz, $J^3_{H-10,H-26}$= 6.8 Hz, $J^4_{H-10,H-25}$= 3.6 Hz, 1H, H-10), 2.66 (d, J^2 = 14.1 Hz, 1H, H-15), 2.38 (dd, J^2 = 14.2 Hz, $J^3_{H-14,H-15}$= 10.1 Hz, 1H, H-15), 2.03 (s, 3H, H-22), 1.80 (d, $J^4_{H-10,H-25}$= 1.3 Hz, 3H, H-25), 1.76 (d, $J^3_{H-12,H-13}$= 2.8 Hz, 2H, H-13), 1.73 (bs, 1H, H-14), 1.03 – 0.97 (m, 6H, H-26,28). dqd - Doublet of Quartet of Doublets; bs - broad singlet
$^{13}C\{1H\}$ NMR (151 MHz, $CDCl_3$) δ 183.9 (C-18), 181.4 (C-21), 168.5 (C-1), 156.2 (C-24), 149.3 (C-33), 144.7 (C-17), 141.3 (C-20), 136.1 (C-5), 135.6 (C-30), 135.1 (C-2), 133.8 (C-9), 133.0 (C-8), 129.9 (C-36), 129.3* (C-31,35), 127.2 (C-3), 126.7 (C-4), 121.8* (C-32,34), 109.5 (C-19), 109.1 (C-16), 81.8 (C-7), 81.5 (C-12), 81.3 (C-6), 72.8 (C-11), 57.3 (C-23), 56.9 (C-27), 49.4 (C-29), 35.1 (C-13), 34.6 (C-15), 32.5 (C-10), 28.7 (C-14), 23.1 (C-28), 13.0 (C-22), 12.7 (C-25), 12.6 (C-26).

Compound ***8.*** (4E,6Z,8S,9S,10E,12S,13R,14S,16R)-19-((4-cyanobenzyl)amino)-13-hydroxy-8,14-dimethoxy-4,10,12,16-tetramethyl-3,20,22-trioxo-2-azabicyclo[16.3.1]docosa-1(21),4,6,10,18-pentaen-9-yl carbamate (184 mg, yield 78 %), mp 142-144 ºC, HPLC (OD-H, water/acetonitrile = 35/65, flow rate = 0.5 mL/min, l = 260 nm) tR = 6.779 min (major). Anal. Calcd for: C, 65.44; H, 6.71; N, 8.48. Found: C, 65.43; H, 6.72; N, 8.50. HRMS (MALDI-TOF) m/z: $[M + H]^+$ Calcd for $C_{36}H_{44}N_4O_8$ 660.3159; Found 660.5632. FT-IR (KBr): $\nu_{as}(N\text{-}H)_{carbamate}$=3492.33 cm^{-1}, $\nu_s(N\text{-}H)_{lactam}$=3331.99 cm^{-1}, ν(O-H) =3195.72 cm^{-1}, $\nu_{as}(C\text{-}H)$ =2930.10 cm^{-1}, $\nu_s(C\text{-}H)$ =2875.63 cm^{-1}, $\nu_s(C\text{-}N)$ =2229.67 cm^{-1}, $\nu(C{=}O)_{carbamate}$ =1726.21 cm^{-1}, $\nu(C{=}O)_{lactam}$=1695.29 cm^{-1}, $\nu(C{=}O)_{quinone}$=1652.12 cm^{-1}, ν(C=C) =1607.99 cm^{-1} , δ(N-H)=1585.58 cm^{-1}, $\nu(C\text{-}N)_{carbamate}$ =1373.02 cm^{-1}, $\nu(C\text{-}O\text{-}C)_{carbamate}$ =1252.24 cm^{-1}, ν(C-O-C) =1099.55 cm^{-1}, $\nu(C\text{-}O\text{-}C)_{methoxy}$ =1057.22 cm^{-1}, $\gamma(=C\text{-}H)_{ar}$ =782.85 cm^{-1} , $\gamma(N\text{-}H)_{carbamate}$=700.50 cm^{-1}. ^1H NMR (600 MHz, $CDCl_3$) δ 9.11 (s, 1H, NH-1), 7.71 – 7.68 (m, 2H, H-32,34), 7.40 – 7.37 (m, 2H, H-31,35), 7.32 (s, 1H, H-19), 6.98 – 6.93 (m, 1H, H-2), 6.58 (ddd, $J^3_{H-3,H-4}$= 11.7 Hz, $J^3_{H-4,H-5}$= 11.1 Hz, $J^4_{H-4,H-22}$= 1.1 Hz, 1H, H-4), 6.48 (t, $J^3_{NH-17,H-29}$= 6.2 Hz, 1H, NH-17), 5.89 – 5.84 (m, 2H, H-5,9), 5.19 (s, 1H, H-7), 4.90 (bs, 2H, NH_2-24), 4.80 (dd, J^2 = 15.6 Hz, $J^3_{NH-17,H-29}$= 6.3 Hz, 1H, H-29), 4.74 (dd, J^2 = 15.6 Hz, $J^3_{NH-17,H-29}$= 6.1 Hz, 1H, H-29), 4.31 (dt, $J^3_{H-6,H-7}$=9.7 Hz, $J^3_{H-4,H-6}$= 1.2 Hz, 1H, H-6), 3.88 (bs, 1H, OH-11), 3.57 – 3.54 (m, 1H, H-11), 3.42 (dt, $J^3_{H-11,H-12}$= 8.9 Hz, $J^3_{H-12,H-13}$= 2.7 Hz, 1H, H-12), 3.35 (s, 3H, H-27), 3.27 (s, 3H, H-23), 2.74 (dqd, $J^3_{H-9,H-10}$= 9.4 Hz, $J^3_{H-10,H-26}$= 6.9 Hz, $J^4_{H-10,H-25}$= 2.3 Hz, 1H, H-10), 2.64 – 2.59 (m, 1H, H-15), 2.30 – 2.22 (m, 1H, H-15), 2.02 (s, 3H, H-22), 1.79 (d, $J^4_{H-10,H-25}$= 1.3 Hz, 3H, H-25), 1.75 – 1.71 (m, 1H, H-14), 1.68 – 1.59 (m, 2H, H-13), 0.99 (d, $J^3_{H-10,H-26}$ = 6.5 Hz, 3H, H-26), 0.98 (d, $J^3_{H-14,H-28}$ = 6.9 Hz, 3H, H-28).
$^{13}C\{1H\}$ NMR (151 MHz, $CDCl_3$) δ 183.9 (C-18), 181.6 (C-21), 168.5 (C-1), 156.2 (C-24), 144.6 (C-17), 142.3 (C-30), 141.1 (C-20), 136.2 (C-5), 135.0 (C-2), 133.6 (C-9), 133.1* (C-8,32,34), 128.2* (C-31,35), 127.2 (C-3), 126.6 (C-4), 118.4 (C-36), 112.5 (C-33), 110.0 (C-19), 109.1 (C-16), 81.8 (C-7), 81.4 (C-12), 81.3 (C-6), 72.8 (C-11), 57.3 (C-23), 56.9 (C-27), 49.3 (C-29), 35.1 (C-13), 34.4 (C-15), 32.5 (C-10), 28.7 (C-14), 23.1 (C-28), 13.0 (C-22), 12.7 (C-25), 12.6 (C-26). dqd - Doublet of Quartet of Doublets; bs - broad singlet

Compound ***9.*** (4*E*,6*Z*,8*S*,9*S*,10*E*,12*S*,13*R*,14*S*,16*R*)-13-hydroxy-8,14-dimethoxy-4,10,12,16-tetramethyl-19-((4-nitrobenzyl)amino)-3,20,22-trioxo-2-azabicyclo[16.3.1]docosa-1(21),4,6,10,18-pentaen-9-yl carbamate (77 mg, yield 32 %), mp 159-161 ºC, HPLC (OD-H, water/acetonitrile = 35/65, flow rate = 0.5 mL/min, l = 260 nm) tR = 7.478 min (major). Anal. Calcd for $C_{35}H_{44}N_4O_{10}$: C, 61.75; H, 6.52; N, 8.23. Found: C, 61.74; H, 6.50; N, 8.25. HRMS (MALDI-TOF) m/z: $[M + H]^+$ Calcd for $C_{35}H_{44}N_4O_{10}$ 680.3057; Found 680.4896. FT-IR (KBr): $\nu_{as}(N\text{-}H)_{carbamate}$=3480.67 cm^{-1}, $\nu_s(N\text{-}H)_{lactam}$=3335.99 cm^{-1}, ν(O-H) =3203.83 cm^{-1}, $\nu_{as}(C\text{-}H)$ =2930.39 cm^{-1}, $\nu_s(C\text{-}H)$ =2873.61 cm^{-1}, $\nu(C{=}O)_{carbamate}$ =1724.06 cm^{-1}, $\nu(C{=}O)_{lactam}$=1689.56 cm^{-1}, $\nu(C{=}O)_{quinone}$=1649.08 cm^{-1}, ν(C=C) =1611.48 cm^{-1} , δ(N-H)=1581.69 cm^{-1}, $\nu_{as}(C\text{-}NO_2)$=1487.56 cm^{-1}, $\nu(C\text{-}N)_{carbamate}$ =1372.17 cm^{-1}, $\nu_s(C\text{-}NO_2)$=1344.27 cm^{-1}, $\nu(C\text{-}O\text{-}C)_{carbamate}$ =1252.23 cm^{-1}, ν(C-O-C) =1099.67 cm^{-1}, $\nu(C\text{-}O\text{-}C)_{methoxy}$ =1055.60 cm^{-1} , $\gamma(=C\text{-}H)_{ar}$ =785.17 cm^{-1} , $\gamma(N\text{-}H)_{carbamate}$=699.83 cm^{-1}. ^1H NMR (500 MHz, $CDCl_3$) δ 9.10 (s, 1H, NH-1), 8.29 – 8.22 (m, 2H, H-32,34), 7.47 – 7.43 (m, 2H, H-31,35), 7.33 (s, 1H, H-19), 6.95 (d, $J^3_{H-3,H-4}$= 11.6 Hz, 1H, H-3), 6.58 (td, $J^3_{H-3,H-4}$= 11.4 Hz, $J^4_{H-4,H-22}$= 1.1 Hz, 1H, H-4), 6.51 (t, $J^3_{NH-17,H-29}$= 6.2 Hz, 1H, NH-17), 5.90 – 5.82 (m, 2H, H-5,9), 5.19 (s, 1H, H-7), 4.85 (dd, J^2 = 16.0 Hz, $J^3_{NH-17,H-29}$= 6.6 Hz, 1H, H-29), 4.81 (dd, J^2 = 15.9 Hz, J^3_{NH-}

$_{17,H-29}$= 6.3 Hz, 1H, H-29), 4.79 (bs, 2H, NH_2-24), 4.31 (dt, $J^3_{H-6,H-7}$=9.5 Hz, $J^3_{H-4,H-6}$= 1.2 Hz, 1H, H-6), 3.85 (bs, 1H, OH-11), 3.56 (d, $J^3_{H-11,H-12}$=8.7, 1H, H-11), 3.42 (dt, $J^3_{H-11,H-12}$= 8.9 Hz, $J^3_{H-12,H-13}$= 2.7 Hz, 1H, H-12), 3.35 (s, 3H, H-27), 3.27 (s, 3H, H-23), 2.74 (tt, $J^3_{H-9,H-10}$= 9.3 Hz, $J^3_{H-10,H-26}$= 6.8 Hz, 1H, H-10), 2.61 (d, J^2 = 15.3 Hz, 1H, H-15), 2.25 (dd, J^2 = 14.1 Hz, $J^3_{H-14,H-15}$= 10.0 Hz, 1H, H-15), 2.03 (d, $J^4_{H-4,H-22}$ = 1.3 Hz, 3H, H-22), 1.79 (d, $J^4_{H-10,H-25}$= 1.3 Hz, 3H, H-25), 1.76 – 1.70 (m, 1H, H-14), 1.69 – 1.63 (m, 2H, H-13), 1.04 – 0.98 (m, 6H, H-26,28). bs - broad singlet

^{13}C{1H} NMR (151 MHz, $CDCl_3$) δ 183.9 (C-18), 181.2 (C-21), 168.5 (C-1), 156.2 (C-24), 148.0 (C-30), 144.6 (C-30), 144.3 (C-17), 141.1 (C-20), 136.2 (C-5), 135.0 (C-2), 133.6 (C-9), 133.1 (C-8), 128.3* (C-31,35), 127.2 (C-3), 126.6 (C-4), 124.5* (C-32,34), 110.1 (C-19), 109.2 (C-16), 81.7 (C-7), 81.4 (C-12), 81.3 (C-6), 72.8 (C-11), 57.3 (C-23), 56.9 (C-27), 49.1 (C-29), 35.1 (C-13), 34.5 (C-15), 32.5 (C-10), 28.7 (C-14), 23.1 (C-28), 13.0 (C-22), 12.7 (C-25), 12.6 (C-26).

Compound ***10.*** (4*E*,6*Z*,8*S*,9*S*,10*E*,12*S*,13*R*,14*S*,16*R*)-13-hydroxy-8,14-dimethoxy-4,10,12,16-tetramethyl-19-((4-methylbenzyl)amino)-3,20,22-trioxo-2-azabicyclo[16.3.1]docosa-1(21),4,6,10,18-pentaen-9-yl carbamate (212 mg, yield 91 %), mp 237-238 ºC, HPLC (OD-H, water/acetonitrile = 35/65, flow rate = 0.5 mL/min, l = 260 nm) tR = 12.065 min (major). Anal. Calcd for $C_{36}H_{47}N_3O_8$: C, 66.54; H, 7.29; N, 6.47. Found: C, 66.53; H, 7.28; N, 6.49. HRMS (MALDI-TOF) m/z: $[M + H]^+$ Calcd for $C_{36}H_{47}N_3O_8$; Found 649.3547. FT-IR (KBr): ν_s(N-H)$_{carbamate}$=3439.88 cm^{-1}, ν_s(N-H)$_{lactam}$=3329.23 cm^{-1}, ν(O-H) =3202.94 cm^{-1}, ν_{as}(C-H)=2929.66 cm^{-1}, ν_s(C-H)=2879.68 cm^{-1}, ν(C=O)$_{carbamate}$ =1732.22 cm^{-1}, ν(C=O)$_{lactam}$=1691.26 cm^{-1}, ν(C=O)$_{quinone}$=1649.34 cm^{-1}, ν(C=C) =1613.55 cm^{-1} , δ(N-H)*$_{lactam,substituent}$=1587.22 cm^{-1}, δ(N-H)$_{carbamate}$ =1487.98 cm^{-1}, ν(C-N)$_{carbamate}$ =1322.54 cm^{-1}, ν(C-O-C)$_{carbamate}$ =1190.51 cm^{-1}, ν(C-O-C)$_{methoxy}$ =1056.16 cm^{-1} , γ(=C-H)$_{ar}$ =783.97 cm^{-1} , γ(N-H)$_{carbamate}$=729.88 cm^{-1}. ^{1}H NMR (600 MHz, $CDCl_3$) δ 9.17 (s, 1H, NH-1), 7.29 (s, 1H, H-19), 7.21 – 7.17 (d, $J^3_{H-31,35,H-32,34}$= 8.2 Hz, 2H, H-32,34), 7.15 (d, $J^3_{H-31,35,H-32,34}$= 8.2 Hz, 2H, H-31,35), 6.95 (d, $J^3_{H-3,H-4}$= 11.6 Hz, 1H, H-3), 6.58 (td, $J^3_{H-3,H-4}$= 11.6 Hz, $J^4_{H-4,H-22}$= 1.1 Hz, 1H, H-4), 6.43 (t, $J^3_{NH-17,H-29}$= 5.5 Hz, 1H, NH-17), 5.89 (d, $J^3_{H-9,H-10}$ = 9.6 Hz, 1H, H-9), 5.86 (t, $J^3_{H-5,H-6}$ = 10.5 Hz, 1H, H-5), 5.18 (s, 1H, H-7), 4.90 (bs, 2H, NH_2-24), 4.71 (dd, J^2 = 14.4 Hz, $J^3_{NH-17,H-29}$= 5.7 Hz, 1H, H-29), 4.56 (dd, J^2 = 14.4 Hz, $J^3_{NH-17,H-29}$= 5.4 Hz, 1H, H-29), 4.31 (dt, $J^3_{H-6,H-7}$=9.9 Hz, $J^3_{H-4,H-6}$= 1.1 Hz, 1H, H-6), 4.23 (s, 1H, OH-11), 3.58 (d, $J^3_{H-11,H-12}$= 9.0 Hz, 1H, H-11), 3.44 (dt, $J^3_{H-11,H-12}$= 9.0 Hz, $J^3_{H-12,H-13}$= 2.9 Hz, 1H, H-12), 3.36 (s, 3H, H-27), 3.27 (s, 3H, H-23), 2.74 (dqd, $J^3_{H-9,H-10}$= 9.2 Hz, $J^3_{H-10,H-26}$= 6.9 Hz, $J^4_{H-10,H-25}$= 2.0 Hz, 1H, H-10), 2.68 – 2.64 (m, 1H, H-15), 2.46 (dd, J^2 = 14.2 Hz, $J^3_{H-14,H-15}$= 10.5 Hz, 1H, H-15), 2.36 (s, 3H, H-36), 2.02 (d, $J^4_{H-4,H-22}$= 1.4 Hz, 3H, H-22), 1.80 (d, $J^4_{H-10,H-25}$= 1.3 Hz, 3H, H-25), 1.77 (d, $J^3_{H-12,H-13}$= 3.2 Hz, 2H, H-13), 1.76 – 1.71 (m, 1H, H-14), 1.03 (d, $J^3_{H-10,H-26}$ = 6.5 Hz, 3H, H-26), 0.99 (d, $J^3_{H-14,H-28}$ = 6.9 Hz, 3H, H-28). ^{13}C{1H} NMR (151 MHz, $CDCl_3$) δ 183.9 (C-18), 181.1 (C-21), 168.5 (C-1), 156.3 (C-24), 144.8 (C-17), 141.4 (C-20), 138.4 (C-33), 136.0 (C-5), 135.1 (C-2), 133.9 (C-30), 133.7 (C-9), 132.9 (C-8), 130.0* (C-32,34), 127.9* (C-31,35), 127.1 (C-3), 126.7 (C-4), 109.0* (C-16,19), 81.8 (C-7), 81.6 (C-12), 81.4 (C-6), 72.8 (C-11), 57.2 (C-23), 56.9 (C-27), 50.1 (C-29), 35.2 (C-13), 34.6 (C-15), 32.5 (C-10), 28.6 (C-14), 23.1 (C-28), 21.3 (C-36), 12.9 (C-22), 12.7 (C-25), 12.5 (C-26). dqd - Doublet of Quartet of Doublets; bs - broad singlet

Compound ***11.*** (4*E*,6*Z*,8*S*,9*S*,10*E*,12*S*,13*R*,14*S*,16*R*)-13-hydroxy-8,14-dimethoxy-19-((4-methoxybenzyl)amino)-4,10,12,16-tetramethyl-3,20,22-trioxo-2-azabicyclo[16.3.1]docosa-1(21),4,6,10,18-pentaen-9-yl carbamate (179 mg, yield 75 %), mp 215-217 ºC, HPLC (OD-H, water/acetonitrile = 35/65, flow rate = 0.5 mL/min, l = 260 nm) tR = 8.924 min (major). Anal. Calcd for $C_{36}H_{47}N_3O_9$: C, 64.95; H, 7.12; N, 6.31. Found: C, 64.94; H, 7.10; N, 6.32. HRMS (MALDI-TOF) m/z: $[M + H]^+$ Calcd for $C_{36}H_{47}N_3O_9$ 665.3312; Found 665.7111. FT-IR (KBr): ν_{as}(N-H)$_{carbamate}$=3483.95 cm^{-1}, ν_s(N-H)$_{carbamate}$=3429.37 cm^{-1}, ν_s(N-H)$_{lactam}$=3333.68 cm^{-1}, ν(O-H) =3194.51 cm^{-1}, ν_{as}(C-H)=2934.14 cm^{-1}, ν_s(C-H)=2861.45 cm^{-1}, ν(C=O)$_{carbamate}$ =1734.45 cm^{-1}, ν(C=O)$_{lactam}$=1701.58 cm^{-1}, ν(C=O)$_{quinone}$=1647.98 cm^{-1}, ν(C=C)=1617.59 cm^{-1}, δ(N-H) =1499.84 cm^{-1}, ν(C-N)$_{carbamate}$ =1329.29 cm^{-1}, ν(C-O-C)$_{carbamate}$ =1174.22 cm^{-1}, ν(C-O-C)$_{methoxy}$=1055.38 cm^{-1} , γ(=C-H)$_{ar}$ =789.80 cm^{-1} , γ(N-H)$_{carbamate}$=712.90 cm^{-1}. ^{1}H NMR (600 MHz, $CDCl_3$) δ 9.17 (s, 1H, NH-1), 7.29 (s, 1H, H-19), 7.21 – 7.18 (m, 2H, H-31,35), 6.96 (d, $J^3_{H-3,H-4}$= 11.7 Hz, 1H, H-3), 6.92 – 6.89 (m, 2H, H-32,34), 6.58 (td, $J^3_{H-3,H-4}$= 11.3 Hz, $J^4_{H-4,H-22}$= 1.1 Hz, 1H, H-4), 6.37 (t, $J^3_{NH-17,H-29}$= 5.5 Hz, 1H, NH-17), 5.90 (d, $J^3_{H-9,H-10}$ = 9.6 Hz, 1H, H-9), 5.86 (t, $J^3_{H-5,H-6}$ = 10.5 Hz, 1H, H-5), 5.19 (s, 1H, H-7), 4.83 (bs, 2H, NH_2-24), 4.72 – 4.67 (m, 1H, H-29), 4.53 (dd, J^2 = 14.2 Hz, $J^3_{NH-17,H-29}$= 5.3 Hz, 1H, H-29), 4.31 (dt, $J^3_{H-6,H-7}$=9.9 Hz, $J^3_{H-4,H-6}$= 1.2 Hz, 1H, H-6), 4.24 (s, 1H, OH-11), 3.82 (s, 3H, H-36), 3.58 (ddd, $J^3_{H-11,H-12}$=9.0 Hz, $J^3_{H-10,H-11}$=6.5 Hz, $J^4_{H-11,H-13}$=2.2 Hz, 1H, H-11), 3.45 (dt, $J^3_{H-11,H-12}$= 9.0 Hz, $J^3_{H-12,H-13}$= 2.9 Hz, 1H, H-12), 3.37 (s, 3H, H-27), 3.27 (s, 3H, H-23), 2.78 – 2.72 (m, 1H, H-10), 2.70 – 2.65 (m, 1H, H-15), 2.47 (dd, J^2 = 14.2 Hz, $J^3_{H-14,H-15}$= 10.6 Hz, 1H, H-15), 2.02 (d, $J^4_{H-4,H-22}$= 1.3 Hz, 3H, H-22), 1.80 (d, $J^4_{H-10,H-25}$= 1.3 Hz, 3H, H-25), 1.78 (d, $J^3_{H-12,H-13}$= 3.2 Hz, 2H, H-13), 1.76 – 1.73 (m, 1H, H-14), 1.03 (d, $J^3_{H-10,H-26}$ = 6.5 Hz, 3H, H-26), 1.00 (d, $J^3_{H-14,H-28}$ = 6.9 Hz, 3H, H-28). bs - broad singlet

^{13}C{1H} NMR (151 MHz, $CDCl_3$) δ 183.9 (C-18), 181.1 (C-21), 168.5 (C-1), 159.8 (C-33), 156.2 (C-24), 144.8 (C-17), 141.4 (C-20), 136.0 (C-5), 135.1 (C-2), 133.9 (C-9), 132.9 (C-8), 129.4* (C-31,35), 128.7 (C-30), 127.1 (C-3), 126.7 (C-4), 114.7* (C-32,34), 109.0 (C-19), 108.9 (C-19), 81.8 (C-7), 81.6 (C-12), 81.4 (C-6), 72.8 (C-

11), 57.3 (C-23), 56.9 (C-27), 55.5 (C-36), 49.9 (C-29), 35.2 (C-13), 34.7 (C-15), 32.5 (C-10), 28.7 (C-14), 23.1 (C-28), 12.9 (C-22), 12.8 (C-25), 12.5 (C-26).

Compound ***12.*** (4*E*,6*Z*,8*S*,9*S*,10*E*,12*S*,13*R*,14*S*,16*R*)-19-((4-(dimethylamino)benzyl)amino)-13-hydroxy-8,14-dimethoxy-4,10,12,16-tetramethyl-3,20,22-trioxo-2-azabicyclo[16.3.1]docosa-1(21),4,6,10,18-pentaen-9-yl carbamate (215 mg, yield 89 %), mp 207-209 ºC, HPLC (OD-H, water/acetonitrile = 35/65, flow rate = 0.5 mL/min, l = 260 nm) tR = 11.915 min (major). Anal. Calcd for $C_{37}H_{50}N_4O_8$: C, 65.47; H, 7.42; N, 8.25. Found: C, 65.48; H, 7.41; N, 8.24. HRMS (MALDI-TOF) m/z: $[M + H]^+$ Calcd for $C_{37}H_{50}N_4O_8$ 678.3629; Found 678.3791. FT-IR (KBr): ν_{as}(N-H)$_{carbamate}$=3476.98 cm^{-1}, ν_s(N-H)$_{carbamate}$=3438.03 cm^{-1}, ν_s(N-H)$_{lactam}$=3333.73 cm^{-1}, ν(O-H) =3206.48 cm^{-1}, ν_{as}(C-H) =2933.02 cm^{-1}, ν_s(C-H) =2824.65 cm^{-1}, ν(C=O)$_{carbamate}$ =1738.23 cm^{-1}, ν(C=O)$_{lactam}$=1696.10 cm^{-1}, ν(C=O)$_{quinone}$=1651.30 cm^{-1}, ν(C=C) =1615.34 cm^{-1} , δ(N-H)*$_{lactam,substituent}$=1558.71 cm^{-1}, δ(N-H)$_{carbamate}$ =1487.16 cm^{-1}, ν(C-N)$_{carbamate}$ =1324.21 cm^{-1}, ν(C-O-C)$_{carbamate}$ =1193.03 cm^{-1}, ν(C-O-C) =1107.35 cm^{-1}, ν(C-O-C)$_{methoxy}$ =1056.87 cm^{-1} , γ(=C-H)$_{ar}$=781.36 cm^{-1} , γ(N-H)$_{carbamate}$=695.36 cm^{-1}. ^{1}H NMR (600 MHz, $CDCl_3$) δ 9.19 (s, 1H, NH-1), 7.28 (s, 1H, H-19), 7.15 – 7.12 (m, 2H, H-31,35), 6.96 (d, $J^3_{H-3,H-4}$= 11.7 Hz, 1H, H-3), 6.73 – 6.69 (m, 2H, H-32,34), 6.58 (ddd, $J^3_{H-3,H-4}$= 11.8 Hz, $J^3_{H-4,H-5}$= 11.1 Hz, $J^4_{H-4,H-22}$= 1.0 Hz, 1H, H-4), 6.38 – 6.34 (m, 1H, NH-17), 5.92 (d, $J^3_{H-9,H-10}$ = 9.5 Hz, 1H, H-9), 5.88 – 5.84 (m, 1H, H-5), 5.19 (s, 1H, H-7), 4.84 (bs, 2H, NH_2-24), 4.66 (dd, J^2 = 14.1 Hz, $J^3_{NH-17,H-29}$= 5.4 Hz, 1H, H-29), 4.48 (dd, J^2 = 14.1 Hz, $J^3_{NH-17,H-29}$= 5.2 Hz, 1H, H-29), 4.35 (bs, 1H, 11-OH), 4.31 (dt, $J^3_{H-6,H-7}$=9.9 Hz, $J^3_{H-4,H-6}$= 1.2 Hz, 1H, H-6), 3.61 – 3.55 (m, 1H, H-11), 3.46 (dt, $J^3_{H-11,H-12}$= 9.0 Hz, $J^3_{H-12,H-13}$= 2.9 Hz, 1H, H-12), 3.37 (s, 3H, H-27), 3.27 (s, 3H, H-23), 2.96 (s, 6H, H-36,37), 2.75 (dqd, $J^3_{H-9,H-10}$= 9.1 Hz, $J^3_{H-10,H-26}$= 6.8Hz, $J^4_{H-10,H-25}$= 2.1 Hz, 1H, H-10), 2.71 – 2.67 (m, 1H, H-15), 2.52 (dd, J^2 = 14.2 Hz, $J^3_{H-14,H-15}$= 10.6 Hz, 1H, H-15), 2.03 (d, $J^4_{H-4,H-22}$= 0.8 Hz, 3H, H-22), 1.80 (d, $J^4_{H-10,H-25}$= 1.4 Hz, 3H, H-25), 1.79 (bs, 1H, H-14), 1.66 (s, 2H, H-13), 1.05 (d, $J^3_{H-10,H-26}$ = 6.6 Hz, 3H, H-26), 1.00 (d, $J^3_{H-14,H-28}$ = 6.9 Hz, 3H, H-28). dqd - Doublet of Quartet of Doublets; bs - broad singlet
^{13}C{1H} NMR (151 MHz, $CDCl_3$) δ 184.0 (C-18), 180.9 (C-21), 168.5 (C-1), 156.2 (C-24), 150.7 (C-33) 144.9 (C-17), 141.5 (C-20),135.9 (C-5), 135.2 (C-2), 134.0 (C-9), 132.9 (C-8), 129.2* (C-31,35), 127.0 (C-3), 126.7 (C-4), 124.0 (C-30), 112.9* (C-32,34), 108.9 (C-19), 108.7 (C-16), 81.8 (C-7), 81.7 (C-12), 81.4 (C-6), 72.8 (C-11), 57.3 (C-23), 56.9 (C-27), 50.2 (C-29), 40.6* (C-36,37), 35.2 (C-13), 34.8 (C-15), 32.5 (C-10), 28.6 (C-14), 23.1 (C-28), 12.9 (C-22), 12.7 (C-25), 12.5 (C-26).

Spectral characteristic of a **GDM** analogs **1a-16a**:
Compound ***1a.*** (4*E*,6*Z*,8*S*,9*S*,10*E*,12*S*,13*R*,14*S*,16*R*)-1^5,13-dihydroxy-8,14-dimethoxy-4,10,12,16-tetramethyl-3-oxo-1^2-phenyl-2-aza-1(6,4) benzo[*d*]oxazolacycloheptadecaphane-4,6,10-trien-9-yl carbamate (72mg, yield 72 %), mp 235-237 ºC, HPLC (OD-H, water/acetonitrile = 35/65, flow rate = 1.0 mL/min, l = 260 nm) tR = 3.912 min (major). Anal. Calcd for $C_{35}H_{43}N_3O_8$: C, 66.33 H,6.84; N, 6.63. Found: C, 66.35; H,6.83; N, 6.66. HRMS (MALDI-TOF) m/z: $[M + H]^+$ Calcd for $C_{35}H_{43}N_3O_8$ 633.3050; Found 633.1470. FT-IR (KBr): ν_{as}(N-H)$_{carbamate}$ =3440.19 cm^{-1}, ν_s(N-H)$_{carbamate}$=3392.27 cm^{-1}, ν_s(N-H)$_{lactam}$=3337.99 cm^{-1}, ν(O-H)$_{phenol}$ =3290.94 cm^{-1}, ν(O-H) =3185.63 cm^{-1}, ν_{as}(C-H) =2927.33 cm^{-1}, ν_s(C-H) =2875.63 cm^{-1}, ν(C=O)$_{carbamate}$ =1733.39 cm^{-1}, ν(C=O)$_{lactam}$=1699.86 cm^{-1}, ν(C=N) =1656.84 cm^{-1}, ν(C=C) =1615.53 cm^{-1} , δ(N-H) =1531.81 cm^{-1}, δ(N-H)$_{carbamate}$ =1480.18 cm^{-1}, ν(C-N)$_{carbamate}$ =1379.11 cm^{-1}, ν(C-O-C)$_{carbamate}$ =1174.70 cm^{-1}, ν(C-O-C) =1108.58 cm^{-1}, ν(C-O-C)$_{methoxy}$ =1061.44 cm^{-1} , γ(=C-H)$_{ar}$=779.78 cm^{-1} , γ(N-H)$_{carbamate}$=695.28 cm^{-1}. ^{1}H NMR (500 MHz, DMSO-d_6+ACN-d_3) δ 9.54 (s, 1H, NH-1), 8.60 (s, 1H, OH-21), 8.19 – 8.14 (m, 2H, H-31,35), 7.96 (s, 1H, H-33), 7.67 – 7.60 (m, 2H, H-32,34), 7.34 (s, 1H, H-19), 6.51 – 6.44 (m, 1H, NH_2-24), 6.37 – 6.28 (m, 2H, NH_2-24, H-4), 6.03 (d, $J^3_{H-3,H-4}$= 11.6 Hz, 1H, H-3), 5.01 – 4.88 (m, 2H, H-5,9), 4.69 (d, $J^3_{H-6,H-7}$= 9.5 Hz, 1H, H-7), 4.33 – 4.28 (m, 1H, OH-11), 3.75 (t, $J^3_{H-6,H-7}$= 10.1 Hz, 1H, H-6), 3.49 – 3.43 (m, 1H, H-11), 3.28 (d, J^2= 12.1 Hz, 1H, H-15), 3.21 (s, 3H, H-27), 3.12 (d, J^2= 11.6 Hz, 1H, H-15), 2.97 (s, 3H, H-23), 2.71 – 2.65 (m, 1H, H-12), 2.24 (bs, 1H, H-14), 1.98 (s, 3H, H-22), 1.88 – 1.78 (m, 1H, H-10), 1.76 – 1.71 (m, 1H, H-13), 0.74 (d, $J^3_{H-14,H-28}$ = 6.6 Hz, 3H, H-28), 0.64 (d, $J^3_{H-10,H-26}$ = 6.3 Hz, 3H, H-26), 0.52 (t, J^2 = 12.7 Hz, 1H, H-13), 0.32 (s, 3H, H-25). bs - broad singlet
^{13}C{1H} NMR (126 MHz, DMSO-d_6+ ACN-d_3) δ 174.2 (C-1), 162.3 (C-29), 156.5 (C-24), 148.1 (C-21), 144.4 (C-18), 140.8 (C-17), 135.9 (C-2), 133.9 (C-9), 132.0 (C-30), 130.0 (C-4), 129.8* (C-32,34), 129.2 (C-8), 127.6* (C-20,31,35), 127.5 (C-33), 127.4 (C-5), 123.5 (C-3), 119.2 (C-16), 105.0 (C-19), 80.4 (C-7), 79.9 (C-12), 74.5 (C-6), 71.3 (C-11), 55.9 (C-27), 55.3 (C-23), 35.0 (C-10), 31.6 (C-15), 31.2 (C-13), 30.5 (C-14), 18.8 (C-26), 16.7 (C-28), 14.1 (C-22), 9.8 (C-25). (*-overlapped)

Compound ***2a.*** (4*E*,6*Z*,8*S*,9*S*,10*E*,12*S*,13*R*,14*S*,16*R*)-1^2-(4-fluorophenyl)-1^5,13-dihydroxy-8,14-dimethoxy-4,10,12,16-tetramethyl-3-oxo-2-aza-1(6,4)-benzo[*d*]oxazolacycloheptadecaphane-4,6,10-trien-9-yl carbamate (74 mg, yield 74 %), mp 249-254 ºC, HPLC (OD-H, water/acetonitrile = 35/65, flow rate = 1.0 mL/min, l = 260 nm) tR = 4.065 min (major). Anal. Calcd for $C_{35}H_{42}FN_3O_8$: C, 64.50; H, 6.50; F, 2.92; N, 6.45. Found: C, 64.51; H, 6.52; F, 2.93; N, 6.46. HRMS (MALDI-TOF) m/z: $[M + H]^+$ Calcd for $C_{35}H_{42}FN_3O_8$ 651.2956; Found 651.2654.

FT-IR (KBr): ν_{as}(N-H)$_{carbamate}$ =3436.49 cm^{-1}, ν_s(N-H)$_{carbamate}$=3391.46 cm^{-1}, ν_s(N-H)$_{lactam}$=3332.08 cm^{-1}, ν(O-H)$_{phenol}$ =3297.02 cm^{-1}, ν(O-H) =3183.48 cm^{-1}, ν_{as}(C-H) =2926.83 cm^{-1}, ν_s(C-H) =2825.05 cm^{-1}, ν(C=O)$_{carbamate}$ =1733.35 cm^{-1}, ν(C=O)$_{lactam}$=1699.44 cm^{-1}, ν(C=N) =1647.18 cm^{-1}, ν(C=C) =1614.52 cm^{-1} , δ(N-H) =1533.62 cm^{-1}, ν(C-F) =1499.19 cm^{-1}, δ(N-H)$_{carbamate}$ =1478.96 cm^{-1}, ν(C-N)$_{carbamate}$ =1379.07 cm^{-1}, ν(C-O-C)$_{carbamate}$ =1177.85 cm^{-1}, ν(C-O-C) =1106.75 cm^{-1}, ν(C-O-C)$_{methoxy}$ =1061.80 cm^{-1} , γ(=C-H)$_{ar}$=785.14 cm^{-1} , γ(N-H)$_{carbamate}$=681.40 cm^{-1}.^{1}H NMR (500 MHz, DMSO-d_6+ACN-d_3) δ 9.45 (s, 1H, NH-1), 8.53 (s, 1H, OH-21), 8.24 – 8.15 (m, 2H, H-31,35), 7.45 – 7.40 (m, 2H, H-32,34), 7.30 (s, 1H, H-19), 6.39 (s, 1H, NH$_2$-24), 6.30 (t, $J^3_{H-3,H-4}$= 11.5 Hz, 1H, H-4), 6.22 (s, 1H, NH$_2$-24), 6.00 (d, $J^3_{H-3,H-4}$= 11.2 Hz, 1H, H-3), 4.95 – 4.87 (m, 2H, H-5,9), 4.67 (d, $J^3_{H-6,H-7}$= 9.6 Hz, 1H, H-7), 4.19 (s, 1H, OH-11), 3.73 (t, $J^3_{H-6,H-7}$= 10.3 Hz, 1H, H-6), 3.29 – 3.23 (m, 1H, H-11), 3.19 (s, 3H, H-27), 3.12 – 3.06 (m, 1H, H-15), 2.95 (s, 3H, H-23), 2.89 (s, 1H, H-15), 2.71 – 2.63 (m, 1H, H-12), 2.29 – 2.20 (m, 1H, H-14), 1.96 (s, 3H, H-22), 1.73 – 1.68 (m, 2H, H-10,13), 0.72 (d, $J^3_{H-14,H-28}$= 6.6 Hz, 3H, H-28), 0.63 (d, $J^3_{H-10,H-26}$= 6.3 Hz, 3H, H-26), 0.54 – 0.45 (m, 1H, H-13), 0.32 (s, 3H, H-25).^{13}C{1H} NMR (126 MHz, DMSO-d_6+ ACN-d$_3$) δ 174.0 (C-1), 164.6 (d, J^1= 249.9 Hz, C-33), 161.4 (C-29), 156.4 (C-24), 148.0 (C-21), 144.3 (C-18), 140.6 (C-17), 135.8 (C-2), 133.8 (C-9), 129.8 (C-4), 129.7* (d, J^3= 8.4 Hz, C-31,35), 129.1 (C-8), 127.6 (C-30), 127.0 (C-5), 124.1 (C-20), 123.4 (C-3), 119.2 (C-16), 116.9* (d, J^2= 22.2 Hz, C-32,34), 104.8 (C-19), 80.4 (C-7), 79.8 (C-12), 74.5 (C-6), 71.4 (C-11), 55.8 (C-27), 55.3 (C-23), 34.9 (C-10), 31.6 (C-15), 31.2 (C-13), 30.4 (C-14), 18.6 (C-26), 16.7 (C-28), 14.0 (C-22), 9.8 (C-25). (*-overlapped)

Compound ***3a.*** (4*E*,6*Z*,8*S*,9*S*,10*E*,12*S*,13*R*,14*S*,16*R*)-1^2-(4-chlorophenyl)-1^5,13-dihydroxy-8,14-dimethoxy-4,10,12,16-tetramethyl-3-oxo-2-aza-1(6,4)-benzo[*d*]oxazolacycloheptadecaphane-4,6,10-trien-9-yl carbamate (68 mg, yield 68 %), mp 229-230 ºC, HPLC (OD-H, water/acetonitrile = 35/65, flow rate = 1.0 mL/min, l = 260 nm) tR = 5.345 min (major). Anal. Calcd for $C_{35}H_{42}ClN_3O_8$: C, 62.91; H, 6.34; Cl, 5.31; N, 6.29. Found: C, 62.90; H, 6.33; Cl, 5.32; N, 6.31. HRMS (MALDI-TOF) m/z: [M + H]$^+$ Calcd for $C_{35}H_{42}ClN_3O_8$ 667.2660; Found 667.4203. FT-IR (KBr): ν_{as}(N-H)$_{carbamate}$ =3437.25 cm^{-1}, ν_s(N-H)$_{carbamate}$=3391.19 cm^{-1}, ν_s(N-H)$_{lactam}$=3332.09 cm^{-1}, ν(O-H)$_{phenol}$ =3301.07 cm^{-1}, ν(O-H) =3181.64 cm^{-1}, ν_{as}(C-H) =2923.19 cm^{-1}, ν_s(C-H) =2850.57 cm^{-1}, ν(C=O)$_{carbamate}$ =1732.71 cm^{-1}, ν(C=O)$_{lactam}$=1698.50 cm^{-1}, ν(C=N) =1646.47 cm^{-1}, ν(C=C) =1614.55 cm^{-1} , δ(N-H) =1535.29 cm^{-1}, δ(N-H)$_{carbamate}$ =1483.43 cm^{-1}, ν(C-N)$_{carbamate}$ =1378.90 cm^{-1}, ν(C-O-C)$_{carbamate}$ =1178.01 cm^{-1}, ν(C-O-C) =1104.94 cm^{-1}, ν(C-O-C)$_{methoxy}$ =1062.06 cm^{-1} , γ(=C-H)$_{ar}$=785.97 cm^{-1} , γ(N-H)$_{carbamate}$=680.09 cm^{-1}.^{1}H NMR (500 MHz, DMSO-d_6+ACN-d_3) δ 9.47 (s, 1H, NH-1), 8.59 (s, 1H, OH-21), 8.12 (d, $J^3_{H-31,35,H-32,34}$= 7.9 Hz, 2H, H-31,35), 7.66 (d, $J^3_{H-31,35,H-32,34}$= 8.9 Hz, 2H, H-32,34), 7.31 (s, 1H, H-19), 6.39 (s, 1H, NH$_2$-24), 6.30 (t, $J^3_{H-3,H-4}$= 11.4 Hz, 1H, H-4), 6.22 (s, 1H, NH$_2$-24), 6.00 (d, $J^3_{H-3,H-4}$= 11.6 Hz, 1H, H-3), 4.94 (d, $J^3_{H-9,H-10}$= 10.7 Hz, 1H, H-9), 4.90 (d, $J^3_{H-5,H-6}$= 10.7 Hz, 1H, H-5), 4.67 (d, $J^3_{H-6,H-7}$= 9.6 Hz, 1H, H-7), 4.20 (s, 1H, OH-11), 3.73 (t, $J^3_{H-6,H-7}$= 10.2 Hz, 1H, H-6), 3.52 (s, 1H, H-11), 3.28 – 3.25 (m, 1H, H-15), 3.19 (s, 3H, H-27), 3.14 – 3.06 (m, 1H, H-15), 2.95 (s, 3H, H-23), 2.70 – 2.62 (m, 1H, H-12), 2.32 – 2.25 (m, 1H, H-14), 1.96 (s, 3H, H-22), 1.82 – 1.72 (m, 2H, H-10,13), 0.73 (d, $J^3_{H-14,H-28}$= 6.5 Hz, 3H, H-28), 0.63 (d, $J^3_{H-10,H-26}$ = 6.4 Hz, 3H, H-26), 0.54 – 0.45 (m, 1H, H-13), 0.31 (s, 3H, H-25).^{13}C{1H} NMR (126 MHz, DMSO-d_6+ ACN-d$_3$) δ 174.0 (C-1), 161.2 (C-29), 156.4 (C-24), 148.1 (C-21), 144.4 (C-18), 140.6 (C-17), 135.8 (C-2), 133.8* (C-9,33), 130.1 (C-4), 129.9 (C-31), 129.8 (C-35), 129.1 (C-8), 128.8* (C-32,34), 127.9 (C-30), 127.6 (C-20), 126.3 (C-5), 123.4 (C-3), 119.3 (C-16), 104.8 (C-19), 80.4 (C-7), 79.7 (C-12), 74.4 (C-6), 71.5 (C-11), 55.8 (C-27), 55.3 (C-23), 34.9 (C-10), 32.0 (C-15), 31.5 (C-13), 30.4 (C-14), 18.7 (C-26), 16.8 (C-28), 14.2 (C-22), 9.7 (C-25). (*-overlapped)

Compound ***4a.*** (4*E*,6*Z*,8*S*,9*S*,10*E*,12*S*,13*R*,14*S*,16*R*)-1^2-(4-bromophenyl)-1^5,13-dihydroxy-8,14-dimethoxy-4,10,12,16-tetramethyl-3-oxo-2-aza-1(6,4)-benzo[*d*]oxazolacycloheptadecaphane-4,6,10-trien-9-yl carbamate (72 mg, yield 72 %), mp 248-250 ºC, HPLC (OD-H, water/acetonitrile = 35/65, flow rate = 1.0 mL/min, l = 260 nm) tR = 5.752 min (major). Anal. Calcd for $C_{35}H_{42}BrN_3O_8$: C, 58.99; H, 5.94; Br, 11.21; N, 5.90. Found: C, 59.01; H, 5.93; Br, 11.20; N, 5.93. HRMS (MALDI-TOF) m/z: [M + H]$^+$ Calcd for $C_{35}H_{42}BrN_3O_8$ 711.2155; Found 711.3469. FT-IR (KBr): ν_{as}(N-H)$_{carbamate}$ =3436.67 cm^{-1}, ν_s(N-H)$_{carbamate}$=3392.17 cm^{-1}, ν_s(N-H)$_{lactam}$=3332.47 cm^{-1}, ν(O-H)$_{phenol}$ =3288.91 cm^{-1}, ν(O-H) =3181.83 cm^{-1}, ν_{as}(C-H) =2924.41 cm^{-1}, ν_s(C-H) =2851.23 cm^{-1}, ν(C=O)$_{carbamate}$ =1731.59 cm^{-1}, ν(C=O)$_{lactam}$=1698.29 cm^{-1}, ν(C=N) =1646.92 cm^{-1}, ν(C=C) =1614.77 cm^{-1} , δ(N-H) =1533.97 cm^{-1}, δ(N-H)$_{carbamate}$ =1481.15 cm^{-1}, ν(C-N)$_{carbamate}$ =1378.11 cm^{-1}, ν(C-O-C)$_{carbamate}$ =1193.35 cm^{-1}, ν(C-O-C) =1105.51 cm^{-1}, ν(C-O-C)$_{methoxy}$ =1069.33 cm^{-1} , γ(=C-H)$_{ar}$=785.72 cm^{-1} , γ(N-H)$_{carbamate}$=679.97 cm^{-1}.^{1}H NMR (500 MHz, DMSO-d_6+ ACN-d$_3$) δ 9.46 (s, 1H, NH-1), 8.56 (s, 1H, OH-21), 8.05 (d, $J^3_{H-31,35,H-32,34}$= 8.4 Hz, 2H, H-31,35), 7.81 (d, $J^3_{H-31,35,H-32,34}$= 8.5 Hz, 2H, H-32,34), 7.31 (s, 1H, H-19), 6.39 (s, 1H, NH$_2$-24), 6.30 (t, $J^3_{H-3,H-4}$= 11.4 Hz, 1H, H-4), 6.22 (s, 1H, NH$_2$-24), 6.00 (d, $J^3_{H-3,H-4}$= 11.6 Hz, 1H, H-3), 4.98 – 4.87 (m, 2H, H-5,9), 4.67 (d, $J^3_{H-6,H-7}$= 9.6 Hz, 1H, H-7), 4.20 (s, 1H, OH-11), 3.73 (t, $J^3_{H-6,H-7}$= 10.1 Hz, 1H, H-6), 3.52 (s, 1H, H-11), 3.19 (s, 3H, H-27), 3.09 (d, J^2 = 12.4 Hz, 1H, H-15), 2.95 (s, 3H, H-23), 2.89 (s, 1H, H-15), 2.70 – 2.64 (m, 1H, H-12), 2.28 – 2.21 (m, 1H, H-14), 1.96 (s, 3H, H-22), 1.80 – 1.73 (m, 1H, H-10), 1.71 – 1.65 (m, 1H, H-13), 0.73 (d, $J^3_{H-14,H-28}$= 6.5 Hz, 3H, H-28), 0.63 (d, $J^3_{H-10,H-26}$= 6.3 Hz, 3H, H-26), 0.51 (d, J^2 = 13.7 Hz, 1H, H-13), 0.31 (s, 3H, H-25).^{13}C{1H} NMR (126 MHz, DMSO-d_6+ ACN-d$_3$) δ 174.0 (C-1), 161.3 (C-29), 156.4 (C-24), 148.1 (C-21), 144.3 (C-18), 140.5 (C-17), 135.7 (C-2), 133.8 (C-9), 132.7 (C-32), 132.6 (C-34), 130.1 (C-4), 129.7

(C-30), 129.1 (C-8), 128.8* (C-31,35), 127.6 (C-5), 126.5 (C-20), 125.5 (C-33), 123.3 (C-3), 119.2 (C-16), 104.8 (C-19), 80.3 (C-7), 79.8 (C-12), 74.4 (C-6), 71.2 (C-11), 55.8 (C-27), 55.3 (C-23), 34.9 (C-10), 32.0 (C-15), 31.6 (C-13), 30.8 (C-14), 18.7 (C-26), 16.7 (C-28), 14.0 (C-22), 9.7 (C-25). (*-overlapped)

Compound ***5a.*** (4*E*,6*Z*,8*S*,9*S*,10*E*,12*S*,13*R*,14*S*,16*R*)-1^5,13-dihydroxy-1^2-(4-iodophenyl)-8,14-dimethoxy-4,10,12,16-tetramethyl-3-oxo-2-aza-1(6,4)-benzo[*d*]oxazolacycloheptadecaphane-4,6,10-trien-9-yl carbamate (84 mg, 84 %), tt. 248-250 ºC, HPLC (OD-H, water/acetonitrile = 35/65, flow rate = 1.0 mL/min, l = 260 nm) tR = 5.778 min (major). Anal. Calcd for $C_{35}H_{42}IN_3O_8$: C, 55.34; H, 5.57; I, 16.71; N, 5.53. Found: C, 55.31; H, 5.54; I, 16.70; N, 5.54. HRMS (MALDI-TOF) m/z: $[M + H]^+$ Calcd for $C_{35}H_{42}IN_3O_8$ 759.2017; Found 759.2023. FT-IR (KBr): ν_{as}(N-H)$_{carbamate}$ =3439 cm^{-1}, ν_s(N-H)$_{carbamate}$=3393 cm^{-1}, ν_s(N-H)$_{lactam}$=3337 cm^{-1}, ν(O-H)$_{phenol}$=3295 cm^{-1}, ν(O-H)=3181 cm^{-1}, ν_{as}(C-H)=2924 cm^{-1}, ν_s(C-H)=2823 cm^{-1}, ν(C=O)$_{carbamate}$=1732 cm^{-1}, ν(C=O)$_{lactam}$=1698 cm^{-1}, ν(C=N)=1645 cm^{-1}, ν(C=C) =1613 cm^{-1}, δ(N-H)=1535 cm^{-1}, δ(N-H)$_{carbamate}$=1478 cm^{-1}, ν(C-N)$_{carbamate}$ =1378 cm^{-1}, ν(C-I)=1328 cm^{-1}, ν(C-O-C)$_{carbamate}$=1174 cm^{-1}, ν(C-O-C) =1106 cm^{-1}, ν(C-O-C)$_{methoxy}$=1061 cm^{-1} , γ(=C-H)$_{ar}$=784 cm^{-1} , γ(N-H)$_{carbamate}$=679 cm^{-1}. ^{1}H NMR (500 MHz, DMSO-d_6+ ACN-d_3) δ 9.41 (s, 1H, NH-1), 8.52 (s, 1H, OH-21), 7.99 (d, $J^3_{H-31,35,H-32,34}$= 8.5 Hz, 2H, H-31,35), 7.89 (d, $J^3_{H-31,35,H-32,34}$= 8.5 Hz, 2H, H-32,34), 7.29 (s, 1H, H-19), 6.35 – 6.26 (m, 1H, H-4), 6.34 (s, 1H, NH_2-24), 6.19 (s, 1H, NH_2-24), 6.01 (d, $J^3_{H-3,H-4}$= 11.6 Hz, 1H, H-3), 5.00 – 4.87 (m, 2H, H-5,9), 4.71 – 4.69 (m, 1H, H-7), 4.11 (s, 1H, OH-11), 3.74 (t, $J^3_{H-6,H-7}$= 10.2 Hz, 1H, H-6), 3.28 (s, 1H, H-11), 3.20 (s, 3H, H-27), 3.11-3.04 (m, 1H, H-15), 2.97 (bs, 1H, H-15), 2.95 (s, 3H, H-23), 2.68 (bs, 1H, H-12), 2.28 – 2.21 (m, 1H, H-14), 1.96 (s, 3H, H-22), 1.81 – 1.69 (m, 1H, H-10), 1.68 – 1.57 (m, 1H, H-13), 0.75-0.71 (m, 3H, H-28), 0.64 (d, $J^3_{H-10,H-26}$= 6.3 Hz, 3H, H-26), 0.51-0.44 (m, 1H, H-13), 0.31 (s, 3H, H-25).^{13}C{1H} NMR (126 MHz, DMSO-d_6+ ACN-d_3) δ 174.1 (C-1),161.6 (C-29), 156.5 (C-24), 148.1 (C-21), 144.4 (C-18), 140.6 (C-17), 138.7 (C-32), 138.6 (C-34), 135.7 (C-2), 133.7 (C-9), 129.8 (C-4), 129.1 (C-8), 128.8* (C-31,35), 127.8 (C-5), 127.6 (C-20), 127.0 (C-30), 123.5 (C-3), 119.3 (C-16), 104.8 (C-19), 99.1 (C-33), 80.4 (C-7), 79.8 (C-12), 74.5 (C-6), 71.5 (C-11), 55.8 (C-27), 55.3 (C-23), 34.9 (C-10), 31.6 (C-15), 31.2 (C-13), 30.4 (C-14), 18.6 (C-26), 16.8 (C-28), 13.9 (C-22), 9.7 (C-25). (*-overlapped)

Compound ***6a.*** (4*E*,6*Z*,8*S*,9*S*,10*E*,12*S*,13*R*,14*S*,16*R*)-1^5,13-dihydroxy-8,14-dimethoxy-4,10,12,16-tetramethyl-3-oxo-1^2-(4-(trifluoromethyl)phenyl)-2-aza-1(6,4)-benzo[*d*]oxazolacycloheptadecaphane-4,6,10-trien-9-yl carbamate (76 mg, yield 76 %), mp 248-250 ºC, HPLC (OD-H, water/acetonitrile = 35/65, flow rate = 1.0 mL/min, l = 260 nm) tR = 5.778 min (major). Anal. Calcd for $C_{36}H_{42}F_3N_3O_8$: C, 61.62; H, 6.03; F, 8.12; N, 5.99. Found: C, 61.60; H, 6.00; F, 8.11; N, 6.00. HRMS (MALDI-TOF) m/z: $[M + H]^+$ Calcd for $C_{36}H_{42}F_3N_3O_8$ 701.2924; Found 701.3189. FT-IR (KBr): ν_{as}(N-H)$_{carbamate}$ =3438.71 cm^{-1}, ν_s(N-H)$_{carbamate}$=3392.06 cm^{-1}, ν_s(N-H)$_{lactam}$=3339.76 cm^{-1}, ν(O-H)$_{phenol}$ =3286.89 cm^{-1}, ν(O-H) =3185.93 cm^{-1}, ν_{as}(C-H) =2927.85 cm^{-1}, ν_s(C-H) =2825.05 cm^{-1}, ν(C=O)$_{carbamate}$ =1731.78 cm^{-1}, ν(C=O)$_{lactam}$=1697.99 cm^{-1}, ν(C=N) =1648.20 cm^{-1}, ν(C=C) =1622.01 cm^{-1} , δ(N-H) =1535.57 cm^{-1}, δ(N-H)$_{carbamate}$ =1481.91 cm^{-1}, ν(C-N)$_{carbamate}$ =1379.09 cm^{-1}, ν(C-F) =1327.93 cm^{-1}, ν(C-O-C)$_{carbamate}$ =1174.58 cm^{-1}, ν(C-O-C) =1109.90 cm^{-1}, ν(C-O-C)$_{methoxy}$ =1070.49 cm^{-1} , γ(=C-H)$_{ar}$=786.36 cm^{-1} , γ(N-H)$_{carbamate}$=679.49 cm^{-1}.^{1}H NMR (500 MHz, DMSO-d_6+ ACN-d_3) δ 9.50 (s, 1H, NH-1), 8.62 (s, 1H, OH-21), 8.32 (d, $J^3_{H-31,35,H-32,34}$= 8.3 Hz, 2H, H-31,35), 7.96 (d, $J^3_{H-31,35,H-32,34}$= 8.4 Hz, 2H, H-32,34), 7.35 (s, 1H, H-19), 6.43 – 6.36 (m, 1H, NH_2-24), 6.30 (t, $J^3_{H-3,H-4}$= 11.5 Hz, 1H, H-4), 6.22 (s, 1H, NH_2-24), 6.02 (d, $J^3_{H-3,H-4}$= 11.6 Hz, 1H, H-3), 4.99 – 4.92 (m, 1H, H-5), 4.92 – 4.87 (m, 1H, H-9), 4.67 (d, $J^3_{H-6,H-7}$= 9.8 Hz, 1H, H-7), 4.21 (s, 1H, OH-11), 3.73 (t, $J^3_{H-6,H-7}$= 10.1 Hz, 1H, H-6), 3.52 (s, 1H, H-11), 3.26 (d, J^2= 12.4 Hz, 1H, H-15), 3.19 (s, 3H, H-27), 3.10 (d, J^2= 12.9 Hz, 1H, H-15), 2.96 (s, 3H, H-23), 2.71 – 2.64 (m, 1H, H-12), 2.27 (s, 1H, H-14), 1.96 (s, 3H, H-22), 1.81 – 1.72 (m, 1H, H-10), 1.71 – 1.63 (m, 1H, H-13), 0.74 (d, $J^3_{H-14,H-28}$= 6.4 Hz, 3H, H-28), 0.61 (d, $J^3_{H-10,H-26}$= 6.1 Hz, 3H, H-26), 0.55 – 0.45 (m, 1H, H-13), 0.31 (s, 3H, H-25).^{13}C{1H} NMR (126 MHz, DMSO-d_6+ ACN-d_3) δ 174.0 (C-1), 160.7 (C-29), 156.4 (C-24), 148.2 (C-21), 144.5 (C-18), 140.5 (C-17), 135.8 (C-2), 133.8 (C-9), 131.1 (C-33), 129.8 (C-4), 129.0 (C-8), 128.4 (C-8), 127.8* (C-31,35), 126.7* (C-32,34), 126.7 (C-5), 126.6 (C-20), 125.4 (C-36), 123.5 (C-3), 119.5 (C-16), 104.9 (C-19), 80.4 (C-7), 79.8 (C-12), 74.4 (C-6), 71.5 (C-11), 55.8 (C-27), 55.3 (C-23), 34.9 (C-10), 31.6 (C-15), 31.1 (C-13), 30.4 (C-14), 18.6 (C-26), 16.8 (C-28), 13.9 (C-22), 9.7 (C-25). (*-overlapped)

Compound ***7a.*** (4*E*,6*Z*,8*S*,9*S*,10*E*,12*S*,13*R*,14*S*,16*R*)-1^5,13-dihydroxy-8,14-dimethoxy-4,10,12,16-tetramethyl-3-oxo-1^2-(4-(trifluoromethoxy)phenyl)-2-aza-1(6,4)-benzo[*d*]oxazolacycloheptadecaphane-4,6,10-trien-9-yl carbamate (82 mg, yield 82 %), mp 220-221 ºC, HPLC (OD-H, water/acetonitrile = 35/65, flow rate = 1.0 mL/min, l = 260 nm) tR = 5.725 min (major). Anal. Calcd for $C_{36}H_{42}F_3N_3O_9$: C, 60.24; H, 5.90; F, 7.94; N, 5.85. Found: C, 60.23; H, 5.91; F, 7.92; N, 5.82. HRMS (MALDI-TOF) m/z: $[M + H]^+$ Calcd for $C_{36}H_{42}F_3N_3O_9$ 717.2873; Found 717.1927. FT-IR (KBr): ν_{as}(N-H)$_{carbamate}$ =3438.36 cm^{-1}, ν_s(N-H)$_{carbamate}$=3395.24 cm^{-1}, ν_s(N-H)$_{lactam}$=3339.36 cm^{-1}, ν(O-H)$_{phenol}$ =3301.07 cm^{-1}, ν(O-H) =3185.62 cm^{-1}, ν_{as}(C-H) =2927.37 cm^{-1}, ν_s(C-H) =2824.60 cm^{-1}, ν(C=O)$_{carbamate}$ =1732.08 cm^{-1}, ν(C=O)$_{lactam}$=1698.10 cm^{-1}, ν(C=N) =1646.94 cm^{-1}, ν(C=C) =1614.63 cm^{-1} , δ(N-H) =1534.18 cm^{-1}, δ(N-H)$_{carbamate}$ =1480.94 cm^{-1}, ν(C-N)$_{carbamate}$ =1379.07 cm^{-1}, ν(C-O-C)$_{carbamate}$ =1176.70 cm^{-1}, ν(C-O-C) =1105.25 cm^{-1}, ν(C-O-C)$_{methoxy}$ =1061.28 cm^{-1} , γ(=C-H)$_{ar}$=785.29 cm^{-1} , γ(N-H)$_{carbamate}$=706.82 cm$^-$

1.^{1}H NMR (500 MHz, DMSO-d_6+ ACN-d_3) δ 9.45 (s, 1H, NH-1), 8.55 (s, 1H, OH-21), 8.22 (d, $J^3_{H-31,35,H-32,34}$= 8.6 Hz, 2H, H-31,35), 7.56 (d, $J^3_{H-31,35,H-32,34}$= 8.7 Hz, 2H, H-32,34), 7.30 (s, 1H, H-19), 6.37 (s, 1H, NH_2-24), 6.28 (t, $J^3_{H-3,H-4}$= 11.4 Hz, 1H, H-4), 6.20 (s, 1H, NH_2-24), 5.99 (d, $J^3_{H-3,H-4}$= 11.6 Hz, 1H, H-3), 4.98 – 4.86 (m, 2H, H-5,9), 4.68 – 4.60 (m, 1H, H-7), 4.18 (s, 1H, OH-11), 3.72 (t, $J^3_{H-6,H-7}$= 10.1 Hz, 1H, H-6), 3.26 – 3.20 (m, 1H, H-11), 3.17 (s, 3H, H-27), 3.08 (d, J^2 = 12.2 Hz, 1H, H-15), 2.94 (s, 3H, H-23), 2.87 (s, 1H, H-15), 2.65 (bs, 1H, H-12), 2.23 (bs, 1H, H-14), 1.94 (s, 3H, H-22), 1.84 – 1.70 (m, 1H, H-10), 1.70 – 1.64 (m, 1H, H-13), 0.71 (d, $J^3_{H-14,H-28}$= 6.4 Hz, 3H, H-28), 0.59 (d, $J^3_{H-10,H-26}$= 6.1 Hz, 3H, H-26), 0.52 – 0.43 (m, 1H, H-13), 0.30 (s, 3H, H-25).^{13}C{1H} NMR (126 MHz, DMSO-d_6+ ACN-d_3) δ 174.0 (C-1), 160.9 (C-29), 156.4 (C-24), 150.9 (C-33), 148.1 (C-21), 144.4 (C-18), 140.6 (C-17), 135.8 (C-2), 133.8 (C-9), 132.2 (C-30), 130.0 (C-4), 129.8 (C-36), 129.3* (C-31,35), 129.0 (C-8), 127.6 (C-5), 126.6 (C-20), 123.5 (C-3), 122.2* (C-31,35), 119.3 (C-16), 104.9 (C-19), 80.4 (C-7), 79.8 (C-12), 74.5 (C-6), 71.4 (C-11), 55.8 (C-27), 55.3 (C-23), 34.9 (C-10), 31.6 (C-15), 31.2 (C-13), 30.4 (C-14), 18.5 (C-26), 16.8 (C-28), 14.0 (C-22), 9.7 (C-25). (*-overlapped), bs - broad singlet

Compound ***8a.*** (4*E*,6*Z*,8*S*,9*S*,10*E*,12*S*,13*R*,14*S*,16*R*)-1^2-(4-cyanophenyl)-1^5,13-dihydroxy-8,14-dimethoxy-4,10,12,16-tetramethyl-3-oxo-2-aza-1(6,4)-benzo[*d*]oxazolacycloheptadecaphane-4,6,10-trien-9-yl carbamate (81 mg, yield 81 %), mp 222-223 ºC, HPLC (OD-H, water/acetonitrile = 35/65, flow rate = 1.0 mL/min, l = 260 nm) tR = 3.692 min (major). Anal. Calcd for $C_{36}H_{42}N_4O_8$: C, 65.64; H, 6.43; N, 8.51. Found: C, 65.66; H, 6.44; N, 8.50. HRMS (MALDI-TOF) m/z: $[M + H]^+$ Calcd for $C_{36}H_{42}N_4O_8$ 658.3003; Found 658.2941. FT-IR (KBr): $ν_{as}$(N-H)$_{carbamate}$ =3433.74 cm^{-1}, $ν_s$(N-H)$_{carbamate}$=3390.72 cm^{-1}, $ν_s$(N-H)$_{lactam}$=3339.52 cm^{-1}, ν(O-H)$_{phenol}$ =3292.97 cm^{-1}, ν(O-H) =3185.13 cm^{-1}, $ν_{as}$(C-H) =2930.27 cm^{-1}, $ν_s$(C-H) =2825.86 cm^{-1}, ν(C-N) =2229.72 cm^{-1}, ν(C=O)$_{carbamate}$ =1730.12 cm^{-1}, ν(C=O)$_{lactam}$=1696.41 cm^{-1}, ν(C=N) =1649.94 cm^{-1}, ν(C=C) =1616.68 cm^{-1} , δ(N-H) =1534.07 cm^{-1}, δ(N-H)$_{carbamate}$ =1481.10 cm^{-1}, ν(C-N)$_{carbamate}$ =1378.55 cm^{-1}, ν(C-O-C)$_{carbamate}$ =1177.62 cm^{-1}, ν(C-O-C) =1104.84 cm^{-1}, ν(C-O-C)$_{methoxy}$ =1060.70 cm^{-1} , γ(=C-H)$_{ar}$=785.26 cm^{-1} , γ(N-H)$_{carbamate}$=688.75 cm^{-1}. ^{1}H NMR (500 MHz, DMSO-d_6+ ACN-d_3) δ 9.57 (s, 1H, NH-1), 8.34 (bs, 1H, OH-21), 8.04 (d, $J^3_{H-31,35,H-32,34}$= 7.9 Hz, 2H, H-31,35), 7.57 (d, $J^3_{H-31,35,H-32,34}$= 7.9 Hz, 2H, H-32,34), 7.34 (s, 1H, H-19), 6.50 (s, 1H, NH_2-24), 6.32 – 6.28 (m, 2H, H-4, NH_2-24), 6.01 (d, $J^3_{H-3,H-4}$= 11.5 Hz, 1H, H-3), 4.96 – 4.91 (m, 1H, H-5), 4.91 – 4.87 (m, 1H, H-9), 4.66 (d, $J^3_{H-6,H-7}$= 9.6 Hz, 1H, H-7), 4.30 (s, 1H, OH-11), 3.72 (t, $J^3_{H-6,H-7}$= 10.2 Hz, 1H, H-6), 3.44 – 3.37 (m, 1H, H-11), 3.18 (s, 3H, H-27), 3.13 – 3.07 (m, 1H, H-15), 2.94 (s, 3H, H-23), 2.93 (bs, 1H, H-15), 2.67 – 2.59 (m, 1H, H-12), 2.23 (bs, 1H, H-14), 1.95 (s, 3H, H-22), 1.71 (bs, 1H, H-10), 1.68 (bs, 1H, H-13), 0.71 (d, $J^3_{H-14,H-28}$= 6.4 Hz, 3H, H-28), 0.60 (d, $J^3_{H-10,H-26}$= 6.3 Hz, 3H, H-26), 0.45 (t, J^2 = 12.9 Hz, 1H, H-13), 0.26 (s, 3H, H-25).^{13}C{1H} NMR (126 MHz, DMSO-d_6+ ACN-d_3) δ 174.2 (C-1), 160.6 (C-29), 156.5 (C-24), 148.4 (C-21), 144.6 (C-18), 140.6 (C-17), 137.9 (C-30), 135.9 (C-2), 133.9 (C-9), 132.8* (C-32,34), 130.0 (C-4), 129.0 (C-8), 128.8* (C-31,35), 127.7(C-5), 127.3 (C-20), 123.6 (C-3), 119.6 (C-16), 119.5 (C-36), 113.0 (C-33), 105.0 (C-19), 80.4 (C-7), 79.8 (C-12), 74.5 (C-6), 71.3 (C-11), 55.9 (C-27), 55.4 (C-23), 35.0 (C-10), 31.6 (C-15), 31.2 (C-13), 30.5 (C-14), 18.8 (C-26), 16.7 (C-28), 14.1 (C-22), 9.8 (C-25). (*-overlapped), bs - broad singlet

Compound ***9a.*** (4*E*,6*Z*,8*S*,9*S*,10*E*,12*S*,13*R*,14*S*,16*R*)-1^5,13-dihydroxy-8,14-dimethoxy-4,10,12,16-tetramethyl-1^2-(4-nitrophenyl)-3-oxo-2-aza-1(6,4)-benzo[*d*]oxazolacycloheptadecaphane-4,6,10-trien-9-yl carbamate (69 mg, yield 69 %), mp 224-226 ºC, HPLC (OD-H, water/acetonitrile = 35/65, flow rate = 1.0 mL/min, l = 260 nm) tR = 4.238 min (major). Anal. Calcd for $C_{35}H_{42}N_4O_{10}$: C, 61.94; H, 6.24; N, 8.25. Found: C, 61.96; H, 6.26; N, 8.26. HRMS (MALDI-TOF) m/z: $[M + H]^+$ Calcd for $C_{35}H_{42}N_4O_{10}$ 678.2901; Found 678.3214. FT-IR (KBr): $ν_{as}$(N-H)$_{carbamate}$ =3433.82 cm^{-1}, $ν_s$(N-H)$_{carbamate}$=3392.23 cm^{-1}, $ν_s$(N-H)$_{lactam}$=3339.69 cm^{-1}, ν(O-H)$_{phenol}$ =3288.91 cm^{-1}, ν(O-H) =3187.03 cm^{-1}, $ν_{as}$(C-H) =2929.00 cm^{-1}, $ν_s$(C-H) =2825.60 cm^{-1}, ν(C=O)$_{carbamate}$ =1729.56 cm^{-1}, ν(C=O)$_{lactam}$=1696.08 cm^{-1}, ν(C=N) =1656.93 cm^{-1}, ν(C=C) =1615.26 cm^{-1} , δ(N-H), $ν_{as}$(C-NO_2)=1526.67 cm^{-1}, δ(N-H)$_{carbamate}$ =1483.18 cm^{-1}, ν(C-N)$_{carbamate}$ =1378.13 cm^{-1}, $ν_s$(C-NO_2)=1344.43 cm^{-1}, ν(C-O-C)$_{carbamate}$ =1177.34 cm^{-1}, ν(C-O-C) =1106.35 cm^{-1}, ν(C-O-C)$_{methoxy}$ =1059.62 cm^{-1} , γ(=C-H)$_{ar}$=785.59 cm^{-1} , γ(N-H)$_{carbamate}$=710.20 cm^{-1}.^{1}H NMR (500 MHz, DMSO-d_6+ ACN-d_3) δ 9.59 (s, 1H, NH-1), 8.76 (s, 1H, OH-21), 8.45 (d, $J^3_{H-31,35,H-32,34}$= 8.5 Hz, 2H, H-32,34), 8.34 (d, $J^3_{H-31,35,H-32,34}$= 8.5 Hz, 2H, H-31,35), 7.36 (s, 1H, H-19), 6.44 (s, 1H, NH_2-24), 6.34 – 6.25 (m, 2H, H-4, NH_2-24), 6.02 (d, $J^3_{H-3,H-4}$= 11.5 Hz, 1H, H-3), 4.96 – 4.87 (m, 2H, H-5,9), 4.66 (d, $J^3_{H-6,H-7}$= 9.6 Hz, 1H, H-7), 4.31 (s, 1H, OH-11), 3.73 (t, $J^3_{H-6,H-7}$= 10.1 Hz, 1H, H-6), 3.28 – 3.24 (m, 1H, H-11), 3.18 (s, 3H, H-27), 3.14 – 3.07 (m, 1H, H-15), 2.94 (s, 3H, H-23), 2.68 – 2.62 (m, 2H, H-12,15), 2.30 – 2.23 (m, 1H, H-14), 1.96 (s, 3H, H-22), 1.70 (bs, 2H, H-10,13), 0.73 (d, $J^3_{H-14,H-28}$= 6.6 Hz, 3H, H-28), 0.61 (d, $J^3_{H-10,H-26}$= 6.3 Hz, 3H, H-26), 0.53 – 0.44 (m, 1H, H-13), 0.27 (s, 3H, H-25).^{13}C{1H} NMR (126 MHz, DMSO-d_6+ ACN-d_3) δ 174.2 (C-1), 160.3 (C-29), 156.5 (C-24), 149.4 (C-33), 148.5 (C-21), 144.8 (C-18), 140.7 (C-17), 137.9 (C-30), 135.9 (C-2), 133.8 (C-9), 133.0* (C-31,35), 129.9 (C-4), 129.0 (C-8), 128.9* (C-32,34), 128.1 (C-5,20), 125.1 (C-3), 119.6 (C-16), 105.1 (C-19), 80.4 (C-7), 79.9 (C-12), 74.6 (C-6), 71.5 (C-11), 55.5 (C-27), 55.2 (C-23), 35.1 (C-10), 32.1 (C-15), 30.6 (C-14), 29.9 (C-13), 18.7 (C-26), 16.8 (C-28), 14.0 (C-22), 9.7 (C-25). (*-overlapped), bs - broad singlet

Compound ***10a.*** (4*E*,6*Z*,8*S*,9*S*,10*E*,12*S*,13*R*,14*S*,16*R*)-1^5,13-dihydroxy-8,14-dimethoxy-4,10,12,16-tetramethyl-3-oxo-1^2-(p-tolyl)-2-aza-1(6,4)-benzo[*d*]oxazolacycloheptadecaphane-4,6,10-trien-9-yl carbamate (79 mg, yield 79 %), mp 241-242 ºC, HPLC (OD-H, water/acetonitrile = 35/65, flow rate = 1.0 mL/min, l = 260 nm) tR = 4.378 min (major). Anal. Calcd for $C_{36}H_{45}N_3O_8$: C, 66.75; H, 7.00; N, 6.49. Found: C, 66.74; H, 7.02; N, 6.50. HRMS (MALDI-TOF) m/z: $[M + H]^+$ Calcd for $C_{36}H_{45}N_3O_8$ 647.3207; Found 647.3139. FT-IR (KBr): $\nu_{as}(N\text{-}H)_{carbamate}$ =3437.35 cm^{-1}, $\nu_s(N\text{-}H)_{carbamate}$=3392.79 cm^{-1}, $\nu_s(N\text{-}H)_{lactam}$=3337.69 cm^{-1}, $\nu(O\text{-}H)_{phenol}$ =3292.97 cm^{-1}, ν(O-H) =3183.55 cm^{-1}, ν_{as}(C-H) =2926.44 cm^{-1}, ν_s(C-H) =2824.98 cm^{-1}, $\nu(C{=}O)_{carbamate}$ =1733.40 cm^{-1}, $\nu(C{=}O)_{lactam}$=1699.71 cm^{-1}, ν(C=N) =1645.55 cm^{-1}, ν(C=C) =1615.72 cm^{-1} , δ(N-H) =1533.94 cm^{-1}, $\delta(N\text{-}H)_{carbamate}$ =1479.88 cm^{-1}, $\nu(C\text{-}N)_{carbamate}$ =1378.43 cm^{-1}, $\nu(C\text{-}O\text{-}C)_{carbamate}$ =1175.32 cm^{-1}, ν(C-O-C) =1105.47 cm^{-1}, $\nu(C\text{-}O\text{-}C)_{methoxy}$ =1061.72 cm^{-1} , $\gamma(=C\text{-}H)_{ar}$=783.60 cm^{-1} , $\gamma(N\text{-}H)_{carbamate}$=678.24 cm^{-1}. ^1H NMR (500 MHz, DMSO-d_6+ ACN-d_3) δ 9.43 (s, 1H, NH-1), 8.49 (s, 1H, OH-21), 8.02 (d, $J^3_{H\text{-}31,35,H\text{-}32,34}$= 7.9 Hz, 2H, H-31,35), 7.41 (d, $J^3_{H\text{-}31,35,H\text{-}32,34}$= 7.8 Hz, 2H, H-32,34), 7.29 (s, 1H, H-19), 6.39 (s, 1H, NH_2-24), 6.30 (t, $J^3_{H\text{-}3,H\text{-}4}$= 11.5 Hz, 1H, H-4), 6.21 (s, 1H, NH_2-24), 5.99 (d, $J^3_{H\text{-}3,H\text{-}4}$= 11.6 Hz, 1H, H-3), 4.93 – 4.87 (m, 2H, H-5,9), 4.69 – 4.63 (m, 1H, H-7), 4.19 (s, 1H, OH-11), 3.72 (t, $J^3_{H\text{-}6,H\text{-}7}$= 10.1 Hz, 1H, H-6), 3.28 – 3.23 (m, 1H, H-11), 3.19 (s, 3H, H-27), 3.08 (d, J^2 = 11.4 Hz, 1H, H-15), 2.95 (s, 3H, H-23), 2.89 (s, 1H, H-15), 2.70 – 2.65 (m, 1H, H-12), 2.42 (s, 3H, H-36), 2.24 (bs, 1H, H-14), 1.96 (s, 3H, H-22), 1.85 – 1.78 (m, 1H, H-10), 1.74 – 1.67 (m, 1H, H-13), 0.73 (d, $J^3_{H\text{-}14,H\text{-}28}$= 6.6 Hz, 3H, H-28), 0.63 (d, $J^3_{H\text{-}10,H\text{-}26}$= 6.3 Hz, 3H, H-26), 0.57 – 0.47 (m, 1H, H-13), 0.30 (s, 3H, H-25).$^{13}C\{1H\}$ NMR (126 MHz, DMSO-d_6+ ACN-d_3) δ 174.0 (C-1), 162.5 (C-29), 156.4 (C-24), 147.9 (C-21), 144.2 (C-18), 142.3 (C-33), 140.7 (C-17), 135.8 (C-2), 133.7 (C-9), 130.3* (C-31,35), 129.8 (C-4), 129.1 (C-8), 127.5 (C-5), 127.3* (C-32,34), 127.2 (C-20), 124.8 (C-30), 123.4 (C-3), 119.1 (C-16), 104.8 (C-19), 80.4 (C-7), 79.9 (C-12), 74.5 (C-6), 71.4 (C-11), 55.8 (C-27), 55.3 (C-23), 34.9 (C-10), 31.6 (C-15), 31.1 (C-13), 30.4 (C-14), 21.3 (C-36), 18.8 (C-26), 16.7 (C-28), 14.0 (C-22), 9.7 (C-25). (*-overlapped), bs - broad singlet

Compound ***11a.*** (4*E*,6*Z*,8*S*,9*S*,10*E*,12*S*,13*R*,14*S*,16*R*)-1^5,13-dihydroxy-8,14-dimethoxy-1^2-(4-methoxyphenyl)-4,10,12,16-tetramethyl-3-oxo-2-aza-1(6,4)-benzo[*d*]oxazolacycloheptadecaphane-4,6,10-trien-9-yl carbamate (85 mg, yield 85 %), mp 202-204 ºC, HPLC (OD-H, water/acetonitrile = 35/65, flow rate = 1.0 mL/min, l = 260 nm) tR = 3.552 min (major). Anal. Calcd for $C_{36}H_{45}N_3O_9$: C, 65.14; H, 6.83; N, 6.33. Found: C, 65.12; H, 6.84; N, 6.32. HRMS (MALDI-TOF) m/z: $[M + H]^+$ Calcd for $C_{36}H_{45}N_3O_9$ 663.3156; Found 663.3274. FT-IR (KBr): $\nu_{as}(N\text{-}H)_{carbamate}$ =3436.41 cm^{-1}, $\nu_s(N\text{-}H)_{carbamate}$=3395.05 cm^{-1}, $\nu_s(N\text{-}H)_{lactam}$=3333.48 cm^{-1}, $\nu(O\text{-}H)_{phenol}$ =3291.67 cm^{-1}, ν(O-H) =3181.41 cm^{-1}, ν_{as}(C-H) =2960.72 cm^{-1}, $\nu(C{=}O)_{carbamate}$ =1731.20 cm^{-1}, $\nu(C{=}O)_{lactam}$=1697.71 cm^{-1}, ν(C=N) =1645.48 cm^{-1}, ν(C=C) =1613.32 cm^{-1} , δ(N-H) =1535.66 cm^{-1}, $\delta(N\text{-}H)_{carbamate}$ =1480.22 cm^{-1}, $\nu(C\text{-}N)_{carbamate}$ =1378.09 cm^{-1}, $\nu(C\text{-}O\text{-}C)_{carbamate}$ =1167.35 cm^{-1}, ν(C-O-C) =1105.38 cm^{-1}, $\nu(C\text{-}O\text{-}C)_{methoxy}$ =1060.78 cm^{-1} , $\gamma(=C\text{-}H)_{ar}$=784.02 cm^{-1} , $\gamma(N\text{-}H)_{carbamate}$=681.67 cm^{-1}.^1H NMR (500 MHz, DMSO-d_6+ ACN-d_3) δ 9.47 (s, 1H, NH-1), 8.50 (s, 1H, OH-21), 8.07 (d, $J^3_{H\text{-}31,35,H\text{-}32,34}$= 8.6 Hz, 2H, H-31,35), 7.27 (s, 1H, H-19), 7.14 (d, $J^3_{H\text{-}31,35,H\text{-}32,34}$= 8.9 Hz, 2H, H-32,34), 6.46 (s, 1H, NH_2-24), 6.33 – 6.26 (m, 2H, H-4, NH_2-24), 5.99 (d, $J^3_{H\text{-}3,H\text{-}4}$= 11.6 Hz, 1H, H-3), 4.98 – 4.85 (m, 2H, H-5,9), 4.69 – 4.62 (m, 1H, H-7), 4.26 (s, 1H, OH-11), 3.86 (s, 3H, H-36), 3.75 – 3.69 (m, 1H, H-6), 3.45 – 3.39 (m, 1H, H-11), 3.18 (s, 3H, H-27), 3.07 (d, J^2 = 11.2 Hz, 1H, H-15), 2.94 (s, 3H, H-23), 2.89 (s, 1H, H-15), 2.69 – 2.61 (m, 1H, H-12), 2.21 (s, 1H, H-14), 1.95 (s, 3H, H-22), 1.83 – 1.77 (m, 1H, H-10), 1.73 – 1.65 (m, 1H, H-13), 0.70 (d, $J^3_{H\text{-}14,H\text{-}28}$= 6.6 Hz, 3H, H-28), 0.63 (d, $J^3_{H\text{-}10,H\text{-}26}$= 6.3 Hz, 3H, H-26), 0.54 – 0.46 (m, 1H, H-13), 0.30 (s, 3H, H-25).$^{13}C\{1H\}$ NMR (126 MHz, DMSO-d_6+ ACN-d_3) δ 174.2 (C-1), 162.5 (C-33), 162.3 (C-29), 156.5 (C-24), 148.0 (C-21), 144.2 (C-18), 140.9 (C-17), 135.8 (C-2), 133.9 (C-9), 130.0 (C-4), 129.2* (C-8,31,35), 127.4 (C-5), 126.9* (C-20,30), 123.5 (C-3), 120.0 (C-16), 104.8 (C-19), 115.1* (C-32,34), 80.4 (C-7), 79.9 (C-12), 74.5 (C-6), 71.3 (C-11), 55.9 (C-36), 55.8 (C-27), 55.3 (C-23), 34.9 (C-10), 31.6 (C-15), 31.2 (C-13), 30.5 (C-14), 18.9 (C-26), 16.6 (C-28), 14.2 (C-22), 9.9 (C-25). (*-overlapped)

Compound ***12a.*** (4*E*,6*Z*,8*S*,9*S*,10*E*,12*S*,13*R*,14*S*,16*R*)-1^2-(4-(dimethylamino)phenyl)-1^5,13-dihydroxy-8,14-dimethoxy-4,10,12,16-tetramethyl-3-oxo-2-aza-1(6,4)-benzo[*d*]oxazolacycloheptadecaphane-4,6,10-trien-9-yl carbamate (65 mg, yield 65 %), mp 206-208 ºC, HPLC (OD-H, water/acetonitrile = 35/65, flow rate = 1.0 mL/min, l = 260 nm) tR = 3.605 min (major). Anal. Calcd for $C_{37}H_{48}N_4O_8$: C, 65.66; H, 7.15; N, 8.28. Found: C, 65.65; H, 7.16; N, 8.30. HRMS (MALDI-TOF) m/z: $[M + H]^+$ Calcd for $C_{37}H_{48}N_4O_8$ 676.3472; Found 676.3027. FT-IR (KBr): $\nu_{as}(N\text{-}H)_{carbamate}$ =3430.46 cm^{-1}, $\nu_s(N\text{-}H)_{carbamate}$=3347.66 cm^{-1}, $\nu_s(N\text{-}H)_{lactam}$, $\nu(O\text{-}H)_{phenol}$ =3294.99 cm^{-1}, ν(O-H) =3191.67 cm^{-1}, ν_{as}(C-H) =2924.75 cm^{-1}, ν_s(C-H) =2820.93 cm^{-1}, $\nu(C{=}O)_{carbamate}$ =1733.03 cm^{-1}, $\nu(C{=}O)_{lactam}$=1612.69 cm^{-1}, ν(C=N) =1658.70 cm^{-1}, ν(C=C) =1609.40 cm^{-1} , δ(N-H) =1508.98 cm^{-1}, $\delta(N\text{-}H)_{carbamate}$ =1481.13 cm^{-1}, $\nu(C\text{-}N)_{carbamate}$ =1366.32 cm^{-1}, $\nu(C\text{-}O\text{-}C)_{carbamate}$ =1172.24 cm^{-1}, ν(C-O-C) =1104.95 cm^{-1}, $\nu(C\text{-}O\text{-}C)_{methoxy}$ =1060.03 cm^{-1} , $\gamma(=C\text{-}H)_{ar}$=782.63 cm^{-1} , $\gamma(N\text{-}H)_{carbamate}$=740.19 cm^{-1}. ^1H NMR (600 MHz, DMSO-d_6+ ACN-d_3) δ 9.42 (s, 1H, NH-1), 8.41 (s, 1H, OH-21), 7.95 – 7.92 (m, 2H, H-31,35), 7.22 (s, 1H, H-19), 6.84 (d, $J^3_{H\text{-}31,35,H\text{-}32,34}$= 8.8 Hz, 2H, H-32,34), 6.47 (s, 1H, NH_2-24), 6.33 – 6.26 (m, 2H, H-4, NH_2-24), 5.98 (d, $J^3_{H\text{-}3,H\text{-}4}$= 11.5 Hz, 1H, H-3), 4.93 – 4.86 (m, 2H, H-5,9), 4.66 (d, $J^3_{H\text{-}6,H\text{-}7}$= 9.6 Hz, 1H, H-7), 4.25 (s, 1H, OH-11), 3.71 (t, $J^3_{H\text{-}6,H\text{-}7}$= 10.1 Hz, 1H, H-6), 3.44 – 3.41 (m, 1H, H-11), 3.18 (s, 3H, H-27), 3.03 (s, 7H, H-15,36,37), 2.94 (s, 3H, H-23), 2.89 (bs, 1H, H-15), 2.68 – 2.63 (m, 1H, H-12), 2.23 – 2.18 (m, 1H, H-14), 1.95 (s, 3H, H-22), 1.87 – 1.81

(m, 1H, H-10), 1.72 – 1.65 (m, 1H, H-13), 0.70 (d, $J^3_{H-14,H-28}$= 6.6 Hz, 3H, H-28), 0.64 (d, $J^3_{H-10,H-26}$= 6.5 Hz, 3H, H-26), 0.52 (t, J^2 = 13.0 Hz, 1H, H-13), 0.32 (s, 3H, H-25).^{13}C{1H} NMR (126 MHz, DMSO-d_6+ ACN-d_3) δ 174.2 (C-1), 163.5 (C-29), 156.5 (C-24), 152.5 (C-33), 147.8 (C-21), 144.0 (C-18), 141.3 (C-17), 135.8 (C-2), 133.8 (C-9), 130.0 (C-4), 129.3 (C-8), 128.7* (C-31,35), 127.4 (C-5), 126.1 (C-20), 123.4 (C-3), 118.5 (C-16), 114.1 (C-30), 112.1* (C-31,35), 104.6 (C-19), 80.5 (C-7), 80.0 (C-12), 74.5 (C-6), 71.4 (C-11), 55.9 (C-27), 55.3 (C-23), 40.1* (C-36,37), 34.9 (C-10), 31.5 (C-15), 31.1 (C-13), 30.5 (C-14), 19.0 (C-26), 16.7 (C-28), 14.1 (C-22), 9.9 (C-25). (*-overlapped). bs - broad singlet

*Compound **13a.*** (4*E*,6*Z*,8*S*,9*S*,10*E*,12*S*,13*R*,14*S*,16*R*)- 1^5,13-dihydroxy-8,14-dimethoxy-4,10,12,16-tetramethyl-3-oxo-1^2-propyl 2-aza-1(6,4)-benzo[*d*]oxazolacycloheptadecaphane-4,6,10-trien-9-yl carbamate (71 mg, yield 66 %), HPLC (OD-H, water/acetonitrile = 55/45, flow rate = 0.75 mL/min, l = 260 nm) tR = 2.065 min (major). Anal. Calcd for C32H45FN3O8: C, 64.09; H, 7.56; N, 7.01. Found: C, 64.12; H, 7.53; N, 6.99. HRMS (MALDI-TOF) m/z: [M + H]$^+$ Calcd for $C_{32}H_{46}N_3O_8$ 600.3279; Found 600.3284. FT-IR (KBr): ν(N-H)$_{carbamate}$ =3439 cm^{-1}, ν(N-H)$_{lactam}$=3310 cm^{-1}, ν(O-H)$_{phenol}$ =3310 cm^{-1}, ν(O-H) =3192 cm^{-1}, $ν_{as}$(C-H) =2962 cm^{-1}, 2931 cm^{-1}, $ν_s$(C-H) =2875 cm^{-1}, 2825. cm^{-1}, ν(C=O)$_{carbamate}$ =1732 cm^{-1}, ν(C=O)$_{lactam}$=1702 cm^{-1}, ν(C=N) =1653 cm^{-1}, ν(C=C) = 1631 cm^{-1}, 1615 cm^{-1}, δ(N-H) =1532 cm^{-1}, δ(N-H)$_{carbamate}$ =1481 cm^{-1}, ν(C-N)$_{carbamate}$ =1393 cm^{-1}, ν(C-O-C)$_{carbamate}$ =1176 cm^{-1}, ν(C-O-C) =1107 cm^{-1}, ν(C-O-C)$_{methoxy}$ =1060 cm^{-1}, γ(=C-H)$_{ar}$=765 cm^{-1}. ^{1}H NMR (500 MHz, DMSO-*d*6+ACN-*d*3) δ 9.32 (s, 1H, NH-1), 8.34 (s, 1H, OH-21), 7.16 (s, 1H, H-19), 6.45 (s, 1H, NH2-24), 6.30 (t, $J^3_{H-3,H-4}$= 11.4 Hz, 1H, H-4), 6.26 (s, 1H, NH_2-24), 6.01 (d, $J^3_{H-3,H-4}$= 11.2 Hz, 1H, H-3), 4.98 – 4.87 (m, 2H, H-5,9), 4.72 (d, $J^3_{H-6,H-7}$= 9.6 Hz, 1H, H-7), 4.16 (d, 1H, $J^3_{HO,H-11}$= 3.5 Hz, OH-11), 3.76 (t, $J^3_{H-6,H-7}$= 10.1 Hz, 1H, H-6), 3.45 (m, 1H, H-11), 3.18 (s, 3H, H-27), 3.02 (dd, J^2= 13.3 Hz, $J^3_{H-15,H-14}$= 5.2 Hz, 1H, H-15), 2.95 (s, 3H, H-23), 2.91-2,76* (m, 3H, H-15, H-30), 2.68 – 2.63 (m, 1H, H-12), 2.19 – 2.02 (m, 1H, H-14), 1.95 (s, 3H, H-22), 1.87 – 1.82 (m, 1H, H-10), 1.82 – 1.74 (m, 2H, H-31), 1.56 – 1.49 (m, 1H, H-13), 1.04 (t, $J^3_{H-31,H-32}$= 7.4 Hz, 2H, H-32), 0.80 (d, $J^3_{H-10,H-26}$= 6.3 Hz, 3H, H-26), 0.70 (d, $J^3_{H-14,H-28}$= 7.0 Hz, 3H, H-28), 0.46 – 0.40 (m, 1H, H-13), 0.39 (s, 3H, H-25). ^{13}C{^{1}H} NMR (126 MHz, DMSO-d_6+ ACN-d_3) δ 174.2 (C-1), 166.5 (C-29), 156.6 (C-24), 147.3 (C-21), 144.1 (C-18), 139.9 (C-17), 135.8 (C-2), 134.0 (C-9), 129.8 (C-4), 129.3 (C-8), 127.6 (C-5), 126.7 (C-20), 123.2 (C-3), 118.8 (C-16), 104.2 (C-19), 80.6 (C-7), 79.9 (C-12), 74.7 (C-6), 71.4 (C-11), 55.8 (C-27), 55.3 (C-23), 34.9 (C-10), 31.5 (C-15), 30.8 (C-13), 30.5 (C-14), 30.2 (C-30), 21.0 (C-31), 18.8 (C-26), 16.8 (C-28), 13.9* (C-22, C-32), 10.1 (C-25). (*-overlapped)

*Compound **14a.*** (4*E*,6*Z*,8*S*,9*S*,10*E*,12*S*,13*R*,14*S*,16*R*)-1^5,13-dihydroxy-8,14-dimethoxy-4,10,12,16-tetramethyl-3-oxo-1^2-vinyl-2-aza-1(6,4)-benzo[*d*]oxazolacycloheptadecaphane-4,6,10-trien-9-yl carbamate (73 mg, yield 70 %), HPLC (OD-H, water/acetonitrile = 55/45, flow rate = 0.75 mL/min, l = 260 nm) tR = 1.672 min (major). Anal. Calcd for $C_{31}H_{41}N_3O_8$: C, 63.79; H, 7.08; N, 7.20; Found: C, 63.84; H, 7.04; N, 7.22. HRMS (MALDI-TOF) m/z: [M + H]$^+$ Calcd for $C_{31}H_{42}N_3O_8$ 584.2966; Found 584.2970. FT-IR (KBr): $ν_{as}$(N-H)$_{carbamate}$ =3446 cm^{-1}, $ν_s$(N-H)$_{carbamate}$ =3394 cm^{-1}, ν(N-H)$_{lactam}$=3335 cm^{-1}, ν(O-H)$_{phenol}$ =3295 cm^{-1}, ν(O-H) =3181 cm^{-1}, $ν_{as}$(C-H) =2962 cm^{-1}, 2927 cm^{-1}, $ν_s$(C-H) =2823. cm^{-1}, ν(C=O)$_{carbamate}$ =1731 cm^{-1}, ν(C=O)$_{lactam}$=1700 cm^{-1}, ν(C=N) =1647 cm^{-1}, ν(C=C) = 1647 cm^{-1}, 1613 cm^{-1}, δ(N-H) =1532 cm^{-1}, δ(N-H)$_{carbamate}$ =1480 cm^{-1}, ν(C-N)$_{carbamate}$ =1378 cm^{-1}, ν(C-O-C)$_{carbamate}$ =1178 cm^{-1}, ν(C-O-C) =1113 cm^{-1}, ν(C-O-C)$_{methoxy}$ =1062 cm^{-1}, γ(=C-H) =879 cm^{-1}, 783 cm^{-1}, γ(=C-H)$_{ar}$=766 cm^{-1}. ^{1}H NMR (500 MHz, DMSO-*d*6+ACN-*d*3) δ 9.42 (s, 1H, NH-1), 8.48 (s, 1H, OH-21), 7.23 (s, 1H, H-19), 6.81 (dd, $J^3_{H-30,H-31-trans}$= 17.5 Hz, $J^3_{H-30,H-31-cis}$= 11.1 Hz, 1H, H-30), 6.46 (s, 1H, NH2-24), 6.32 (d, $J^3_{H-30,H-31-trans}$= 17.5 Hz, 1H, H-31$_{trans}$), 6.30 (t, $J^3_{H-3,H-4}$= 11.5 Hz, 1H, H-4), 6.27 (s, 1H, NH_2-24), 6.01 (d, $J^3_{H-3,H-4}$= 11.6 Hz, 1H, H-3), 5.87 (d, $J^3_{H-30,H-31-cis}$= 11.4 Hz, 1H, H-31$_{trans}$), 4.97 – 4.90 (m, 2H, H-5,9), 4.71 (d, $J^3_{H-6,H-7}$= 9.6 Hz, 1H, H-7), 4.22 (s, 1H, OH-11), 3.74 (t, $J^3_{H-6,H-7}$= 10.1 Hz, 1H, H-6), 3.45-3.41 (m, 1H, H-11), 3.27-3.19 (m, 1H, H-15), 3.18 (s, 3H, H-27), 3.07-3.01 (m, 1H, H-15), 2.96 (s, 3H, H-23), 2.87-2.84 (m, 1H, H-15), 2.68-2.62 (m, 1H, H-12), 2.25 – 2.12 (m, 1H, H-14), 1.95 (s, 3H, H-22), 1.83-1.76 (m, 1H, H-10), 1.57 – 1.50 (m, 1H, H-13), 0.78 (d, $J^3_{H-10,H-26}$= 6.7 Hz, 3H, H-26), 0.71 (d, $J^3_{H-14,H-28}$= 6.9 Hz, 3H, H-28), 0.48 – 0.40 (m, 1H, H-13), 0.36 (s, 3H, H-25). ^{13}C{^{1}H} NMR (126 MHz, DMSO-d_6+ ACN-d_3) δ 174.1 (C-1), 161.5(C-29), 156.5 (C-24), 147.9 (C-21), 143.7(C-18), 140.4 (C-17), 135.7 (C-2), 133.9 (C-9), 129.8 (C-4), 129.1 (C-8), 128.0 (C-5), 127.6 (C-20), 125.0 (C-30),124.2 (C-31), 123.3 (C-3), 119.3 (C-16), 104.5 (C-19), 80.6 (C-7), 79.9 (C-12), 74.5 (C-6), 71.4 (C-11), 55.8 (C-27), 55.3 (C-23), 34.8 (C-10), 31.4 (C-15), 30.8 (C-13), 30.5 (C-14), 29.7 (C-30), 19.0 (C-26), 16.7 (C-28), 13.9 (C-22), 9.8 (C-25). (*-overlapped)

*Compound **15a.*** (4*E*,6*Z*,8*S*,9*S*,10*E*,12*S*,13*R*,14*S*,16*R*)-1^2-ethynyl-1^5,13-dihydroxy-8,14-dimethoxy-4,10,12,16-tetramethyl-3-oxo-2-aza-1(6,4)-benzo[d]oxazolacycloheptadecaphane-4,6,10-trien-9-yl carbamate (70 mg, yield

67 %) mp 249-254 ºC, HPLC (OD-H, water/acetonitrile = 55/45, flow rate = 0.75 mL/min, l = 260 nm) tR = 1.985 min (major). Anal. Calcd for $C_{31}H_{39}N_3O_8$: C, 64.01; H, 6.76; N, 7.22; Found: C, 63.98; H, 6.78; N, 7.20. HRMS (MALDI-TOF) m/z: $[M + H]^+$ Calcd for $C_{31}H_{40}N_3O_8$ 582.2810; Found 582.2814. FT-IR (KBr): ν_{as}(N-H)$_{carbamate}$ =3443 cm^{-1}, ν_s(N-H)$_{carbamate}$ =3398 cm^{-1}, ν(N-H)$_{lactam}$ =3315 cm^{-1}, ν(O-H)$_{phenol}$ =3301 cm^{-1}, ν(O-H) =3199 cm^{-1}, ν_{as}(C-H) =2960 cm^{-1}, 2927 cm^{-1}, ν_s(C-H) =2824. cm^{-1}, ν(C≡C) =2122 cm^{-1}, ν(C=O)$_{carbamate}$ =1731 cm^{-1}, ν(C=O)$_{lactam}$=1700 cm^{-1}, ν(C=N) =1657 cm^{-1}, ν(C=C) = 1649 cm^{-1}, 1621 cm^{-1}, δ(N-H) =1527 cm^{-1}, δ(N-H)$_{carbamate}$ =1482 cm^{-1}, ν(C-N)$_{carbamate}$ =1376 cm^{-1}, ν(C-O-C)$_{carbamate}$ =1190 cm^{-1}, ν(C-O-C) =1113 cm^{-1}, ν(C-O-C)$_{methoxy}$ =1059 cm^{-1}, γ(=C-H) =877 cm^{-1}, 783 cm^{-1}, γ(H-C≡C) =632 cm^{-1}. ^{1}H NMR (500 MHz, DMSO-*d*6+ACN-*d*3) δ 9.52 (s, 1H, NH-1), 8.68 (s, 1H, OH-21), 7.26 (s, 1H, H-19), 6.45 (s, 1H, NH2-24), 6.31-6.27 (m, 2H, H-4, NH_2-24), 6.00 (d, $J^3_{H-3,H-4}$= 11.4 Hz, 1H, H-3), 4.99 – 4.88 (m, 2H, H-5,9), 4.87 (s, 1H, H-31), 4.72 (d, $J^3_{H-6,H-7}$= 9.9 Hz, 1H, H-7), 4.30 (d, $J^3_{H-11,OH}$= 3.1 Hz, 1H, OH-11), 3.74 (t, $J^3_{H-6,H-7}$= 10.3 Hz, 1H, H-6), 3.45-3.41 (m, 1H, H-11), 3.30-3.22 (m, 1H, H-15), 3.17 (s, 3H, H-27), 3.07-3.01 (dd, J^2= 14.1 Hz, $J^3_{H-15,H-14}$= 5.0 Hz, 1H, H-15), 2.96 (s, 3H, H-23), 2.88-2.81 (m, 1H, H-15), 2.64-2.60 (m, 1H, H-12), 2.21 – 2.11 (m, 1H, H-14), 1.94 (s, 3H, H-22), 1.86-1.77 (m, 1H, H-10), 1.59 – 1.49 (m, 1H, H-13), 0.78 (d, $J^3_{H-10,H-26}$= 6.7 Hz, 3H, H-26), 0.83 (d, $J^3_{H-14,H-28}$= 6.4 Hz, 3H, H-28), 0.42 – 0.36 (m, 1H, H-13), 0.35 (s, 3H, H-25). ^{13}C{^{1}H} NMR (126 MHz, DMSO-d_6+ ACN-d_3) δ 174.0 (C-1), 156.5 (C-24), 148.6 (C-21), 145.6 (C-29), 143.7 (C-18), 138.9 (C-17), 135.8 (C-2), 133.9 (C-9), 129.8 (C-4), 129.6 (C-8), 129.0 (C-5), 127.7 (C-20), 123.6 (C-3), 119.3 (C-16), 104.5 (C-19), 85.5 (C-31), 80.5 (C-7), 79.7 (C-12), 74.4 (C-6), 71.9 (C-11), 71.2 (C-30), 55.8 (C-27), 55.3 (C-23), 34.9 (C-10), 31.3 (C-15), 30.9 (C-13), 30.5 (C-14), 30.5 (C-30), 19.0 (C-26), 16.4 (C-28), 13.9 (C-22), 9.7 (C-25). (*-overlapped)

Compound ***16a.*** (4*E*,6*Z*,8*S*,9*S*,10*E*,12*S*,13*R*,14*S*,16*R*)-15,13-dihydroxy-8,14-dimethoxy-4,10,12,16-tetramethyl-3-oxo-12-(pyridine-4-yl)-2-aza-1(6,4)-benzo[d]oxazolacycloheptadecaphane-4,6,10-trien-9-yl carbamate (76 mg, yield 67 %), HPLC (OD-H, water/acetonitrile = 55/45, flow rate = 0.75 mL/min, l = 260 nm) tR = 1.812 min (major). Anal. Calcd for $C_{34}H_{43}N_4O_8$: C, 64.34; H, 6.67; N, 8.83; Found: C, 64.37; H, 6.63; N, 8.82. HRMS (MALDI-TOF) m/z: $[M + H]^+$ Calcd for $C_{34}H_{43}N_4O_8$ 635.3075; Found 635.3071. FT-IR (KBr): ν_{as}(N-H)$_{carbamate}$ =3428 cm^{-1}, ν_s(N-H)$_{carbamate}$ =3378 cm^{-1}, ν(N-H)$_{lactam}$ =3315 cm^{-1}, ν(O-H)$_{phenol}$ =3003 cm^{-1}, ν(O-H) =3209 cm^{-1}, ν(C-H)$_{pyridine}$ =3114 cm^{-1}, ν(C-H)$_{pyridine}$ =3044cm^{-1}, ν_s(C-H) = 2900 cm^{-1}, ν_s(C-H) =2874. cm^{-1}, 2824. cm^{-1}, ν(C=O)$_{carbamate}$ =1731 cm^{-1}, ν(C=O)$_{lactam}$=1701 cm^{-1}, ν(C=N)+ ν(C=C)$_{pyridine}$ =1643 cm^{-1}, ν(C=C) =1630 cm^{-1}, δ(N-H) =1531 cm^{-1}, δ(N-H)$_{carbamate}$ =1484 cm^{-1}, ν(C-N)$_{carbamate}$ =1381 cm^{-1}, ν(C-O-C)$_{carbamate}$ =1195 cm^{-1}, ν(C-O-C) =1103 cm^{-1}, ν(C-O-C)$_{methoxy}$ =1056 cm^{-1}, γ(=C-H)$_{ar}$=765 cm^{-1}. ^{1}H NMR (500 MHz, DMSO-*d*6+ACN-*d*3) δ 9.53 (s, 1H, NH-1), 8.82 (d, $J^3_{H-31,H-32\ (H-33,H-34)}$= 5.9 Hz, 2H, H-32,33), 8.68 (s, 1H, OH-21), 8.02 (d, $J^3_{H-31,H-32\ (H-33,H-34)}$= 5.9 Hz, 2H, H-31,34), 7.36 (s, 1H, H-19), 6.39 (s, 1H, NH2-24), 6.30 (t, $J^3_{H-3,H-4}$= 11.4 Hz, 1H, H-4), 6.23 (s, 1H, NH_2-24), 6.02 (d, $J^3_{H-3,H-4}$= 12.7 Hz, 1H, H-3), 5.04 – 4.83 (m, 2H, H-5,9), 4.67 (d, $J^3_{H-6,H-7}$= 9.7 Hz, 1H, H-7), 4.23 (s, 1H, OH-11), 3.73 (t, $J^3_{H-6,H-7}$= 10.3 Hz, 1H, H-6), 3.25 (m, 1H, H-11), 3.19 (s, 3H, H-27), 3.15-3.08 (m, 1H, H-15), 2.95 (s, 3H, H-23), 2.94-2,72 (m, 1H, H-15), 2.70 – 2.62 (m, 1H, H-12), 2.30 – 2.19 (m, 1H, H-14), 1.96 (s, 3H, H-22), 1.76 – 1.65 (m, 2H, H-10,13), 0.73 (d, $J^3_{H-10,H-26}$= 6.7 Hz, 3H, H-26), 0.61 (d, $J^3_{H-14,H-28}$= 6.7 Hz, 3H, H-28), 0.50 – 0.41 (m, 1H, H-13), 0.29 (s, 3H, H-25). ^{13}C{^{1}H} NMR (126 MHz, DMSO-d_6+ ACN-d_3) δ 174.0 (C-1), 160.0 (C-29), 156.4 (C-24), 151.3 (C-32,33), 148.3 (C-21), 144.5 (C-18), 140.4 (C-17), 135.8 (C-2), 134.4 (C-30), 133.8 (C-9), 129.8 (C-4), 128.9 (C-8), 127.7 (C-5), 123.5 (C-20), 123.3 (C-3), 120.7 (C-31,34), 119.5 (C-16), 105.0 (C-19), 80.4 (C-7), 79.8 (C-12), 74.5 (C-6), 71.4 (C-11), 55.9 (C-27), 55.3 (C-23), 34.9 (C-10), 31.5 (C-15), 31.2 (C-13), 30.4 (C-14), 29.8 (C-30), 18.6 (C-26), 16.7 (C-28), 13.9 (C-22), 9.7 (C-25). (*-overlapped)

Table S1 Cyclization reaction tests, presented for the amine derivative of **GDM**, which in position C(17) contains an aminobenzyl substituent, leading to the formation of an oxazole ring using various organic and inorganic bases as catalysts, and carried out in various solvents.

	time of reaction (h) / yield of reaction (%) / conversion of substrate (%)				
	DMF	THF	ACN	Acetone	DMSO
NaH	0.5 / 40 / 62<br>1.5 / 51 / 72<br>24 /64 / 90	---	---	---	---
K_2CO_3	0.5 / 35 / 59<br>1 / 60 / 83<br>1.5 / 74 / >99	1 / 1 / 7<br>4 / 2 / 8<br>24 / 10 / 29	0.5 / 1 / 5<br>1 / 2 / 6<br>24 / 21 / 29	1 / 10 / 17<br>4 / 33 / 42<br>24 / 70 / 85	0.5 / 58 / 99<br>1 / 62 / >99
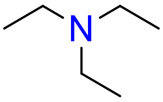 TEA	22 / 21 / 27<br>96 / 43 / 50	---	---	---	---
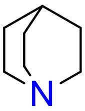 Quinuclidine	0.5 / 3 / 95<br>1 / 5 / 92<br>2 / 8 / 90<br>19 / 25 / 70<br>24 / 28 / 53	---	---	---	---
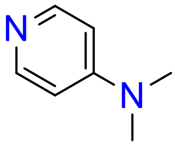 DMAP	22 / 11 / 19<br>96 / 27 / 36	---	---	---	---
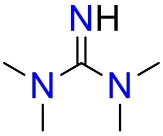 TMG	0.5 / 70 / >99<br>1 / 71 / >99	1 / 29 / 38<br>5 / 64 / 75	1 / 46 / 58<br>2 / 67 / 81	0.5 / 56 / 71<br>1 / 70 / 86<br>1.5 / 89 / 97	0.5 / 68 / 99<br>1 / 68 / >99
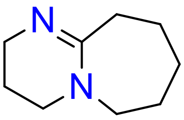 DBU	0.5 / 64 / 83<br>1 / 70 / 95	---	---	---	---
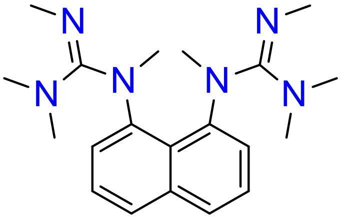 TMGN	0.5 / 55 / 89<br>1 / 65 / 98	1 / 4 / 19<br>2 / 4 / 19	---	0.5 / 54 / 72<br>1 / 67 / 82<br>2 / 76 / 92	0.5 / 51 / 81<br>1 / 63 / 94<br>1.5 / 67 / 98
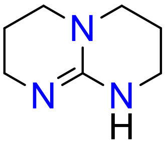	1.5 / 75 / 88	---	---	0.5 / 55 / 74<br>1 / 57 / 76<br>2 / 60 / 78	0.5 / 82 / 94<br>1 / 87 / >99

TBD					
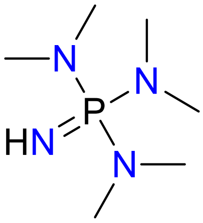<br>P_1-H	0.5 / 80 / >99<br>1 / 80 / >99	---	---	---	---

Table S2 Cyclization reaction tests, presented for the amine derivatives (**1-12**) of **GDM**, leading to the formation of an oxazole ring using TMG as catalysts, and carried out in DMF (0°C).

	Conversion of substrate [%]/ yield of product [%]						
	10 min	20 min	30 min	40 min	50 min	60 min	80 min
**1**	50/43	68/58	77/69	83/72	88/79	91/81	94/88
**2**	51/36	69/52	81/67	87/76	90/80	92/84	96/87
**3**	74/63	88/73	95/83	97/86	98/87	99/89	>99/90
**4**	73/37	87/58	93/63	96/76	98/74	98/79	99/75
**6**	72/57	90/62	96/81	98/76	99/81	99/82	100/90
**7**	63/49	85/71	93/81	96/83	98/86	99/87	100/91
**8**	91/77	98/83	99/83	99/84	99/85	99/85	99/85
**9**	95/80	99/86	99/87	99/87	99/87	99/87	99/87
**10**	35/27	56/41	69/56	78/62	83/72	87/74	91/80
**11**	46/39	59/51	72/60	79/70	85/75	89/74	93/80
**12**	39/32	54/44	61/50	70/58	82/69	88/73	95/78

Table S3 Crystal data and refinement details

Compound	**1a**	**2a**	**10a**	**3a**	**4a**	**5a**
empirical formula	$C_{35}H_{43}N_3O_8\cdot(C_3H_8O)_2$	$C_{35}H_{42}FN_3O_8\cdot(C_3H_8O)_2$	$C_{36}H_{45}N_3O_8\cdot C_3H_8O$	$C_{35}H_{42}ClN_3O_8\cdot C_3H_8O$	$C_{35}H_{42}BrN_3O_8\cdot C_3H_8O$	$C_{35}H_{42}IN_3O_8$
CCDC no.	2155685	2155686	2155693	2155687	2155688	2245620
formula weight	753.91	771.90	707.84	728.26	772.72	759.42
crystal system	Monoclinic	Monoclinic	Monoclinic	Monoclinic	Monoclinic	Monoclinic
space group	$P2_1$	$P2_1$	$P2_1$	$P2_1$	$P2_1$	$P2_1$
a (Å)	14.2947(3)	14.2162(2)	15.3464(4)	15.3943(4)	15.3999(3)	15.3830(14)
b (Å)	7.65370(15)	7.63320(12)	7.6850(2)	7.68493(17)	7.69141(10)	7.5875(11)
c (Å)	18.5012(3)	18.6354(3)	16.6105(5)	16.6460(4)	16.6762(3)	16.7894(14)
β (°)	90.5720(17)	90.7489(14)	108.399(3)	108.905(3)	108.5909(17)	108.429(10)
volume ($Å^3$)	2024.05(6)	2022.04(5)	1858.85(9)	1863.07(8)	1872.18(5)	1859.1(4)
Z	2	2	2	2	2	2
temperature (K)	130	130	130	130	130	130
radiation type	Cu *K*α	Cu *K*α	Cu *K*α	Cu *K*α	Cu *K*α	Cu *K*α
μ (mm^{-1})	0.720	0.770	0.731	1.390	1.977	7.189
refl. collected, unique	23906, 6666	30320, 8211	9878, 5633	17085, 6185	16673, 6169	8241, 4770
restrains/parameters	1/517	1/534	890/539	878/550	877/539	88/501
GOF on F^2	1.053	1.039	1.063	1.053	1.042	1.010
R_1 [I>2σ(I)]	0.0412	0.0315	0.0593	0.0451	0.0677	0.0837
wR_2 [I>2 σ(I)]	0.0832	0.0804	0.1604	0.1324	0.1955	0.2053
R_1 (all data)	0.0564	0.0339	0.0662	0.0483	0.0702	0.1340
wR_2 (all data)	0.0919	0.0822	0.1680	0.1358	0.2042	0.2683
largest pick and hole (e $Å^{-3}$)	0.17, -0.17	0.16, -0.14	0.23, -0.54	0.29, -0.58	0.74, -0.98	0.41/-0.69

Compound	**7a**	**11a**	**6a**	**9a**	**8a**
empirical formula	$C_{36}H_{42}F_3N_3O_9$	$C_{36}H_{45}N_3O_9\cdot(C_3H_8O)_{0.85}$	$C_{36}H_{42}F_3N_3O_8\cdot(C_3H_8O)_{0.46}$	$C_{35}H_{42}N_4O_{10}\cdot(C_3H_8O)_{0.70}$	$C_{36}H_{42}N_4O_8\cdot C_3H_8O$
CCDC no.	2155690	2155694	2155689	2155692	2155691
formula weight	717.50	713.74	729.29	720.79	718.83
crystal system	Monoclinic	Monoclinic	Monoclinic	Monoclinic	Monoclinic
space group	$P2_1$	$P2_1$	$P2_1$	$P2_1$	$P2_1$
a (Å)	15.3405(3)	15.3941(4)	15.4812(6)	15.4049(6)	15.2943(4)
b (Å)	7.68484(14)	7.6808(2)	7.6792(3)	7.6636(4)	7.70332(19)
c (Å)	16.9699(4)	17.1009(5)	16.8804(5)	17.0079(9)	16.8756(5)
β (°)	109.018(2)	109.763(3)	109.424(4)	109.189(5)	108.751(3)
volume (Å^3)	1891.37(7)	1902.90(10)	1892.57(12)	1896.34(17)	1882.72(10)
Z	2	2	2	2	2
temperature (K)	130	130	130	130	130
radiation type	Cu *K*α	Cu *K*α	Cu *K*α	Cu *K*α	Cu *K*α
μ (mm^{-1})	0.847	0.737	0.843	0.771	0.740
refl. collected, unique	17243, 6470	17925, 6498	18512, 6535	10963, 5965	17744, 6021
restrains/parameters	1061/665	399/446	1131/598	897/547	14/504
GOF on F^2	1.040	1.037	1.036	1.038	1.065
R_1 [I>2σ (I)]	0.0551	0.0558	0.0763	0.0798	0.0396
wR_2 [I>2σ (I)]	0.1571	0.1472	0.2054	0.2083	0.1096
R_1 (all data)	0.0589	0.0623	0.0937	0.1044	0.0419
wR_2 (all data)	0.1641	0.1537	0.2225	0.2271	0.1129
largest pick and hole (e Å^{-3})	0.36, -0.23	0.55, -0.50	0.85, -0.42	0.43, -0.35	0.25, -0.30

Table S4 Comparison of biological activity between **GDM**, benzoxazole (**1a-12a**) and C(17)-amine derivatives (**1-12**) in cancer cell lines (SKBR-3, SKOV-3, PC-3) and in healthy cell line (HDF); the activity expressed as IC_{50} [μM]; selectivity indices (SI) are given in square brackets.

	**SKBR-3**	**SKOV-3**	**PC-3**	**HDF**	**ilogP***
**GDM**	0.58 ±0.05<br>[1.81]	0.64 ±0.11<br>[1.64]	0.60 ±0.03<br>[1.75]	1.05 ±0.08	3.04
**1**	1.84 ±0.11<br>[0.82]	1.90 ±0.09<br>[0.79]	1.37 ±0.02<br>[1.1]	1.51 ±0.06	3.86
**2**	0.92 ±0.01<br>[3.05]	0.84 ±0.01<br>[3.35]	0.95 ±0.05<br>[2.96]	2.81 ±0.44	4.25
**3**	0.96 ±0.08<br>[1.42]	0.96 ±0.03<br>[1.42]	0.87 ±0.01<br>[1.56]	1.36 ±0.02	4.33
**4**	2.16 ±0.12<br>[1.33]	2.02 ±0.07<br>[1.43]	1.93 ±0.01<br>[1.49]	2.88 ±0.14	4.36
**5**	1.06 ±0.33<br>[1.42]	1.04 ±0.03<br>[1.45]	1.11 ±0.01<br>[1.36]	1.51 ±0.12	4.33
**6**	1.07 ±0.41<br>[2.44]	1.03 ±0.05<br>[2.53]	1.04 ±0.02<br>[2.51]	2.61 ±0.08	4.30
**7**	0.71 ±0.02<br>[1.52]	0.79 ±0.03<br>[1.37]	0.71 ±0.01<br>[1.52]	1.08 ±0.07	4.34
**8**	1.49 ±0.05<br>[1.70]	1.51 ±0.04<br>[1.68]	1.63 ±0.03<br>[1.55]	2.53 ±0.13	4.05
**9**	1.38 ±0.06<br>[1.28]	1.44 ±0.04<br>[1.23]	1.27 ±0.09<br>[1.39]	1.77 ±0.04	3.51
**10**	1.66 ±0.04<br>[1.12]	1.32 ±0.05<br>[1.41]	1.16 ±0.02<br>[1.6]	1.86 ±0.11	4.10
**11**	1.88 ±0.02<br>[1.43]	1.91 ±0.08<br>[1.40]	1.96 ±0.02<br>[1.37]	2.68 ±0.28	4.36
**12**	0.93 ±0.01<br>[1.71]	0.91 ±0.02<br>[1.75]	0.88 ±0.05<br>[1.81]	1.59 ±0.02	4.15
**1a**	1.03 ±0.03<br>[1.69]	1.16 ±0.10<br>[1.50]	1.09 ±0.01<br>[1.60]	1.74 ±0.22	4.35
**2a**	1.09 ±0.05<br>[1.37]	1.08 ±0.05<br>[1.38]	1.01 ±0.03<br>[1.48]	1.49 ±0.02	4.12
**3a**	0.81 ±0.01<br>[2.05]	0.84 ±0.08<br>[1.98]	0.79 ±0.02<br>[2.10]	1.66 ±0.01	4.20
**4a**	0.94 ±0.01<br>[2.17]	0.92 ±0.02<br>[2.22]	0.97 ±0.01<br>[2.10]	2.04 ±0.06	4.20
**5a**	2.33 ±0.16<br>[1.28]	2.04 ±0.04<br>[1.47]	2.39 ±0.05<br>[1.25]	2.99 ±0.09	4.19
**6a**	0.85 ±0.01<br>[1.73]	0.82 ±0.03<br>[1.79]	0.87 ±0.03<br>[1.69]	1.47 ±0.06	3.97

**7a**	1.62 ±0.02 [1.27]	1.53 ±0.07 [1.35]	1.53 ±0.04 [1.35]	2.06 ±0.12	4.20
**8a**	1.93 ±0.06 [1.64]	1.88 ±0.05 [1.69]	1.84 ±0.03 [1.72]	3.17 ±0.03	3.89
**9a**	0.99 ±0.01 [1.08]	0.96 ±0.05 [1.11]	0.85 ±0.02 [1.26]	1.07 ±0.01	3.45
**10a**	1.33 ±0.04 [1.46]	1.05 ±0.01 [1.85]	1.18 ±0.01 [1.64]	1.94 ±0.11	4.01
**11a**	1.38 ±0.02 [1.58]	1.32 ±0.07 [1.65]	1.39 ±0.01 [1.57]	2.18 ±0.01	4.17
**12a**	0.81 ±0.05 [2.38]	0.85 ±0.01 [2.27]	0.84 ±0.04 [2.30]	1.93 ±0.03	4.32

SKBR-3 human breast cancer cell line
SKOV-3 human ovarian cancer cell line
PC-3 human prostate cancer cell line
HDF human dermal fibroblasts

*** - ilogP** calculated by **SwissADME** tool (http://www.swissadme.ch/)[5,6]

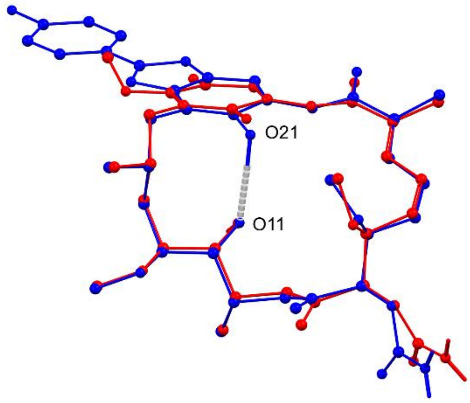

Figure S1 Overlay of the geldanamycin (CCDC 1988068)[7] (red) and **2a** (blue) molecules (rms = 0.233 Å) in conformations found in their crystal structures. Intramolecular O-H···O hydrogen bond in **2a** is shown with a dashed line. Only N-H and O-H hydrogen atoms are shown.

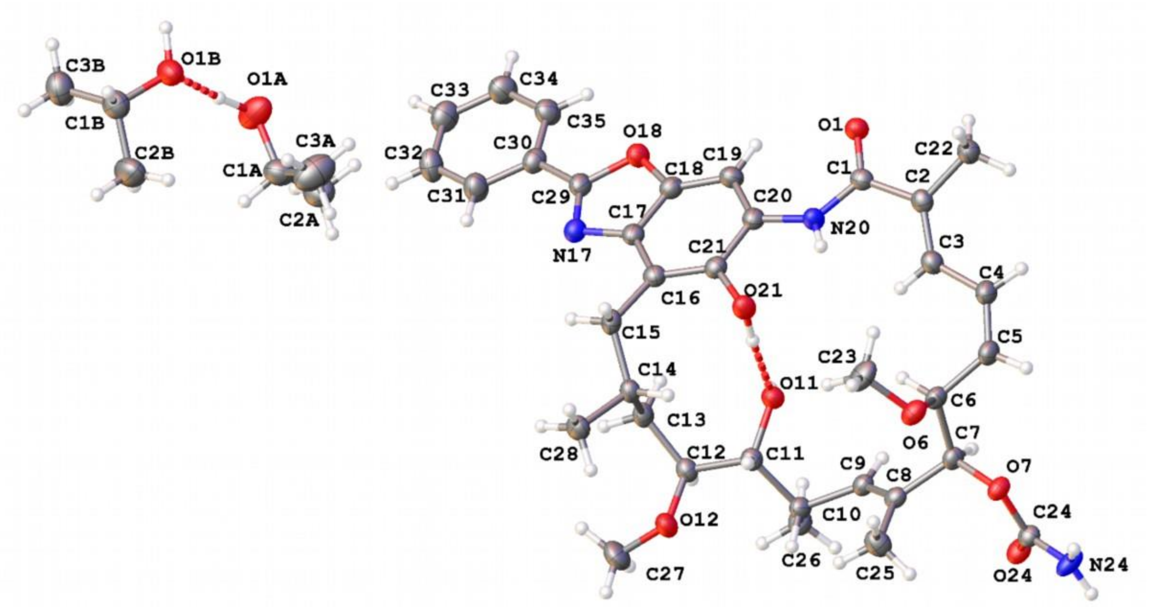

Figure S2 The asymmetric unit of **1a**. Displacement ellipsoids are shown at the 50% probability level.

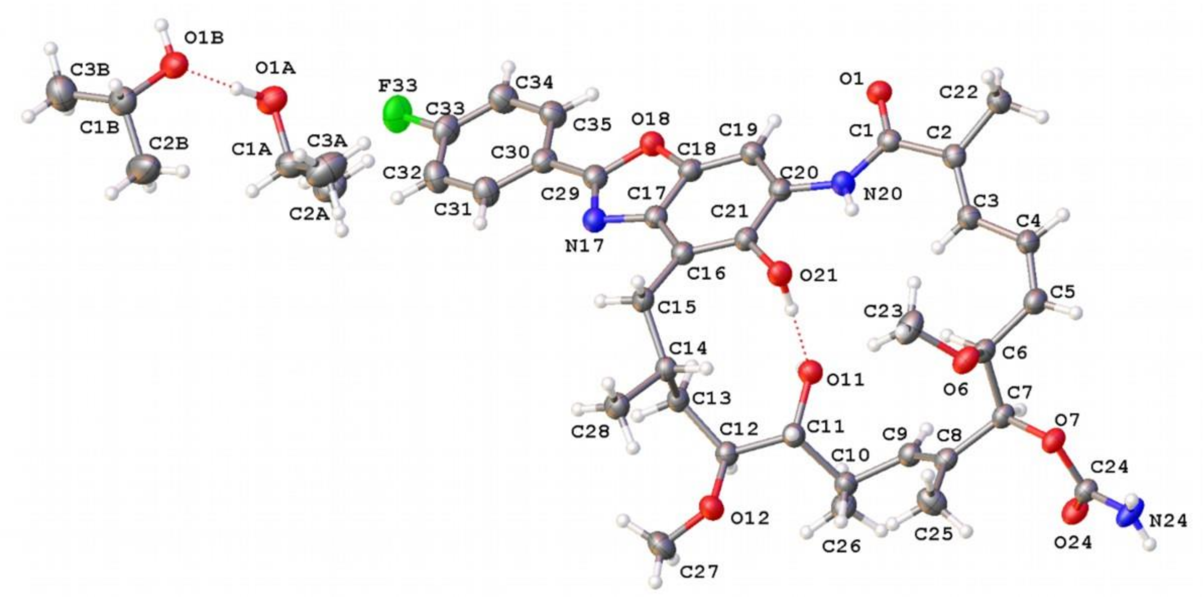

Figure S3 The asymmetric unit of **2a**. Displacement ellipsoids are shown at the 50% probability level.

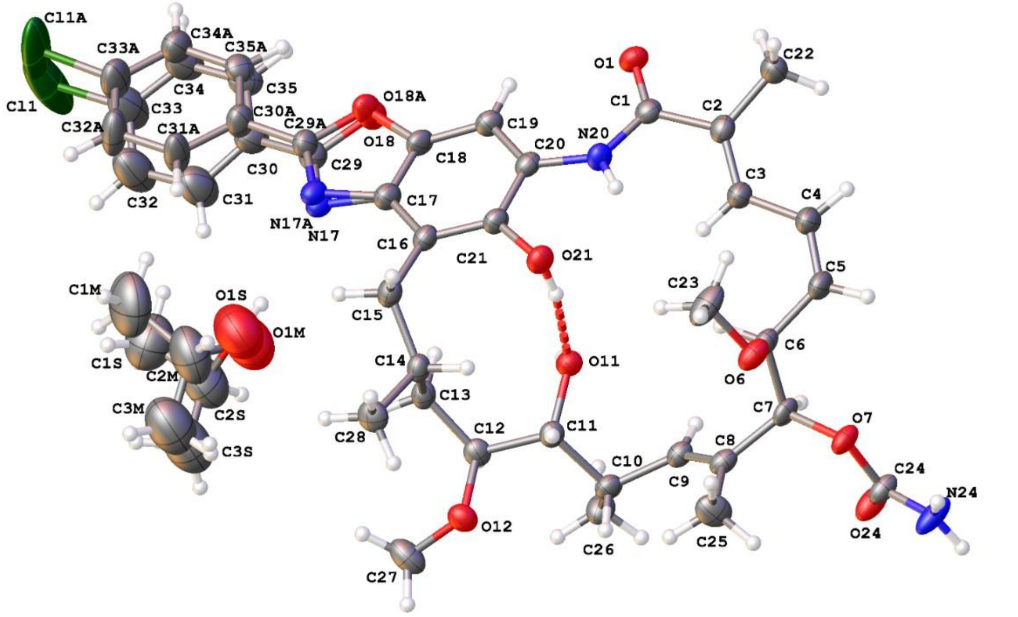

Figure S4 The asymmetric unit of **3a**. Displacement ellipsoids are shown at the 50% probability level. The isopropanol molecule and the substituent at C(29) are disordered.

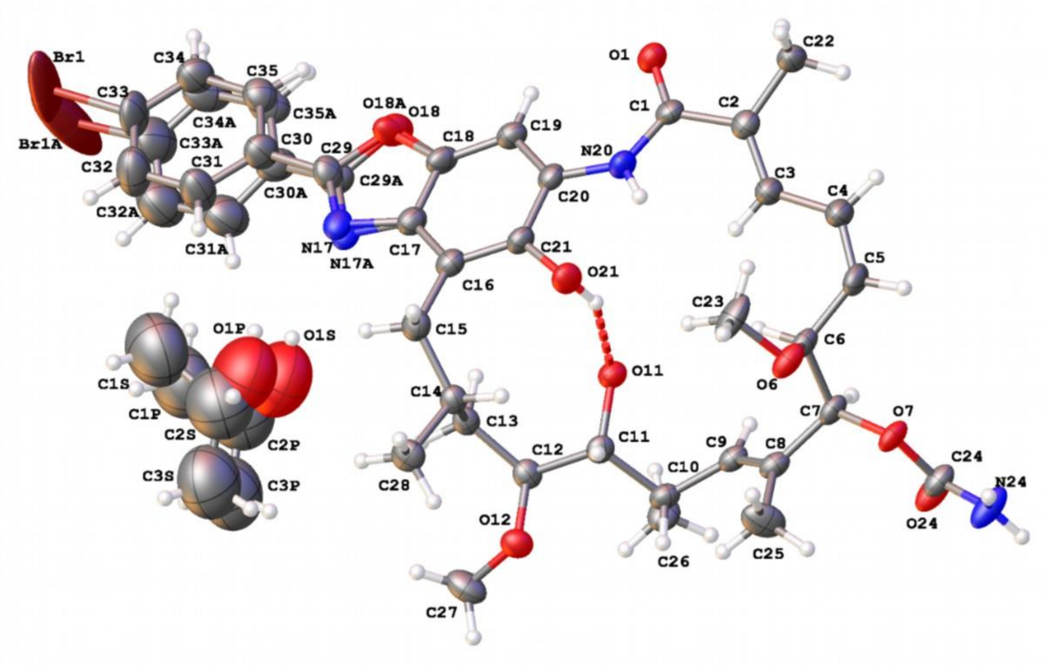

Figure S5 The asymmetric unit of **4a**. Displacement ellipsoids are shown at the 50% probability level. The isopropanol molecule and the substituent at C(29) are disordered.

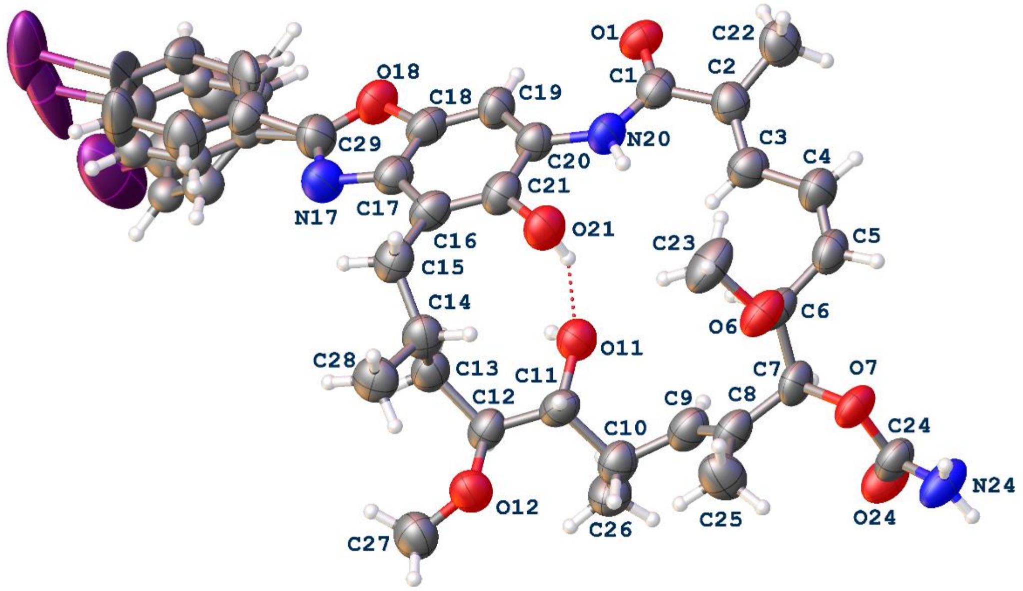

Figure S6 The asymmetric unit of **5a**. Displacement ellipsoids are shown at the 50% probability level. The isopropanol molecule and the substituent at C(29) are disordered.

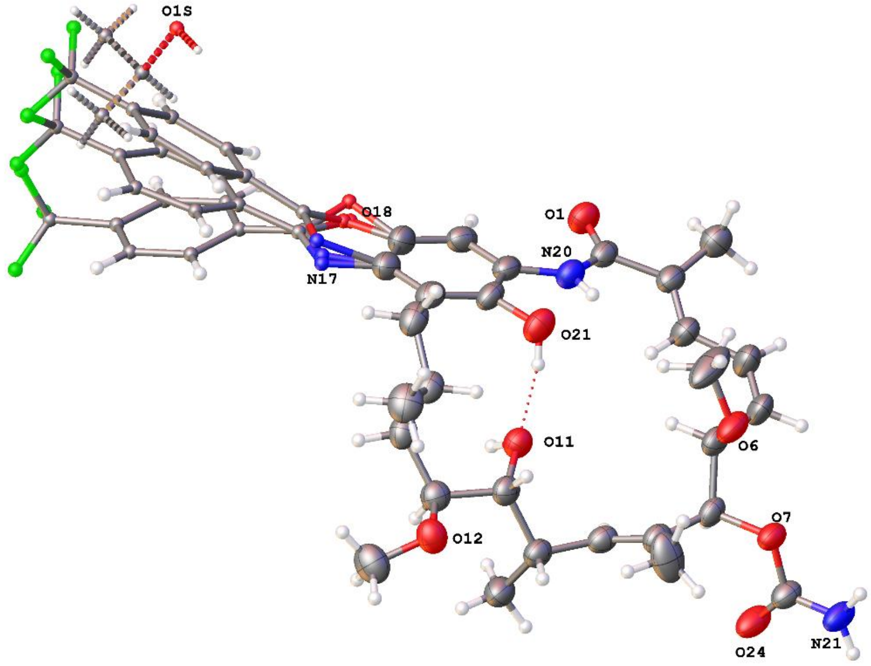

Figure S7 The asymmetric unit of **6a**. Displacement ellipsoids for the ordered part of the molecule are shown at the 50% probability level. The substituent at C(29) shown as a 'ball and stick' model is disordered over three sites. The isopropanol is shown with dashed bonds.

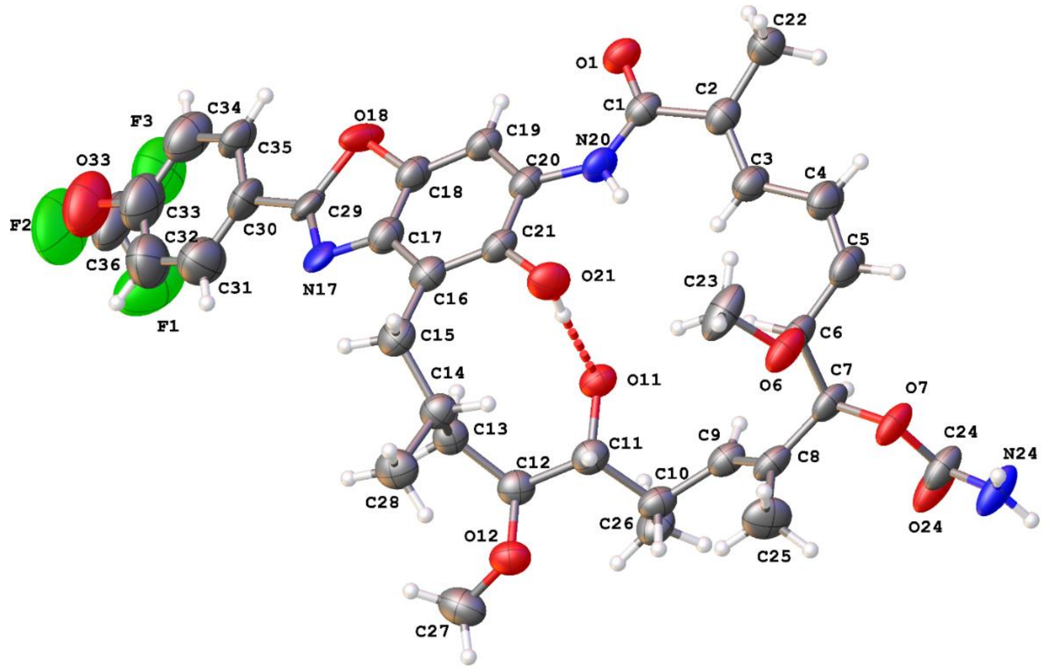

Figure S8 The asymmetric unit of **7a**. Displacement ellipsoids are shown at the 50% probability level. The substituent at C(29) is disordered over three sites and only one site with the major occupancy factor is shown in the diagram.

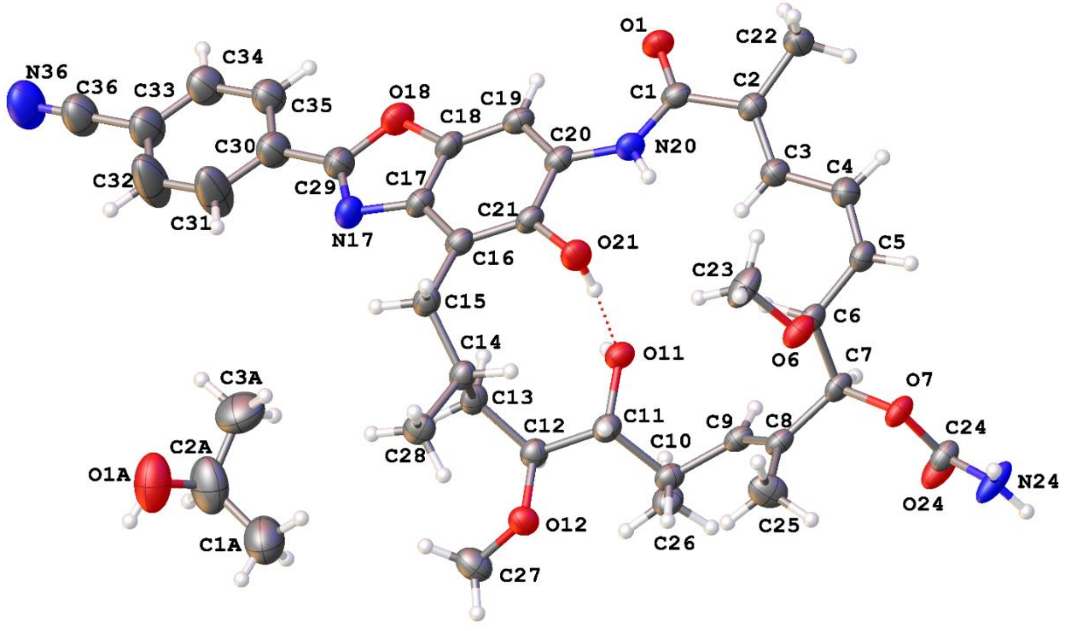

Figure S9 The asymmetric unit of **8a**. Displacement ellipsoids are shown at the 50% probability level.

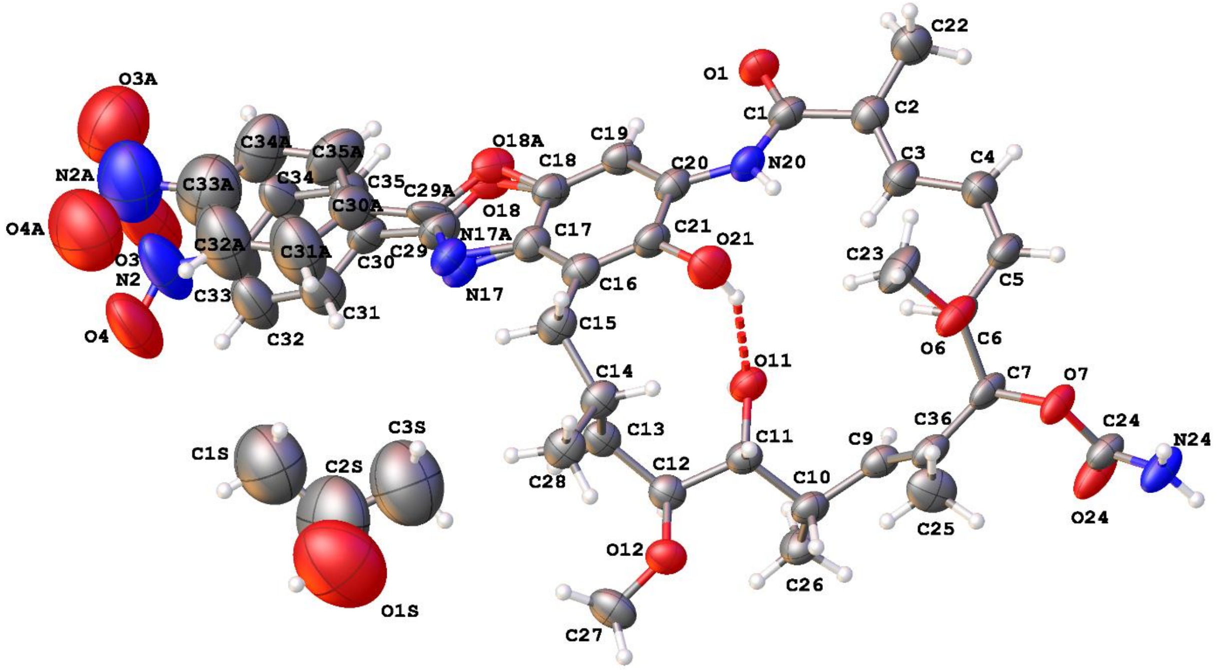

Figure S10 The asymmetric unit of **9a**. Displacement ellipsoids are shown at the 50% probability level. The substituent at C(29) is disordered over two sites. The isopropanol molecule has a partial occupancy.

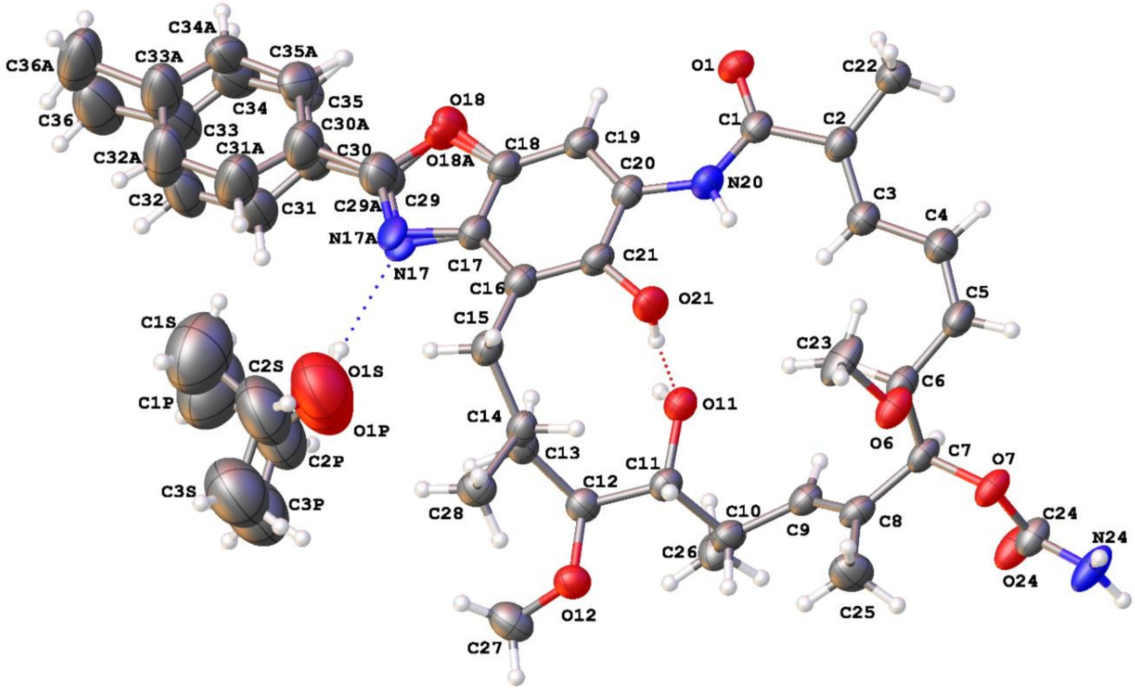

Figure S11 The asymmetric unit in **10a**. Displacement ellipsoids are shown at the 50% probability level. The isopropanol molecule and the substituent at C(29) are disordered.

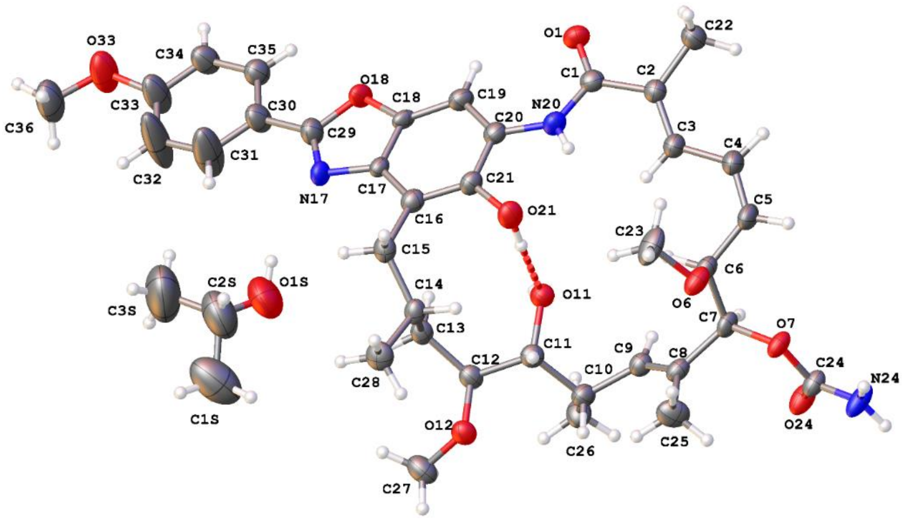

Figure S12 The asymmetric unit of **11a**. Displacement ellipsoids are shown at the 50% probability level. The substituent at C(29) is disordered over three sites and only one site with the major occupancy factor is shown in the diagram. The isopropanol solvent molecule has a partial occupancy.

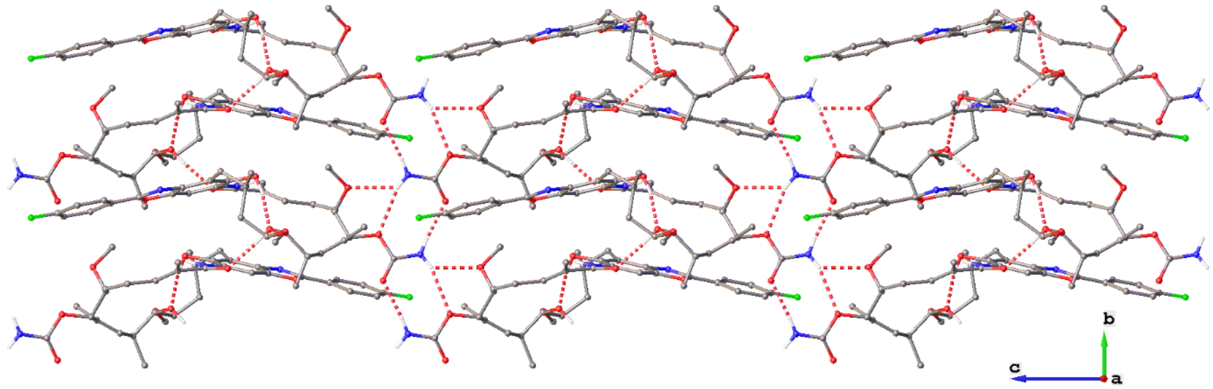

Figure S13 View of the 2D assembly *via* hydrogen bonds in **2a**. This structural motif is observed in all studied crystal structures.

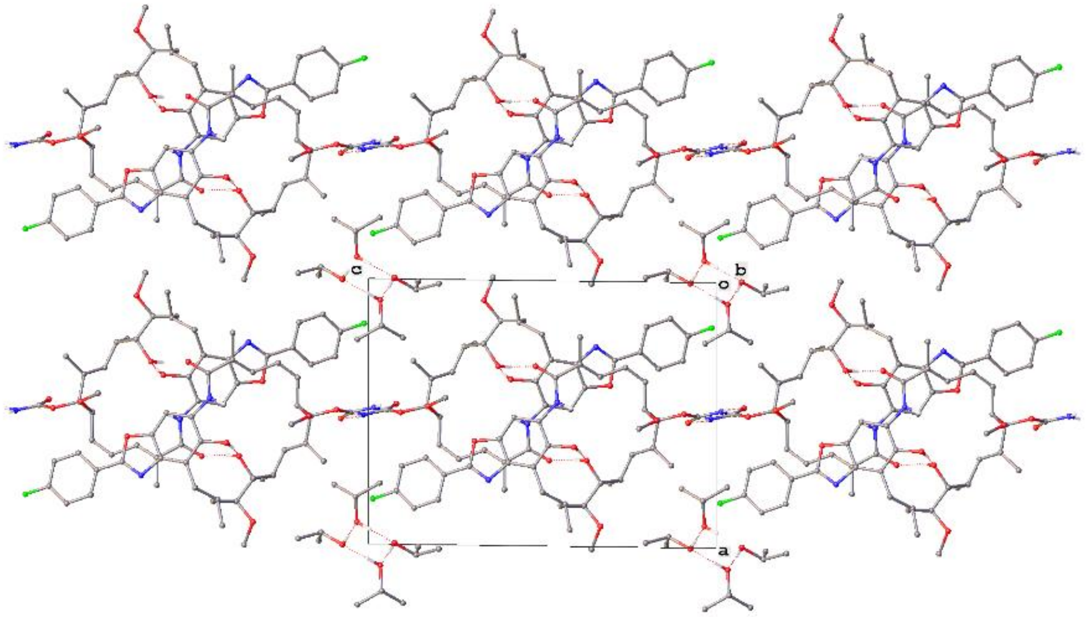

Figure S14 Crystal packing of **2a** shown in the projection along the b axis. Hydrogen bonded isopropanol molecules form a chain extended along the b axis.

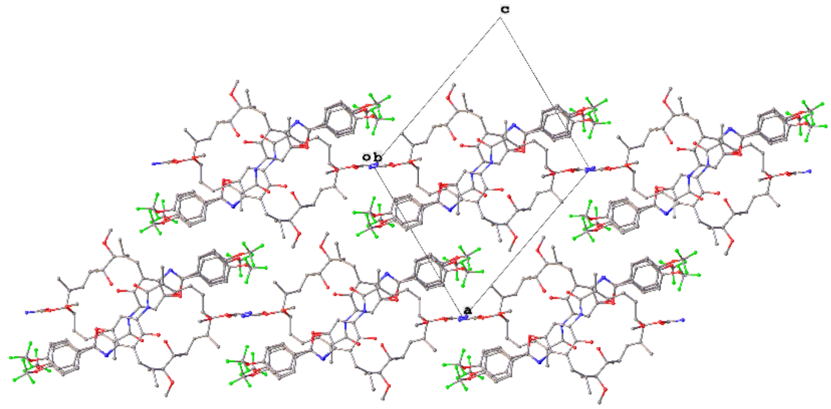

Figure S15 Crystal packing of **7a** shown in the projection along the b axis.

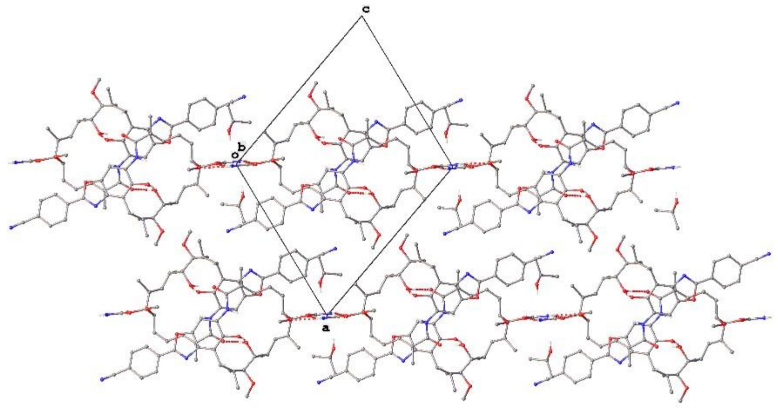

Figure S16 Crystal packing of **8a** shown in the projection along the b axis. The isopropanol solvent molecule in the site with the major occupation is not hydrogen-bonded to the host molecules.

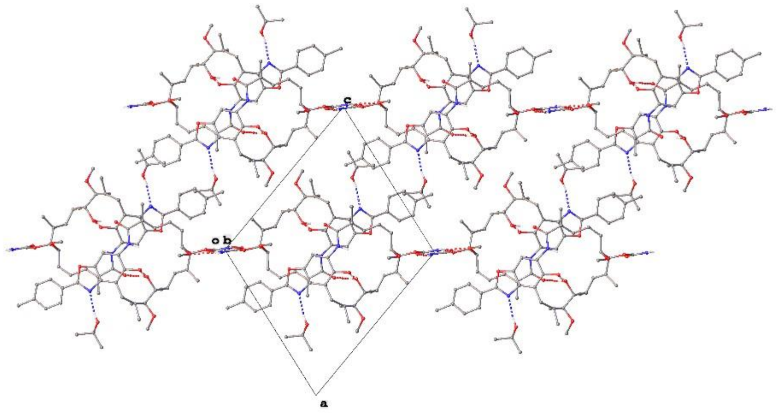

Figure S17 Crystal packing of **10a** shown in the projection along the b axis. Isopropanol molecules are hydrogen boded to the host molecules.

Figure S18 Progress of the substrate conversion [%] per unit of time [min] for derivatives **1a**-**11a**. The colors and shapes were assigned appropriately to the derivatives: **1a**-black square, **2a**-pink square, **3a**-red triangle, **4a**-blue triangle, **6a**-green triangle, **7a**-purple triangle, **8a**-orange dot, **9a**-blue dot, **10a**-green diamond, **11a**-purple diamond, **12a**-orange diamond.

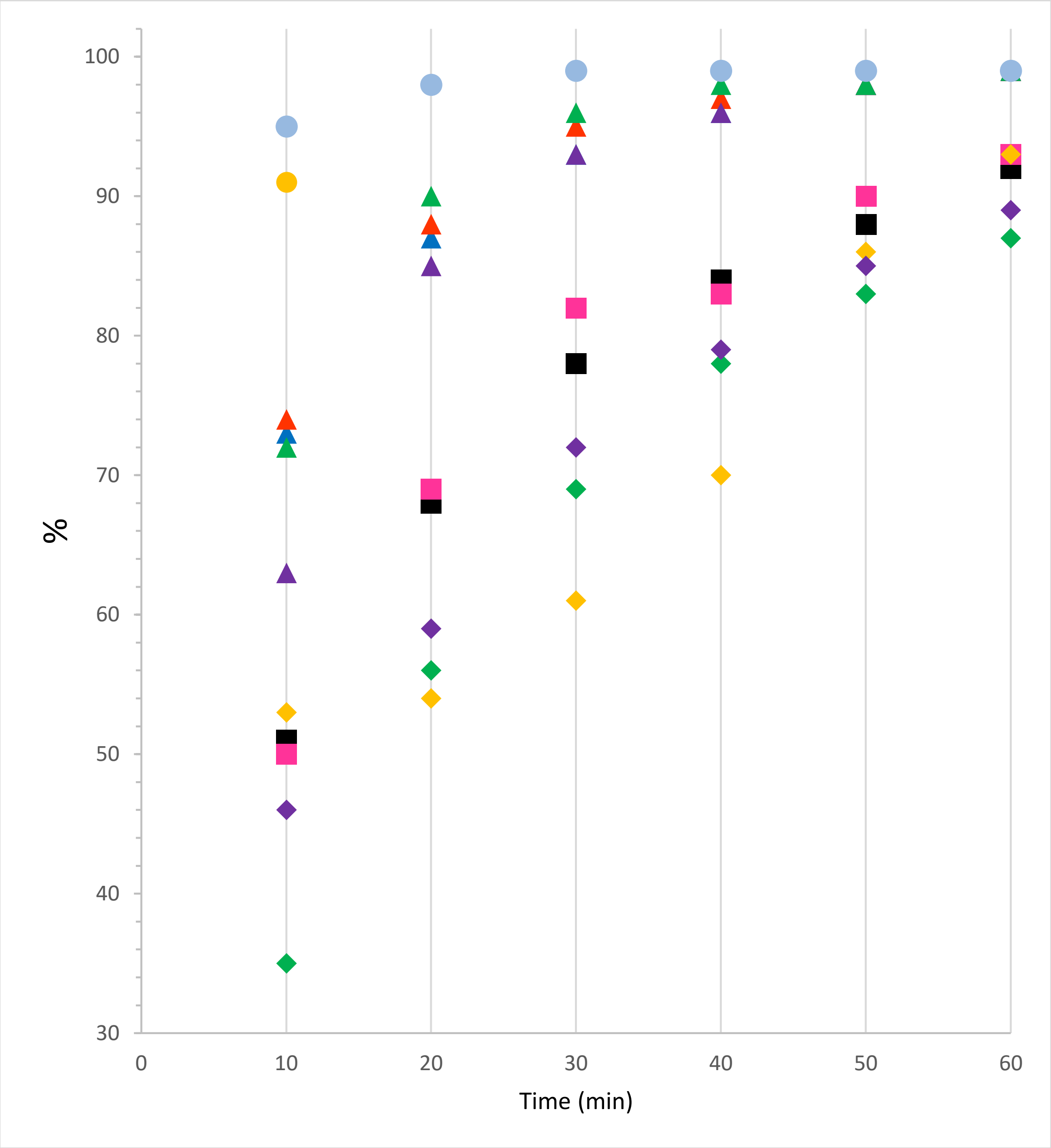

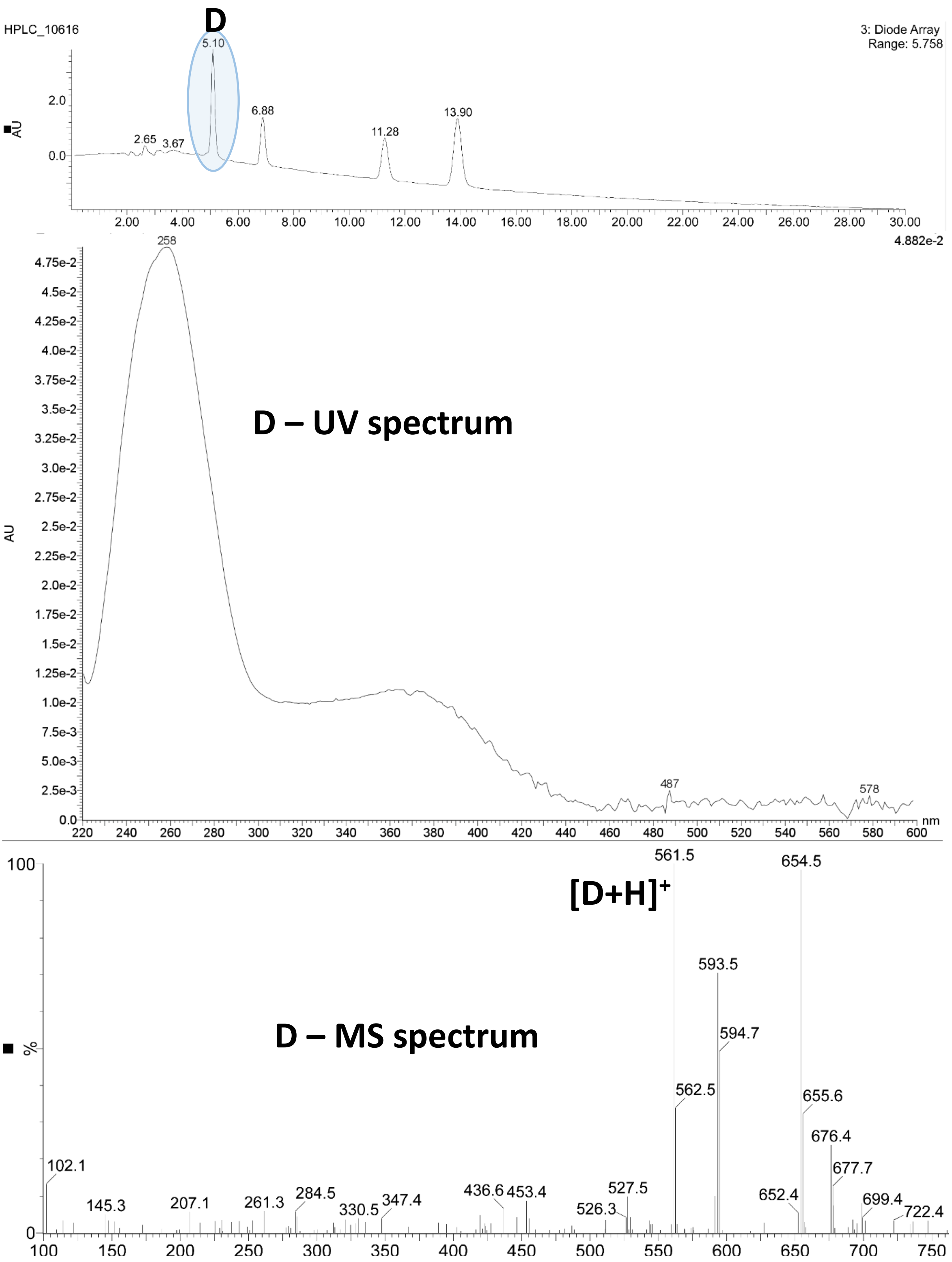

Figure S19 HPLC chromatogram recorded for the reaction mixture during conversion **2** to **2a**, captured with intermediates (**2'**and **2"**). UV-vis and MS spectrum for the product of the first intermediate **2'**.

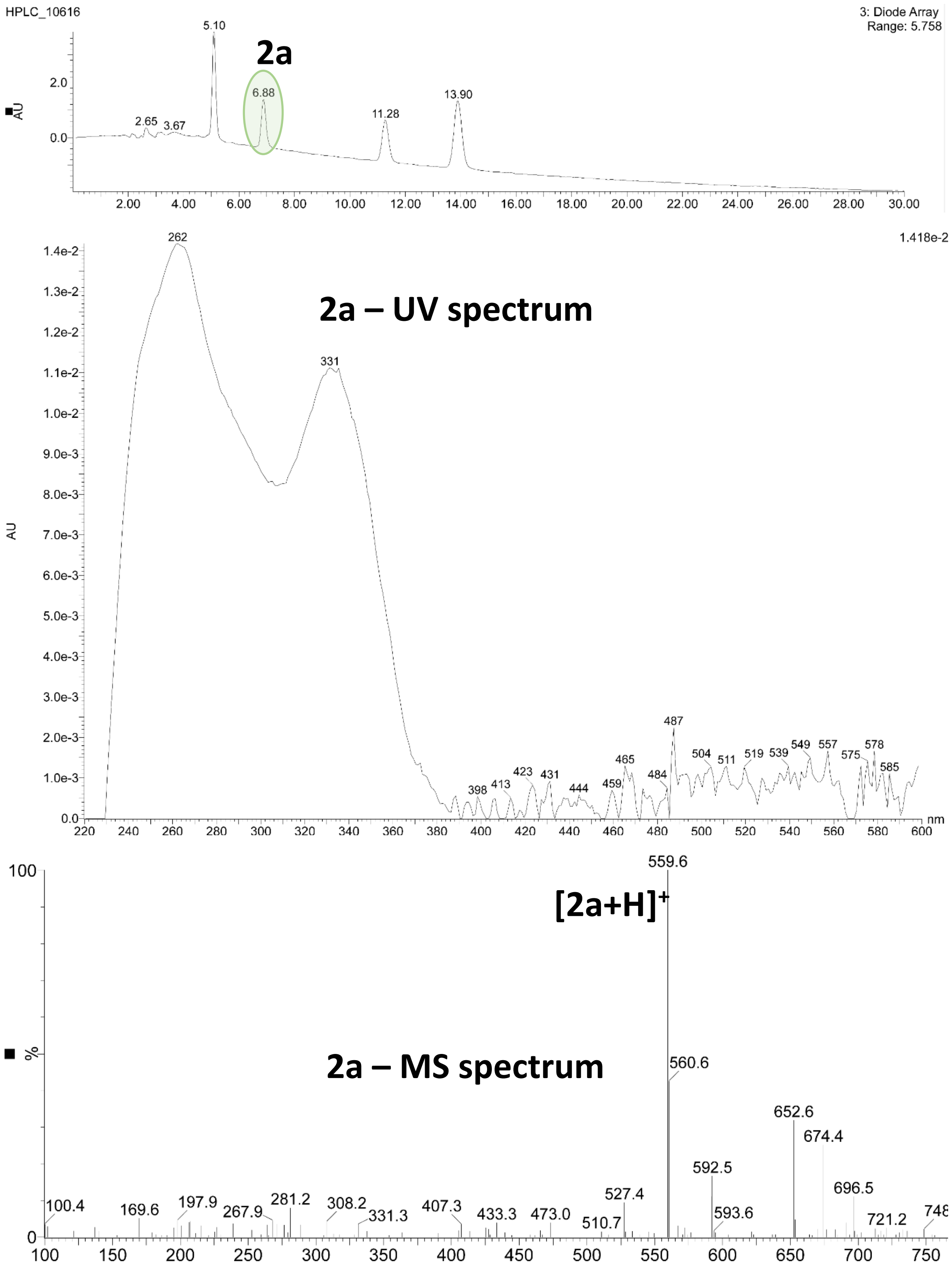

Figure S20 HPLC chromatogram recorded for the reaction mixture during conversion **2** to **2a**, captured with intermediates (**2’** and **2”**). UV-vis and MS spectrum for the product **2a**.

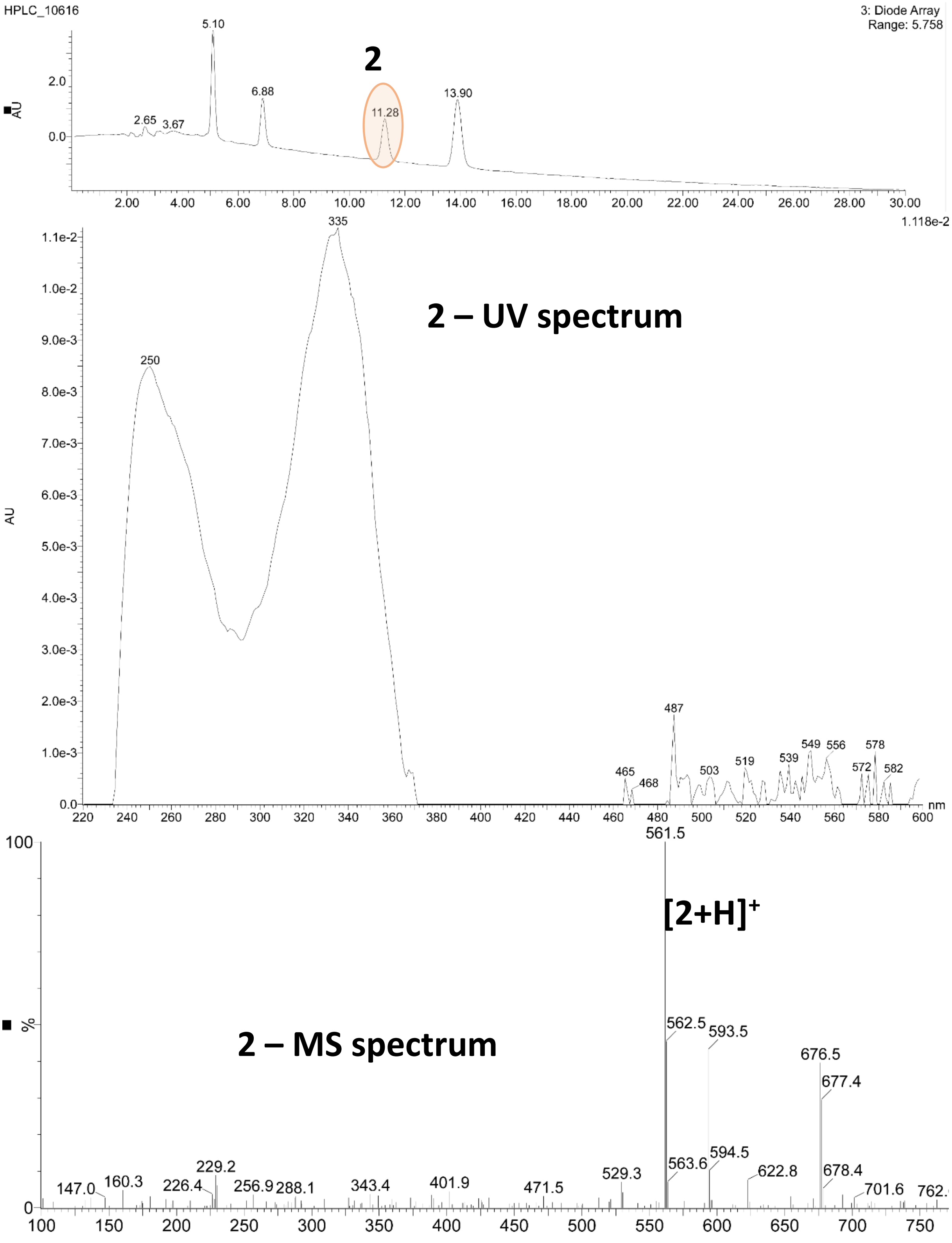

Figure S21 HPLC chromatogram recorded for the reaction mixture during conversion **2** to **2a**, captured with intermediates (**2’** and **2”**). UV-vis and MS spectrum for substrate **2**.

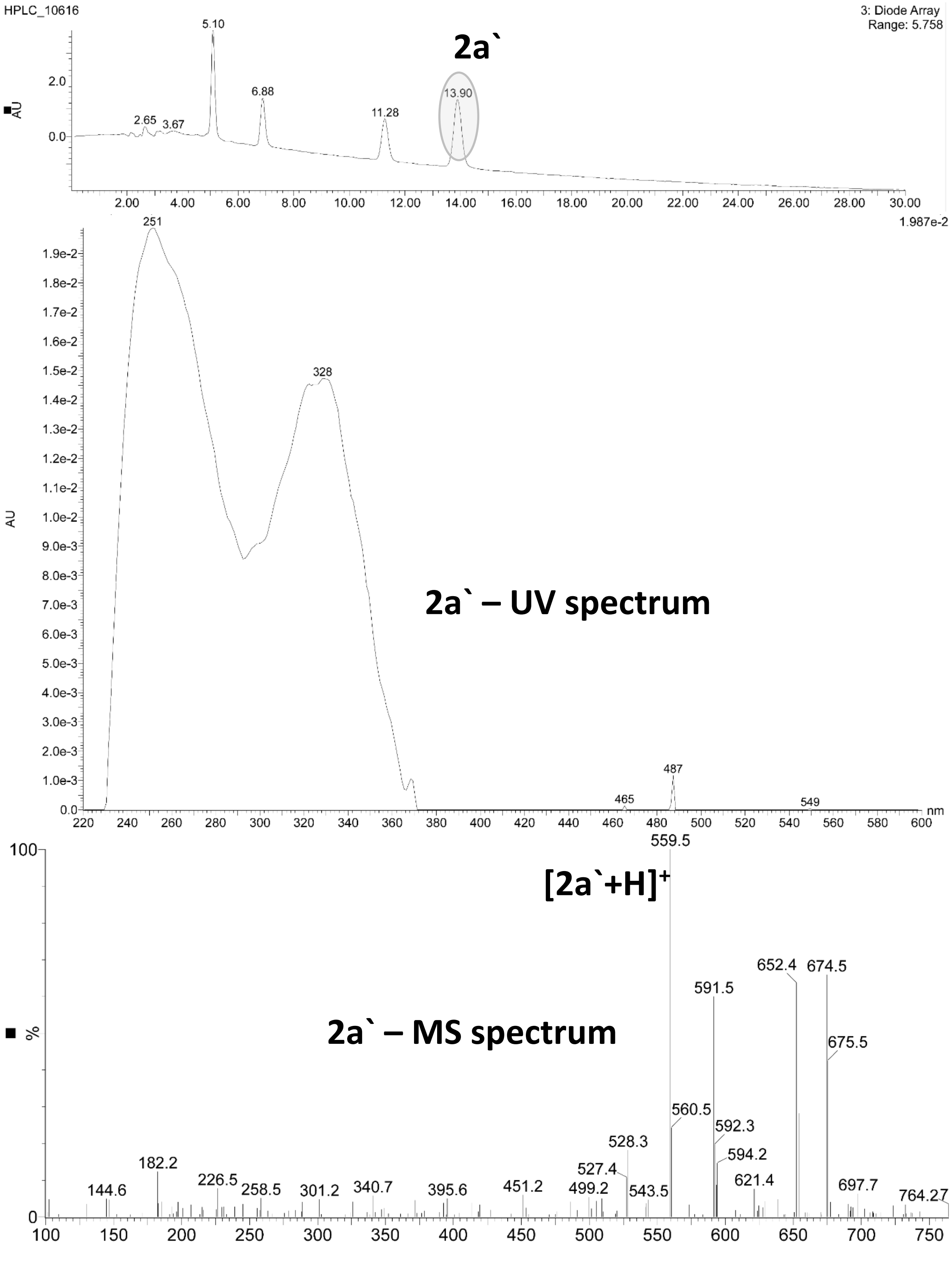

Figure S22 HPLC chromatogram recorded for the reaction mixture during conversion **2** to **2a**, captured with intermediates (**2’**and **2”**). UV-vis and MS spectrum for the product of the second intermediate **2”**.

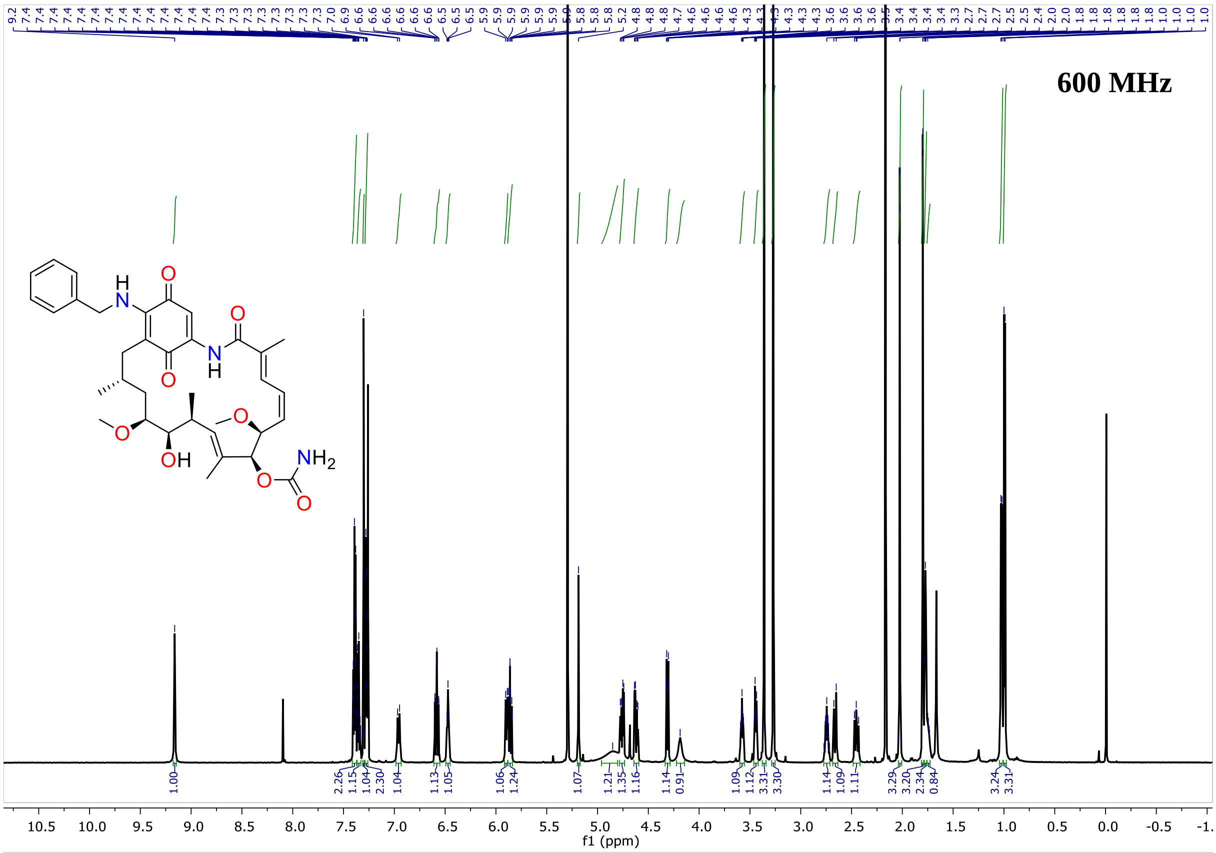

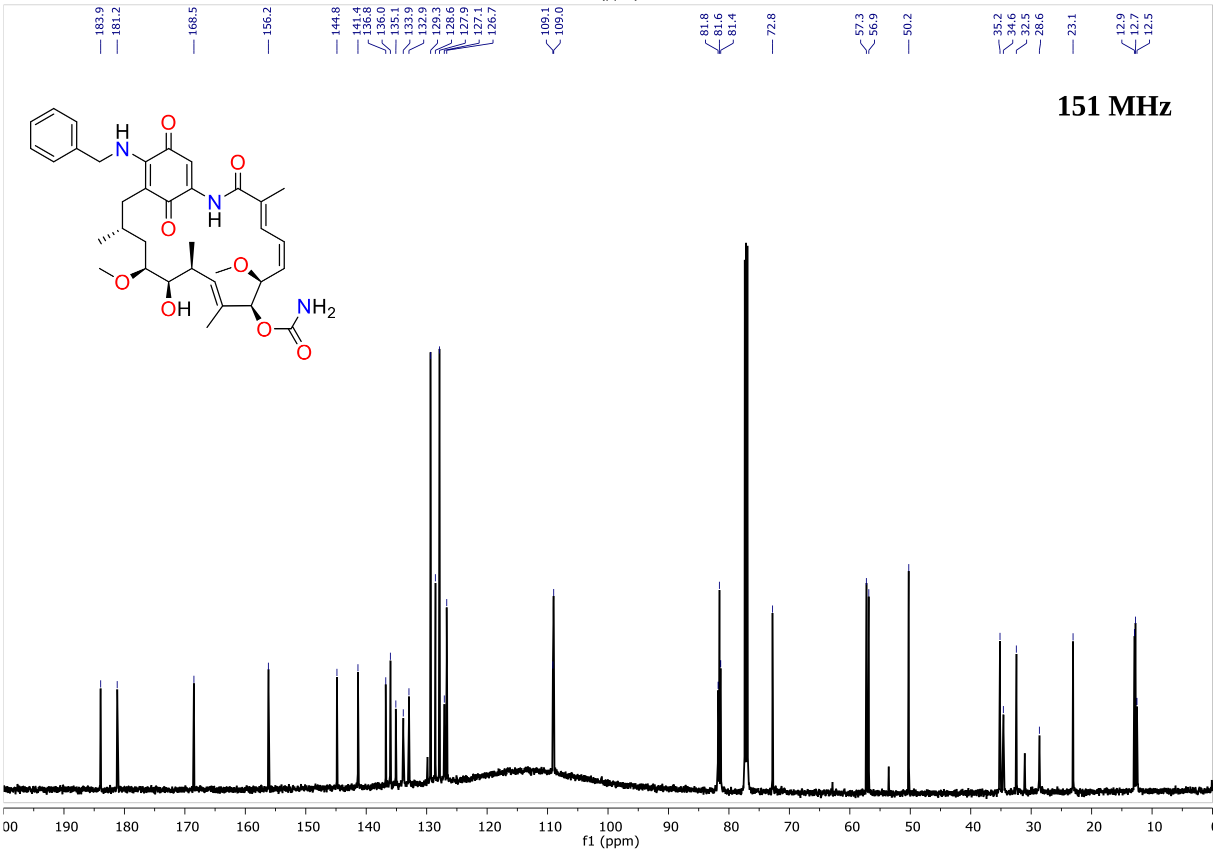

Figure S23 ^{1}H and $^{13}C\{1H\}$ spectra of compound **1** in $CDCl_3$.

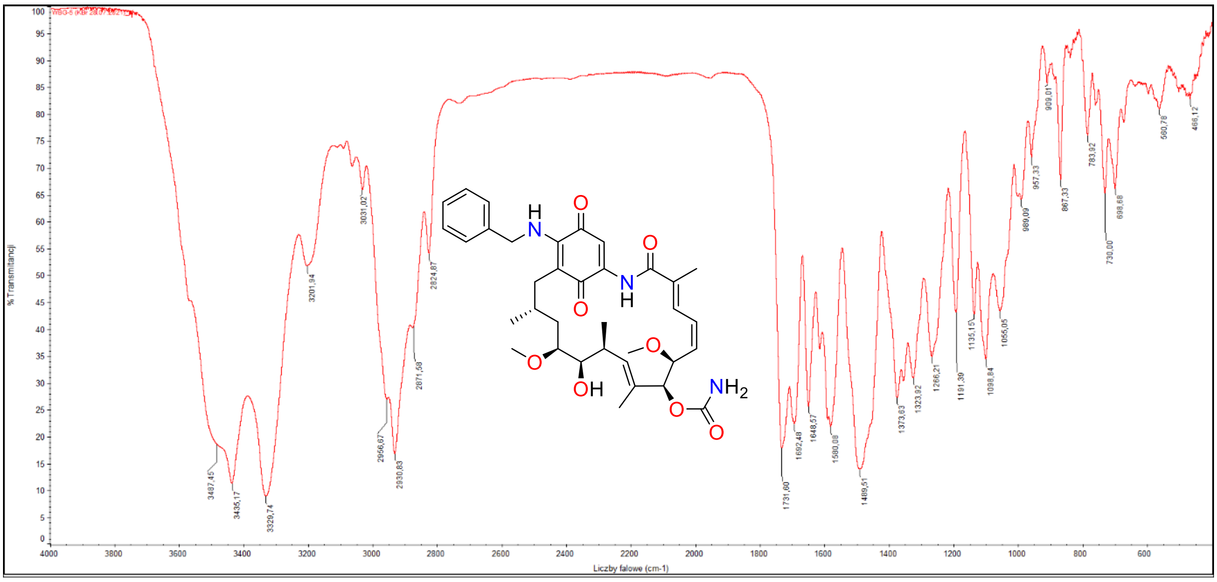

Figure S24 FT-IR spectrum of compound **1** (in KBr).

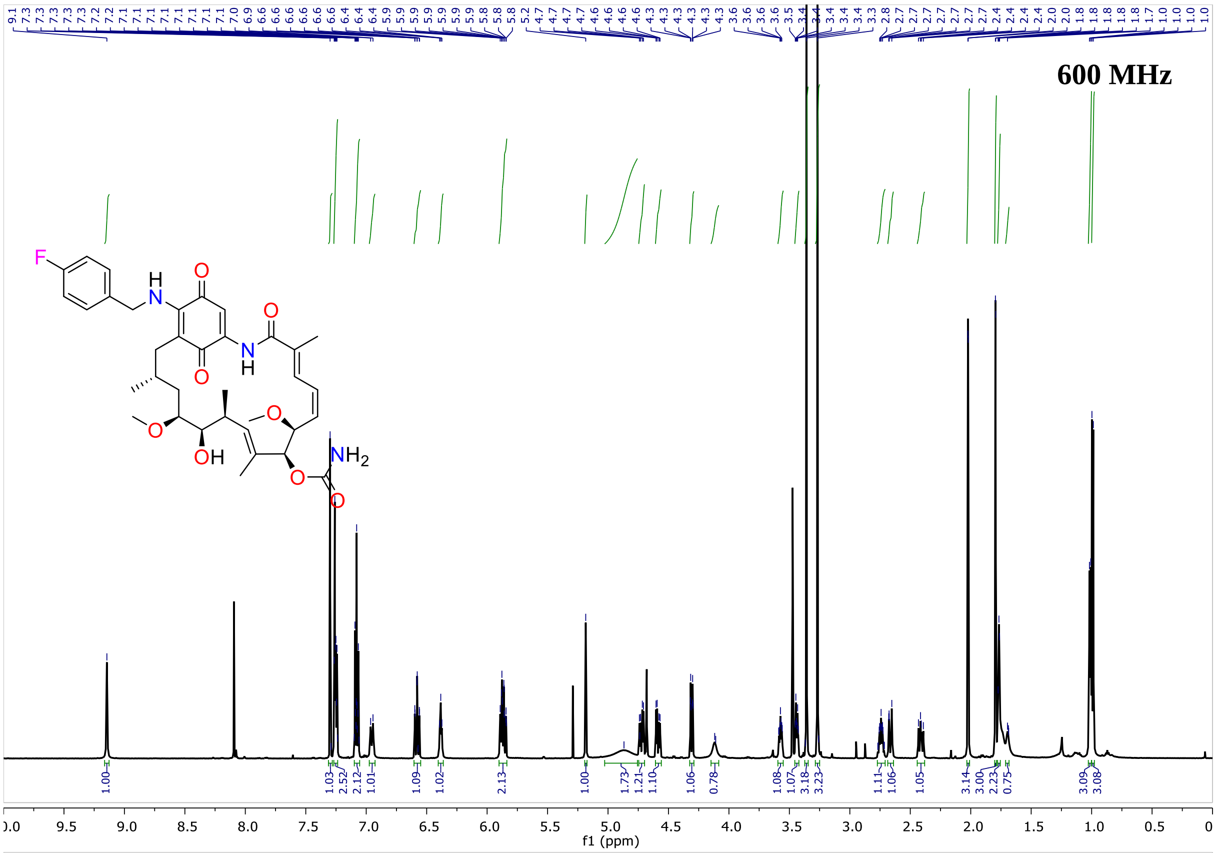

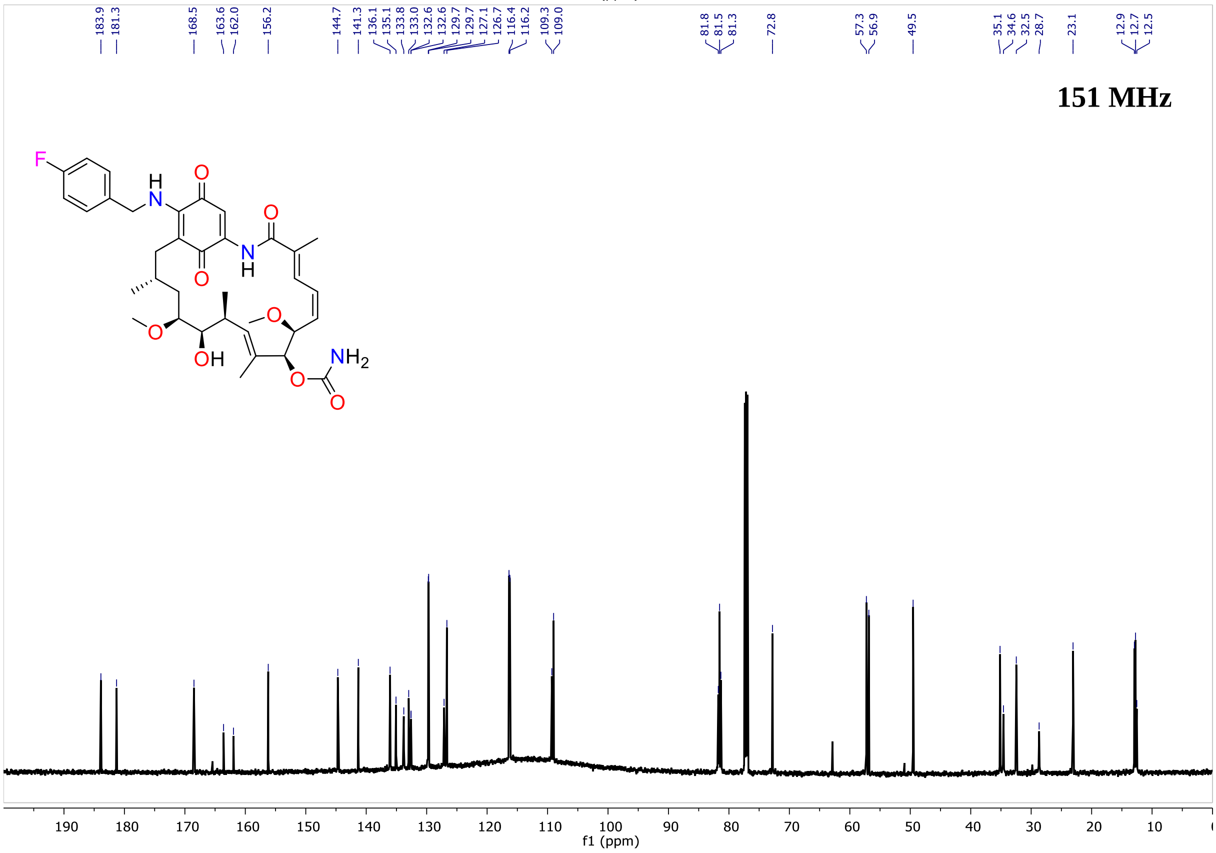

Figure S25 ^{1}H and $^{13}C\{1H\}$ spectra of compound **2** in $CDCl_3$.

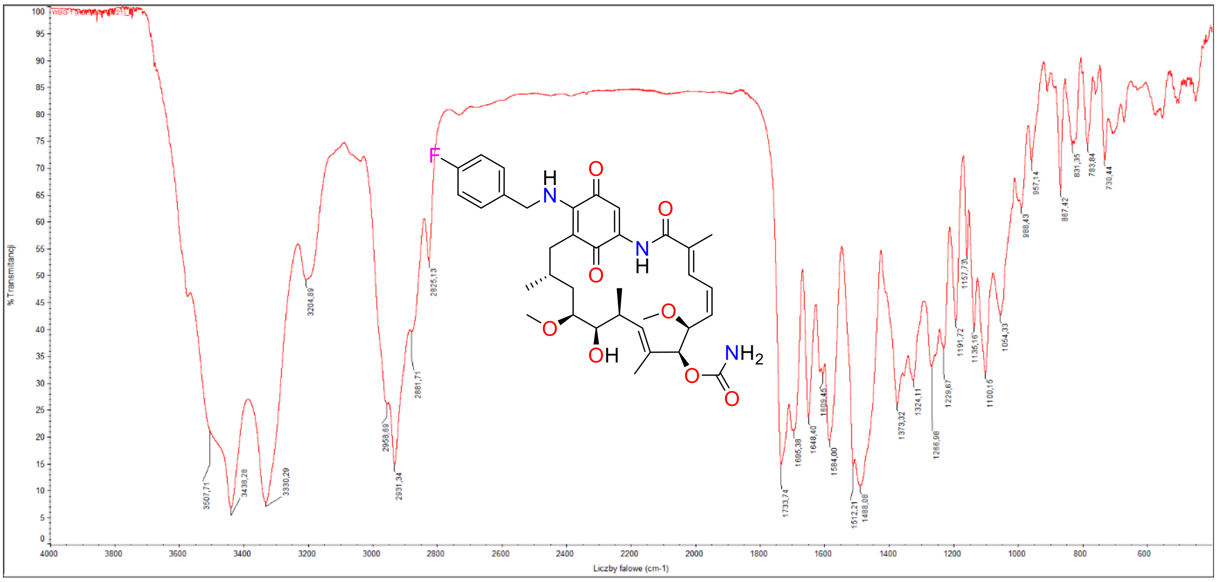

Figure S26 FT-IR spectrum of compound **2** (in KBr).

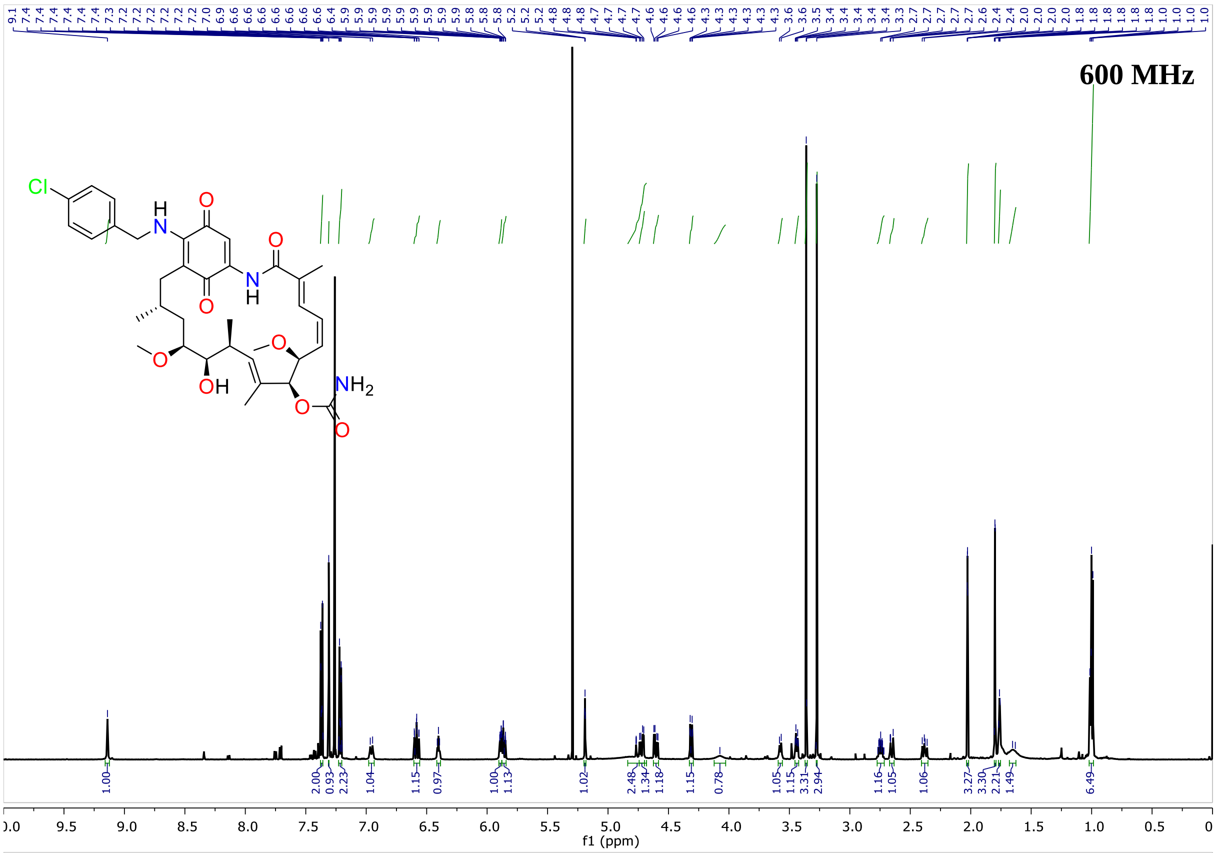

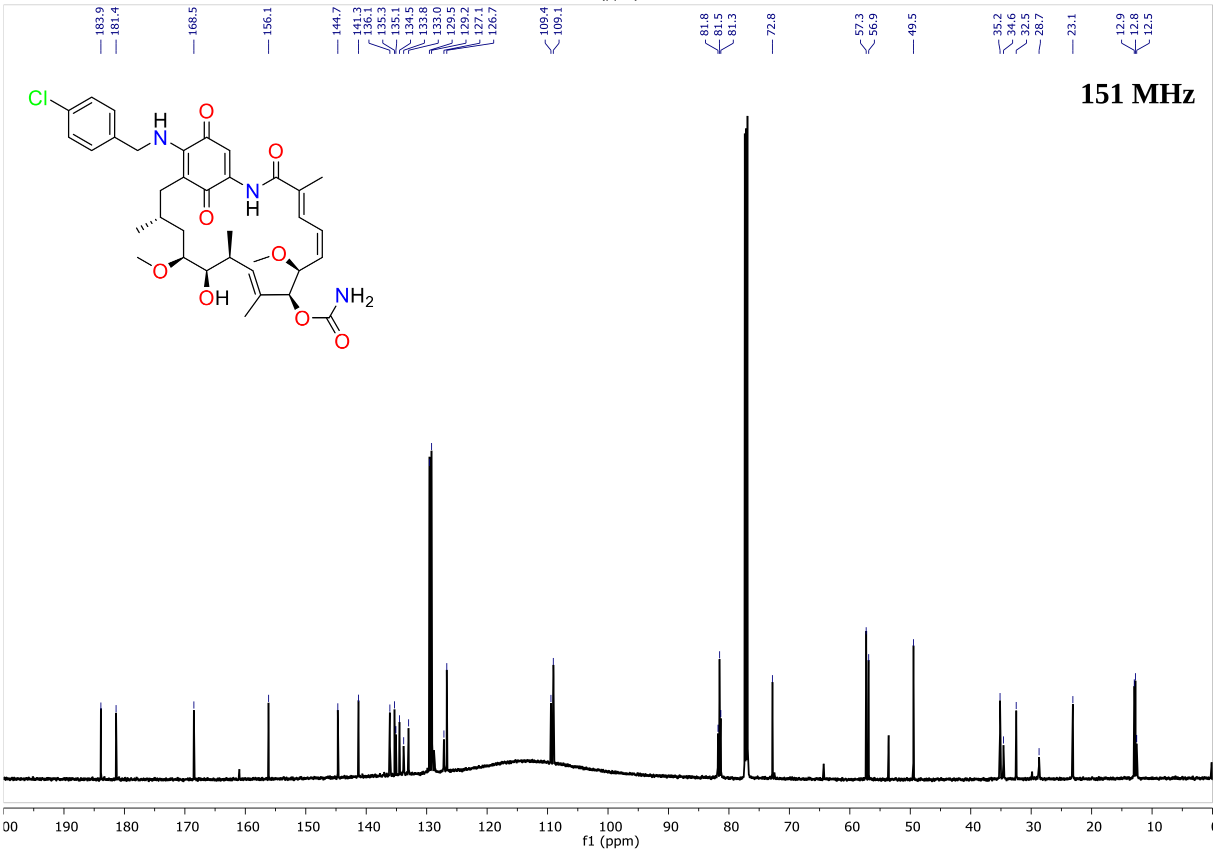

Figure S27 ^{1}H and ^{13}C{1H} spectra of compound **3** in $CDCl_3$.

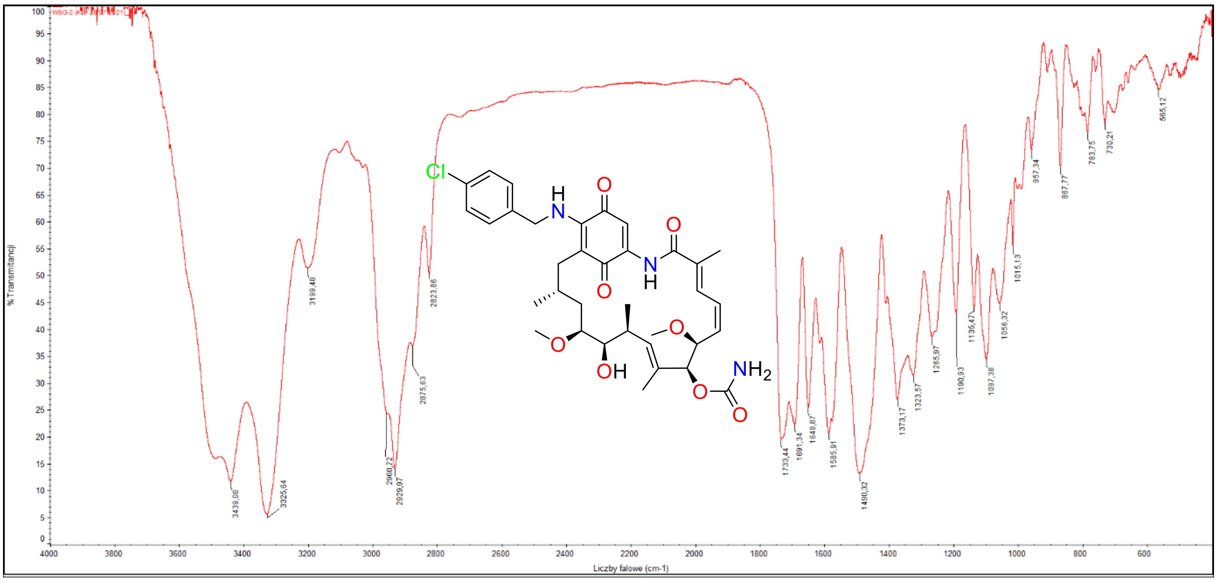

Figure S28 FT-IR spectrum of compound **3** (in KBr).

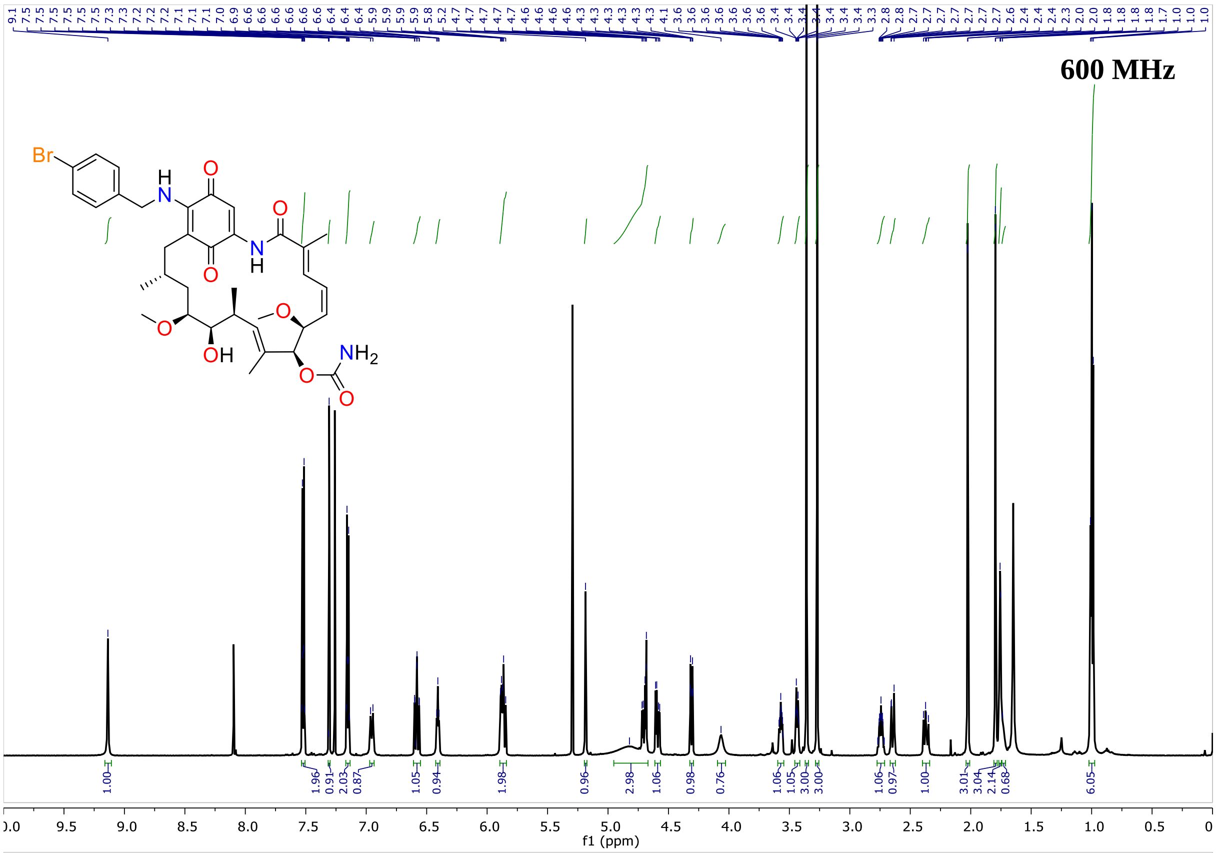

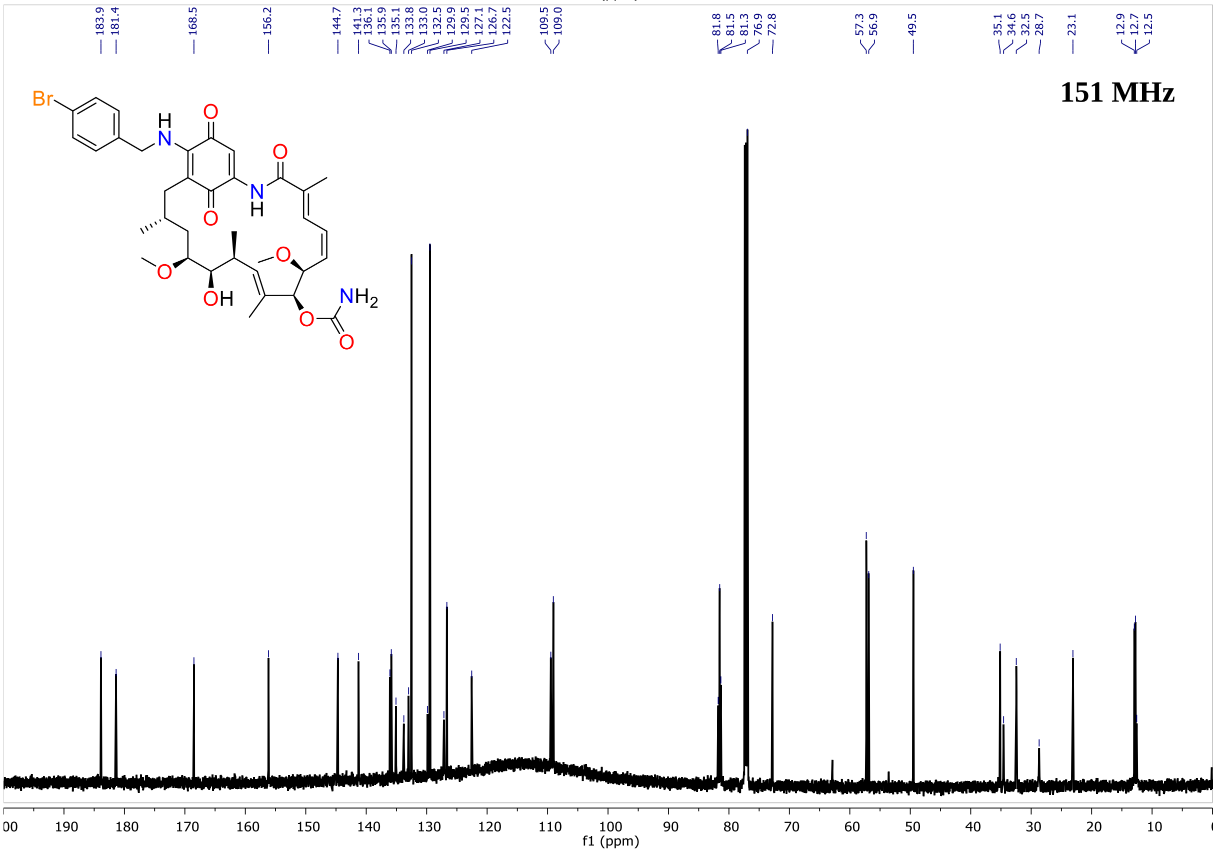

Figure S29 ^{1}H and $^{13}C\{1H\}$ spectra of compound **4** in $CDCl_3$.

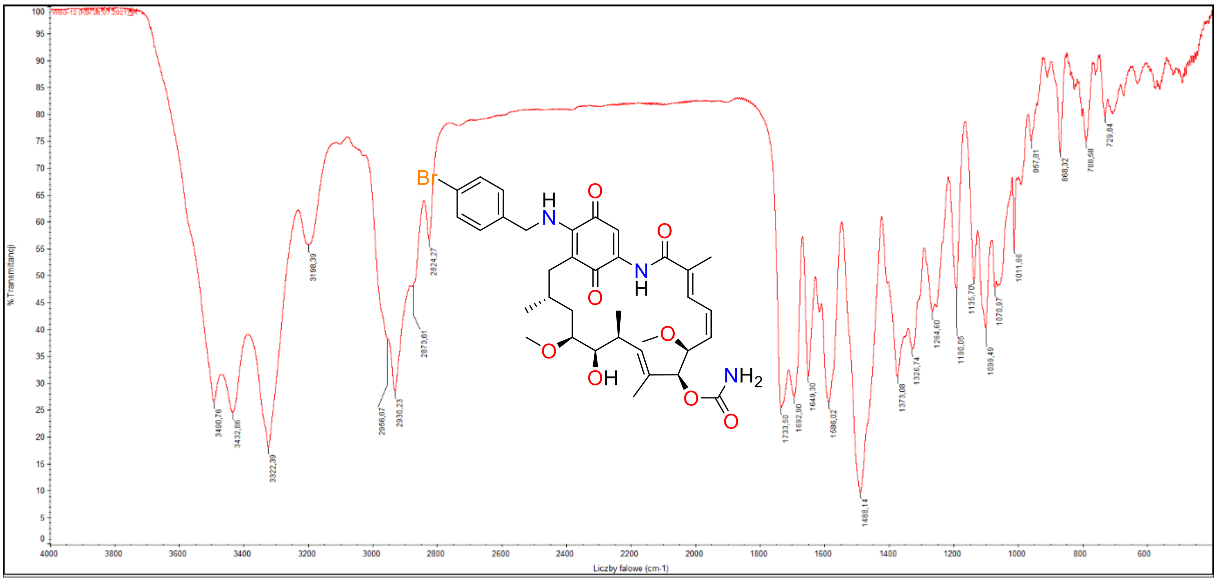

Figure S30 FT-IR spectrum of compound **4** (in KBr).

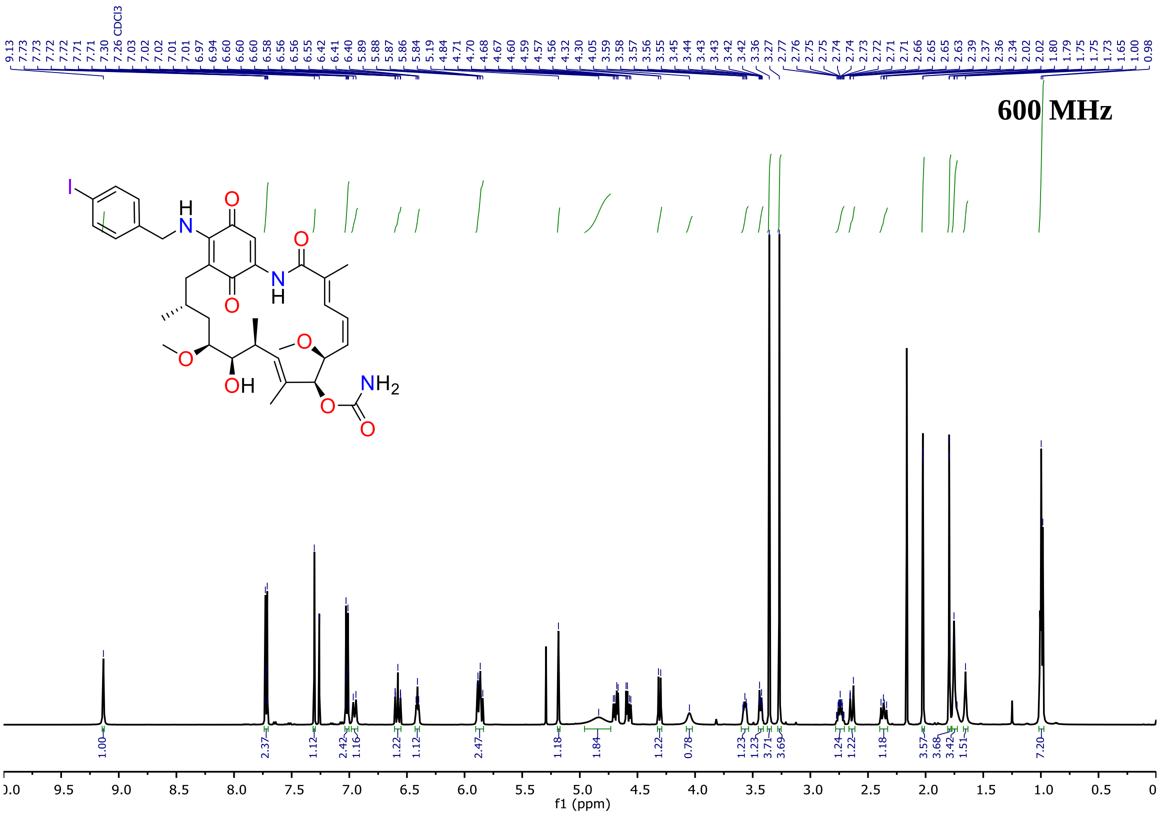

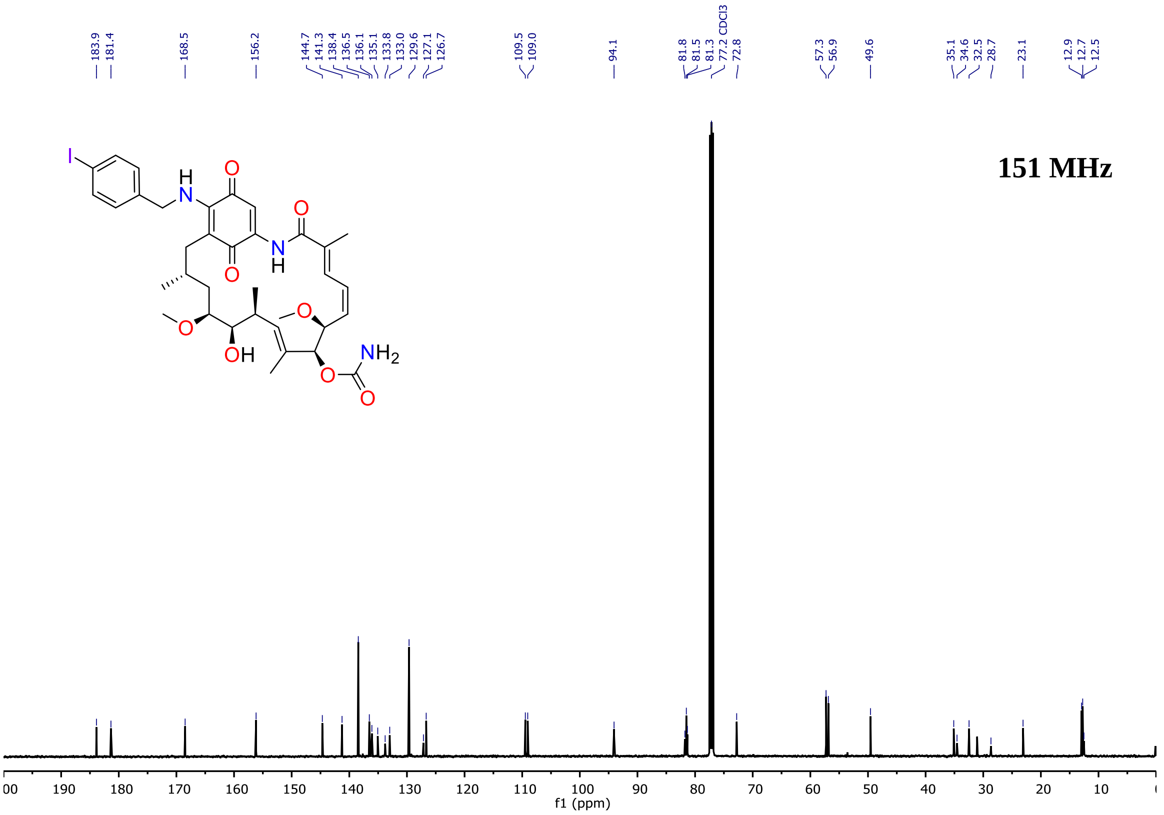

Figure S31 ^{1}H and ^{13}C{1H} spectra of compound **5** in $CDCl_3$.

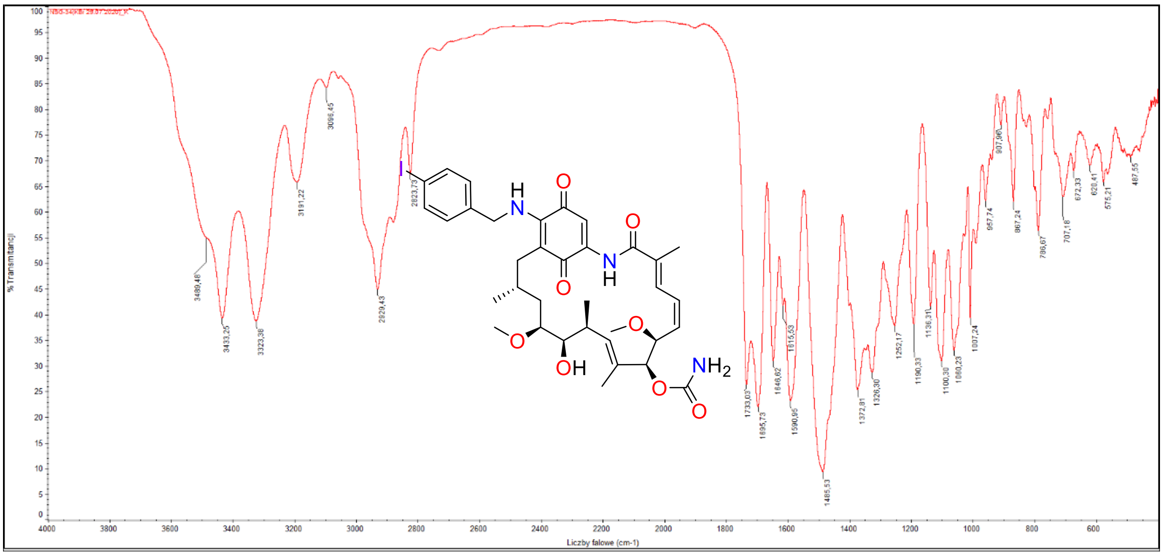

Figure S32 FT-IR spectrum of compound **5** (in KBr).

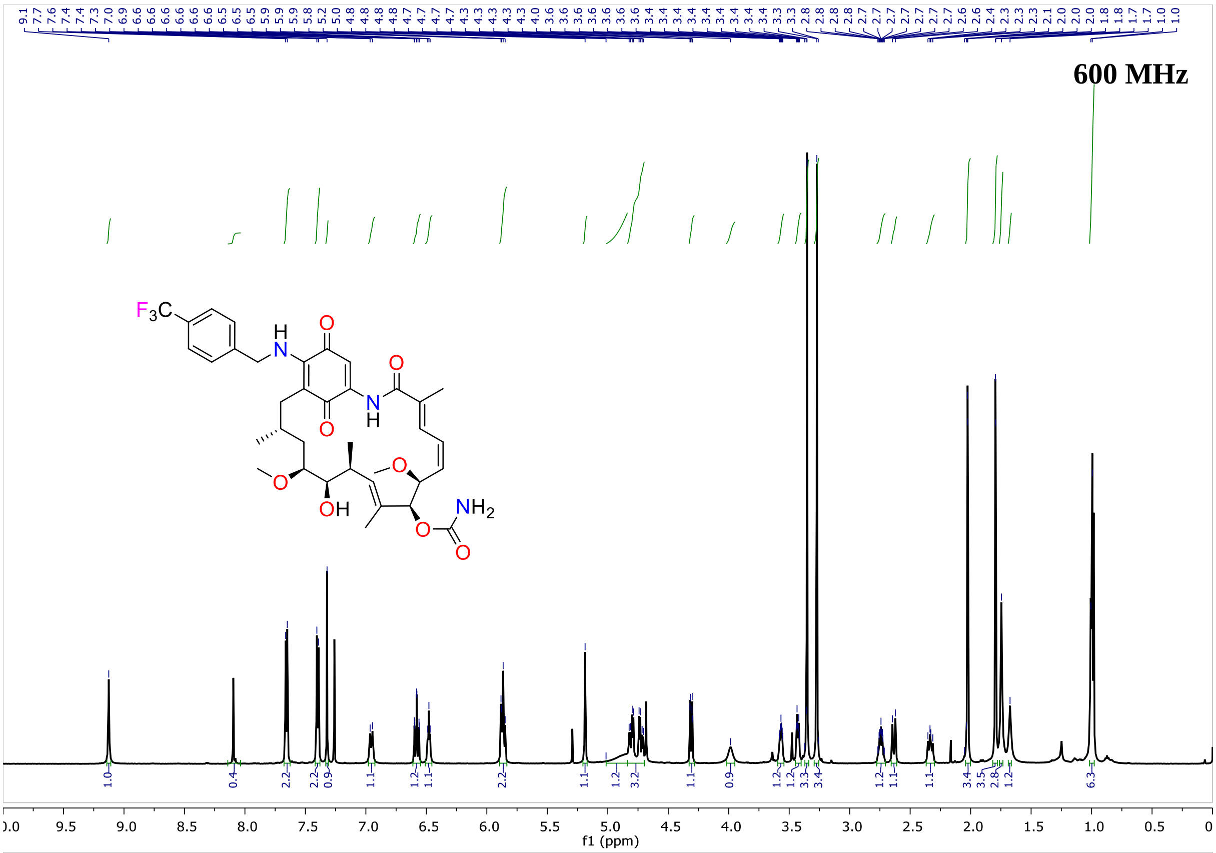

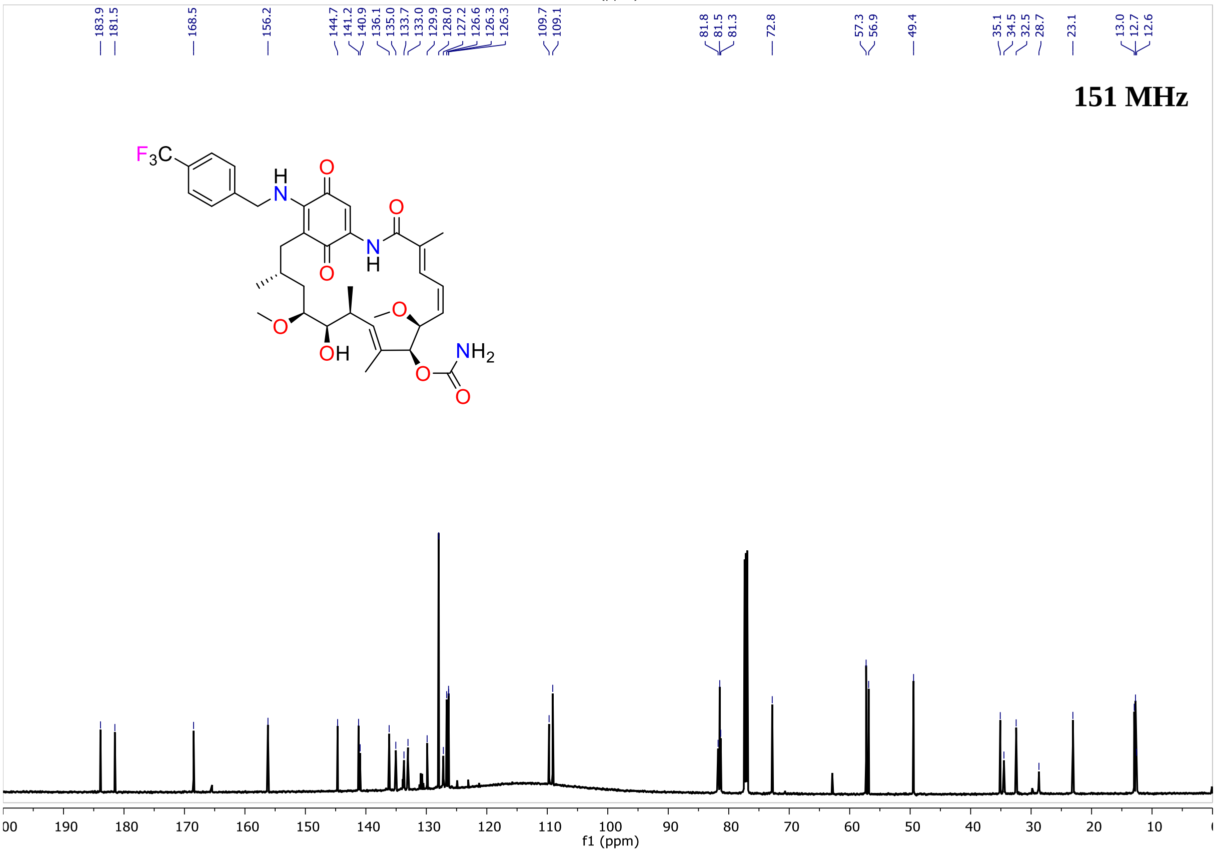

Figure S33 ^{1}H and $^{13}C\{1H\}$ spectra of compound **6** in $CDCl_3$.

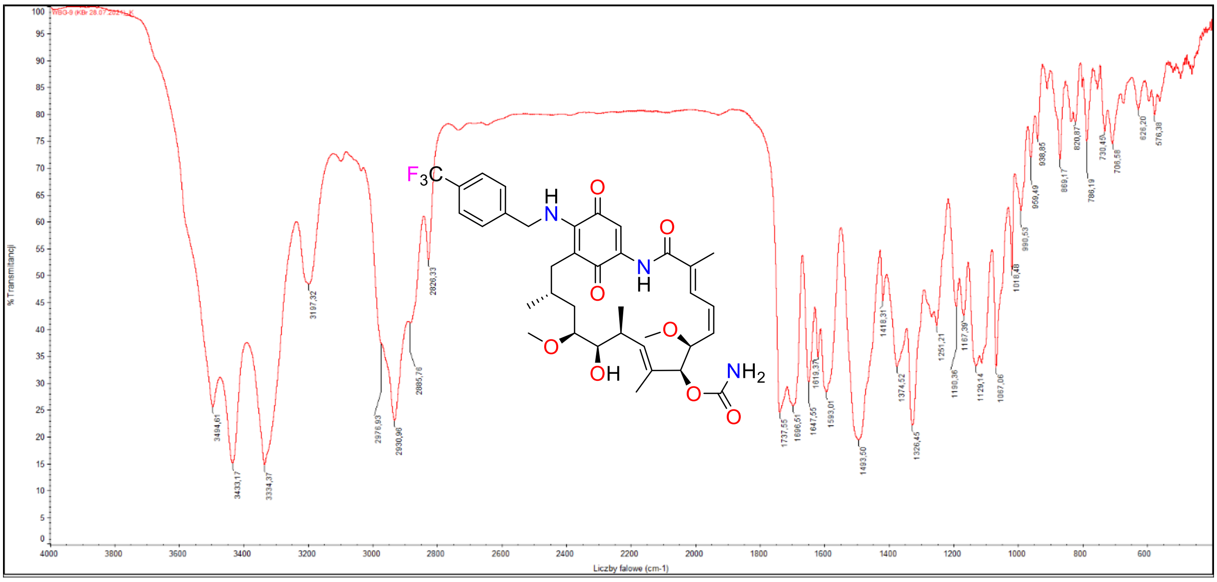

Figure S34 FT-IR spectrum of compound **6** (in KBr).

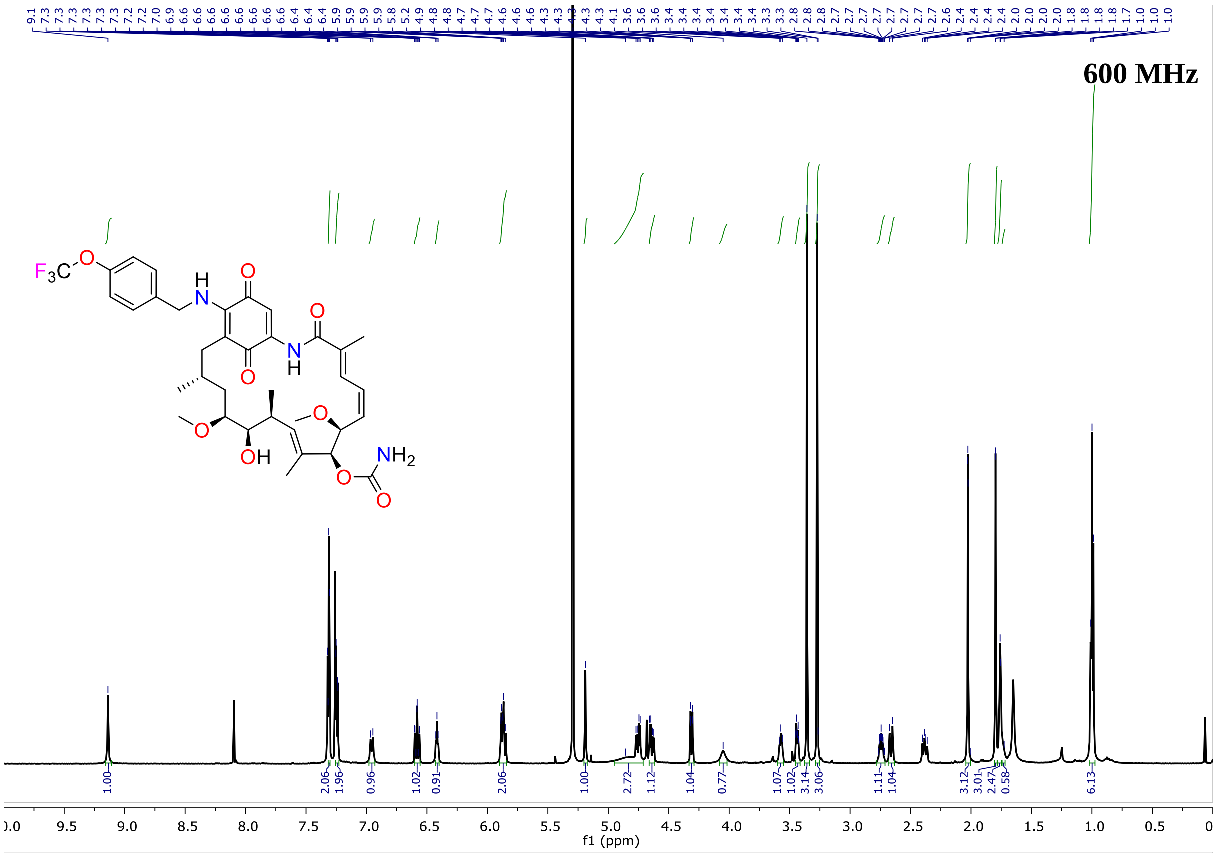

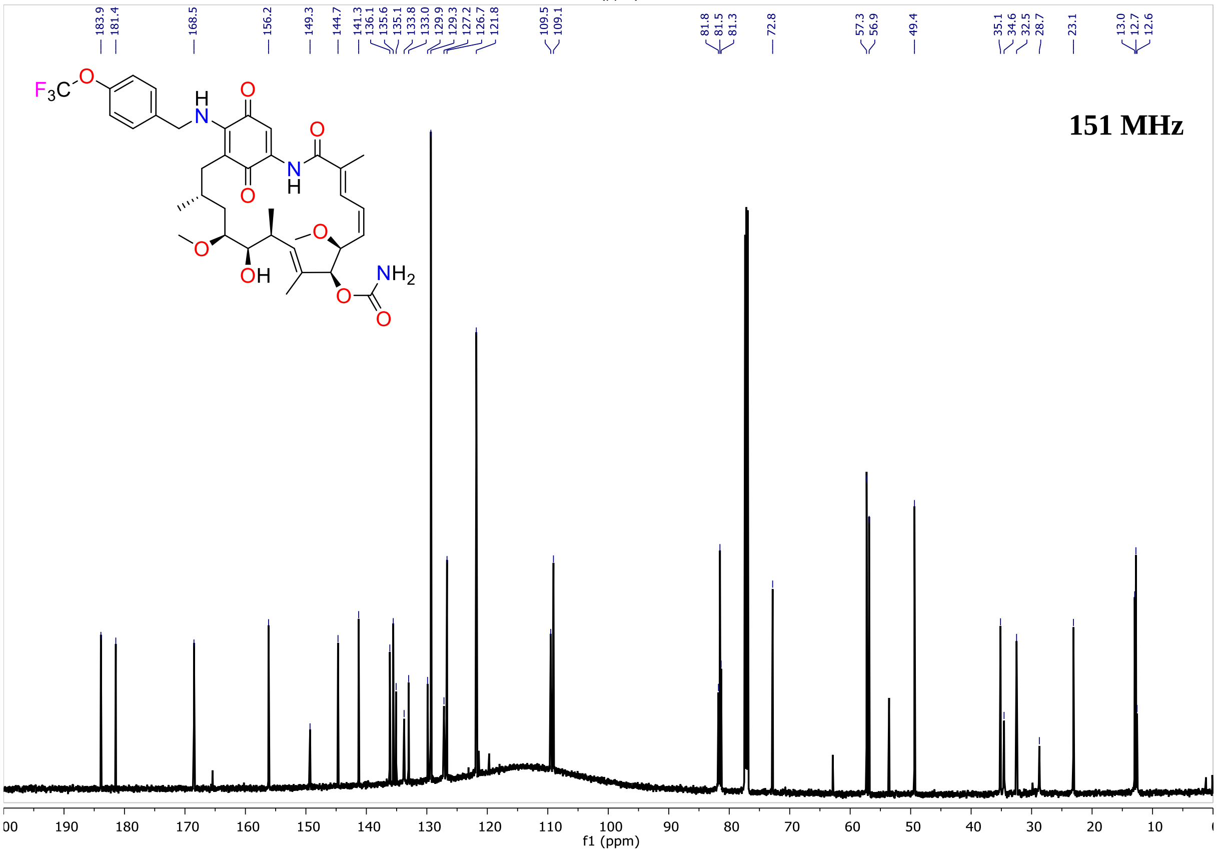

Figure S35 ^{1}H and ^{13}C{1H} spectra of compound **7** in $CDCl_3$.

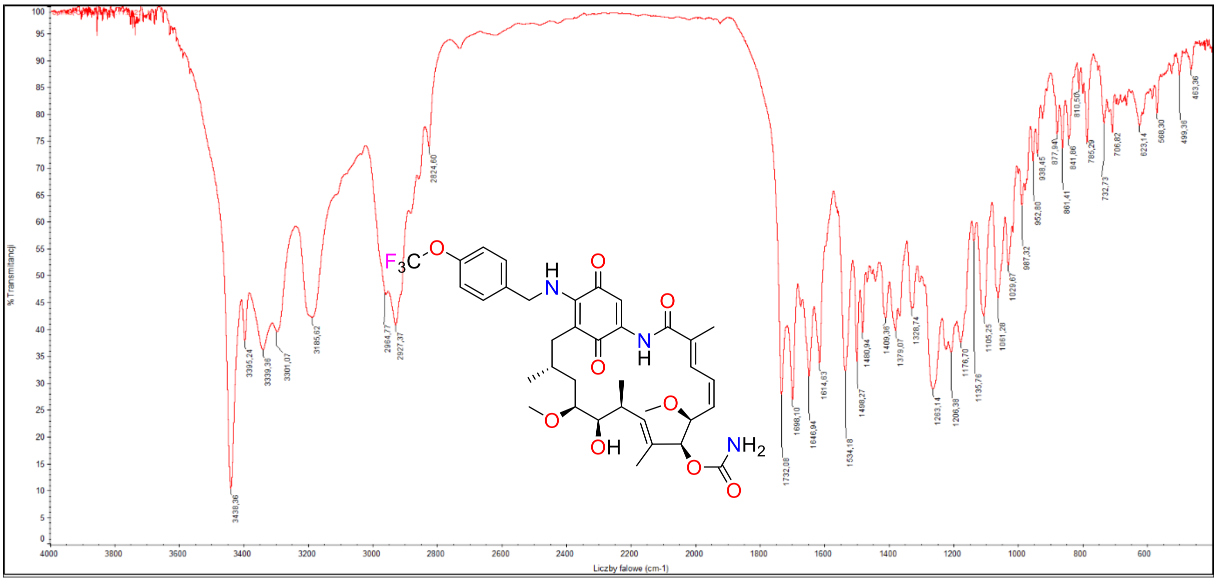

Figure S36 FT-IR spectrum of compound **7** (in KBr).

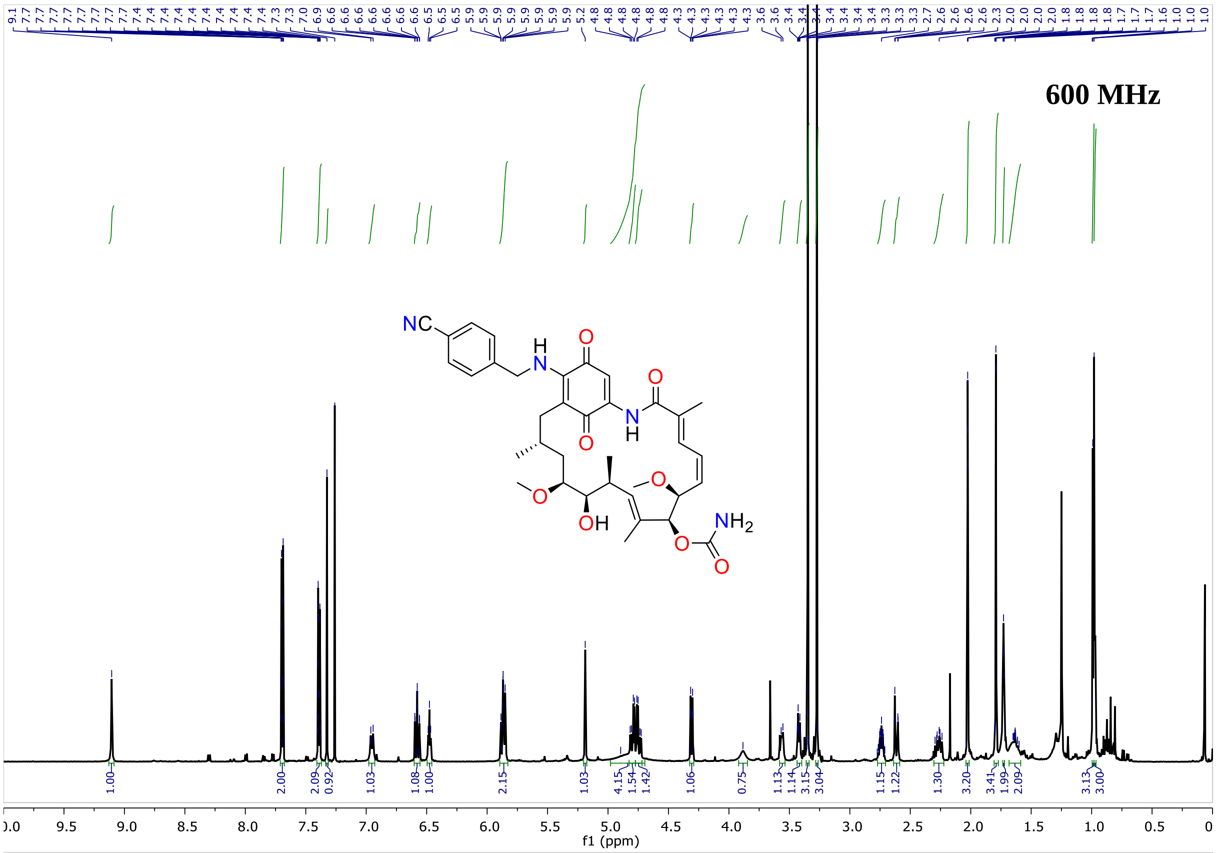

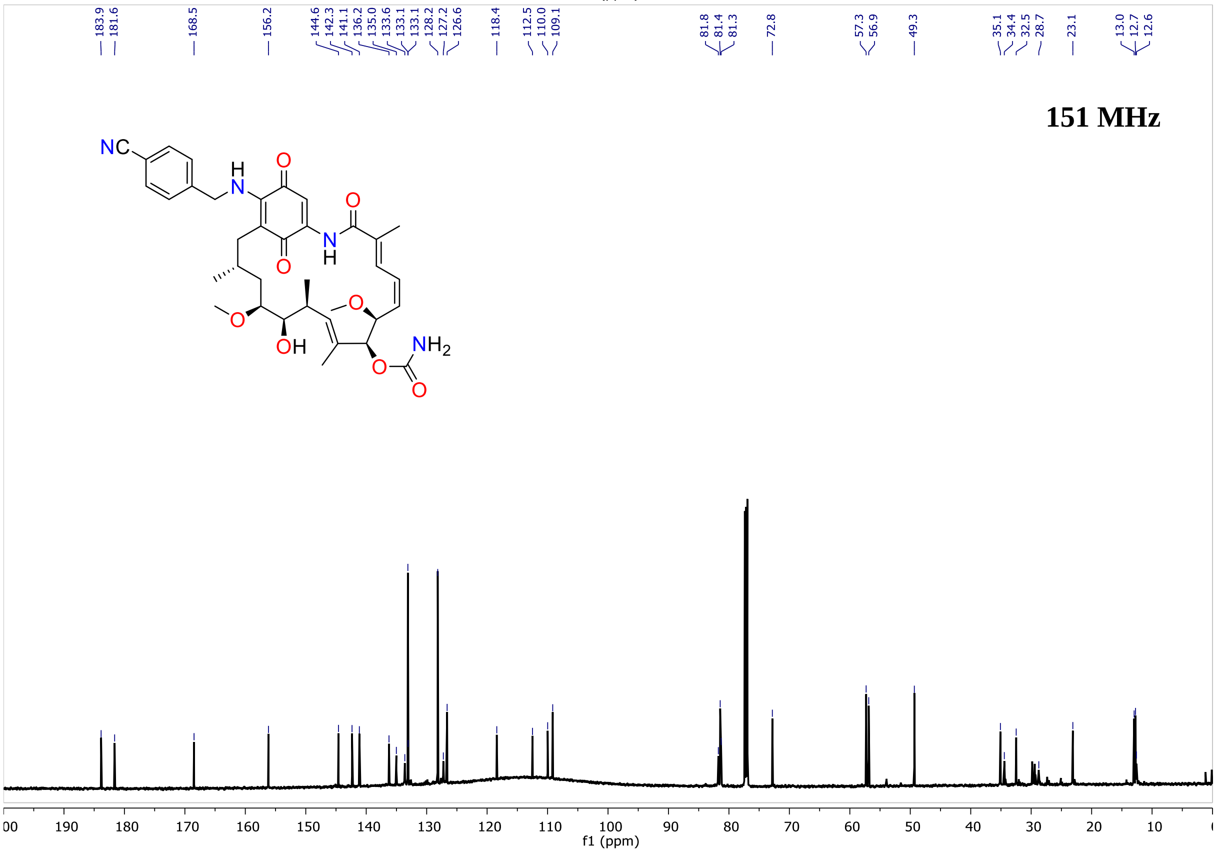

Figure S37 ^{1}H and $^{13}C\{1H\}$ spectra of compound **8** in $CDCl_3$.

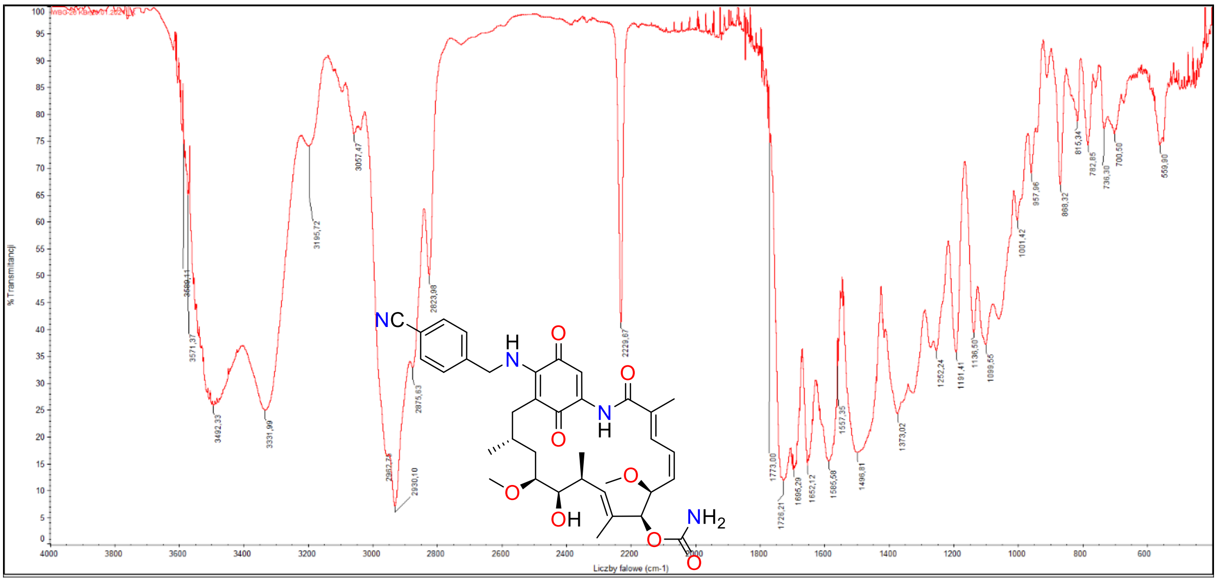

Figure S38 FT-IR spectrum of compound **8** (in KBr).

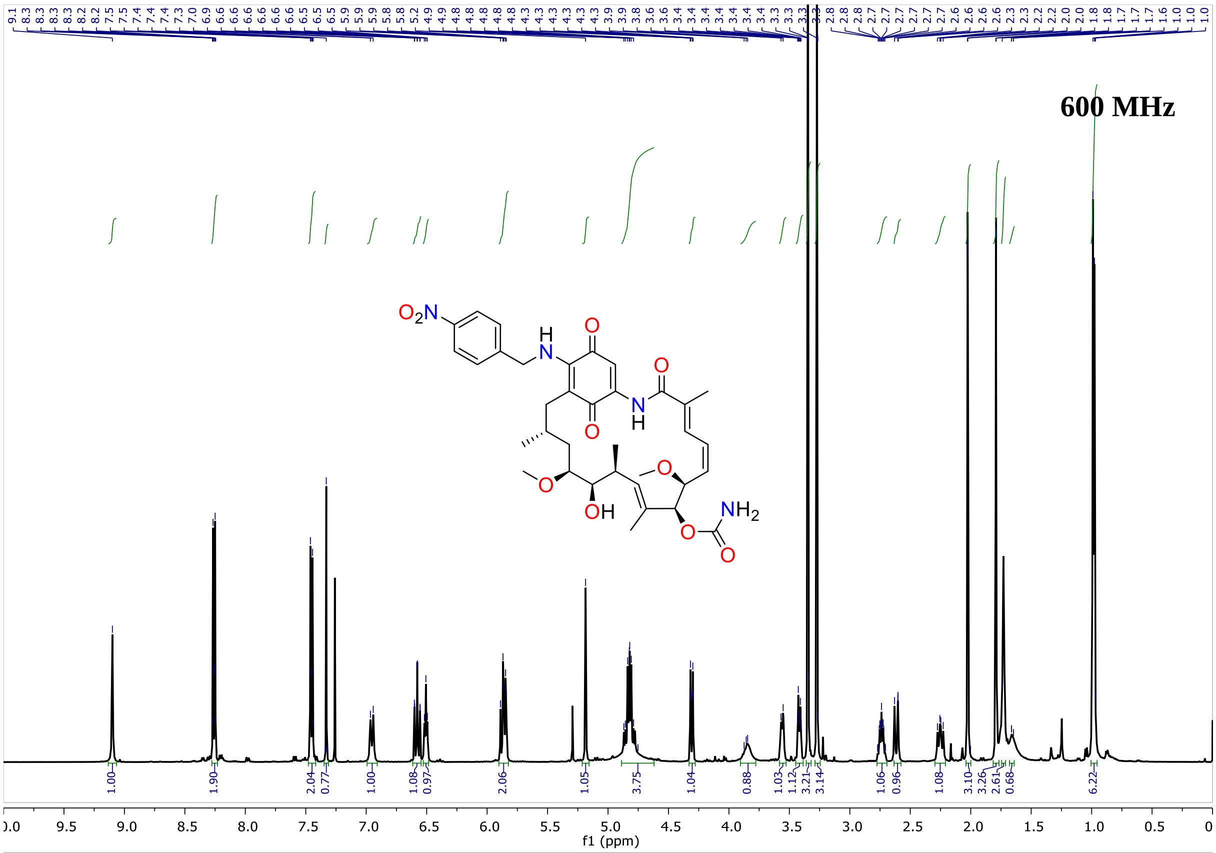

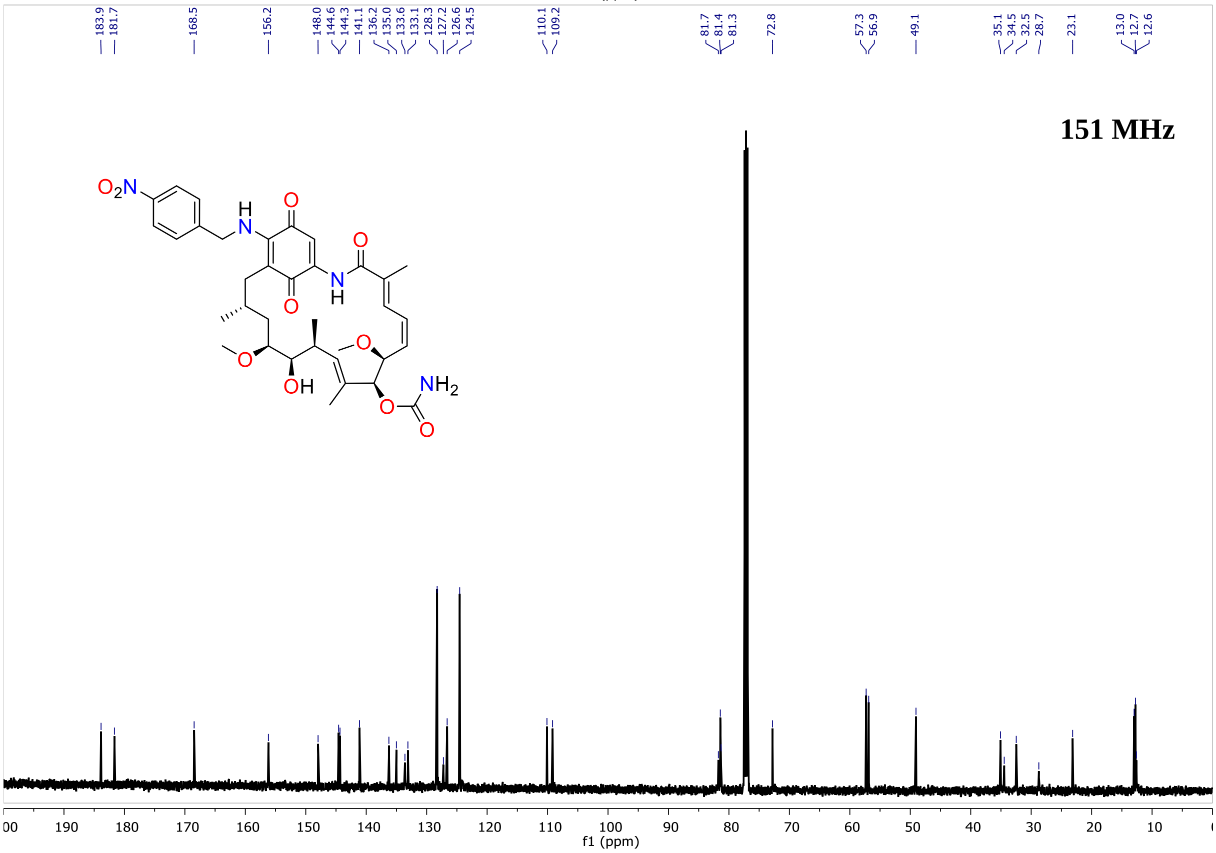

Figure S39 ^{1}H and ^{13}C{1H} spectra of compound **9** in $CDCl_3$.

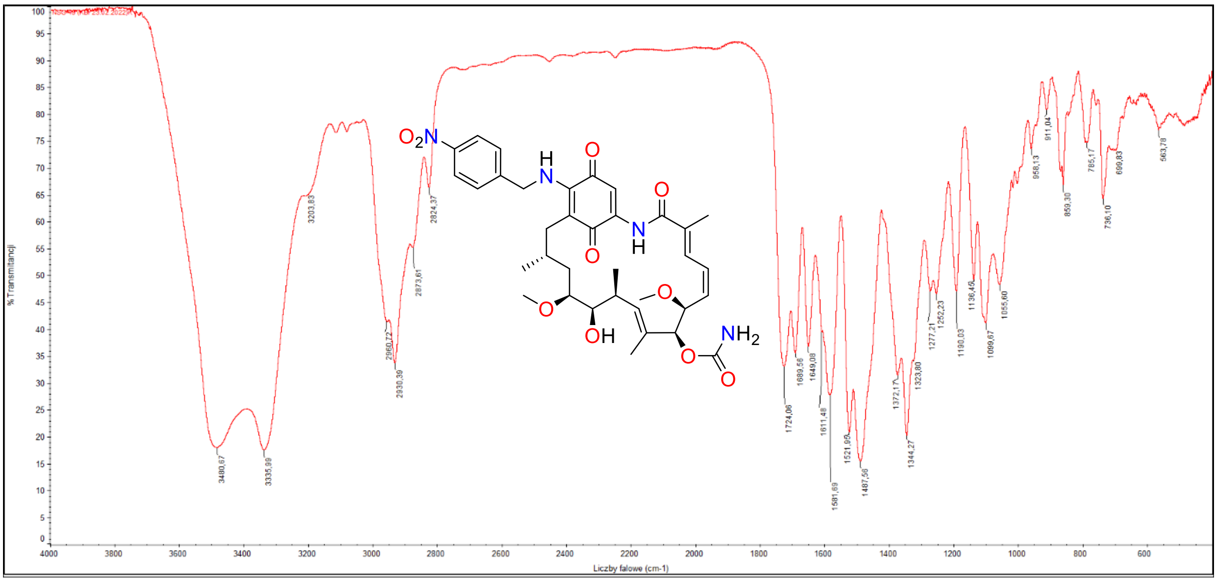

Figure S40 FT-IR spectrum of compound **9** (in KBr).

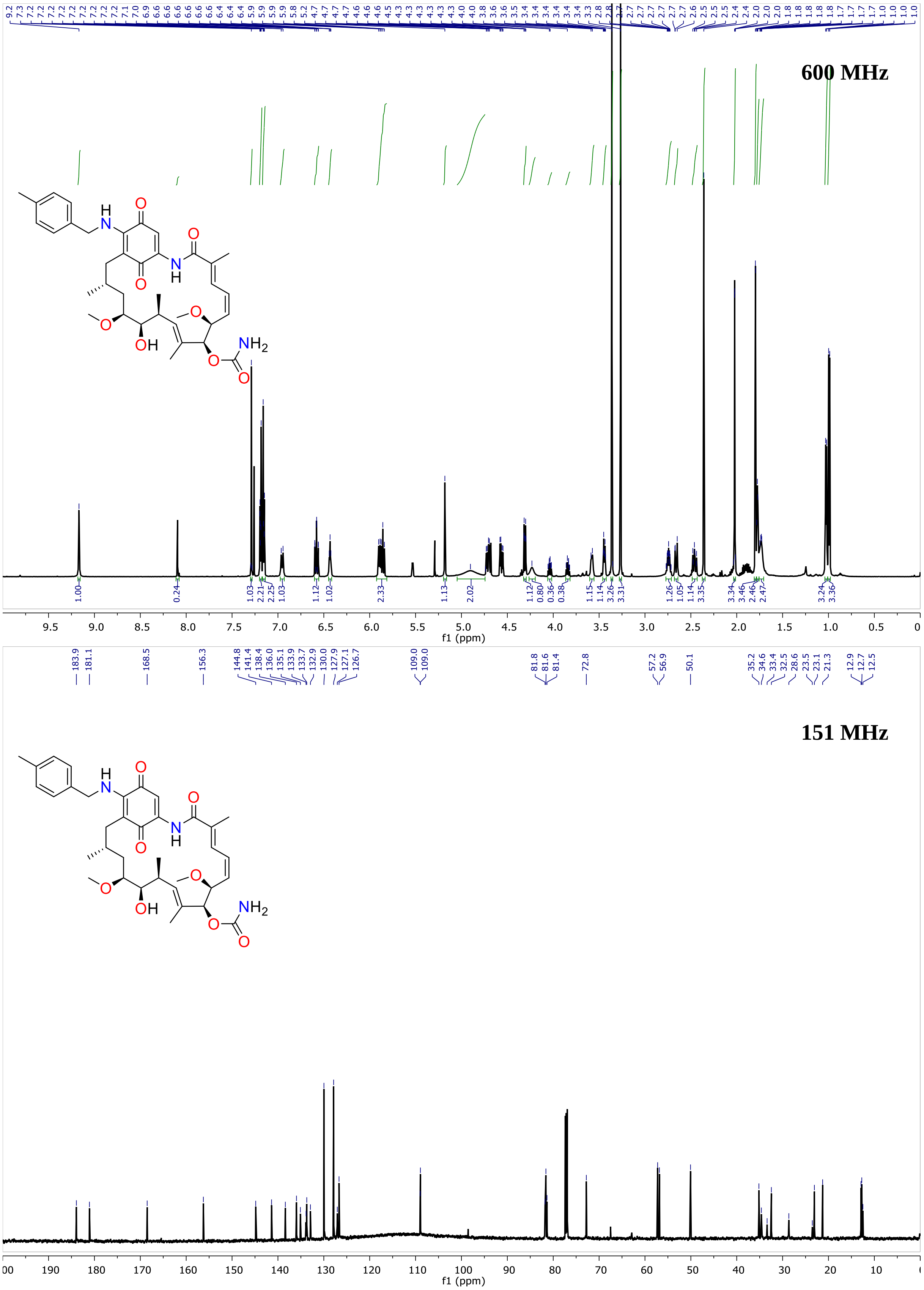

Figure S41 ^{1}H and ^{13}C{1H} spectra of compound **10** in $CDCl_3$.

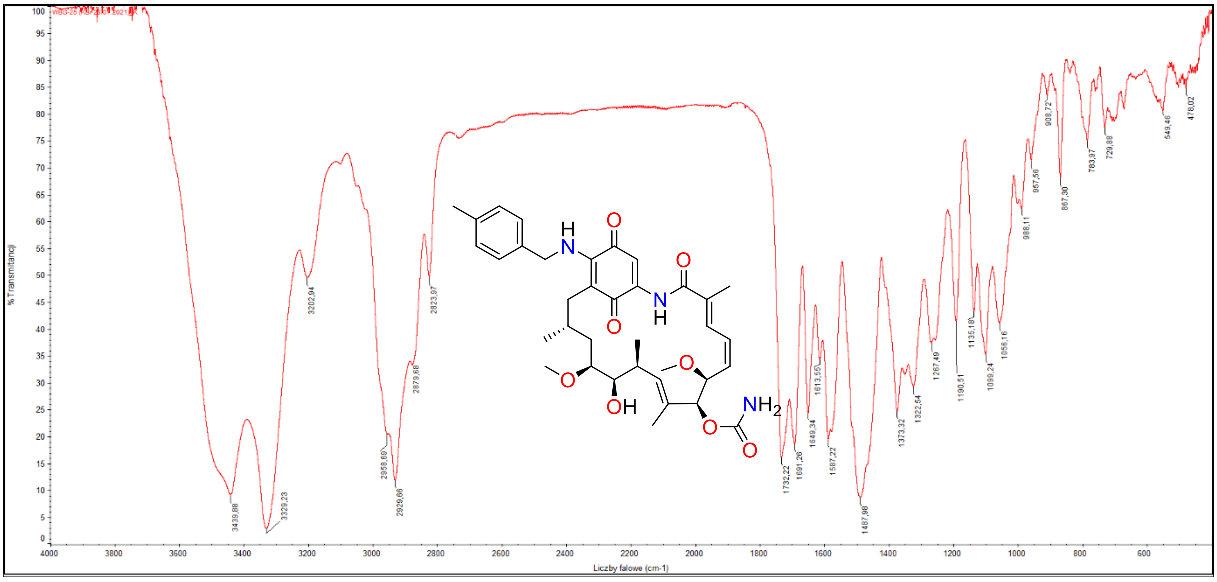

Figure S42 FT-IR spectrum of compound **10** (in KBr).

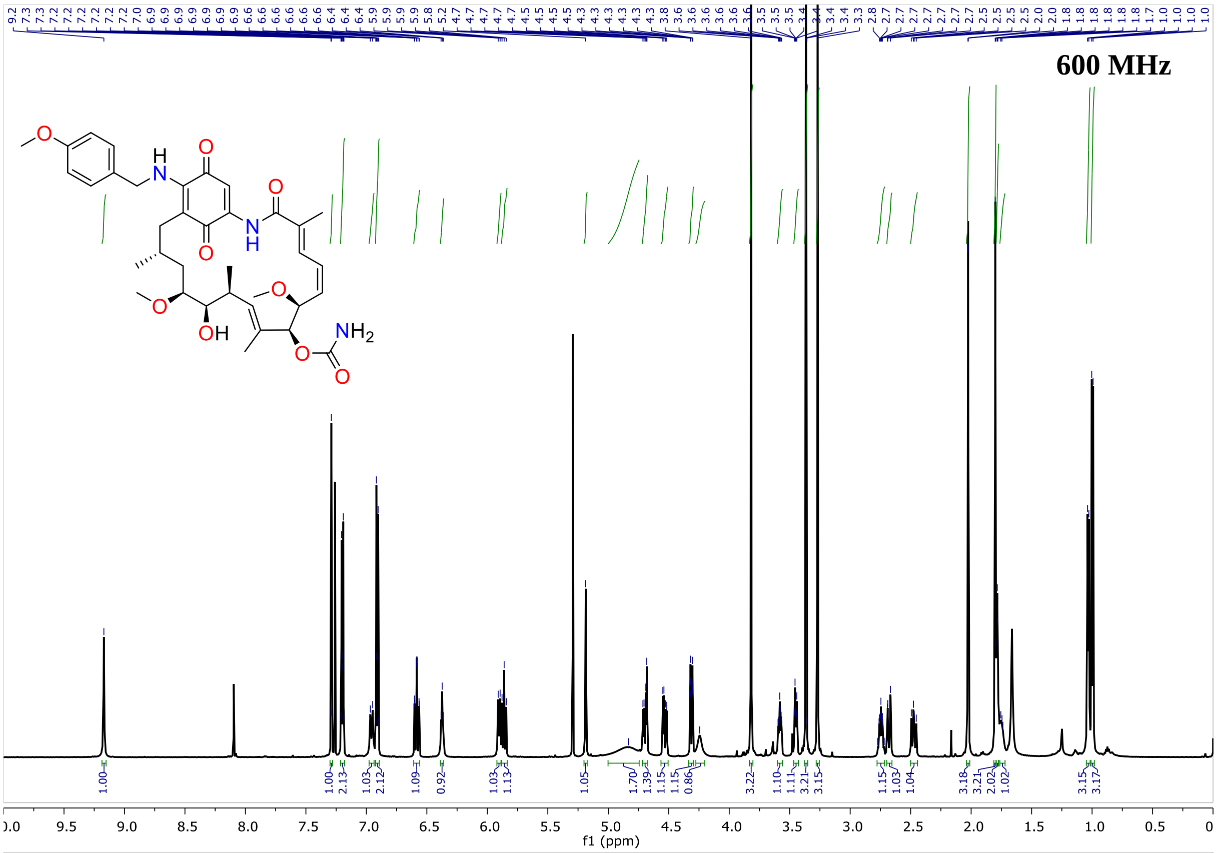

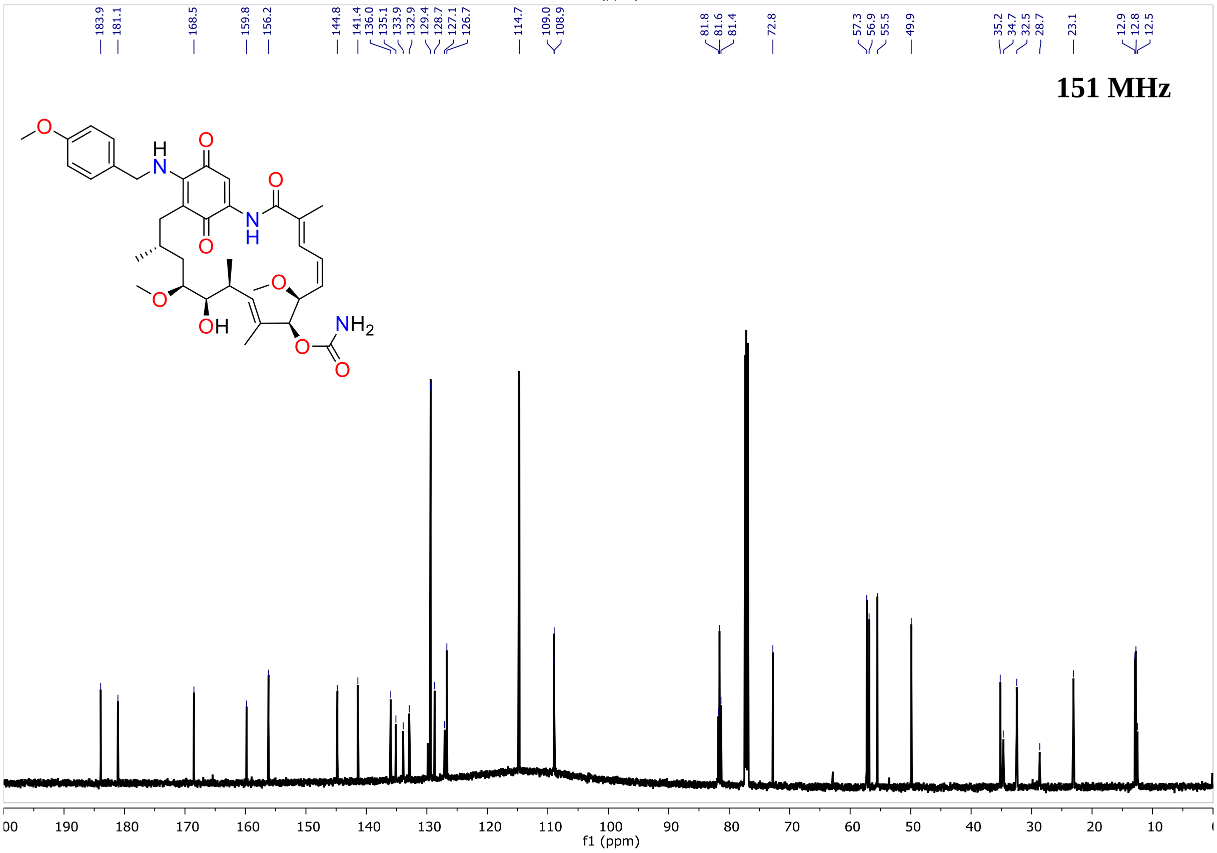

Figure S43 ^{1}H and ^{13}C{1H} spectra of compound **11** in $CDCl_3$.

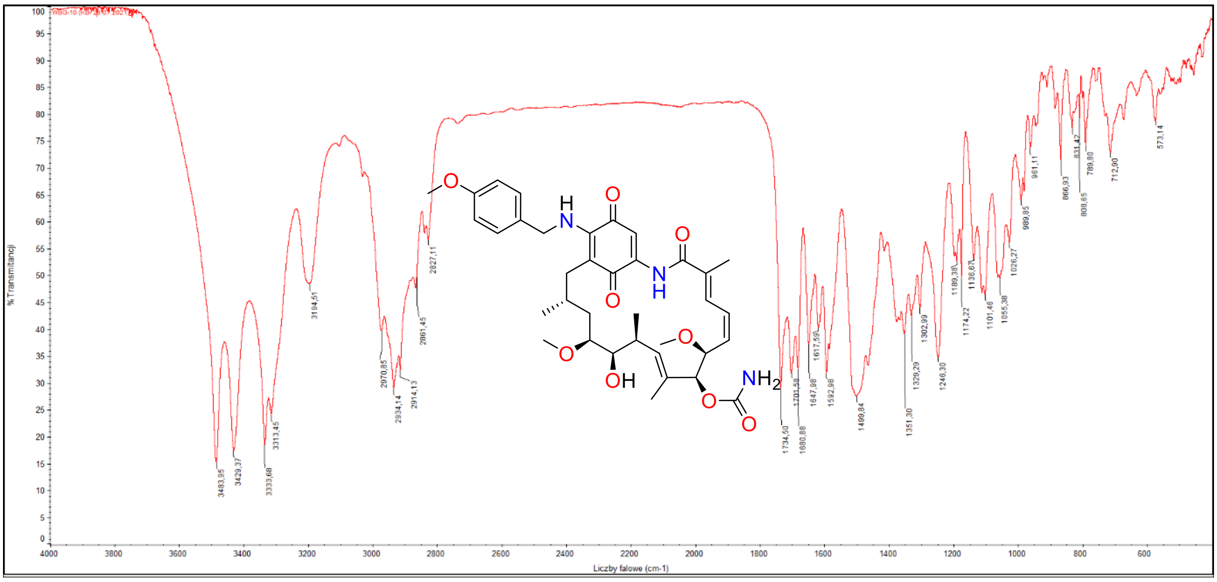

Figure S44 FT-IR spectrum of compound **11** (in KBr).

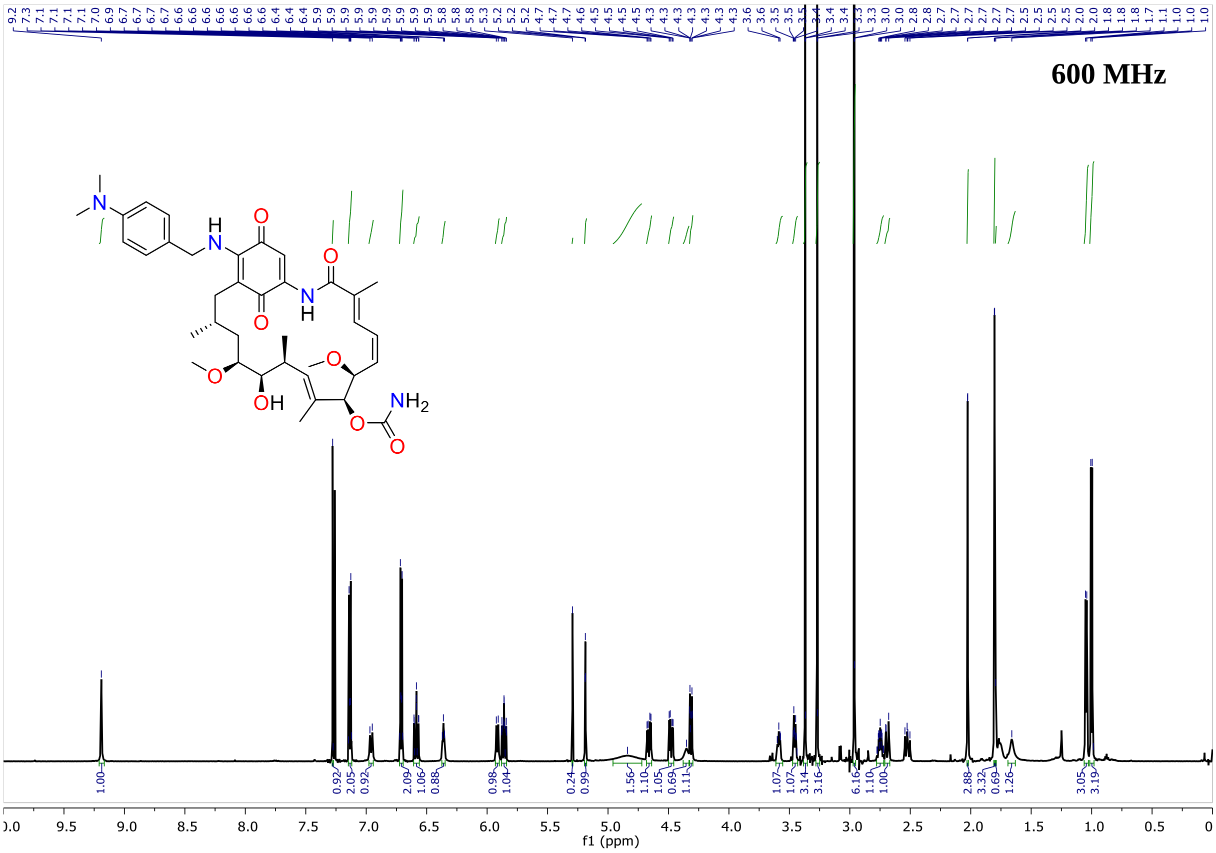

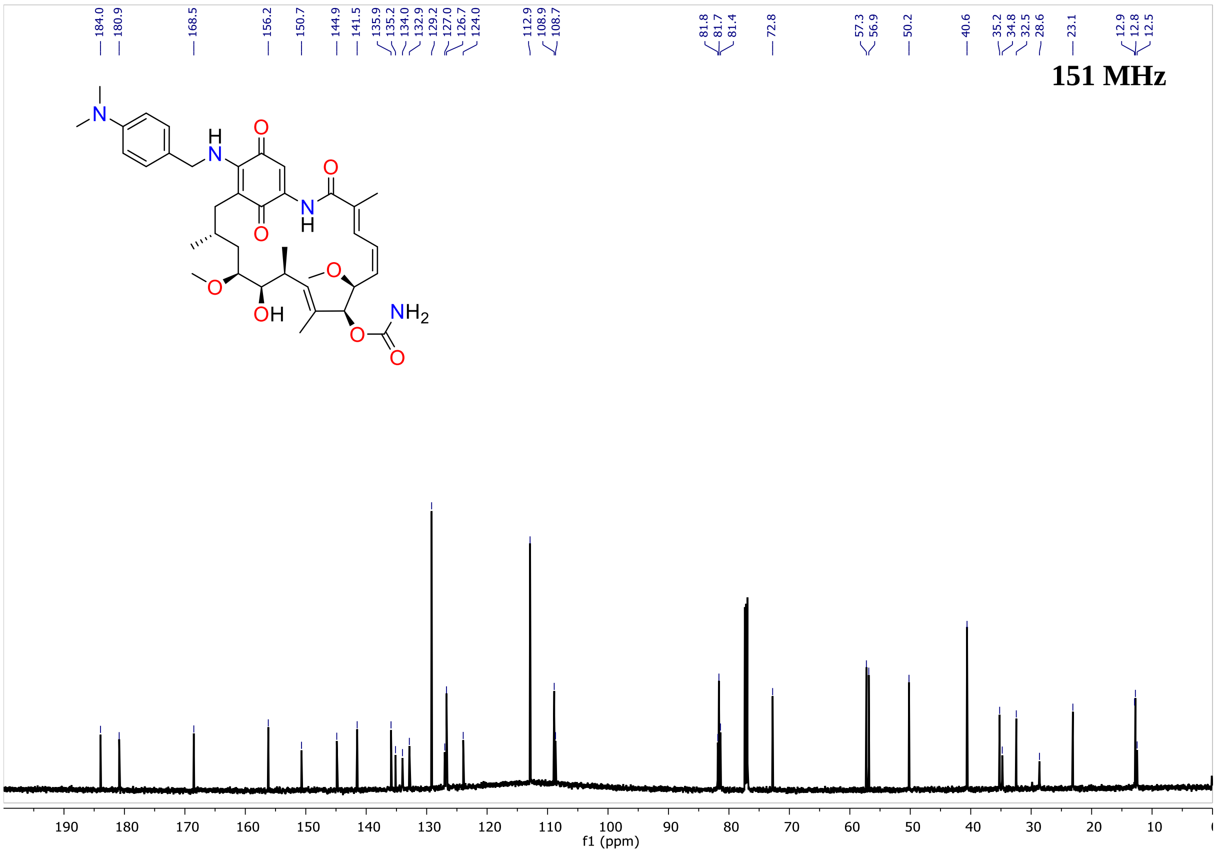

Figure S45 ^{1}H and ^{13}C{1H} spectra of compound **12** in $CDCl_3$.

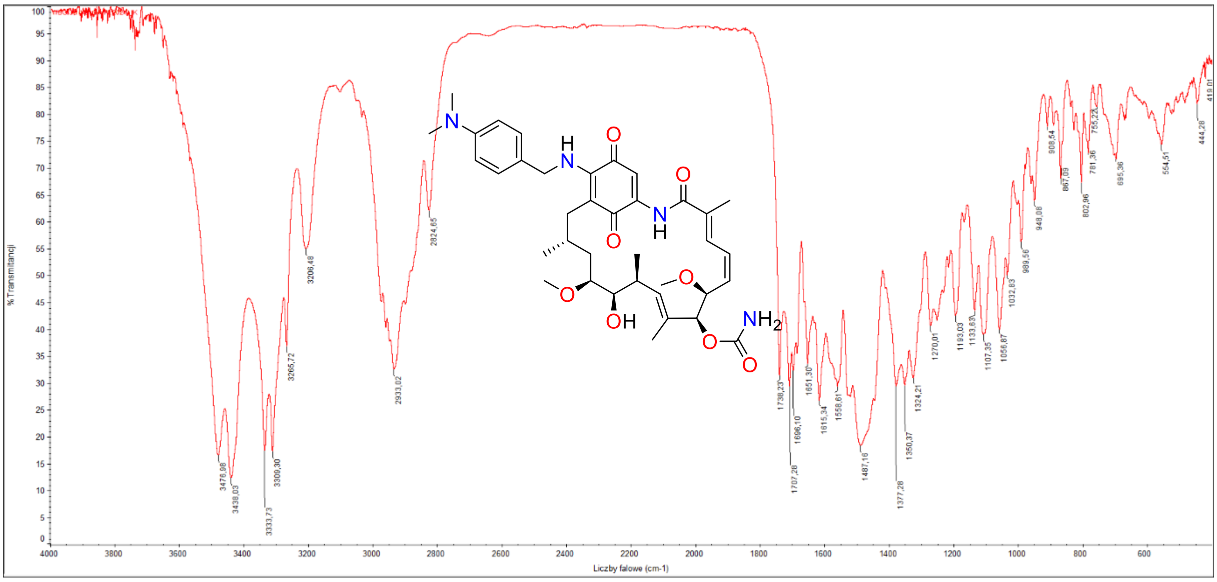

Figure S46 FT-IR spectrum of compound **12** (in KBr).

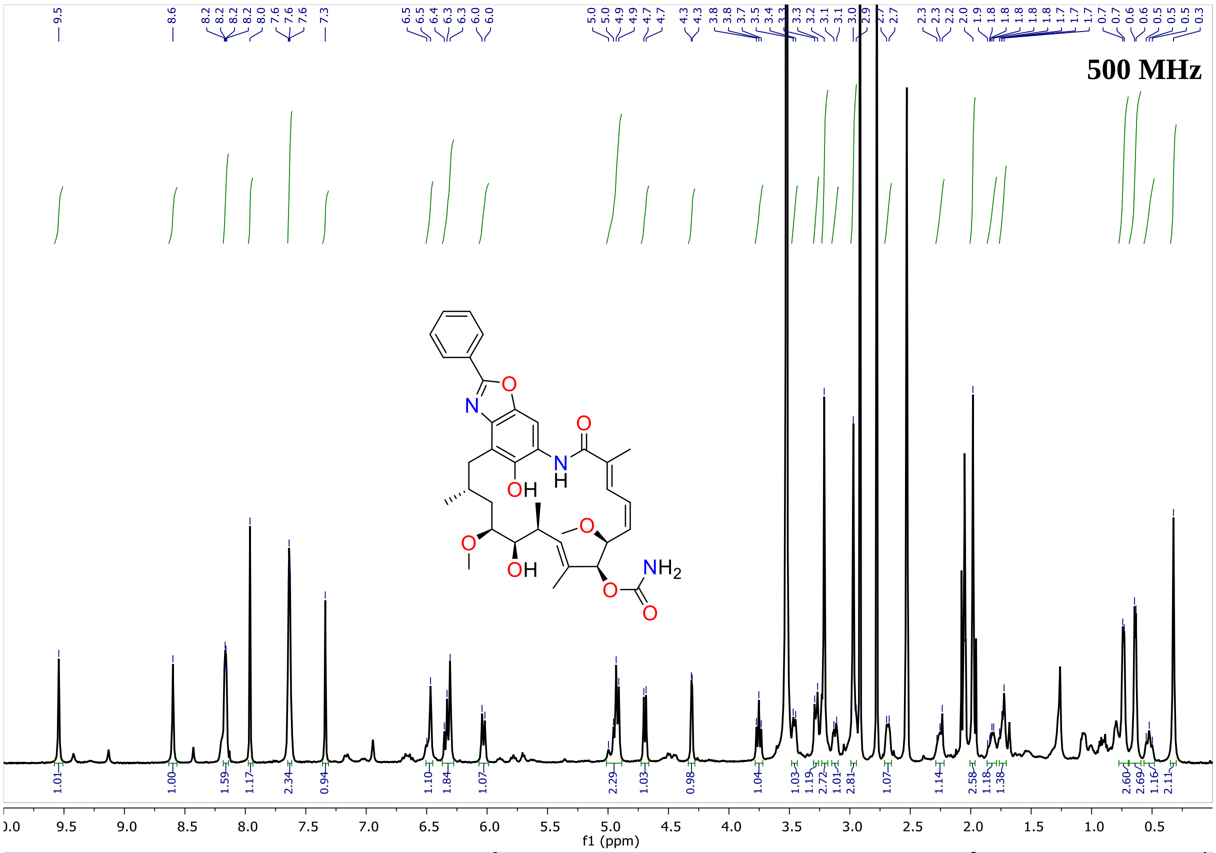

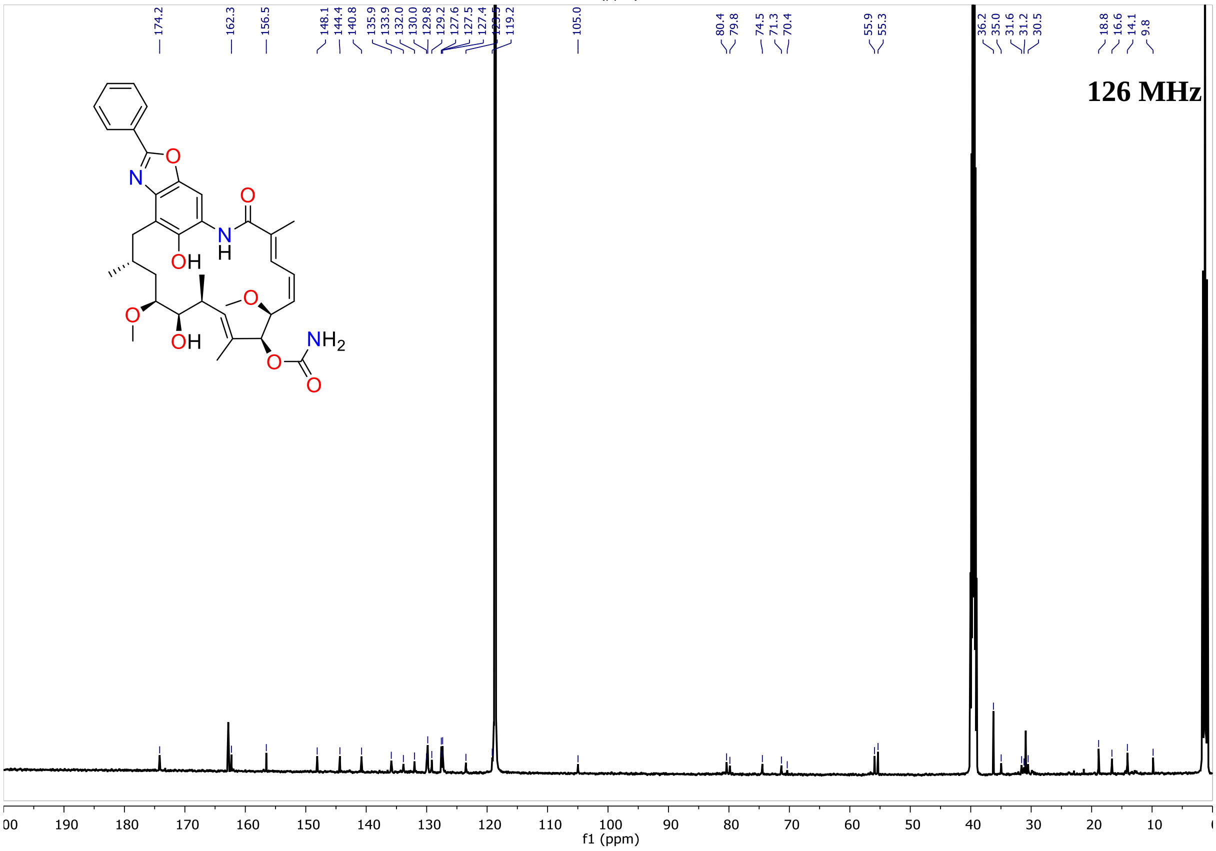

Figure S47 ^{1}H and ^{13}C{1H} spectra of compound **1a** in DMSO-d_6+ ACN-d$_3$.

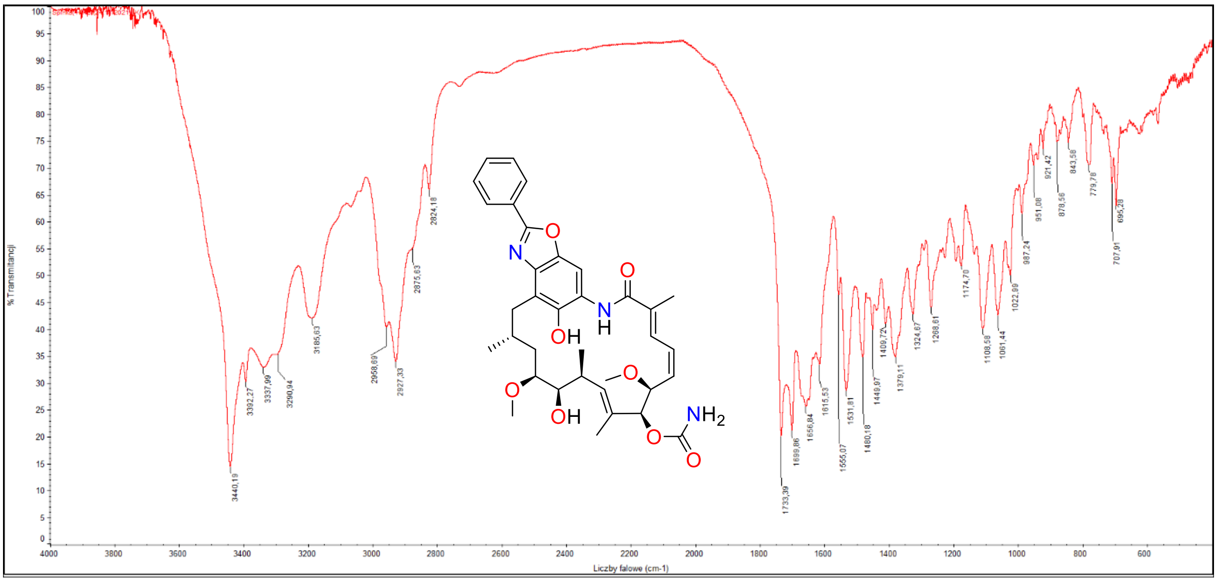

Figure S48 FT-IR spectrum of compound **1a** (in KBr).

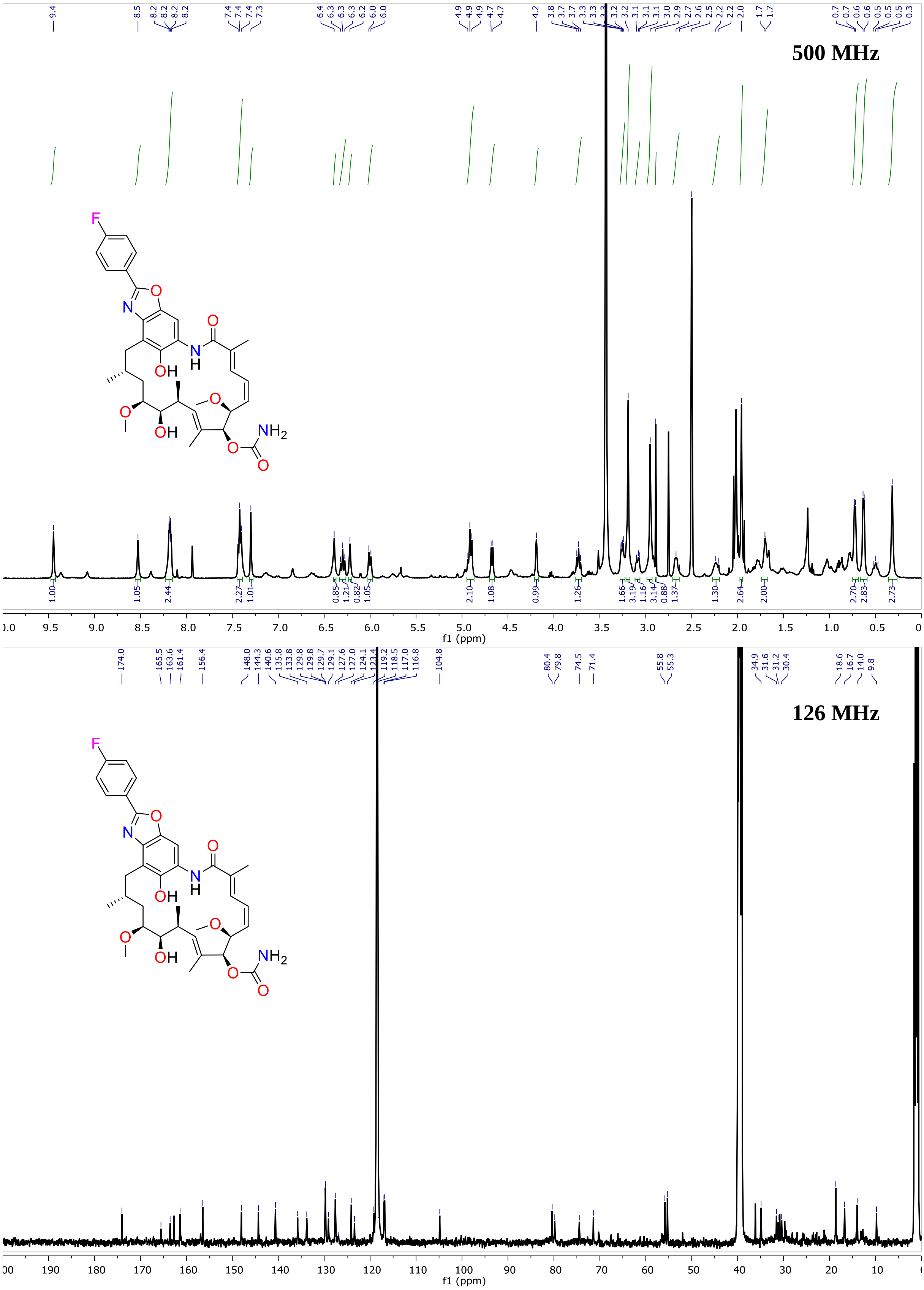

Figure S49 ^{1}H and ^{13}C{1H} spectra of compound **2a** in DMSO-d_6+ ACN-d$_3$.

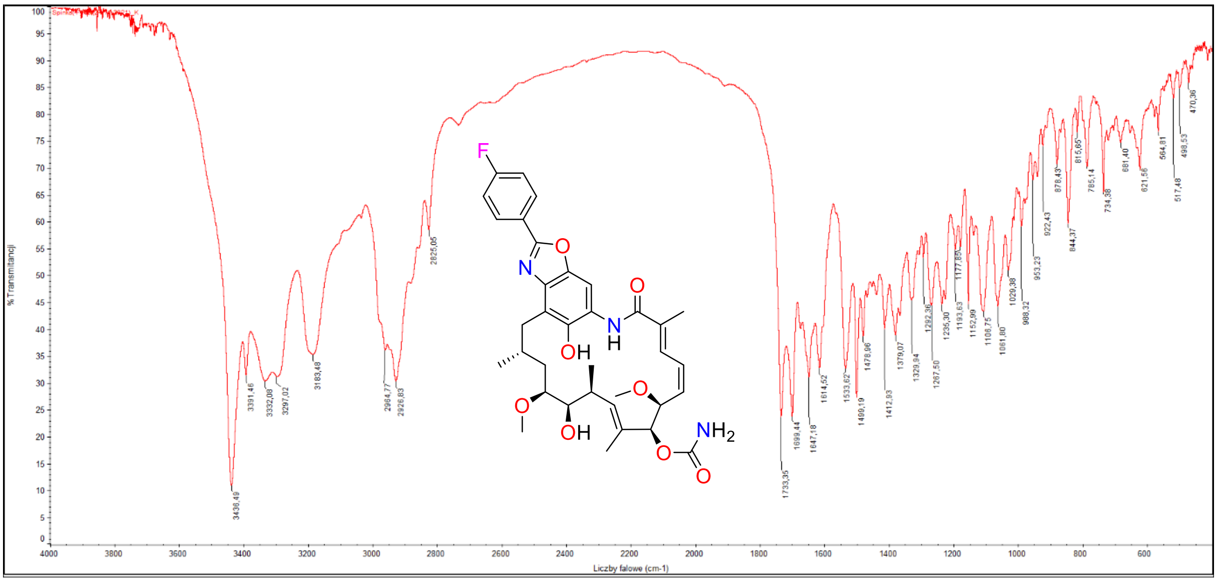

Figure S50 FT-IR spectrum of compound **2a** (in KBr).

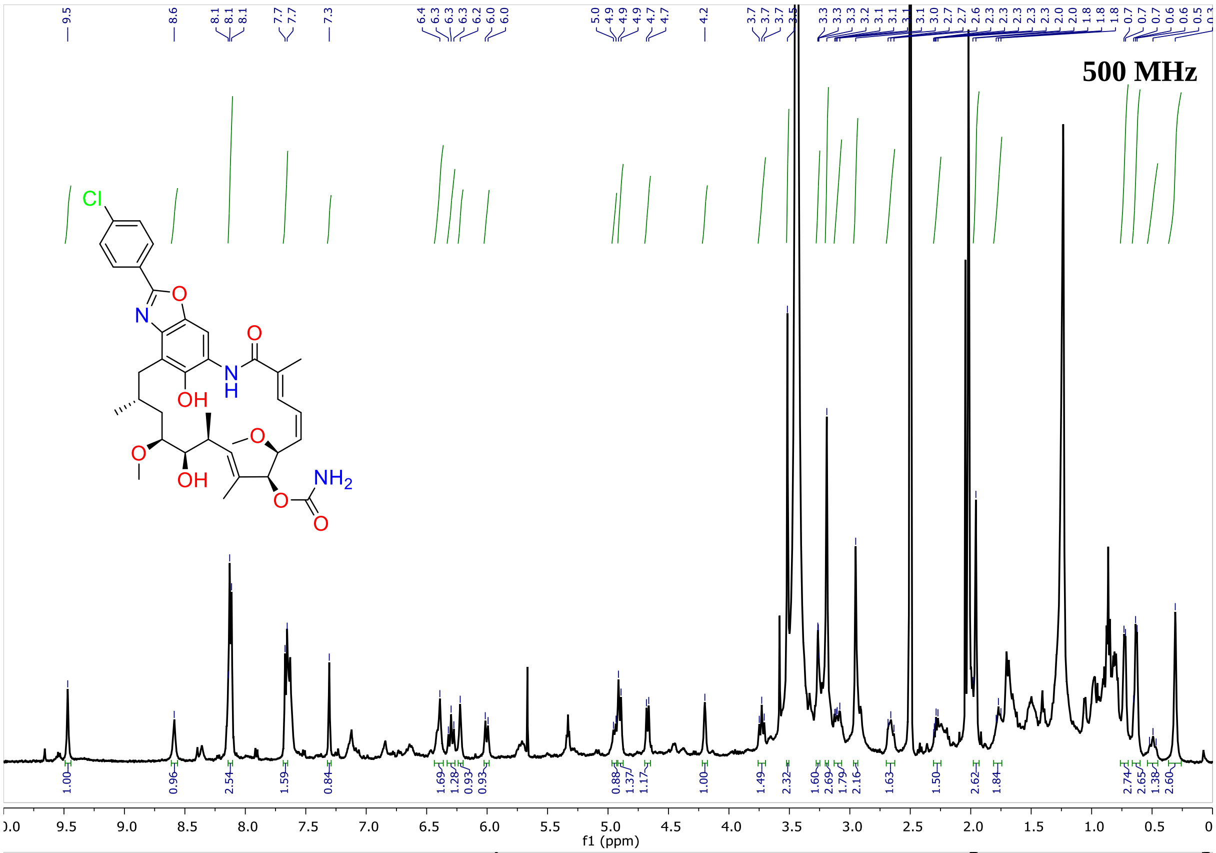

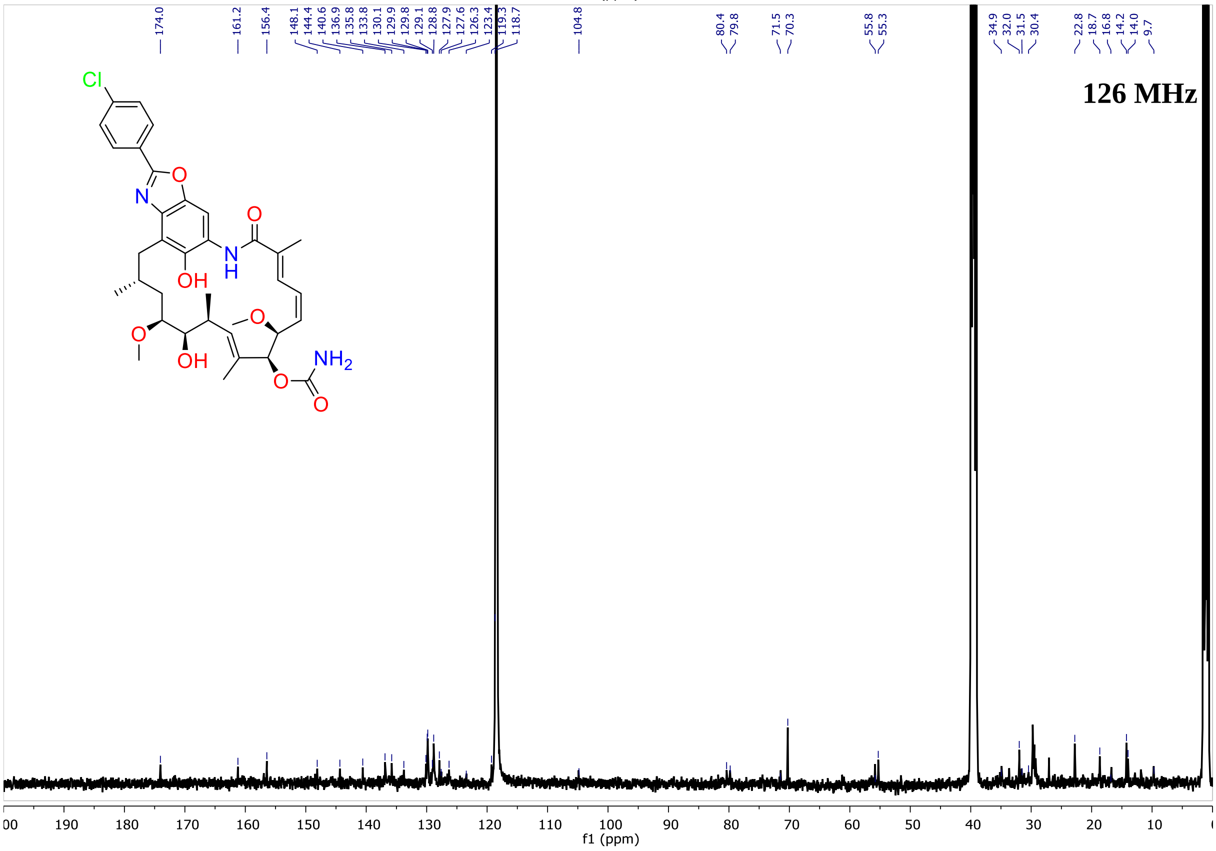

Figure S51 ^{1}H and $^{13}C\{1H\}$ spectra of compound **3a** in DMSO-d_6+ ACN-d_3.

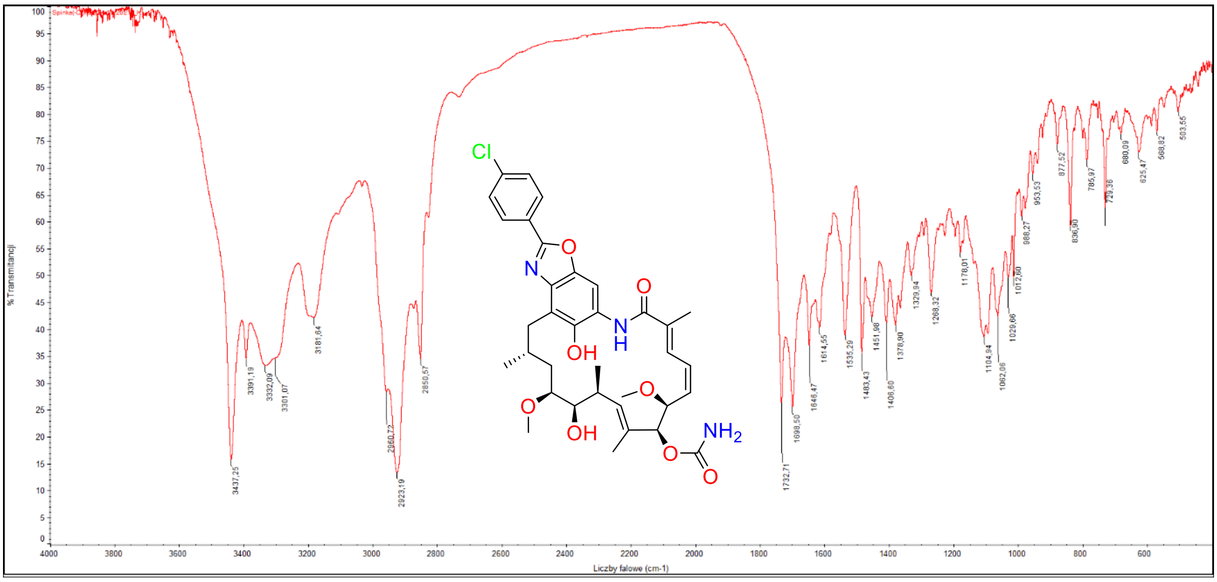

Figure S52 FT-IR spectrum of compound **3a** (in KBr).

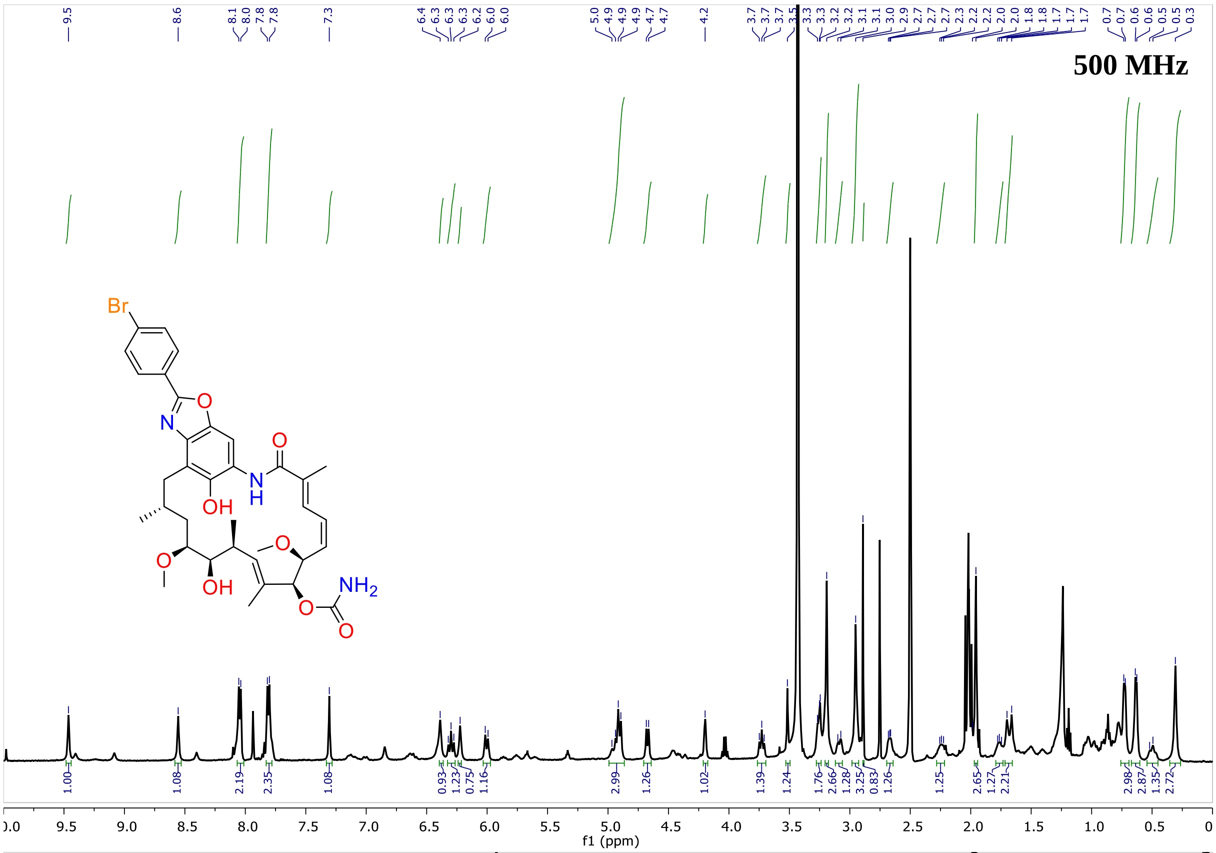

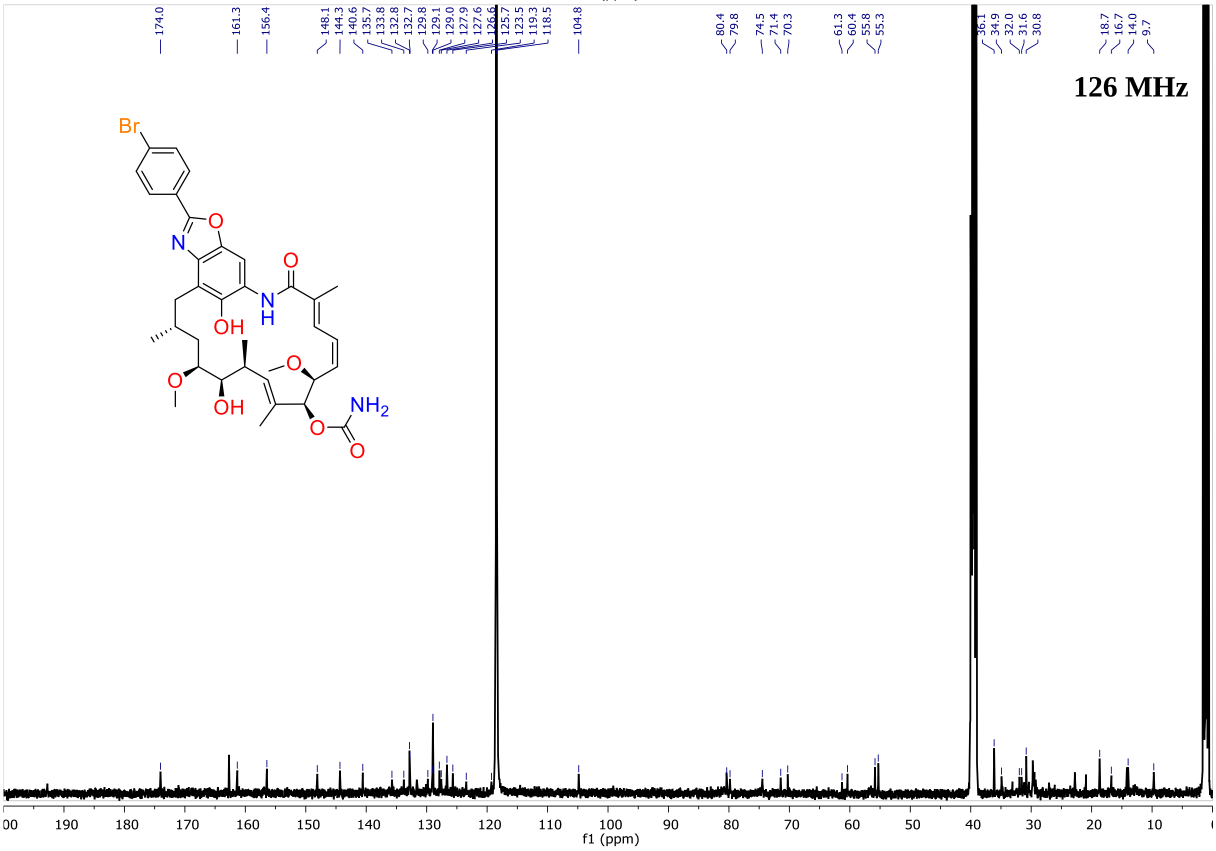

Figure S53 ^{1}H and ^{13}C{1H} spectra of compound **4a** in DMSO-d_6+ ACN-d$_3$.

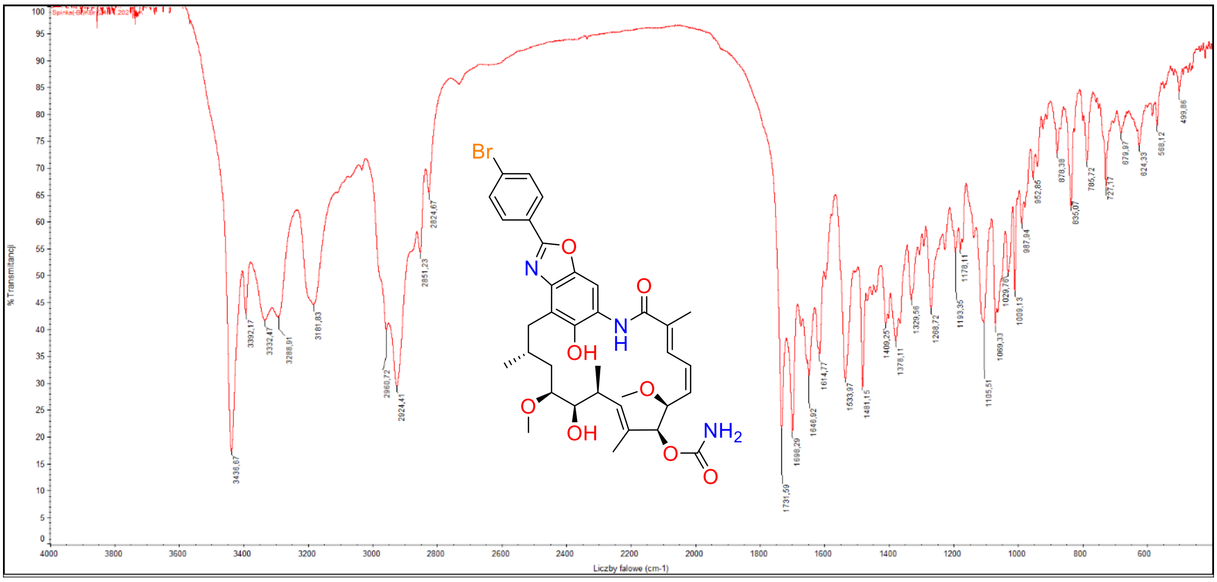

Figure S54 FT-IR spectrum of compound **4a** (in KBr).

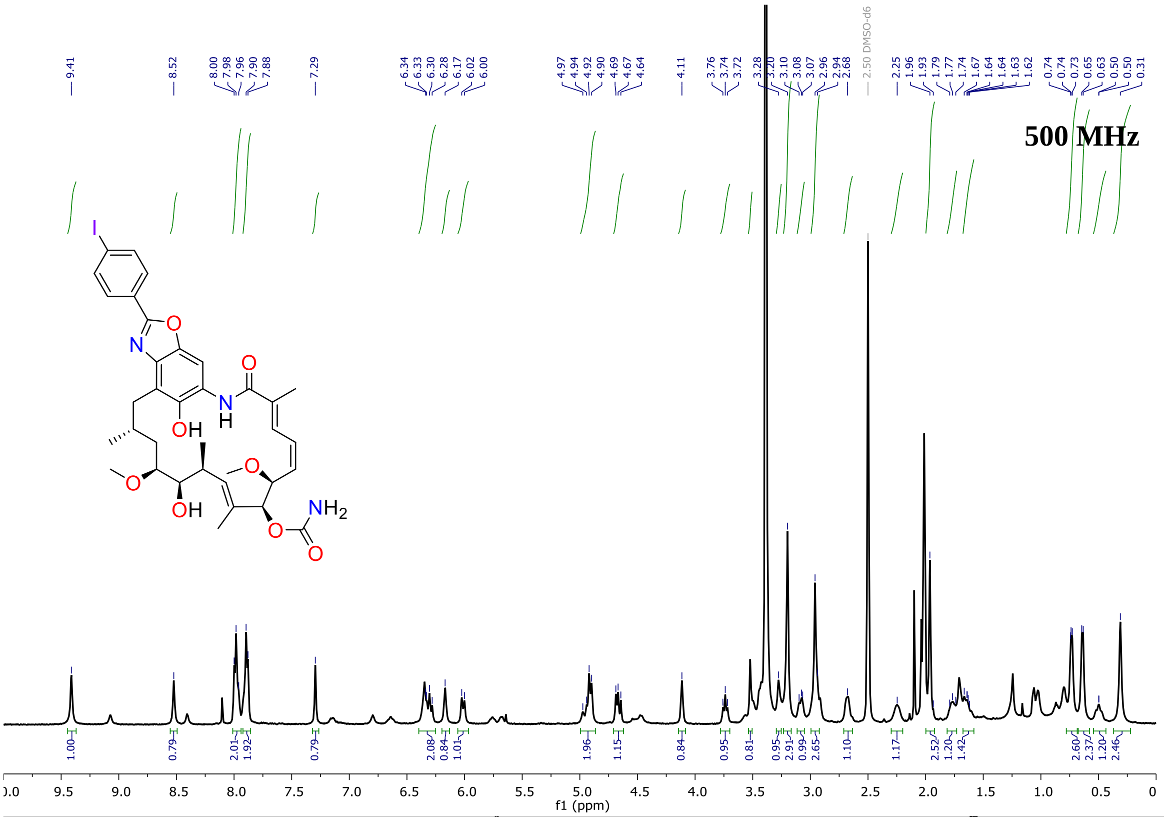

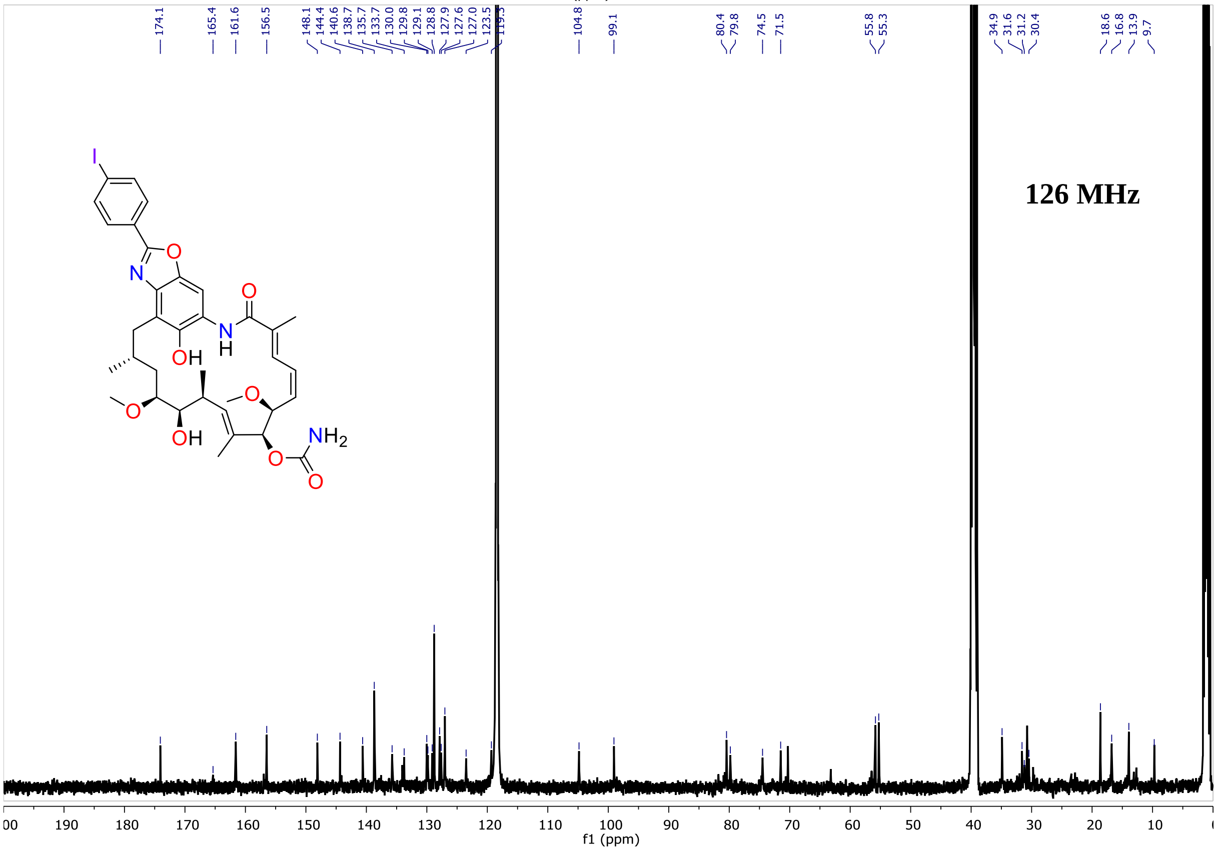

Figure S55 ^{1}H and $^{13}C\{1H\}$ spectra of compound **5a** in DMSO-d_6+ ACN-d_3.

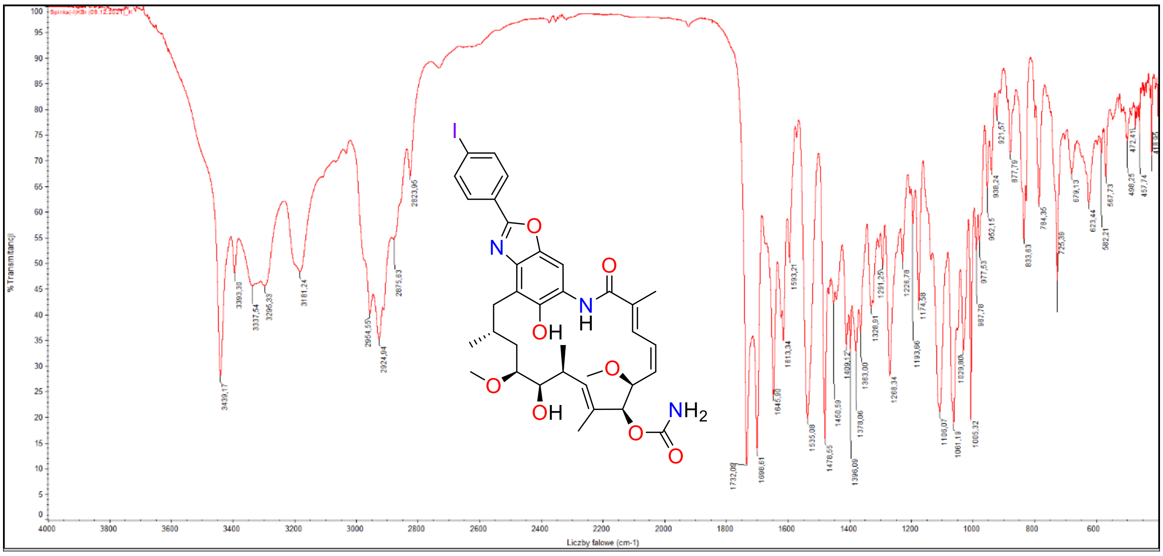

Figure S56 FT-IR spectrum of compound **5a** (in KBr).

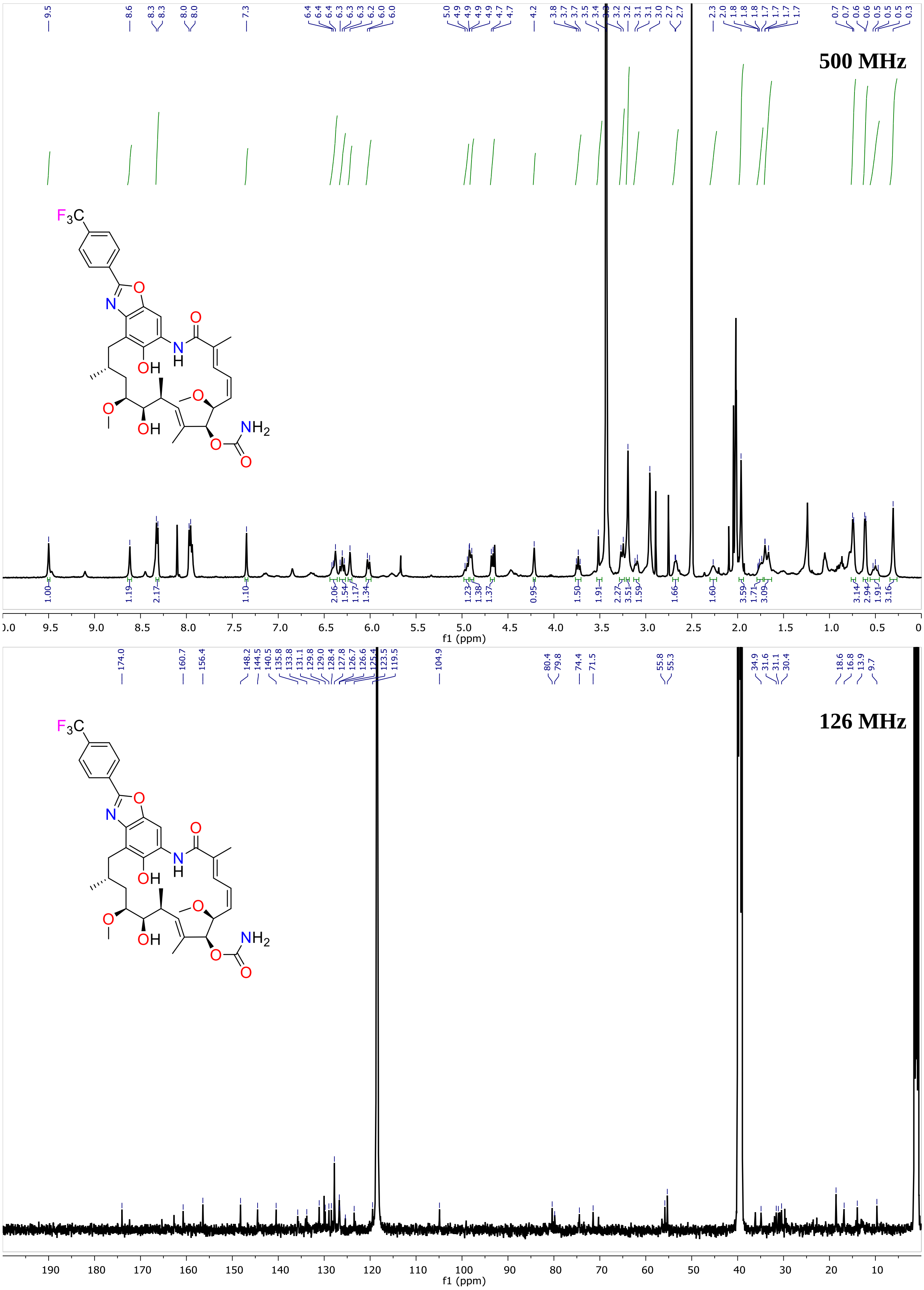

Figure S57 ^{1}H and ^{13}C{1H} spectra of compound **6a** in DMSO-d_6+ ACN-d$_3$.

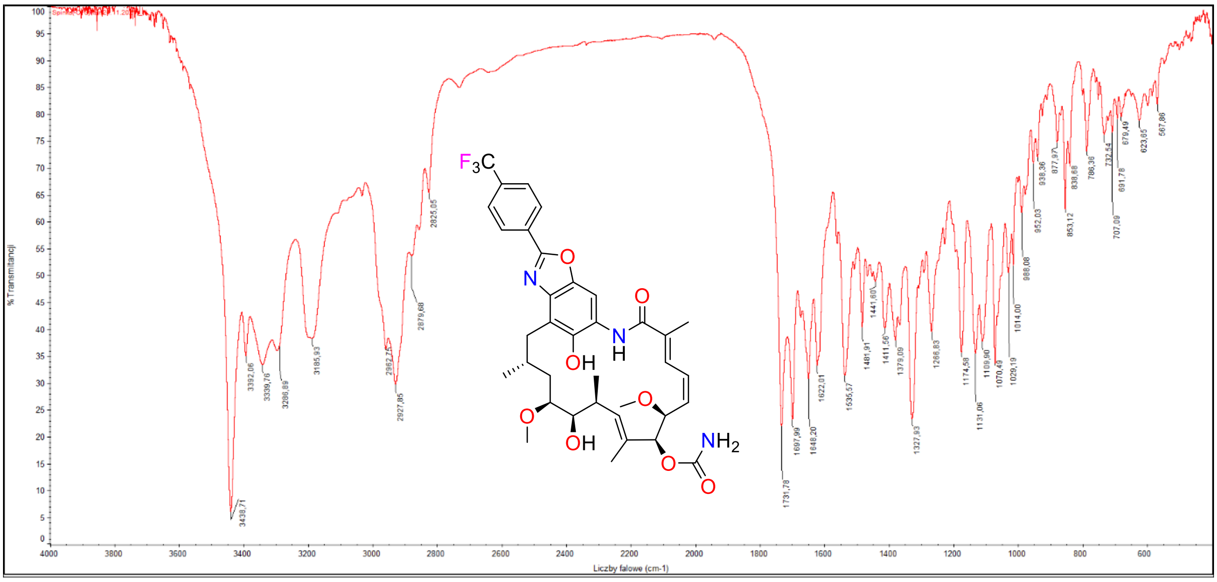

Figure S58 FT-IR spectrum of compound **6a** (in KBr).

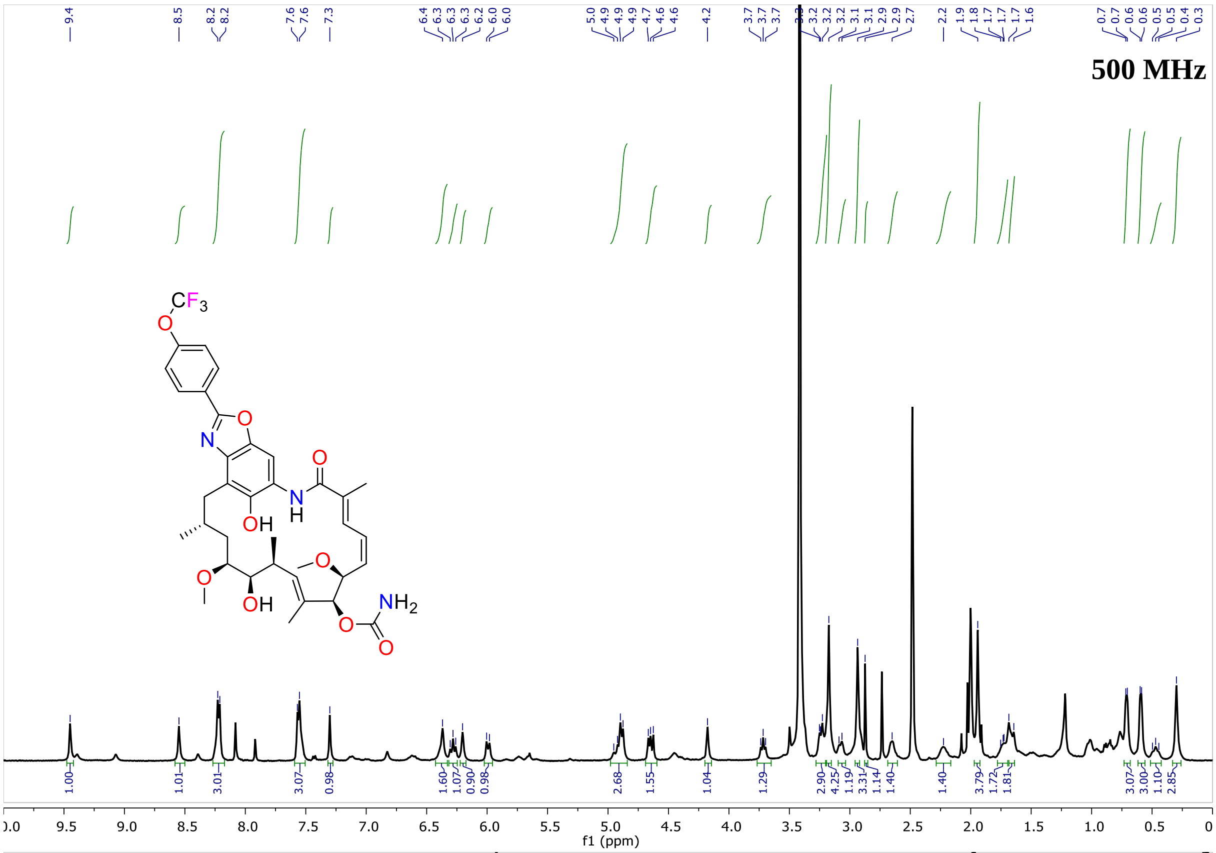

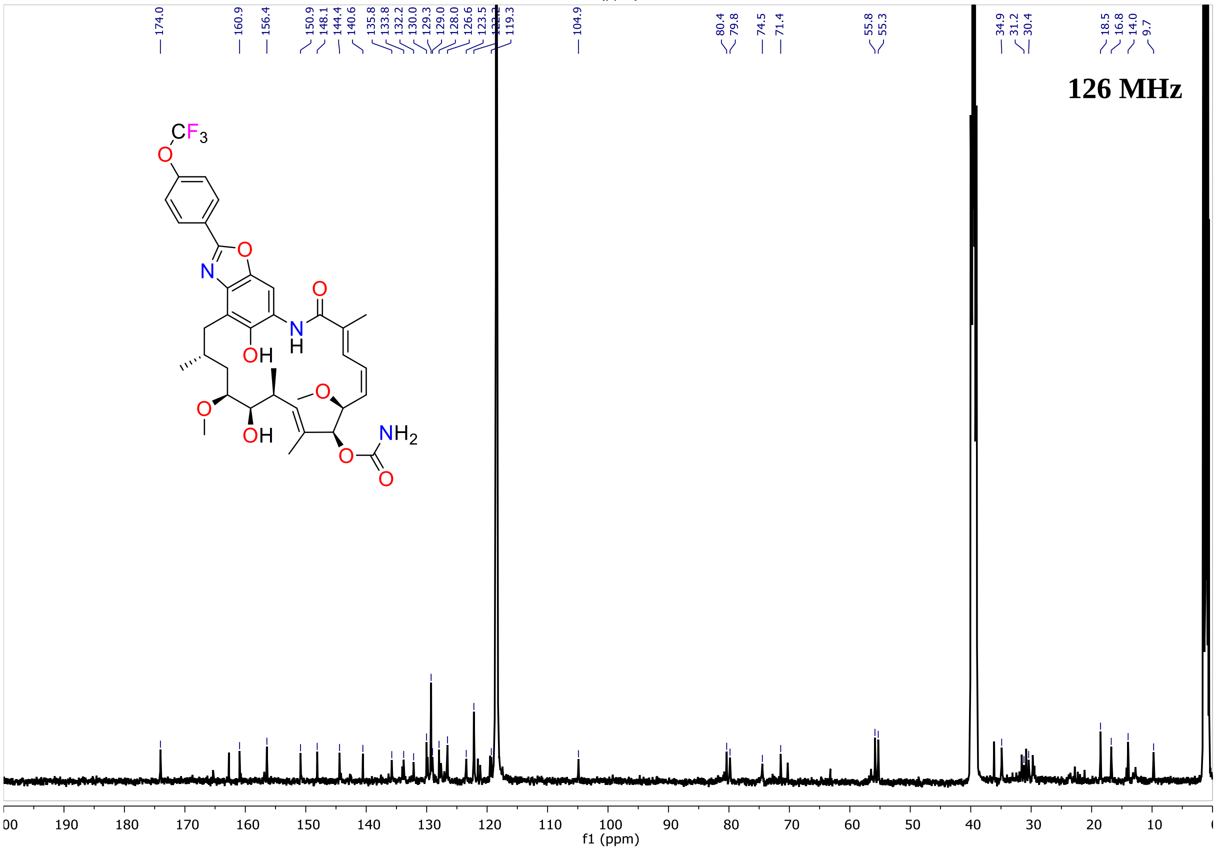

Figure S59 ¹H and ¹³C{1H} spectra of compound **7a** in DMSO-d_6+ ACN-d$_3$.

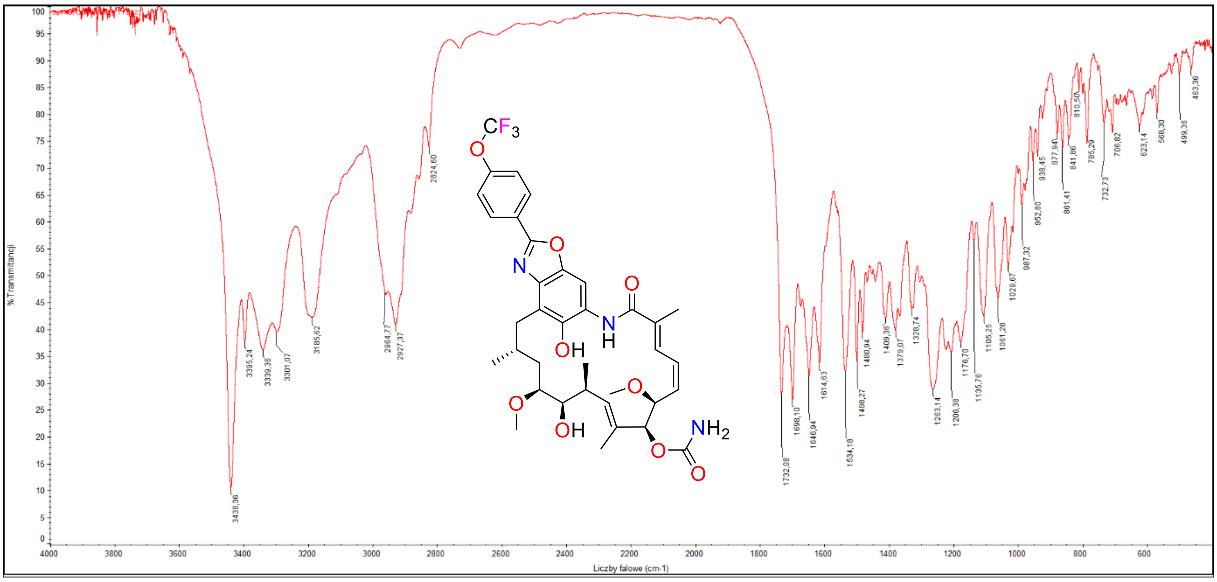

Figure S60 FT-IR spectrum of compound **7a** (in KBr).

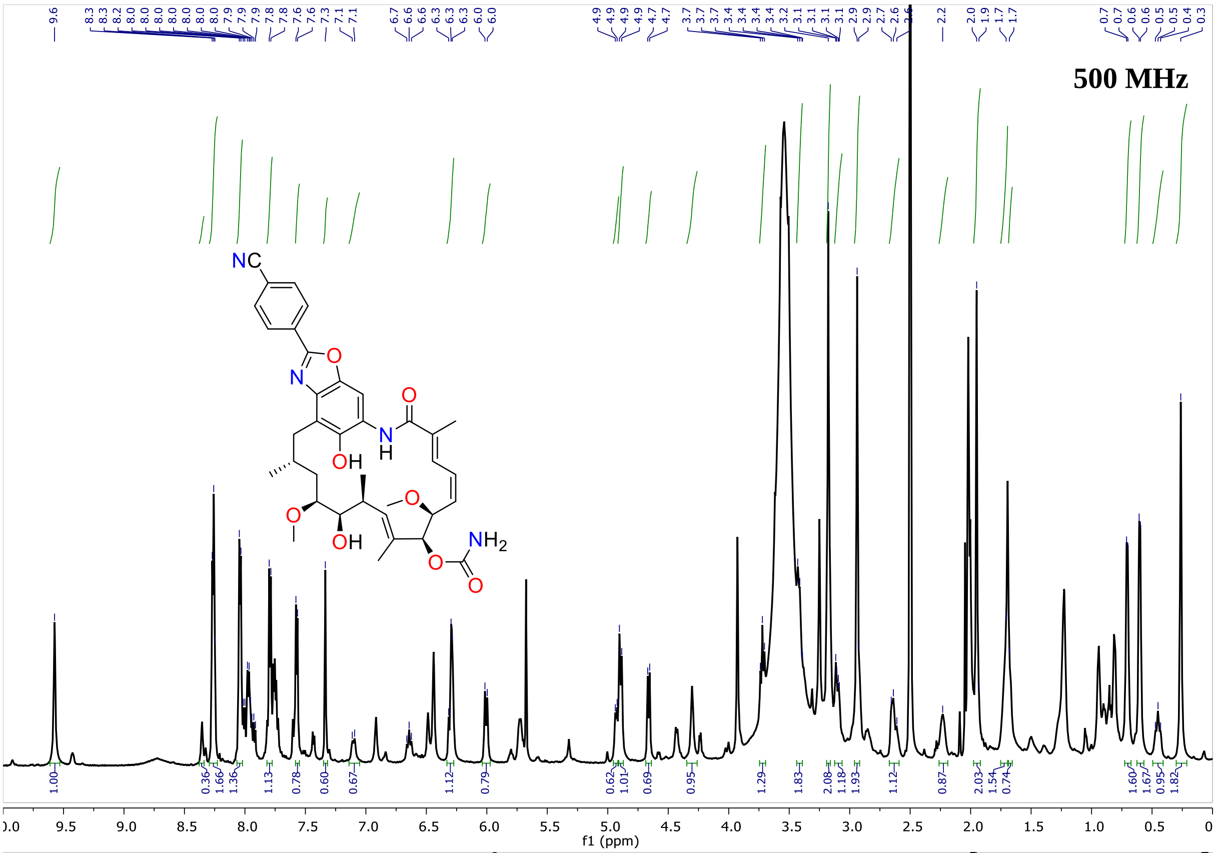

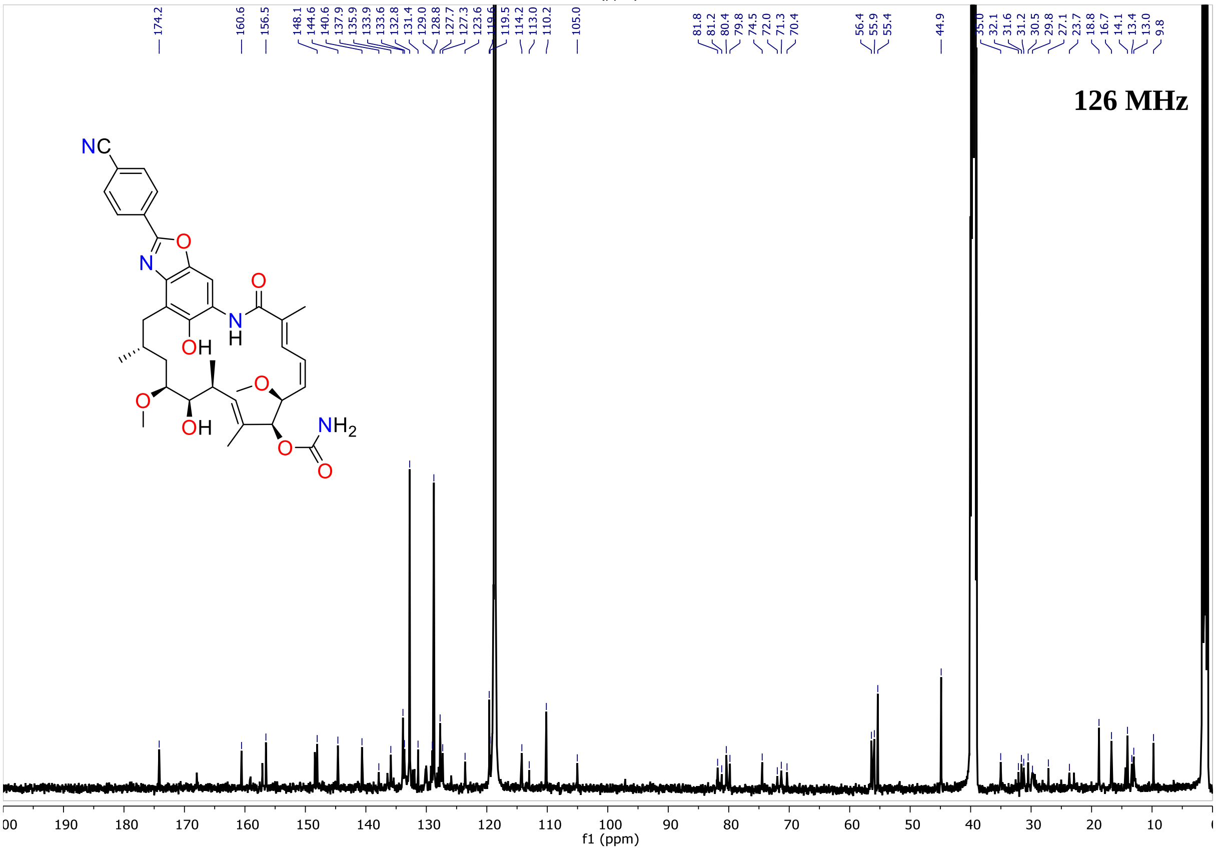

Figure S61 ^{1}H and ^{13}C{1H} spectra of compound **8a** in DMSO-d_6+ ACN-d_3.

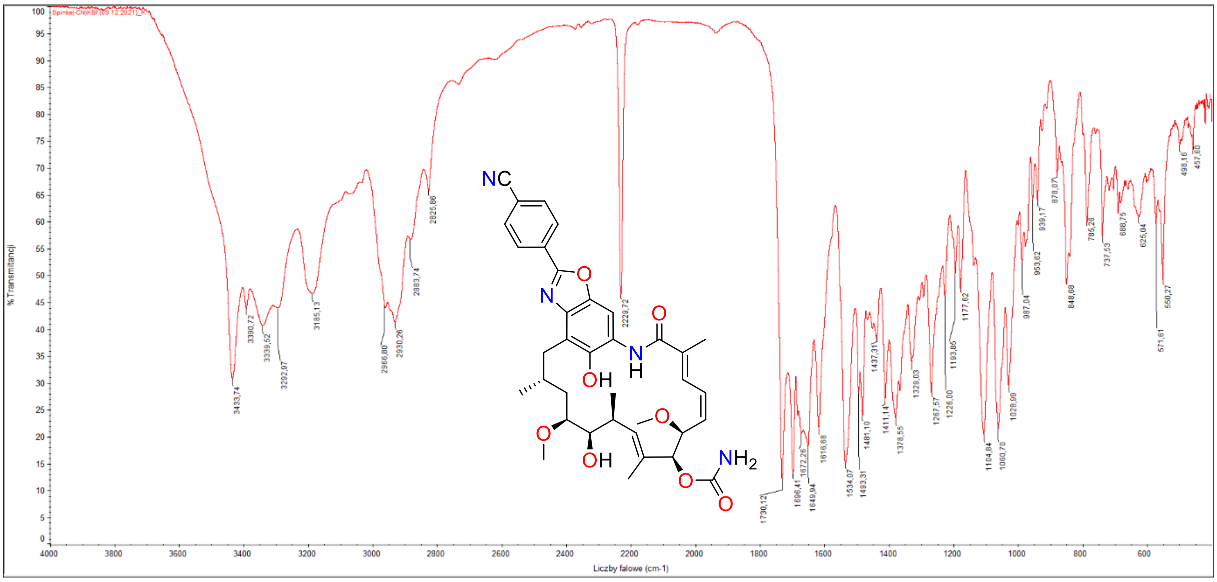

Figure S62 FT-IR spectrum of compound **8a** (in KBr).

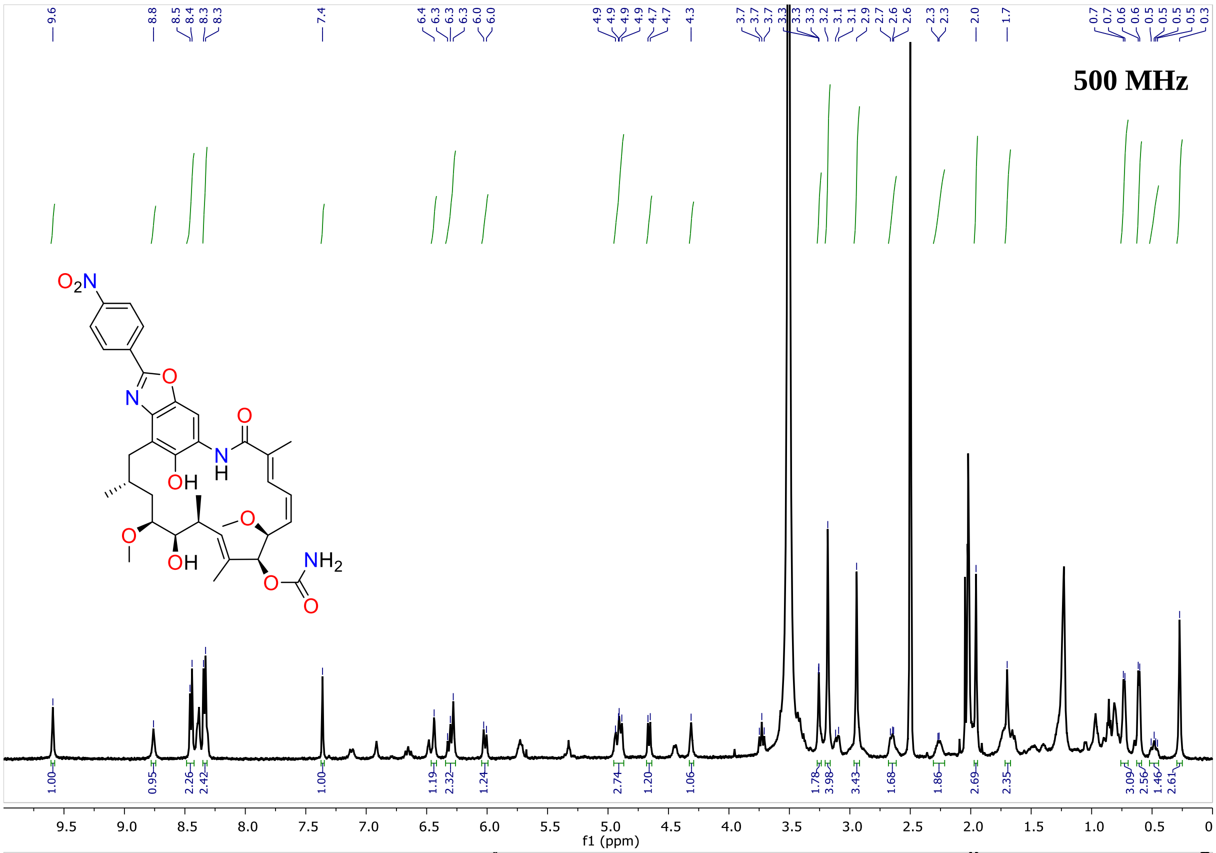

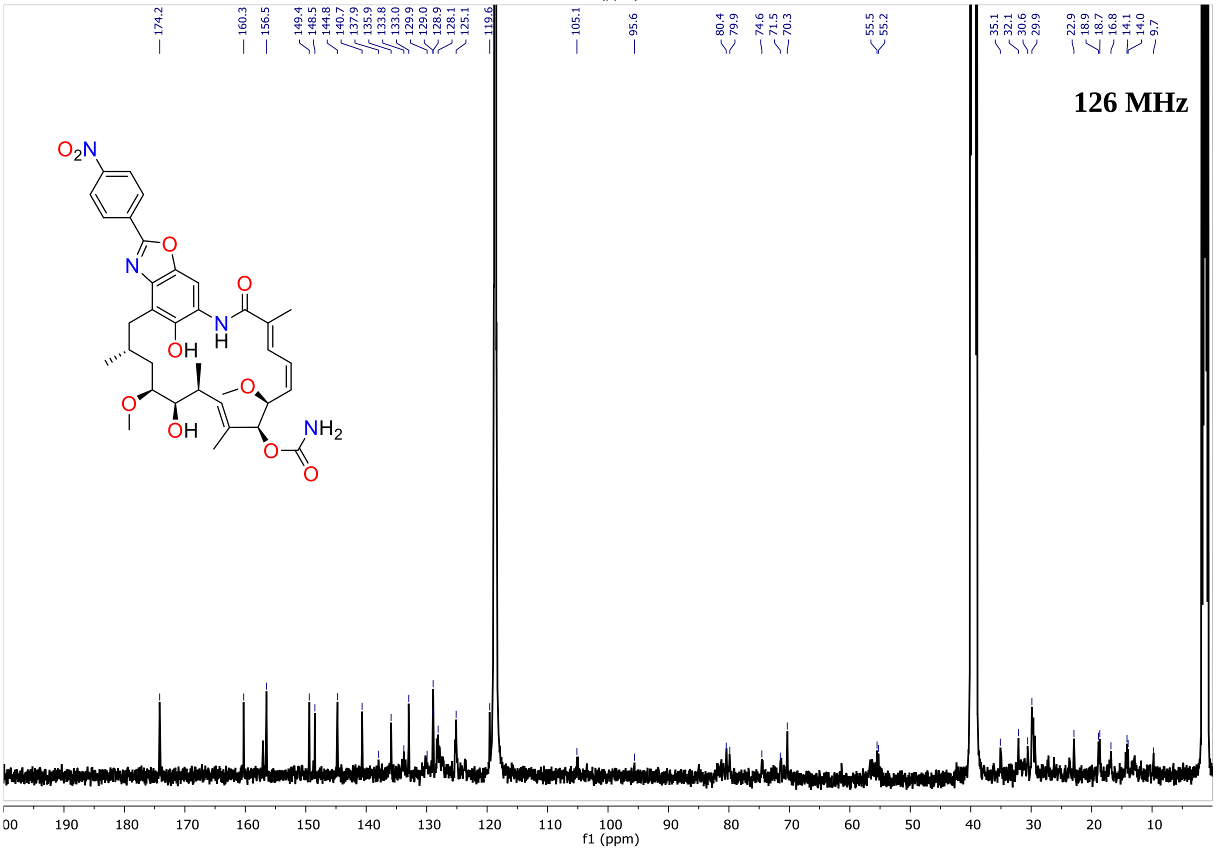

Figure S63 ^{1}H and ^{13}C{1H} spectra of compound **9a** in DMSO-d_6+ ACN-d$_3$.

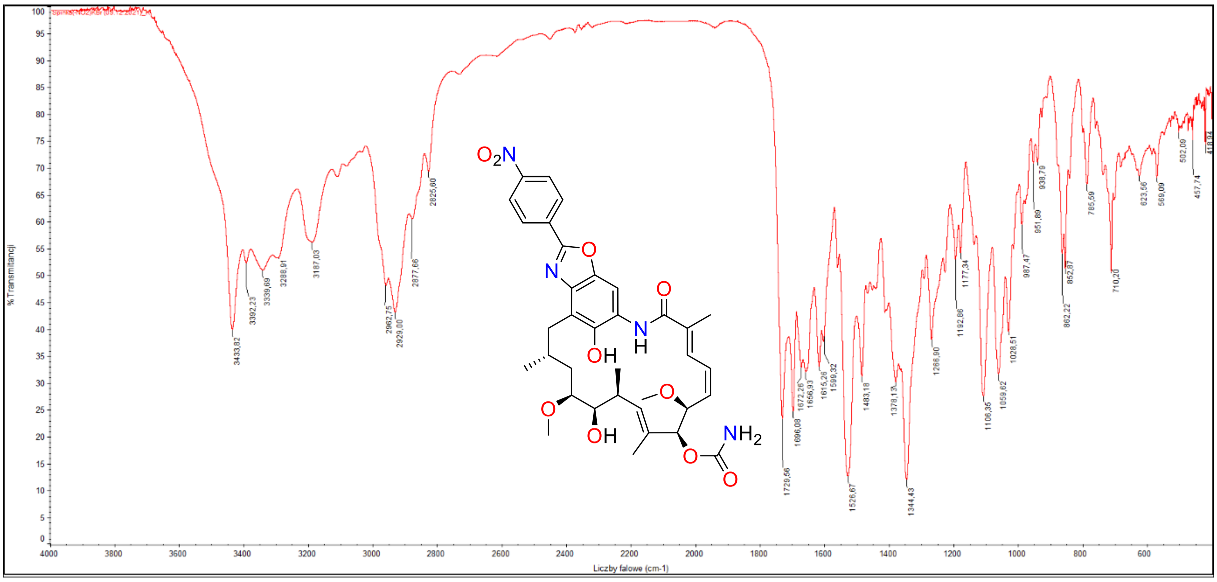

Figure S64 FT-IR spectrum of compound **9a** (in KBr).

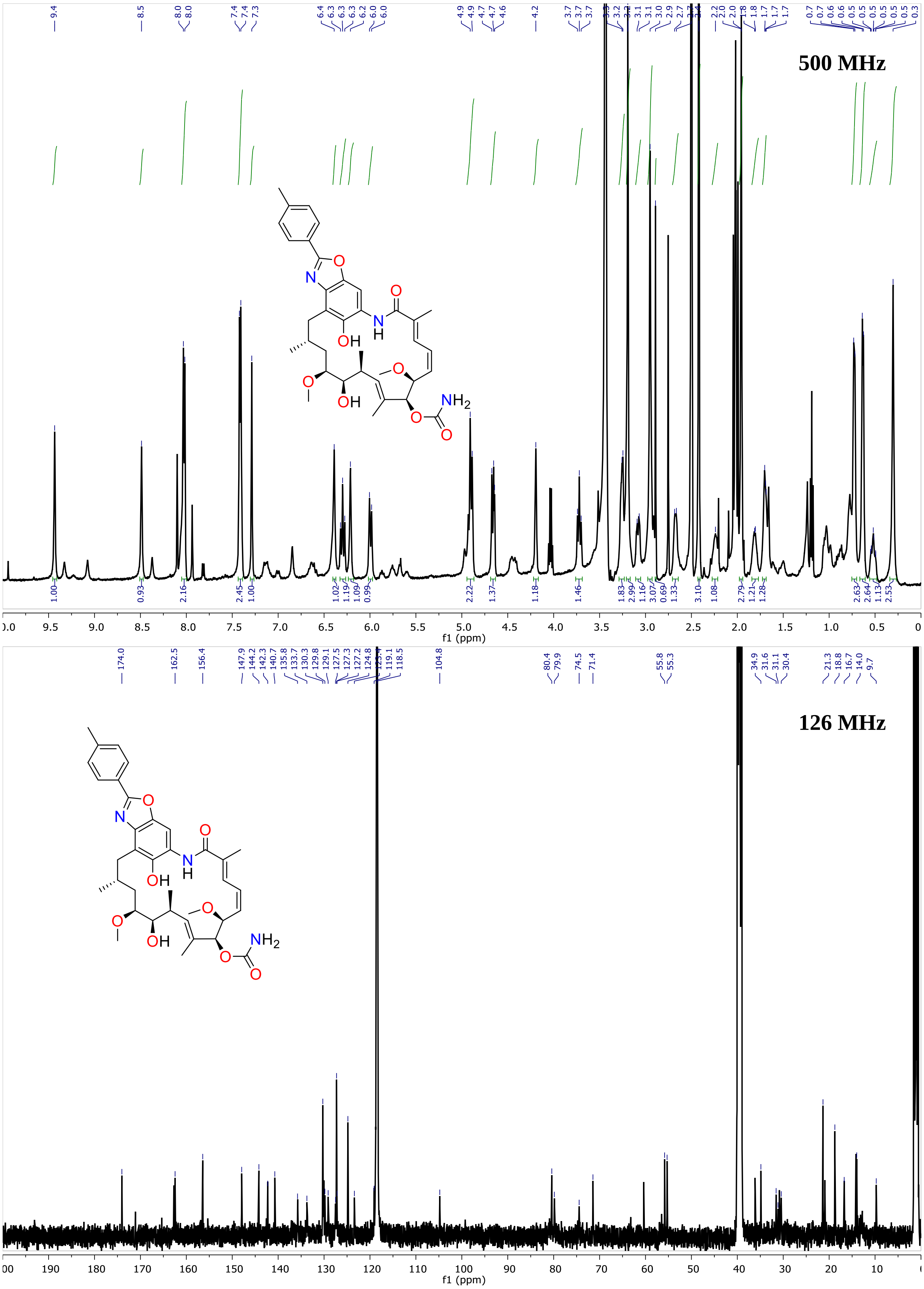

Figure S65 ^{1}H and ^{13}C{1H} spectra of compound **10a** in DMSO-*d*$_6$+ ACN-d$_3$.

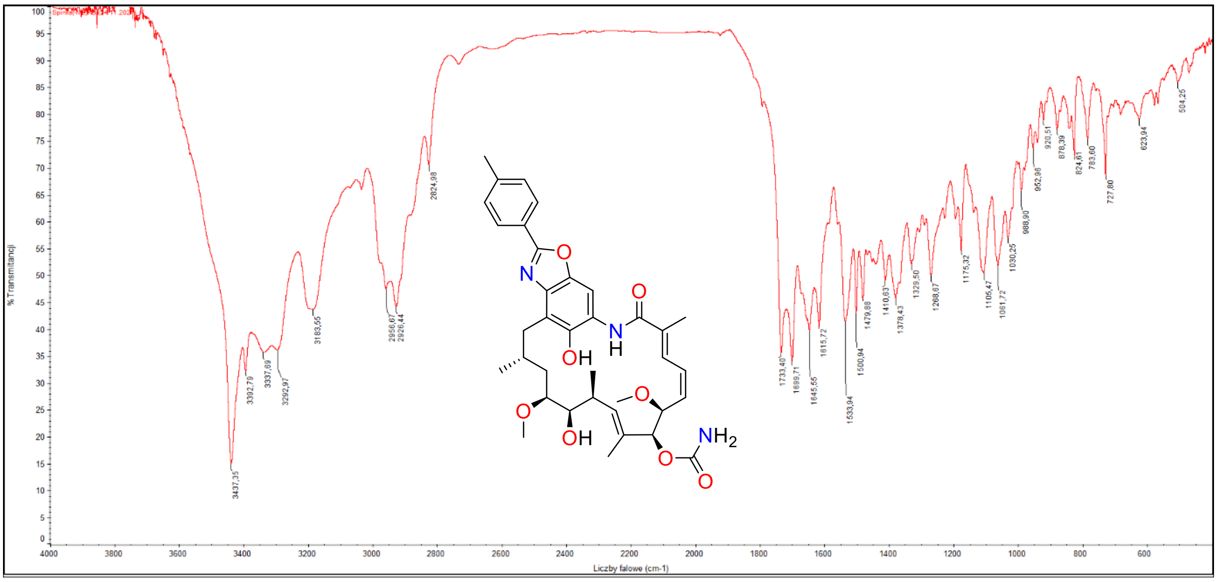

Figure S66 FT-IR spectrum of compound **10a** (in KBr).

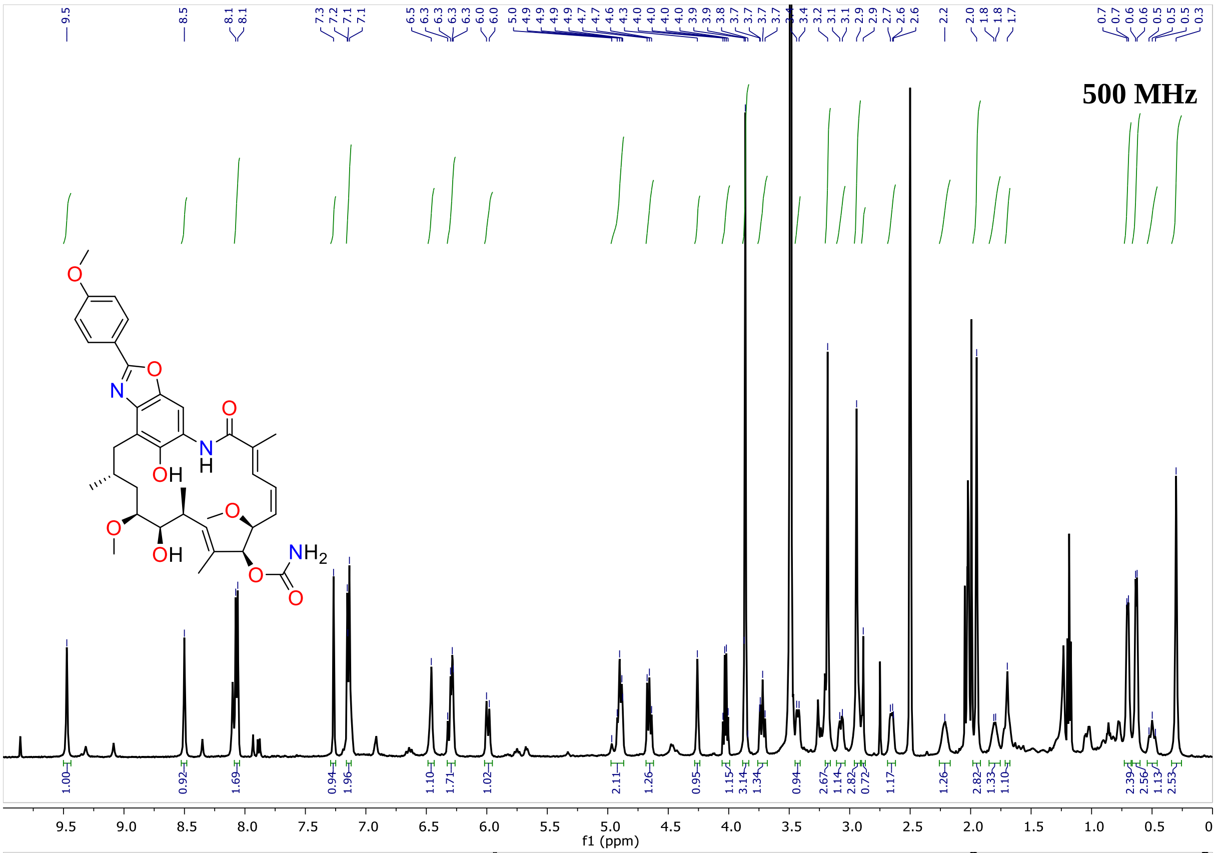

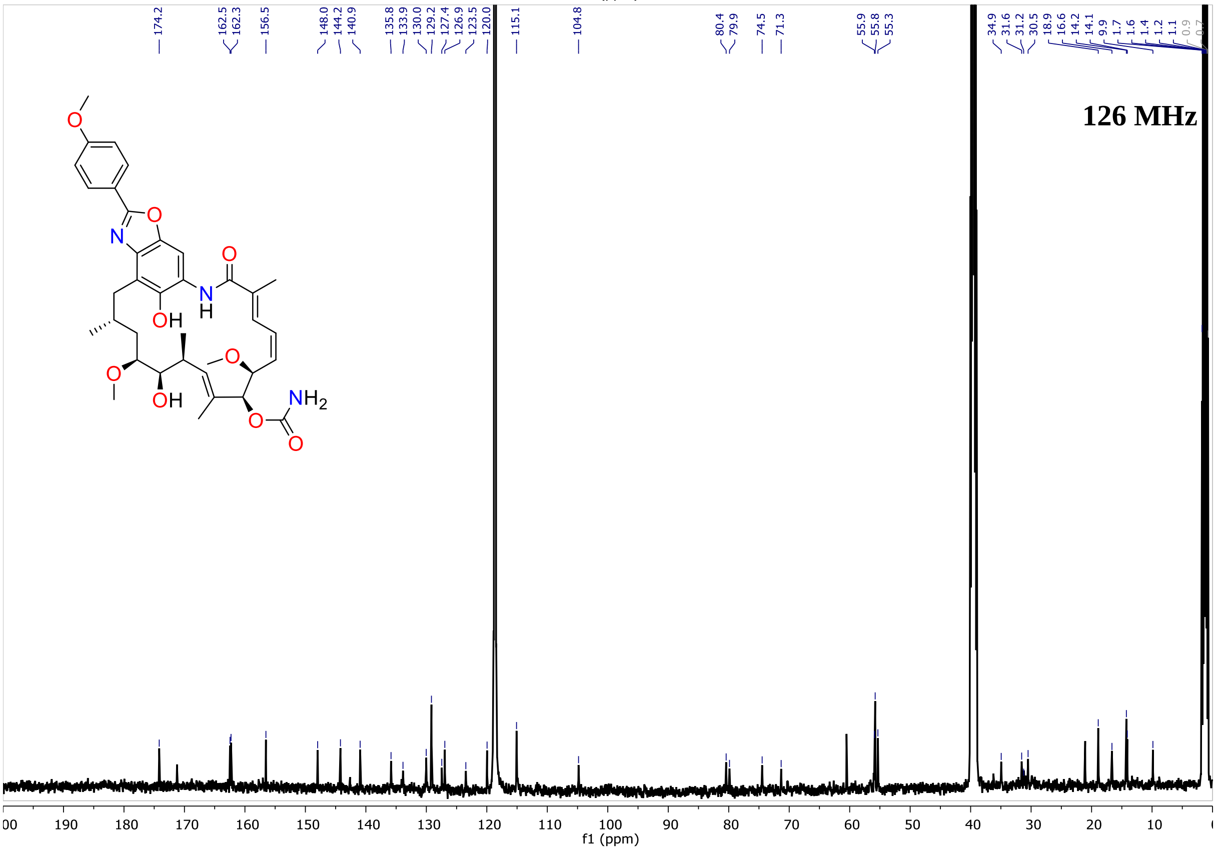

Figure S67 ^{1}H and ^{13}C{1H} spectra of compound **11a** in DMSO-d_6+ ACN-d$_3$.

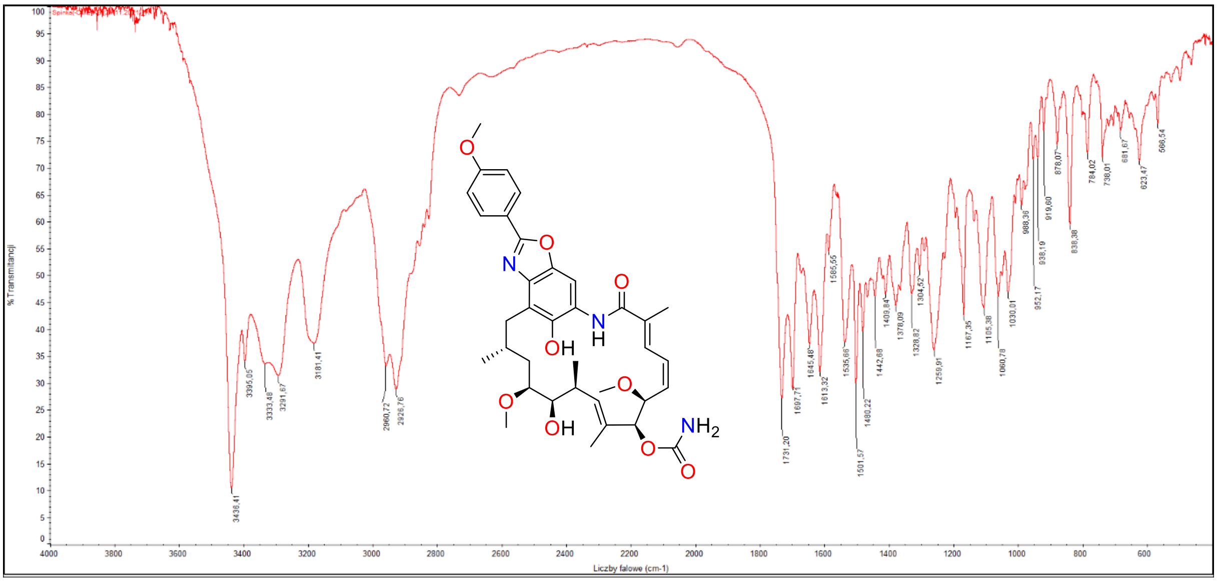

Figure S68 FT-IR spectrum of compound **11a** (in KBr).

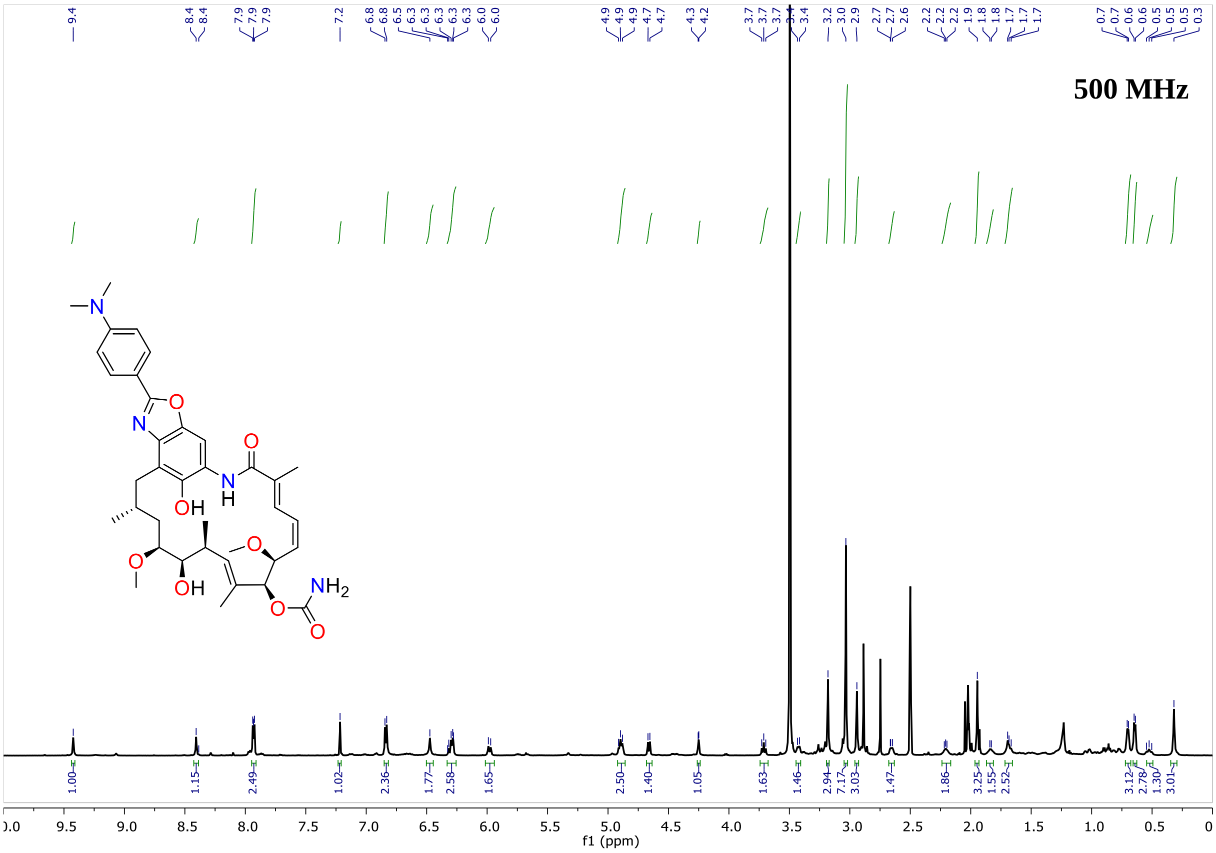

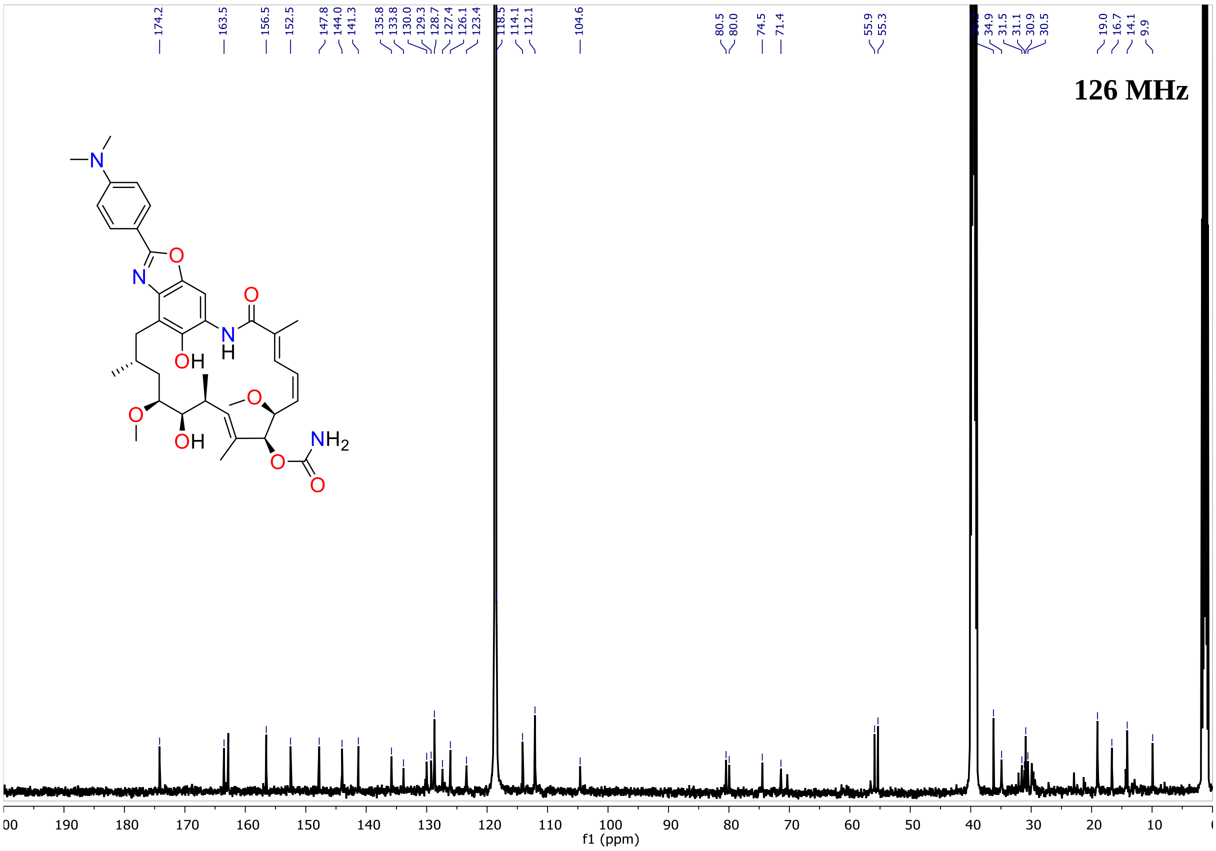

Figure S69 ^{1}H and ^{13}C{1H} spectra of compound **12a** in DMSO-d_6+ ACN-d_3.

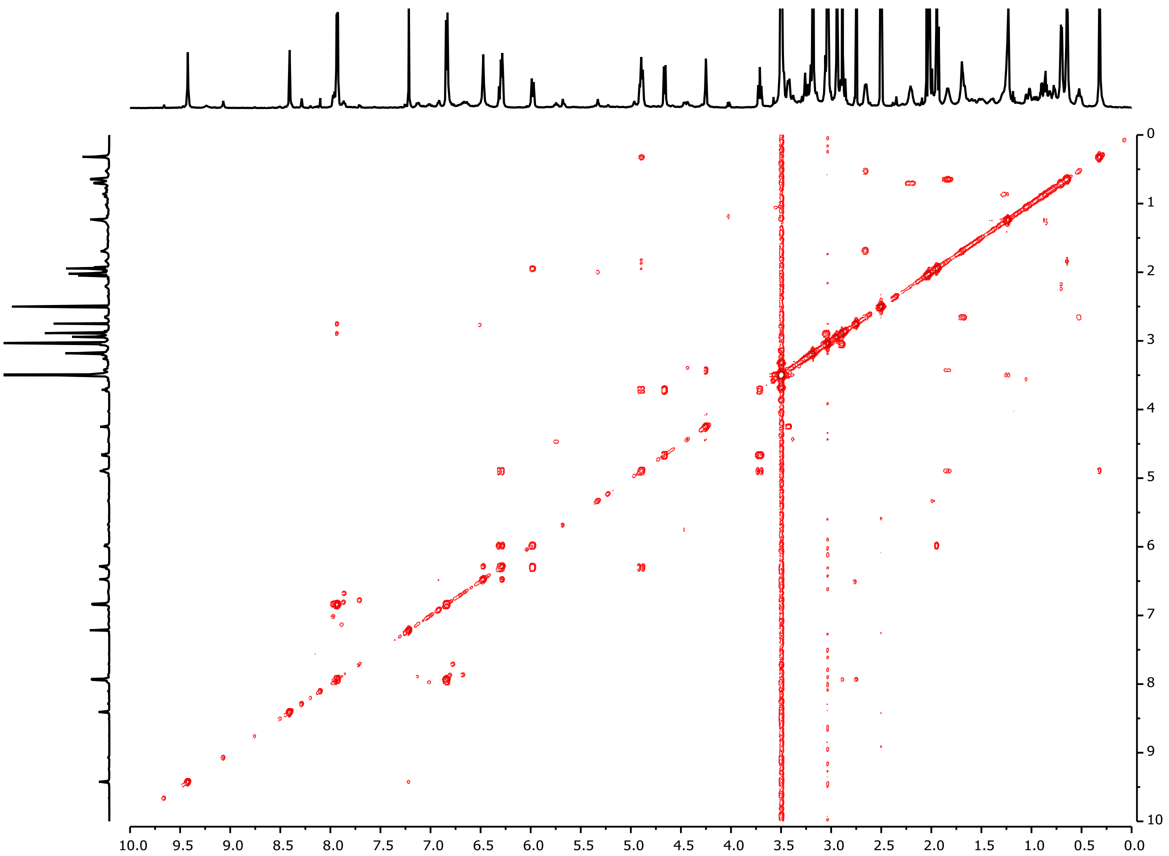

Figure S70 ^{1}H-^{1}H COSY spectrum of compound **12a** in DMSO-d_6+ ACN-d_3.

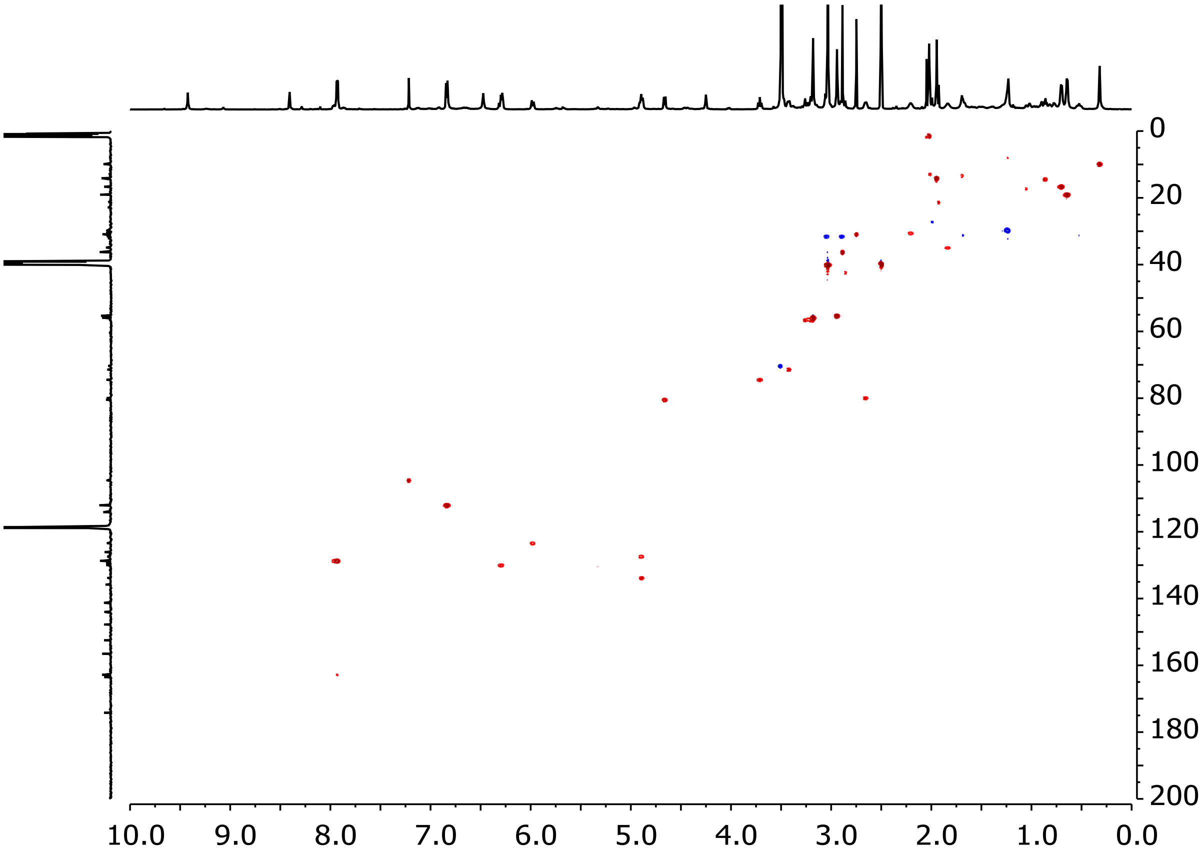

Figure S71 ^{1}H-^{13}C HSQC spectrum of compound **12a** in DMSO-d_6+ ACN-d_3.

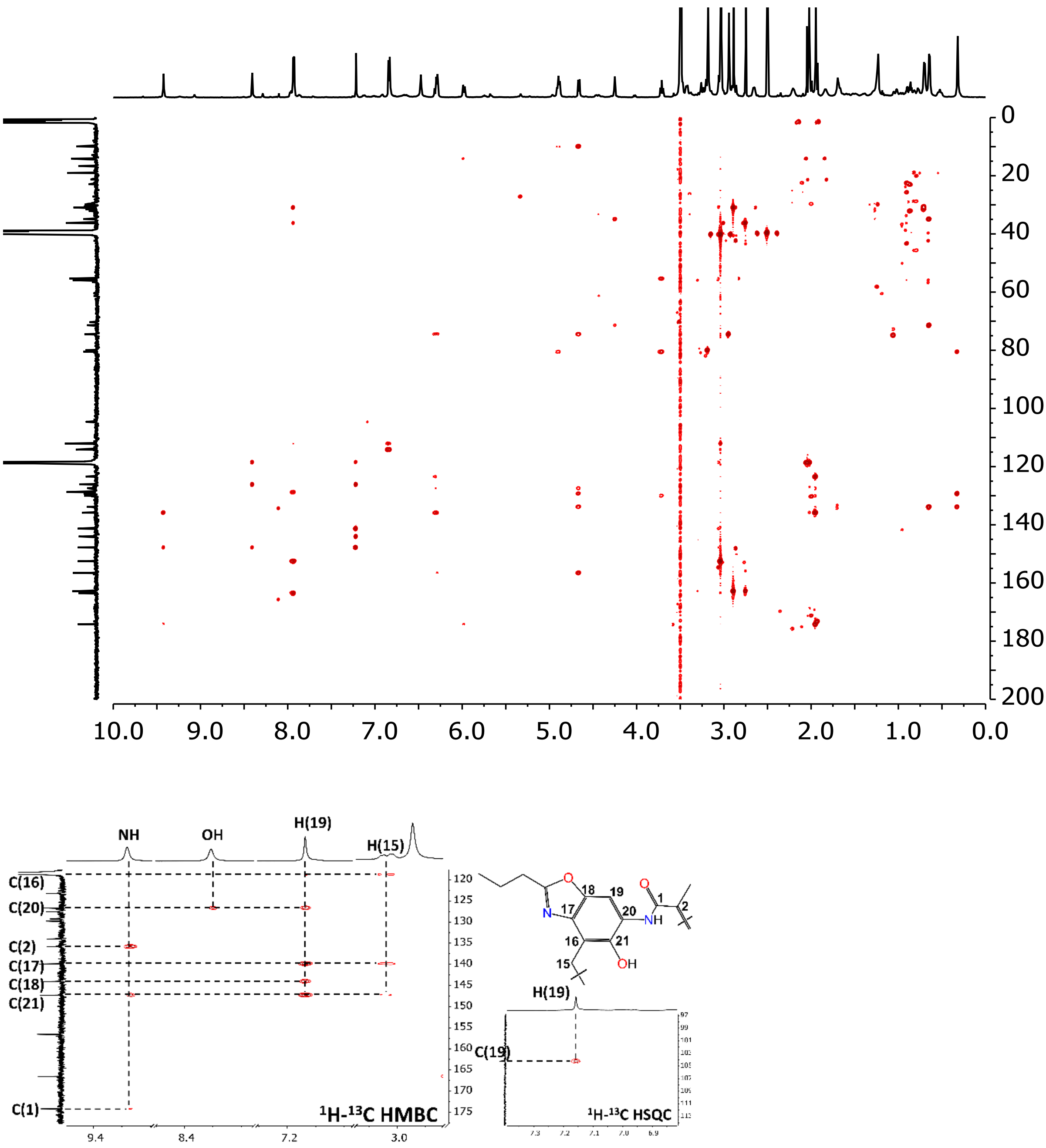

Figure S72 ^{1}H-^{13}C HMBC spectra of compound **12a** in DMSO-d_6+ ACN-d_3.

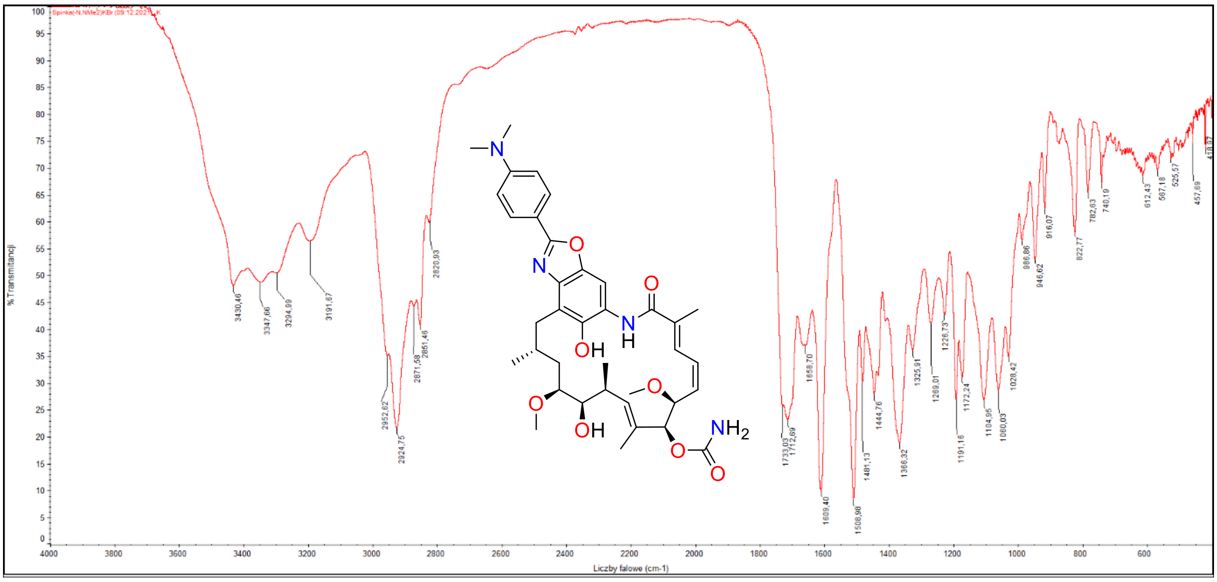

Figure S73 FT-IR spectrum of compound **12a** (in KBr).

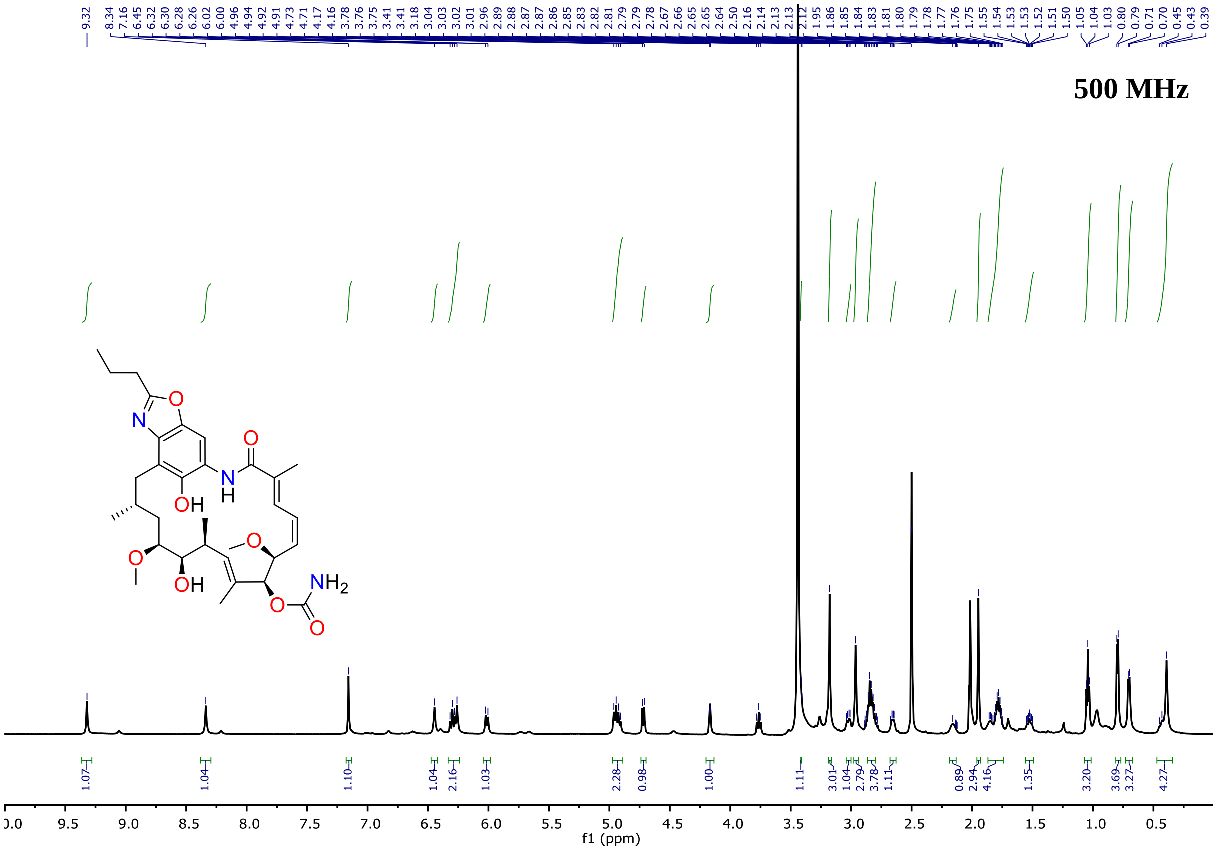

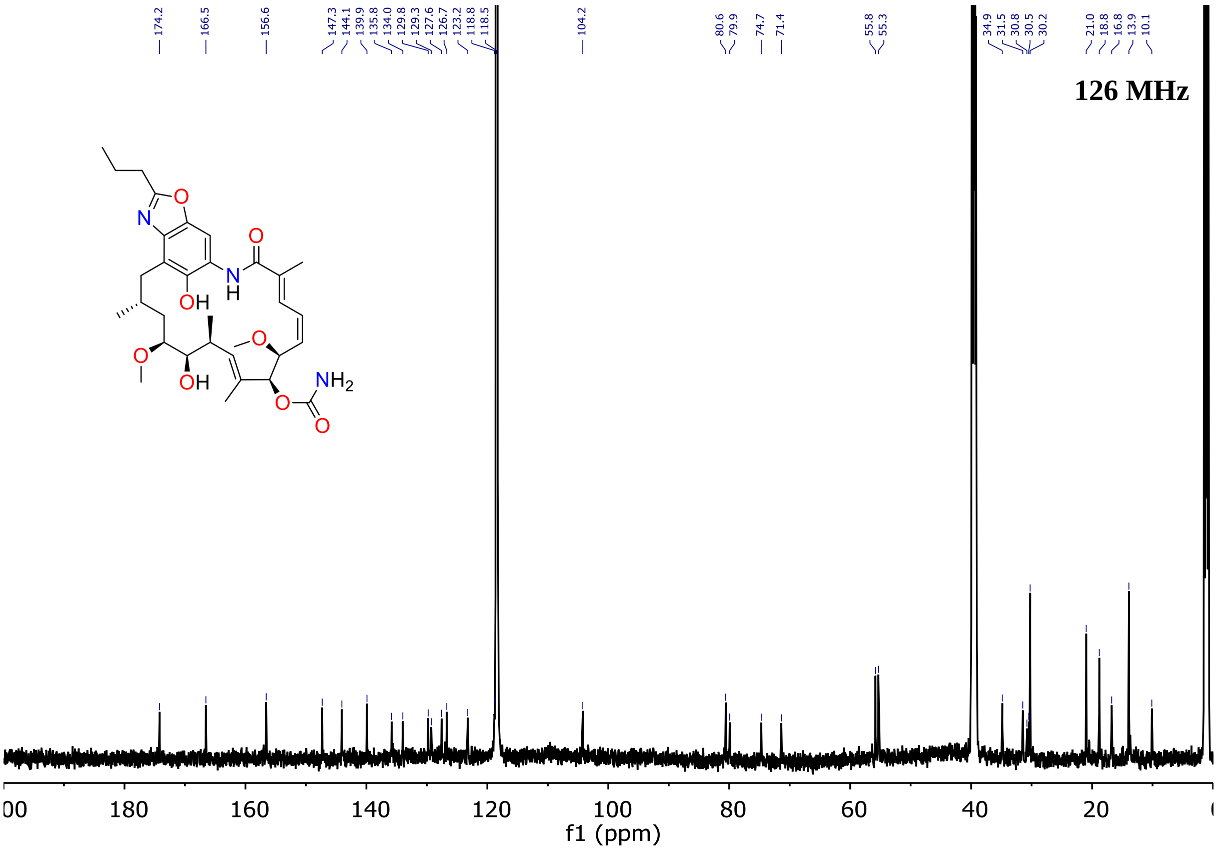

Figure S74 ^{1}H and $^{13}C\{1H\}$ spectra of compound **13a** in DMSO-d_6+ ACN-d_3.

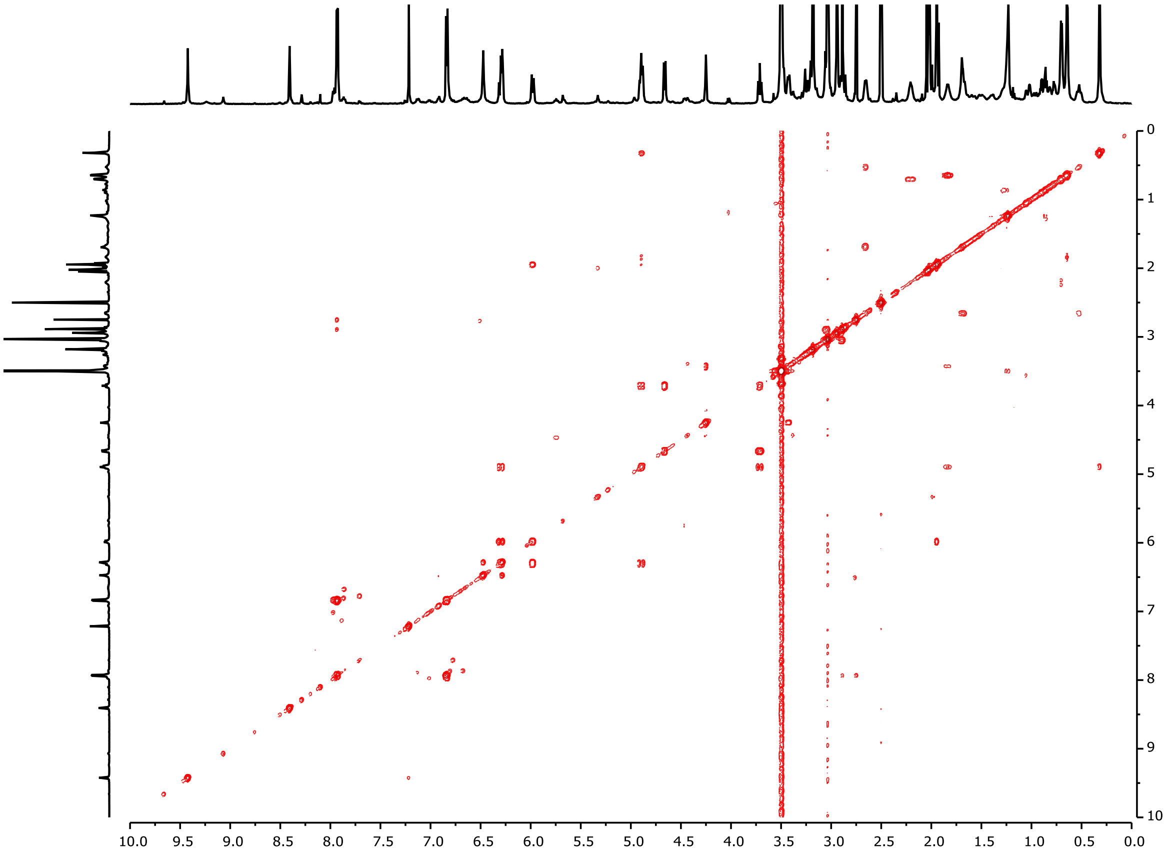

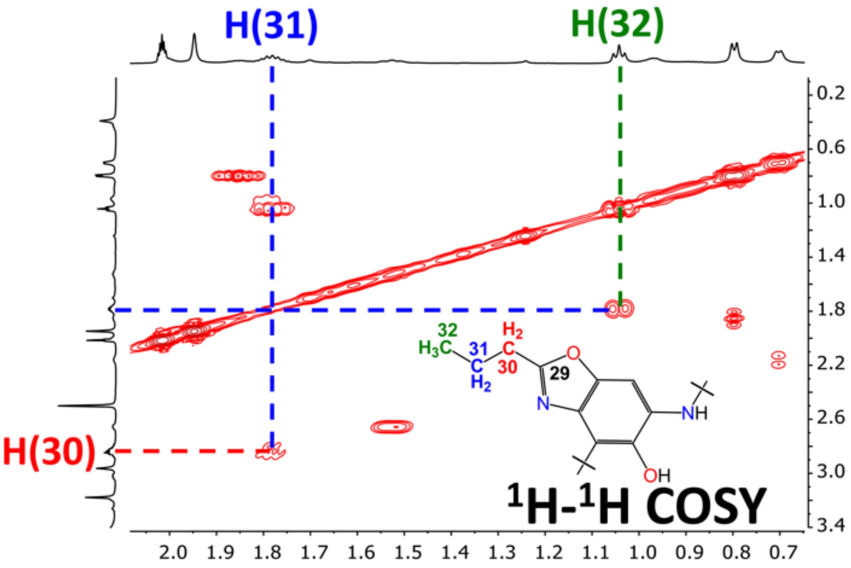

Figure S75 ^{1}H-^{1}H COSY spectra of compound **13a** in DMSO-d_6+ ACN-d_3.

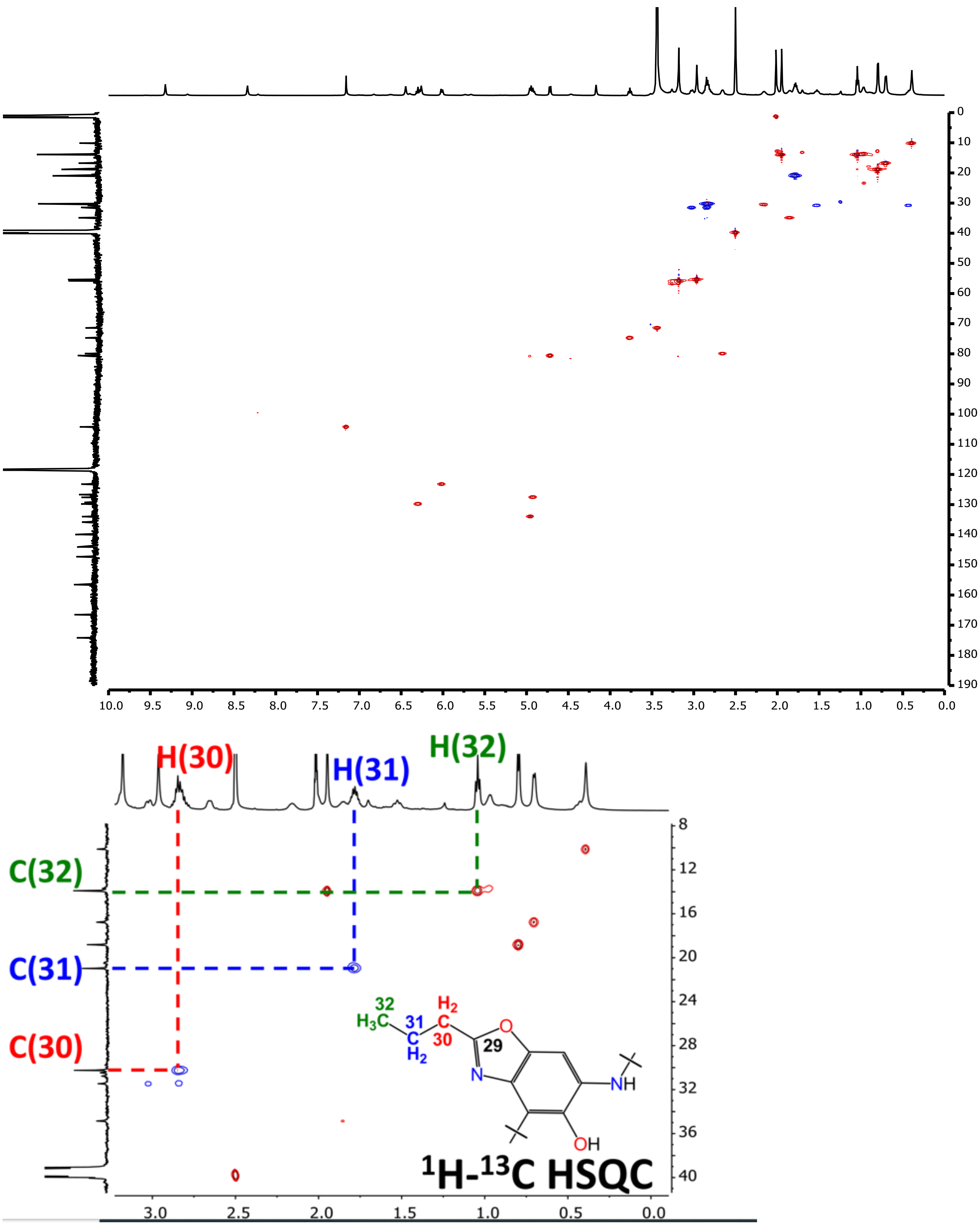

Figure S76 ^{1}H-^{13}C HSQC spectra of compound **13a** in DMSO-d_6+ ACN-d_3.

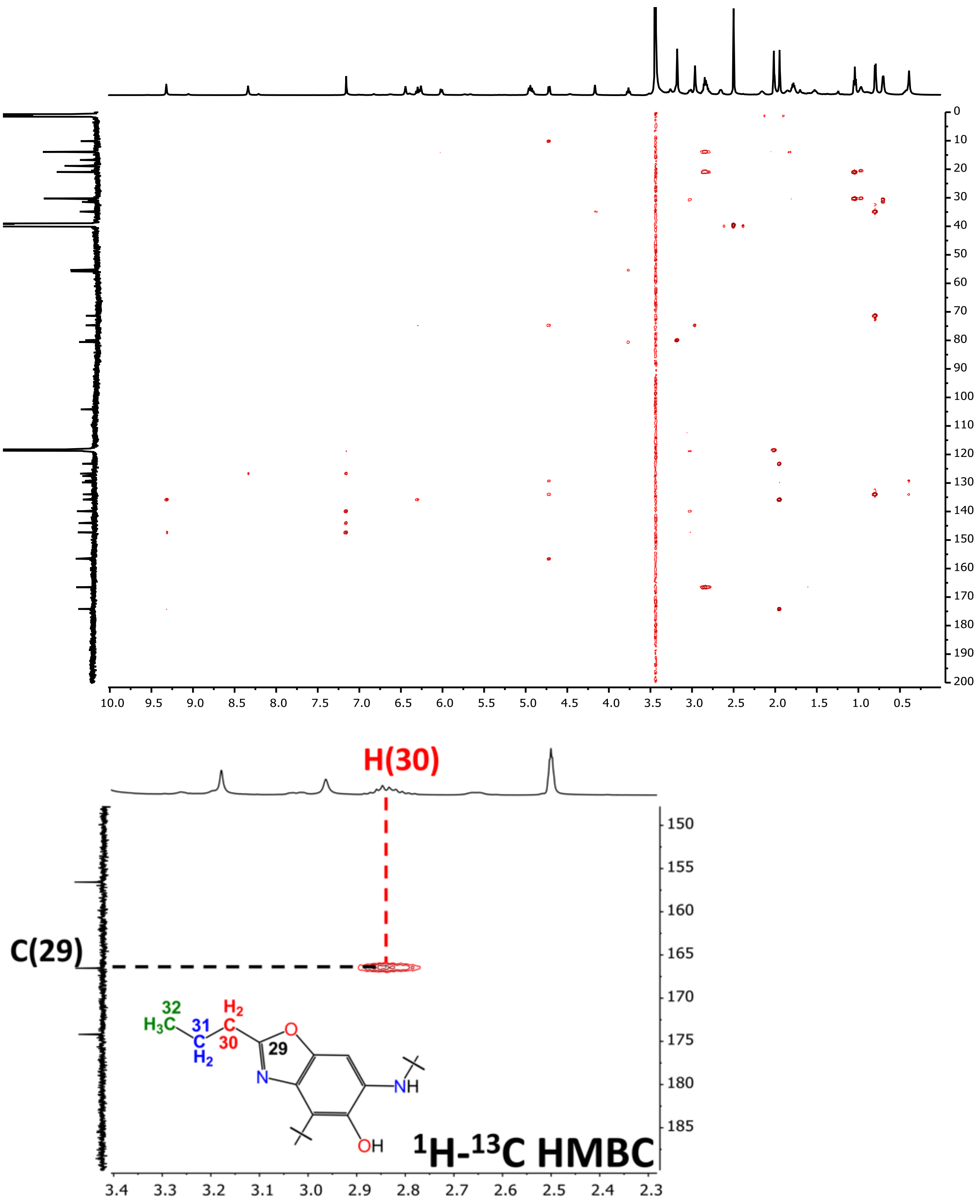

Figure S77 ^1H-^{13}C HMBC spectra of compound **13a** in DMSO-d_6+ ACN-d_3.

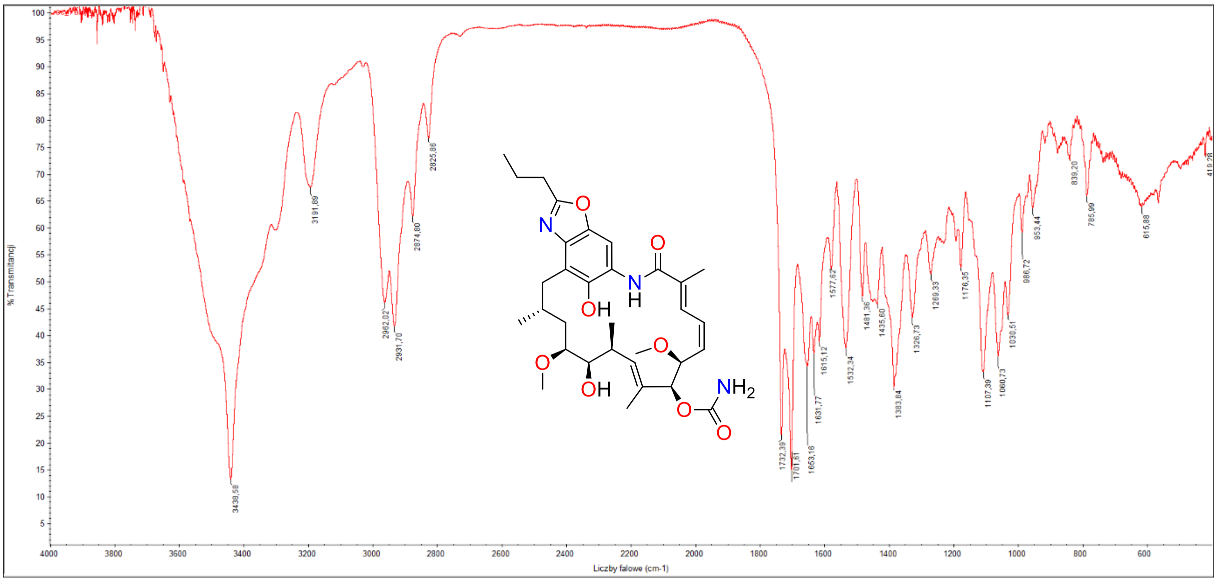

Figure S78 FT-IR spectrum of compound **13a** (in KBr).

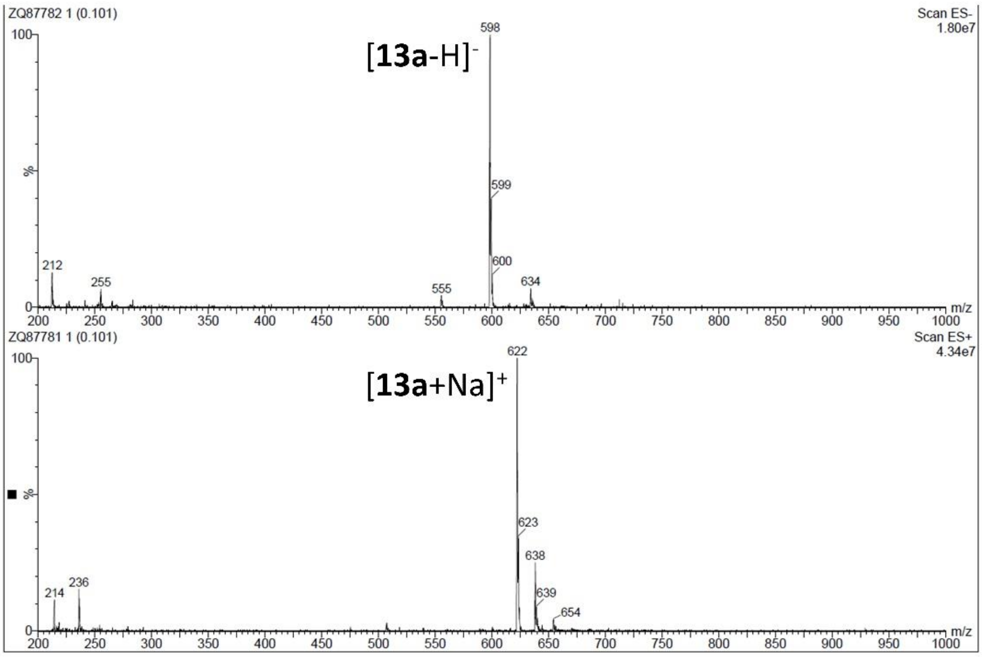

Figure S79 ESI-MS spectrum of compound **13a**.

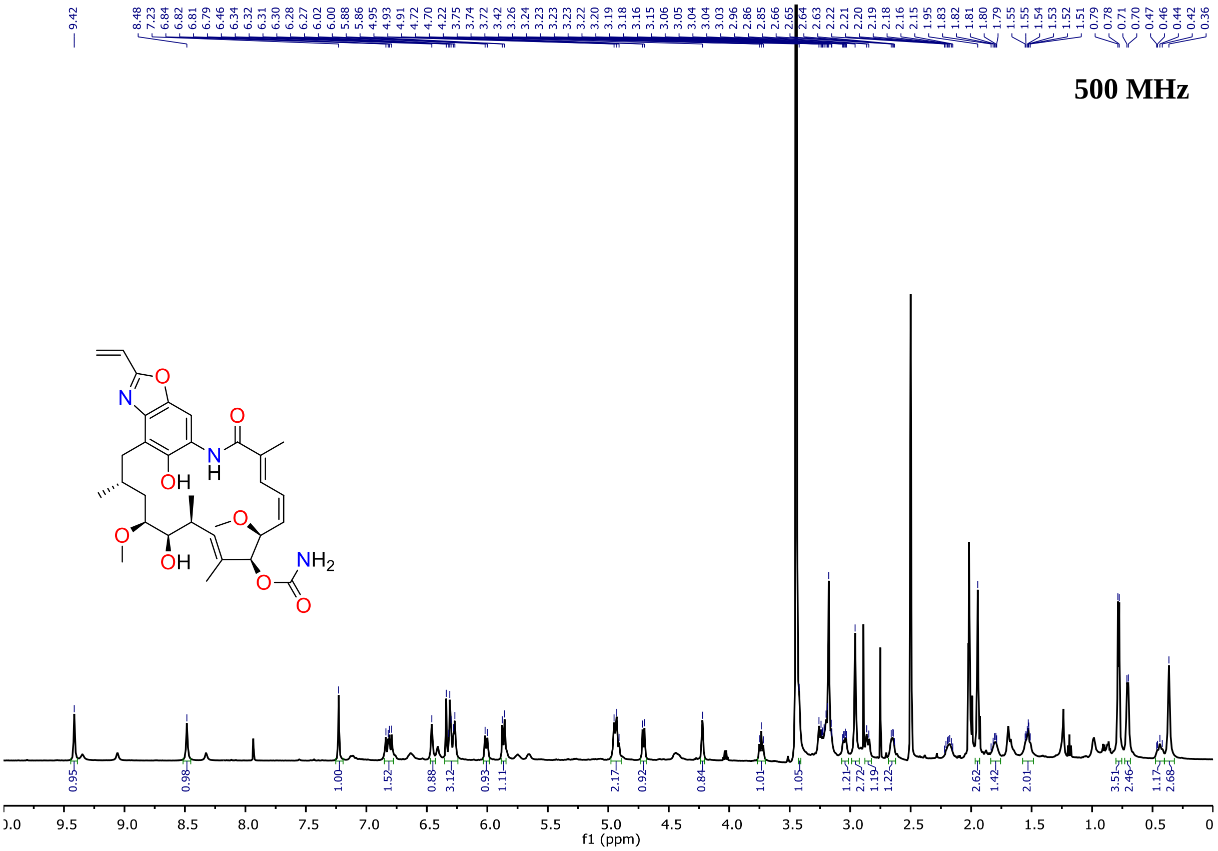

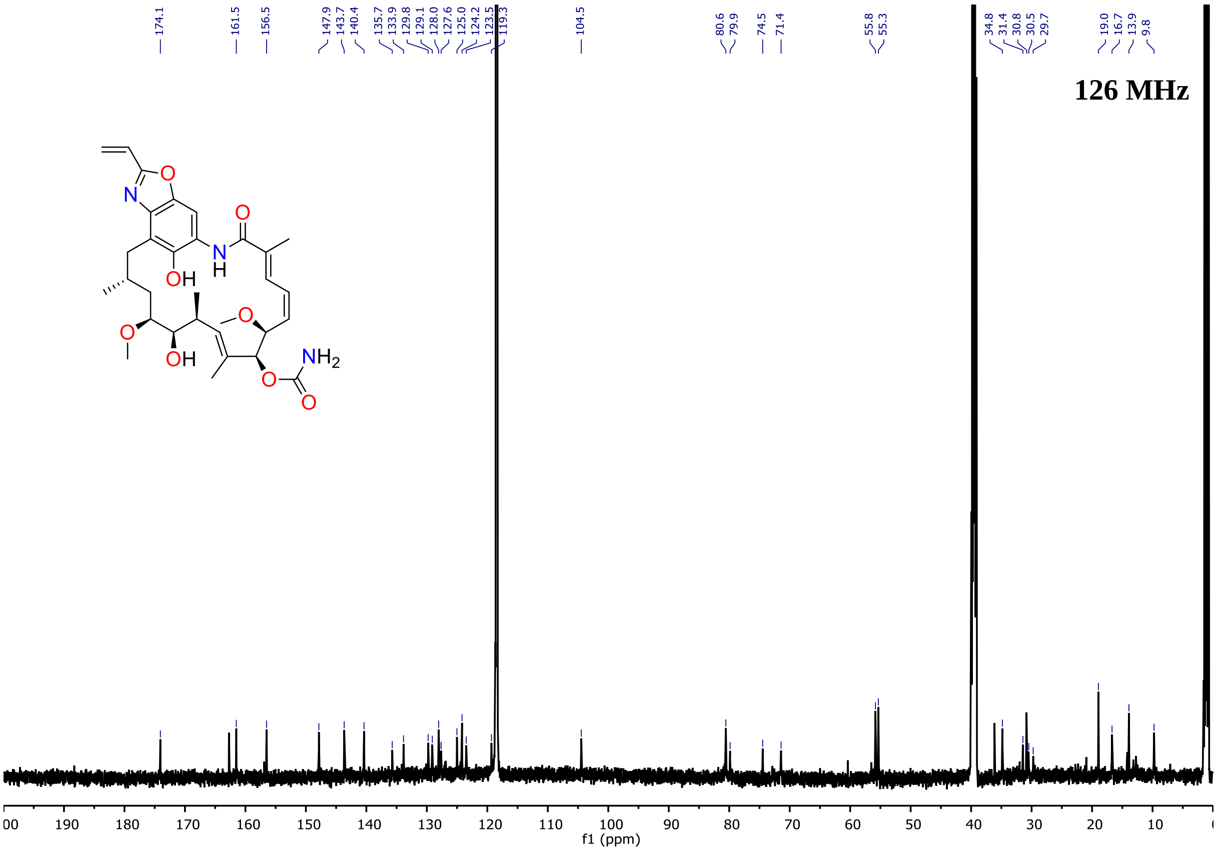

Figure S80 ^{1}H and $^{13}C\{1H\}$ spectra of compound **14a** in DMSO-d_6+ ACN-d_3.

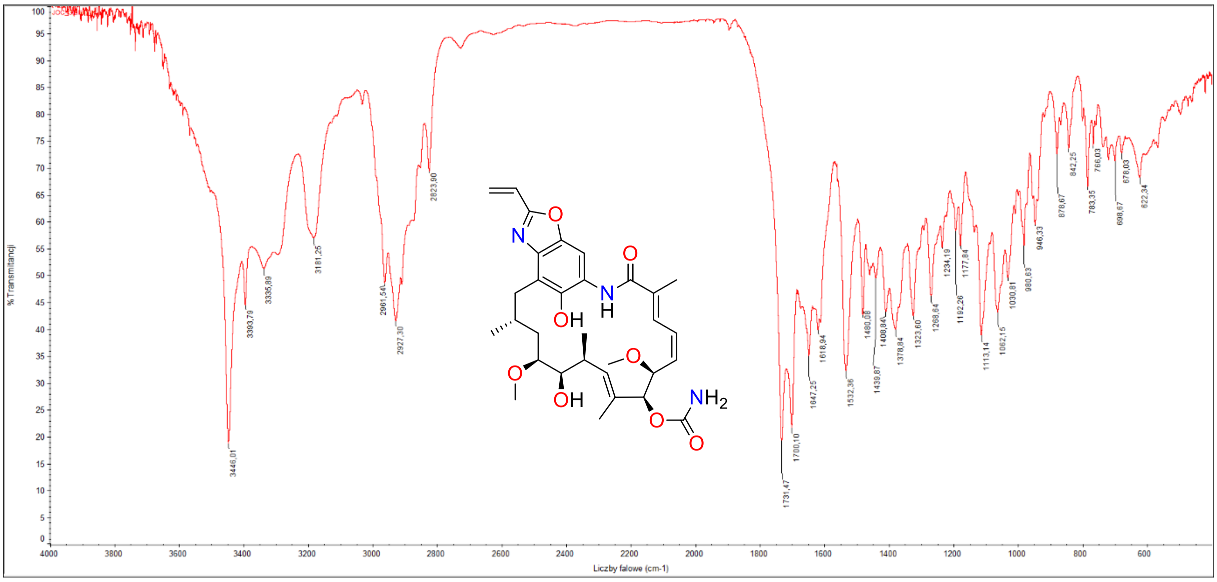

Figure S81 FT-IR spectrum of compound **14a** (in KBr).

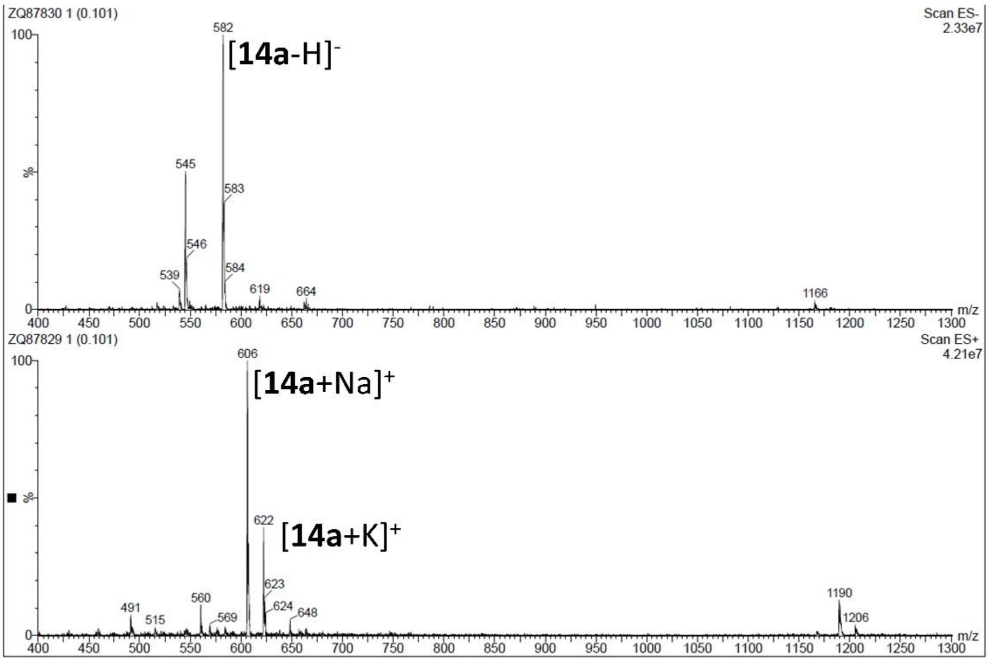

Figure S82 ESI-MS spectrum of compound **14a**.

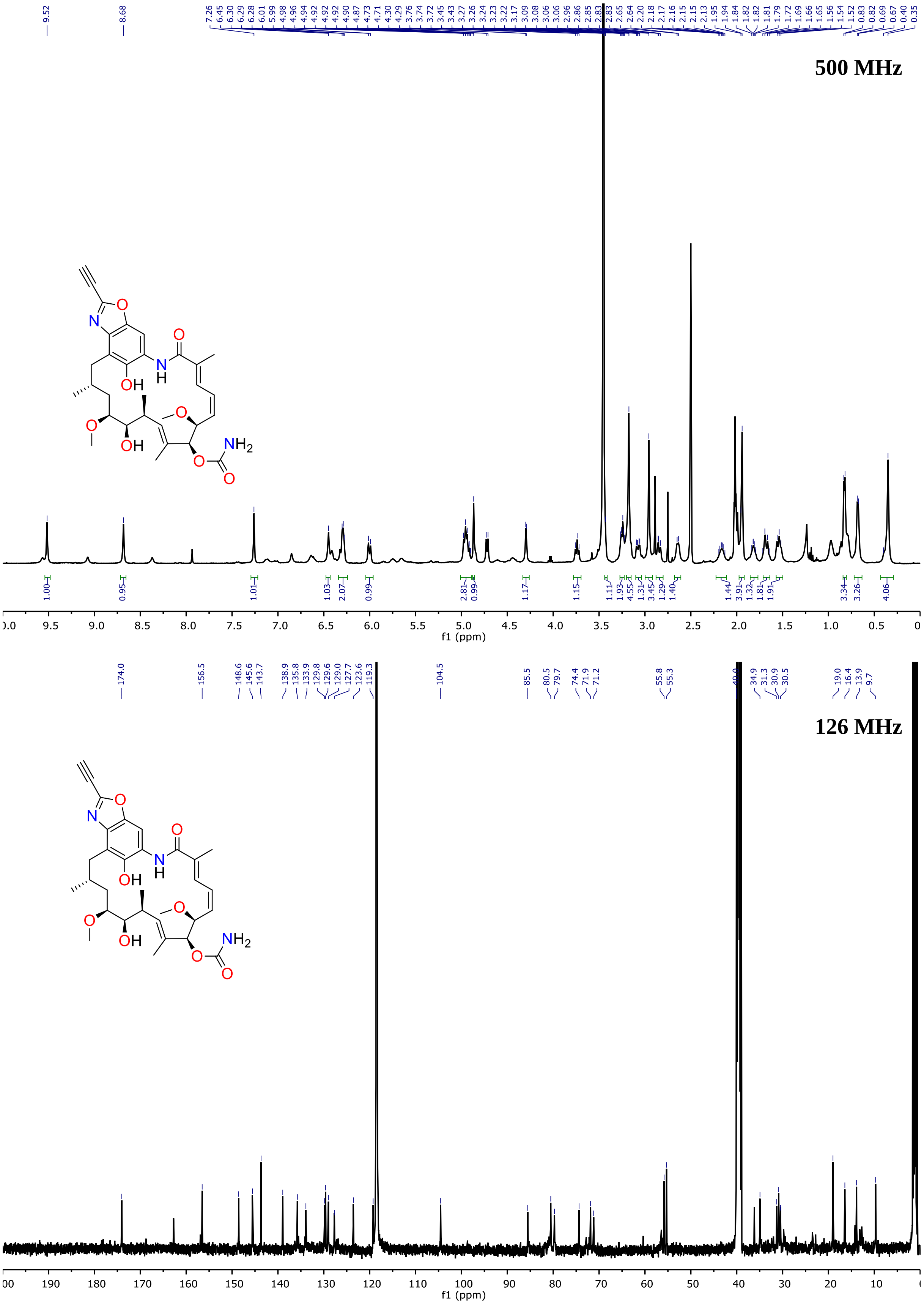

Figure S83 ^{1}H and $^{13}C\{1H\}$ spectra of compound **15a** in DMSO-d_6+ ACN-d_3.

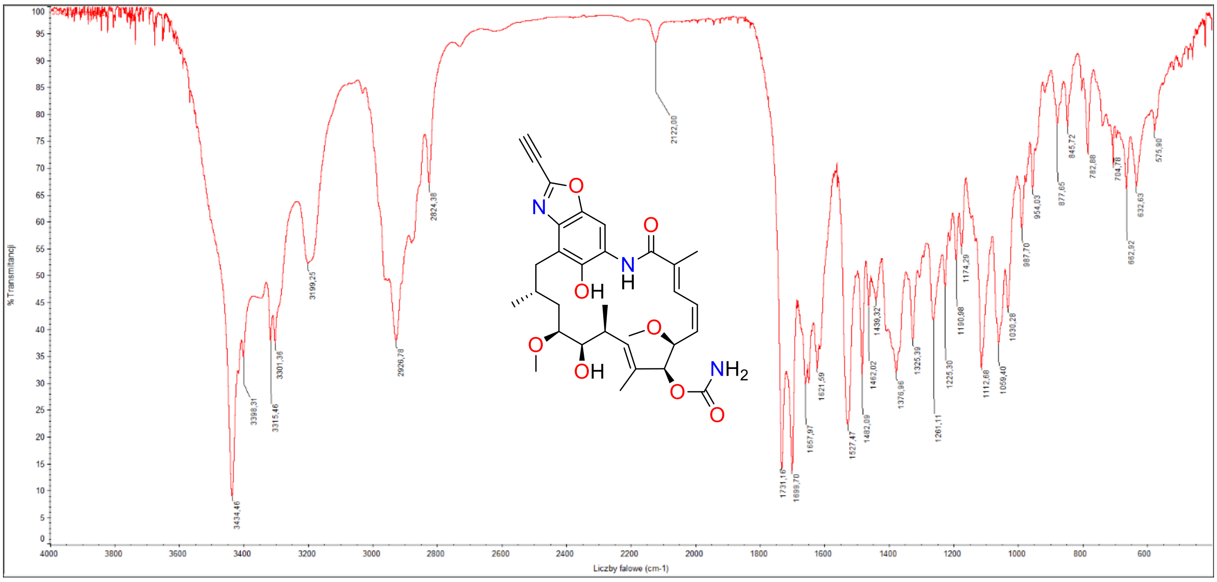

Figure S84 FT-IR spectrum of compound **15a** (in KBr).

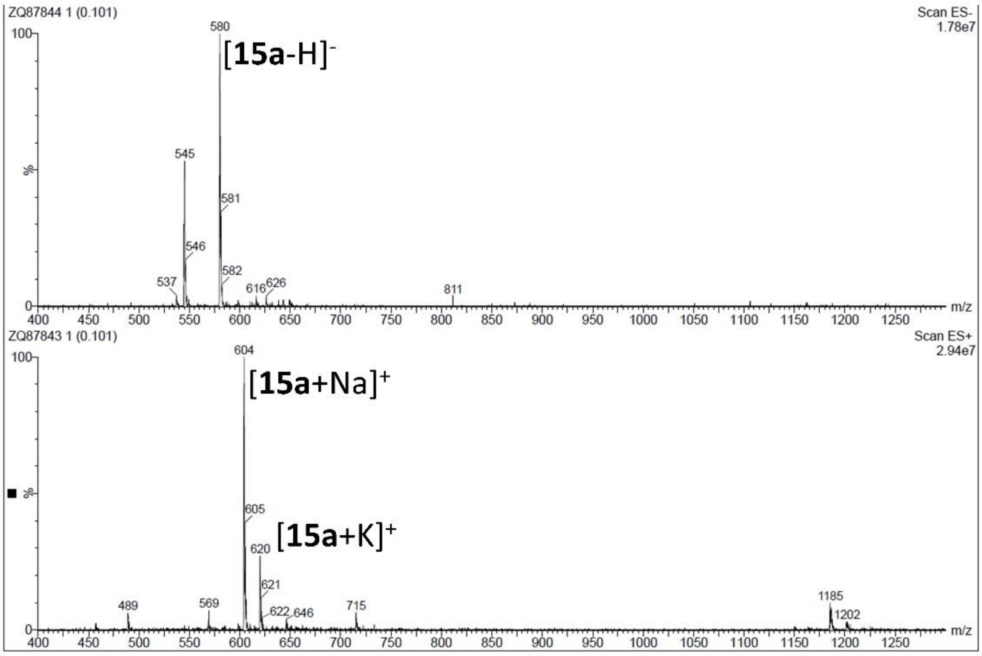

Figure S85 ESI-MS spectrum of compound **15a**.

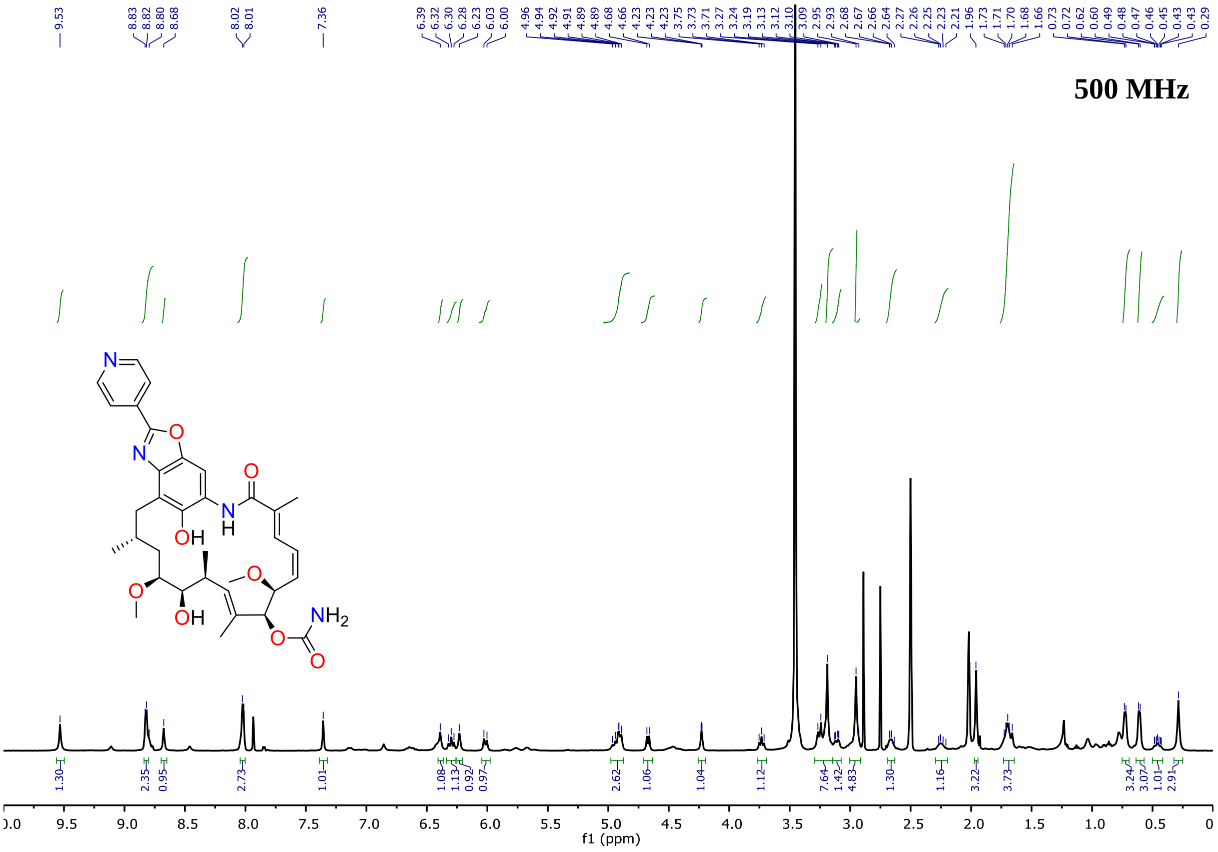

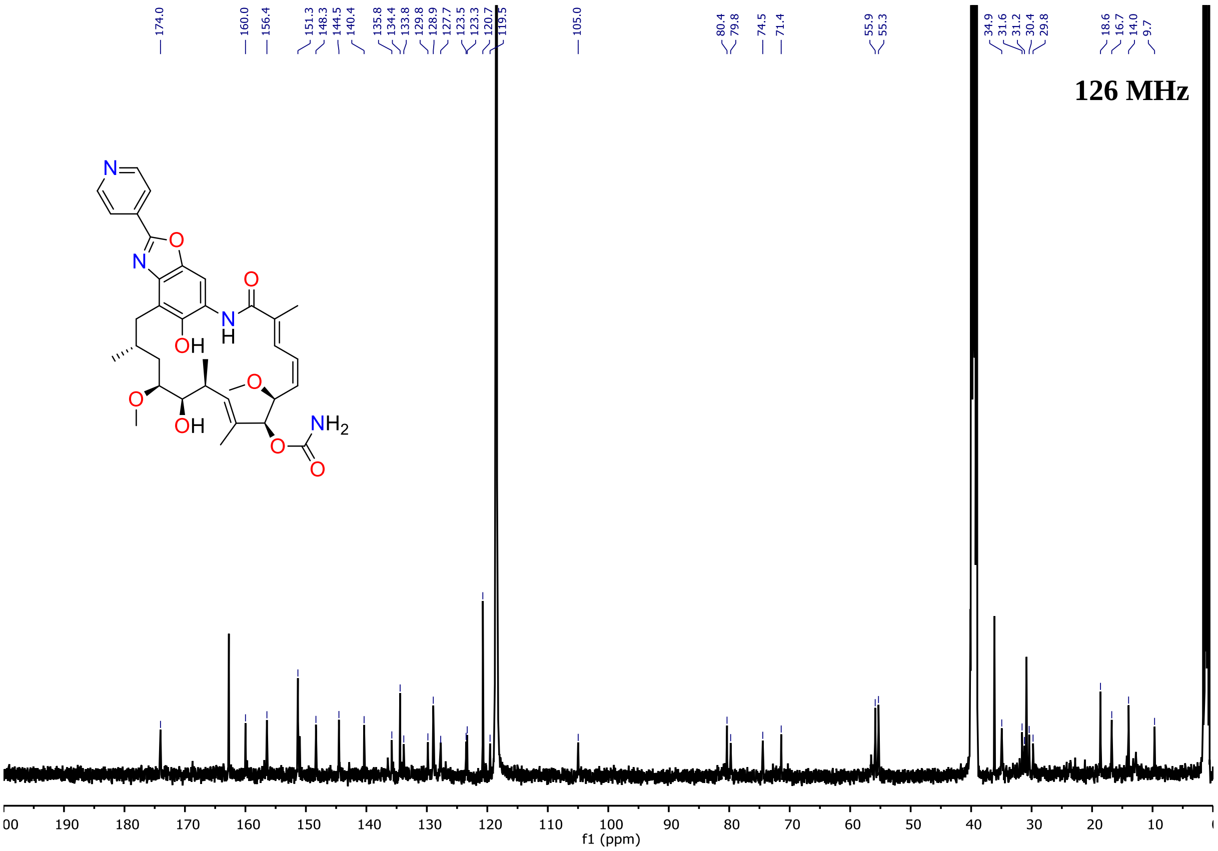

Figure S86 ^{1}H and $^{13}C\{1H\}$ spectra of compound **16a** in DMSO-d_6+ ACN-d_3.

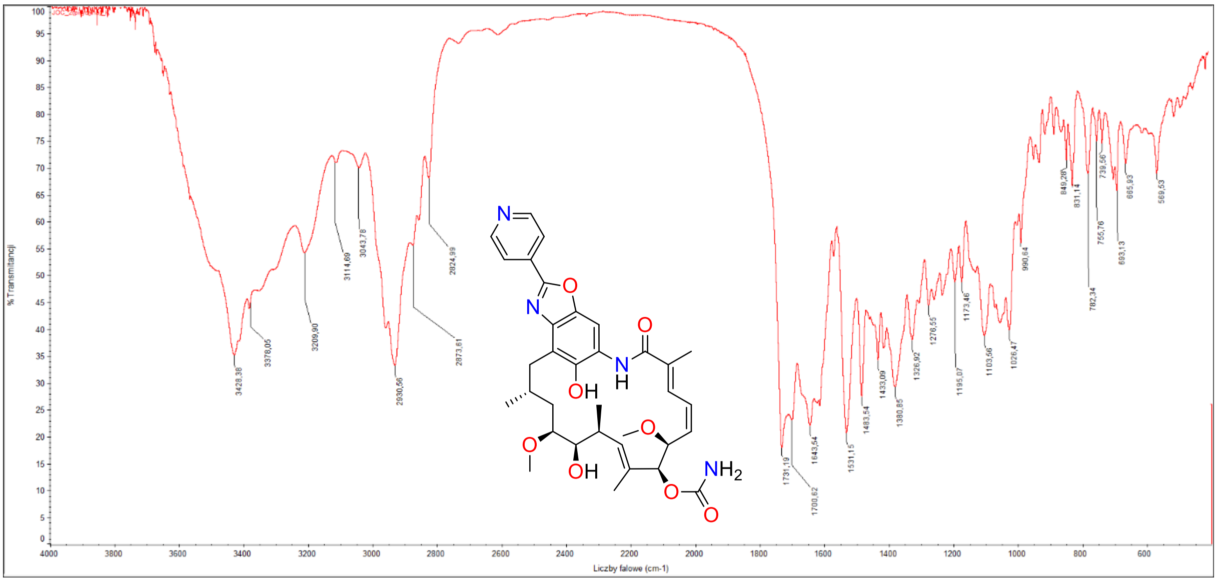

Figure S87 FT-IR spectrum of compound **16a** (in KBr).

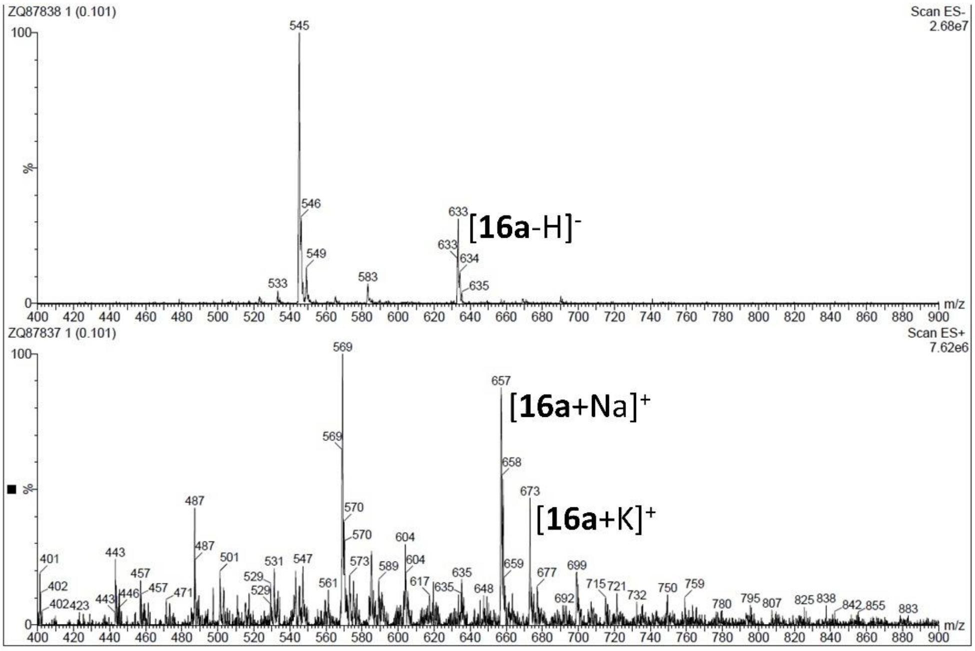

Figure S88 ESI-MS spectrum of compound **16a**.

References:

(1) Rigaku Oxford Diffraction, 2020.

(2) Sheldrick, G. M. SHELXT – Integrated Space-Group and Crystal-Structure Determination. *Acta Cryst A* **2015**, *71* (1), 3–8. https://doi.org/10.1107/S2053273314026370.

(3) Sheldrick, G. M. Crystal Structure Refinement with *SHELXL*. *Acta Crystallogr C Struct Chem* **2015**, *71* (1), 3–8. https://doi.org/10.1107/S2053229614024218.

(4) Dolomanov, O. V.; Bourhis, L. J.; Gildea, R. J.; Howard, J. A. K.; Puschmann, H. *OLEX2* : A Complete Structure Solution, Refinement and Analysis Program. *J Appl Crystallogr* **2009**, *42* (2), 339–341. https://doi.org/10.1107/S0021889808042726.

(5) SwissADME Http://Www.Swissadme.Ch/Index.Php.

(6) Daina, A.; Michielin, O.; Zoete, V. ILOGP: A Simple, Robust, and Efficient Description of n-Octanol/Water Partition Coefficient for Drug Design Using the GB/SA Approach. *J. Chem. Inf. Model.* **2014**, *54* (12), 3284–3301. https://doi.org/10.1021/ci500467k.

(7) Skrzypczak, N.; Pyta, K.; Ruszkowski, P.; Gdaniec, M.; Bartl, F.; Przybylski, P. Synthesis, Structure and Anticancer Activity of New Geldanamycin Amine Analogs Containing C(17)- or C(20)- Flexible and Rigid Arms as Well as Closed or Open Ansa-Bridges. *European Journal of Medicinal Chemistry* **2020**, *202*, 112624. https://doi.org/10.1016/j.ejmech.2020.112624.
